# Real-World Study on Chai-Shi-Jie-Du Granules for the Treatment of Dengue Fever and the Possible Mechanisms Based on Network Pharmacology

**DOI:** 10.1155/2023/9942842

**Published:** 2023-08-30

**Authors:** Huiqin Yang, Dehong Ma, Qin Li, Wen Zhou, Hongyi Chen, Xiyun Shan, Haipeng Zheng, Chun Luo, Zhiyue Ou, Jielan Xu, Changtai Wang, Lingzhai Zhao, Rui Su, Yuehong Chen, Qingquan Liu, Xinghua Tan, Luping Lin, Tao Jiang, Fuchun Zhang

**Affiliations:** ^1^Infectious Disease Center, Guangzhou Eighth People's Hospital, Guangzhou Medical University, Guangzhou 510440, Guangdong, China; ^2^Department of Infectious Diseases, The People's Hospital of Xishuangbanna Dai Nationality Autonomous Prefecture, Xishuangbanna 666100, Yunnan, China; ^3^Department of Infectious Diseases, Mengchao Hepatobiliary Hospital of Fujian Medical University, Fuzhou 350025, Fujian, China; ^4^Department of Infectious Diseases, The Ninth Hospital of Nanchang, Nanchang 330002, Jiangxi, China; ^5^Department of Traditional Chinese Medicine, Guangzhou Eighth People's Hospital, Guangzhou Medical University, Guangzhou 510440, Guangdong, China; ^6^Infectious Diseases Institute, Guangzhou Eighth People's Hospital, Guangzhou Medical University, Guangzhou 510440, Guangdong, China; ^7^Department of Clinical Laboratory, Guangzhou Eighth People's Hospital, Guangzhou Medical University, Guangzhou 510440, Guangdong, China; ^8^Scientific Research Department, Capital Medical University Beijing Hospital of Traditional Chinese Medicine, Beijing 100010, China; ^9^State Key Laboratory of Pathogen and Biosecurity, Institute of Microbiology and Epidemiology, AMMS, Beijing100071, China

## Abstract

**Objectives:**

Traditional Chinese medicine (TCM) is a widely used method for treating dengue fever in China. TCM improves the symptoms of patients with dengue, but there is no standard TCM prescription for dengue fever. This real-world study aimed to evaluate the effects of Chai-Shi-Jie-Du (CSJD) granules for the treatment of dengue fever and the underlying mechanisms.

**Methods:**

We implemented a multicenter real-world study, an *in vitro* assay and network pharmacology analysis. Patients from 5 hospitals in mainland China who received supportive western treatment in the absence or presence of CSJD were assigned to the control and CSJD groups between 1 August and 31 December 2019. Propensity score matching (PSM) was performed to correct for biases between groups. The clinical data were compared and analyzed. The antidengue virus activity of CSJD was tested in Syrian baby hamster kidney (BHK) cells using the DENV2-NGC strain. Network pharmacological approaches along with active compound screening, target prediction, and GO and KEGG enrichment analyses were used to explore the underlying molecular mechanisms.

**Results:**

137 pairs of patients were successfully matched according to age, sex, and the time from onset to presentation. The time to defervescence (1.7 days vs. 2.5 days, *P* < 0.05) and the disease course (4.1 days vs. 6.1 days, *P* < 0.05) were significantly shorter in the CSJD group than those in the control group. CSJD showed no anti-DENV2-NGC virus activity in BHK cells. Network pharmacology analysis revealed 108 potential therapeutic targets, and the top GO and KEGG terms were related to immunity, oxidative stress response, and the response to lipopolysaccharide.

**Conclusions:**

CSJD granules exhibit high potential for the treatment of dengue fever, and the therapeutic mechanisms involved could be related to regulating immunity, moderating the oxidative stress response, and the response to lipopolysaccharide.

## 1. Introduction

Dengue fever is one of the most prevalent mosquito-borne infectious diseases globally; it is endemic in more than 100 countries and has an incidence of 400 million infections per year [1, 2]. In 2019, it was reported to be one of the top ten threats to global health according to the World Health Organization. In China, the annual number of dengue cases showed an increasing trend from 1990 to 2019 and peaked in 2014 with 46,864 cases [3]. Currently, treatments for dengue infection focus on hastening symptom resolution, as antiviral agents cannot be used to combat the dengue virus [2, 4]. Only one type of dengue vaccine is available for use in a few areas around the world. Most dengue vaccines are still under development or in the preclinical experimental stage, so no widely accepted vaccines are currently available [5, 6].

Traditional Chinese medicine (TCM) may provide an alternative treatment option for dengue. First, TCM has been used in China for thousands of years to treat warm diseases, which refer to acute infectious diseases. To date, TCM is still extensively used by Chinese people and encouraged by the Chinese government, especially after the outbreak of COVID-19. WHO has recognized and promoted TCM as safe and effective for use in existing healthcare systems [7]. Second, many types of research studies on TCM have shown its effectiveness, including RCTs [8, 9], and drugs, such as artemisinin, have been extracted from herbs. However, there is no widely accepted TCM formula or single herb medicine used to treat dengue fever. Most TCM and dengue clinical studies were retrospective single-center studies with a small number of participants.

Chai-Shi-Jie-Du (CSJD) granules were first used in the 2014 dengue outbreak in Guangzhou, and they have been modified and improved during subsequent years. They have been proven to be effective in relieving fever and other symptoms of dengue virus infection [10] and to have very low acute toxicity in rats [11]. CSJD was explored especially for treating dengue fever, and it has been used in clinic settings for many years. To further investigate the effects of CSJD, we conducted a multicenter real-world study and explored the possible mechanism mainly based on network pharmacology. Real-world studies investigate health interventions whose aims are to reflect health intervention effectiveness in routine clinical practice and generate real-world data that can subsequently be analyzed to produce real-world evidence. Real-world evidence helps bridge the evidence gap from the RCTs to understand the true safety and efficacy of the health intervention in a real-world setting.

## 2. Methods

### 2.1. Participants

This retrospective multicenter real-world study was carried out in 5 tertiary hospitals located in 3 provinces in Southern China from 1 August to 31 December 2019. The study was approved by the Institutional Research Ethics Committee of Guangzhou Eighth People's Hospital, Guangzhou Medical University (20170885), and registered in the Chinese Clinical Trial Registry (ChiCTR2100049922). The recruited patients were diagnosed by using a DENV-specific reverse transcription-polymerase chain reaction (RT-PCR) assay or nonstructural protein 1- (NS1-) specific ELISA [12]. Patients younger than 14 years old, who met the WHO criteria for severe dengue (SD) [4] or those who used other TCM formulations, were excluded.

### 2.2. Design of the Real-World Study

**Figure 1:**
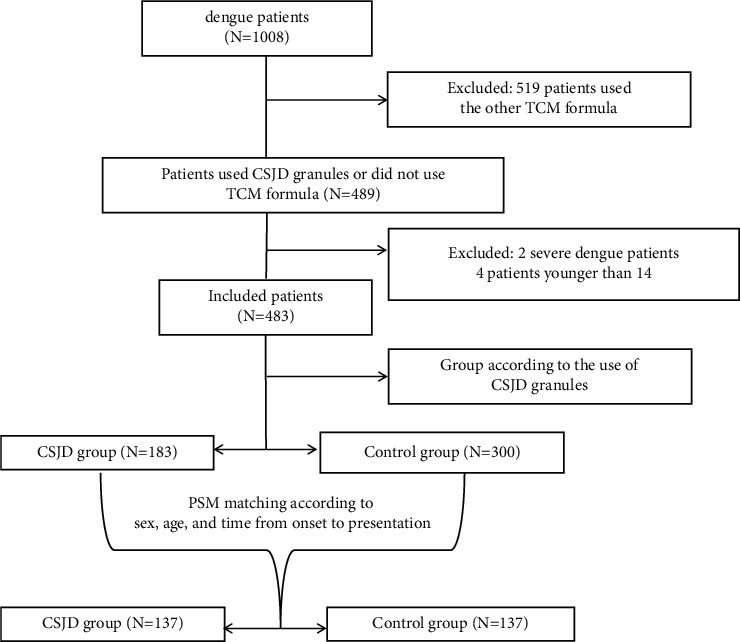
Flowchart of the participants. TCM, traditional Chinese medicine. CSJD, Chai-Shi-Jie-Du granules. PSM, propensity score matching.

First, the enrolled patients were divided into a CSJD group and a control group based on the presence or absence of CSJD. The CSJD group comprised patients treated with CSJD and conventional western therapies such as oral and intravenous rehydration therapy, antipyretics, analgesic, glycyrrhizin preparations, and proton pump inhibitor, while the control group included patients who were solely using conventional western medicine. Next, the two groups were matched according to age, sex, and time from onset to presentation through propensity score matching (PSM) using SPSS 23.0. Clinical data were collected before treatment and on the last day of the treatment. The disease course was defined as the number of days between the first and the last days of the treatment. The primary outcomes were the time to defervescence (from presentation to defervescence and lasting for at least 24 h), the disease course, and the rate of progression to SD. The secondary outcomes were the laboratory examination results, symptoms, and signs. The safety survey was used to record adverse effects according to guidelines from the Common Terminology Criteria for Adverse Events (CTCAE) version 5.0. All of the data were retrospectively collected by the researchers at each site through the Cloud Platform for Traditional Chinese Medicine Fever Specialist Alliance (203.86.79.218:8188). The study flowchart is shown in Figure 1.

### 2.3. Composition of the CSJD Granules

CSJD comprised 10 TCM drugs, including *Gypsum Fibrosum (Shi gao)*, *Scutellariae Radix (Huang qin)*, *Radix Bupleuri (Chai hu)*, *Pogostemon cablin (Blanco) Benth. (Guang huo xiang)*, *Radix Puerariae (Ge gen)*, *Talc (Hua shi)*, *Bubalus bubalis (Shui niu jiao)*, *Codonopsis Radix (Dang shen)*, *Coicis Semen (Yi yi ren)*, and *licorice (Gan cao)*. It was manufactured by E-FONG Pharmaceutical, Foshan, Guangdong, China, strictly following the technical requirements for quality control and the standard formulation of Chinese medicine granules developed by the National Administration of Traditional Chinese Medicine (NATCM). CSJD is a brown powder (9.1 g per packet). Patients took one packet twice a day for five days.

### 2.4. *In Vitro* Cytotoxicity Assay

CSJD was dissolved in dimethyl sulfoxide (DMSO) to achieve a concentration of 500 mg/mL, then, the fully dissolved mixture was centrifuged, and the supernatant was stored at 4°C prior to use. Syrian baby hamster kidney (BHK) cells were adopted to evaluate the cytotoxic effects. The cytotoxic effects of CSJD were evaluated using luminescent CellTiter-Glo® (Promega Corp., Madison, WI, USA) according to the manufacturer's instructions. Briefly, monolayers of BHK cells in 96-well plates were rinsed with PBS followed by incubation with the indicated concentrations of CSJD. After 96 h of incubation, 100 *µ*L of CellTiter-Glo solution was directly mixed with the culture medium, followed by incubation at room temperature for 15 min. Then, the luminescence intensity was recorded, and the 50% cytotoxic concentration (CC_50_) was calculated by using GraphPad Prism 7.0 software.

### 2.5. *In Vitro* Antiviral Assay

The DENV2-NGC virus was propagated in C6/36 cells (<4 generations), and the virus titers were determined based on a 50% tissue culture infectious dose (TCID_50_) assay. BHK-cell monolayers were grown in 96-well plates and inoculated with 100 TCID_50_ of DENV-2 NGC at 37°C for 1 h. The inoculum was removed, and the cells were rinsed with PBS twice and subsequently incubated with the indicated concentrations of CSJD. Following 96 h of incubation, the cell viability was evaluated using luminescent CellTiter-Glo®, and IC_50_ was calculated as described in the cytotoxic assay.

### 2.6. Screening of Bioactive Components, Proteins, and Targets Related to CSJD

The compounds and targets of the constituents in CSJD were retrieved from the online public databases TCMSP [13] (https://www.tcmsp-e.com) and BATMAN-TCM [14] (https://bionet.ncpsb.org.cn/batman-tcm/). *Gypsum Fibrosum* was not included in any of the databases. The ingredients of *Gypsum Fibrosum* were obtained from articles [15, 16], and the targets were screened using STITCH [17] (https://stitch.embl.de/) with “*Homo sapiens*” as the species. The molecules with oral bioavailability (OB ≥ 30%) and druglikeness (DL ≥ 0.18) were identified as candidate compounds for analysis except for molecules of *Bubalus bubalis, Talc,* and *Gypsum Fibrosum*, because their OB and DL data were not available in the database. All the targets obtained above were standardized according to their gene names by searching the UniProtKB (https://www.UniProt.org/) database with “*Homo sapiens*” as the species.

### 2.7. Identification of Genes Associated with Dengue Fever

Using “dengue fever” as keywords and selecting the species as “*Homo sapiens*,” the corresponding gene sets were retrieved from the GeneCards [18] and DisGeNET [19] databases. Then, the union of the two gene sets was collected to obtain the disease gene set for dengue fever.

### 2.8. Selection of Potential Therapeutic Target Proteins at the Intersection of the Drug Targets and Disease Targets

The CSJD-related proteins and the disease genes were mapped employing the Venny2.1 online tool (https://bioinfogp.cnb.csic.es/tools/venny/), and the intersection of the drug targets and disease targets were regarded as the potential therapeutic target proteins.

### 2.9. Network Topology Analysis of Potential Therapeutic Targets

The Search Tool for the Retrieval of Interacting Genes/Proteins (STRING) [20] (https://string-db.org/, version: 11.5) was used to generate a protein-protein interaction (PPI) network of the potential target proteins. The species was set to “*Homo sapiens*”; the lowest mutual action threshold was set to “highest confidence” (>0.9), and other parameters were set to their default settings. Next, the network topology parameters were analyzed by Cytoscape 3.9.0, and the hub target proteins were screened depending on the value of degree.

### 2.10. Establishment of the Network and Analysis

The constituents and the obtained active ingredients of CSJD and the corresponding targets were imported into Cytoscape 3.9.0 to establish the drug-active ingredient-potential target network. R and R studio with packages org. Hs.eg.db [21] and clusterProfiler [22] were used to perform the gene ontology (GO) enrichment analysis [23] and the Kyoto Gene and Genome Encyclopedia (KEGG) enrichment analysis [24].

### 2.11. Statistical Analysis

Data management and statistical analysis were performed by using SPSS 23.0. Categorical data were expressed as the frequency and percentage and analyzed using the chi-squared (*χ*^2^) or Fisher's tests. Continuous data are presented as the means ± standard deviations and were analyzed with independent *t*-tests or Mann–Whitney *U* tests. When the *p* value was less than 0.05, the result was considered to be statistically significant.

## 3. Results

### 3.1. Demographic and Clinical Data at Baseline

A total of 525 of the 1,008 patients were excluded. Of the remaining 483 nonsevere dengue patients, 137 pairs were successfully matched according to age, sex, and the time from onset to presentation. Patient demographic characteristics, such as age, sex, time from onset to presentation, and presence of comorbidities, did not differ significantly between the two groups. DENV-1 was the predominant serotype, and secondary infection was rare in both groups. However, the temperature at presentation was slightly higher in the CSJD group than that in the control group (37.9°C vs. 37.7°C, *P* < 0.05) (Table 1).

### 3.2. Main Outcomes

**Figure 2:**
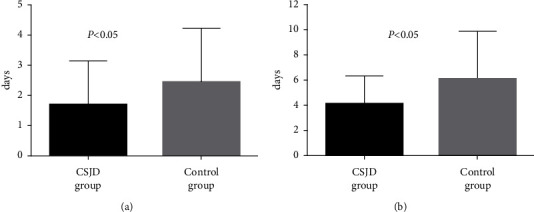
Comparison of the time to defervescence and the disease course between the CSJD and the control groups.

The time to defervescence in the CSJD group was significantly shorter than that in the control group (1.7 days vs. 2.5 days, *P* < 0.05). The disease course in the CSJD group was significantly shorter than that in the control group (4.1 days vs. 6.1 days, *P* < 0.05). Each group had a patient who progressed to SD due to acute kidney injury, and the rates of progression to SD were the same in the two groups (Figure 2).

### 3.3. Symptoms, Laboratory Examinations, and Adverse Events

There were no differences in the duration of symptoms, including chills, dizziness, headache, myalgia, fatigue, loss of appetite, nausea, vomiting, abdominal pain, diarrhea, minor bleeding, or rash (Table S1). Patients in the TCM group had slightly higher levels of HCT and PLT before treatment, and the differences in these values were 1.1% and 16 × 10^9^/L, respectively. After treatment, most of the between-group differences in blood and biochemical values did not reach statistical significance. However, the AST level in the CSJD group was significantly higher than that in the control group (78.3 U/L vs. 59.4 U/L, *P* < 0.05) (Table 2). The most frequent adverse events were level 2 digestive system discomfort according to the CTCAE 5.0 guidelines, including 5 cases of diarrhea, 3 cases of vomiting, 2 cases of abdominal pain, 1 case of epigastric discomfort, and 1 case of hiccups. The rate of adverse events in the two groups did not differ significantly (6.6% vs. 2.2%, *P*=0.077) (Table 3).

### 3.4. Cytotoxicity Assay and Antiviral Activity of CSJD *In Vitro*

**Figure 3:**
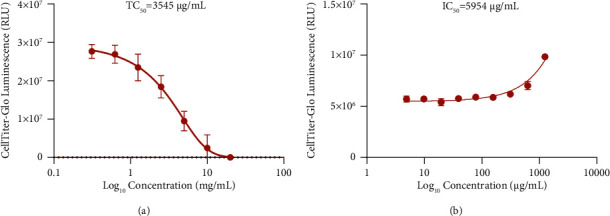
Cytotoxic effect and antiviral activity of CSJD granules on BHK cells. (a) Cytotoxic effect of CSJD; (b) antiviral activity of CSJD granules against DENV-2. Data are presented as the mean ± SD.

CSJD showed no apparent cytotoxicity at concentrations up to 1250 *μ*g/mL, and TC_50_ was 3545 *μ*g/mL (Figure 3(a)). As shown in Figure 3(b), CSJD inhibited the replication of DENV-2 with an IC_50_ value of 5954 *μ*g/mL, which was much higher than its TC_50_, and the therapeutic index was 0.6.

### 3.5. Identification of Potential Therapeutic Target Proteins

**Figure 4:**
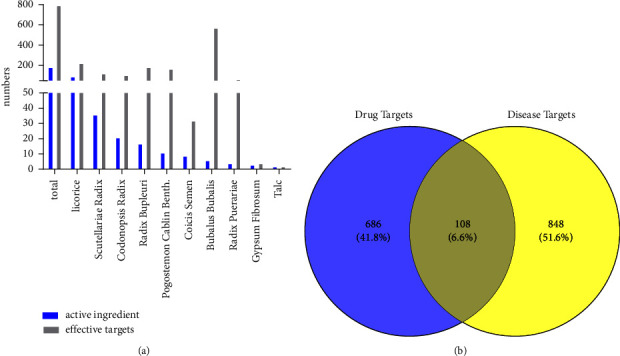
(a) Active compounds and targets of constituents in CSJD granules. (b) A total of 794 and 956 targets of CSJD and dengue fever, respectively, with 108 shared targets.

Based on the TCMSP, BATMAN-TCM, and STITCH databases, 187 active compounds were selected by the filtering criteria “OB ≥ 30%, DL ≥ 0.18,” and a total of 794 potential target proteins were selected (Figure 4(a), Table S2). A total of 773 and 360 disease genes related to “dengue fever” were obtained from the GeneCards and DisGeNET databases, respectively, and 956 disease-related genes were selected after combination. A total of 108 potential therapeutic target proteins were identified by mapping the target protein set of CSJD and the disease gene set of dengue fever (Figure 4(b)).

### 3.6. Network Topology Analysis and Drug-Active Compound-Potential Target Network

There were 99 nodes and 381 edges in the PPI network, and 9 disconnected proteins were excluded from the network (Figure S1). After further analysis of the network topology parameters, 29 hub target proteins were identified, which were divided into 2 clusters depending on their importance. As shown in Figure S2, Cytoscape was used to generate the drug-active compound-potential therapeutic target network. In this network, the different nodes represented the drug components, potential active compounds, and effector targets of CSJD, and the network edges showed relationships among these three factors. This result fully illustrated the characteristics of the multiple components and targets of CSJD.

### 3.7. GO Enrichment Analysis

**Figure 5:**
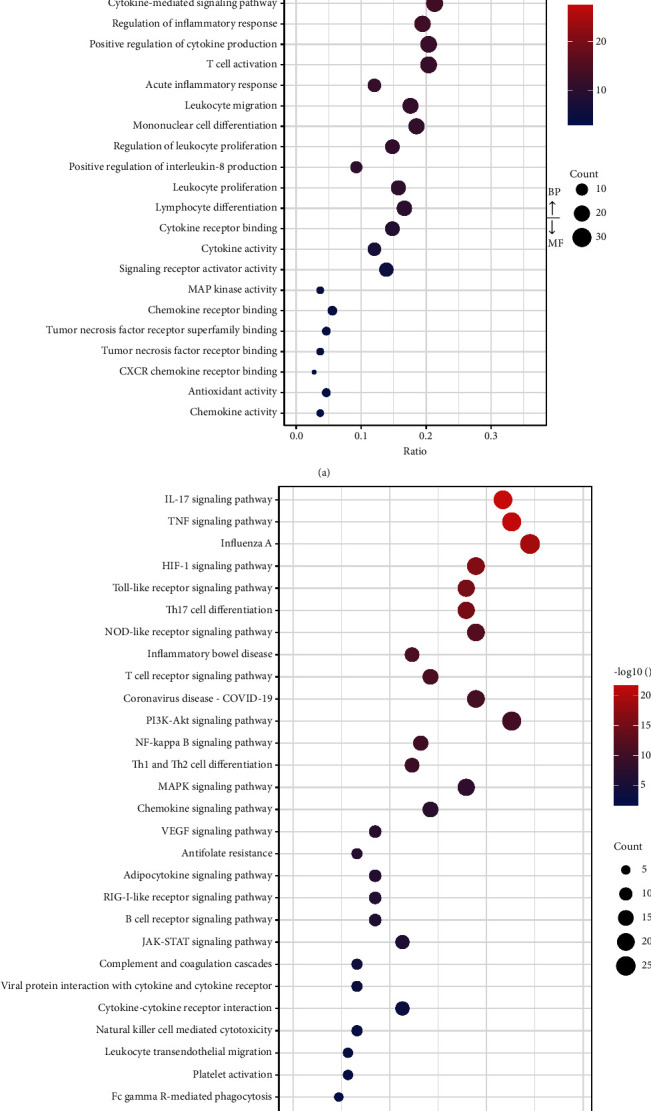
(a) The top terms of GO enrichment of BP and MF (*P* < 0.05). (b) The top 30 pathways identified in the KEGG enrichment analysis (*P* < 0.05).

A total of 2,426 significantly enriched GO terms were obtained using a *p* value of <0.05 as the threshold, yielding 108 potential therapeutic target proteins.  Figure 5(a) shows the 20 most significant BP terms and the top 10 MF terms. The terms were significantly enriched for three aspects: the regulation of the immune response, the oxidative stress system, and lipopolysaccharide.

### 3.8. KEGG Pathway Enrichment Analysis

A total of 165 pathways were obtained using a threshold of *p*  < 0.05, and the top 30 pathways are shown in Figure 5(b). The top 2 pathways were the IL-17 signaling pathway and the TNF signaling pathway. We also found that several inflammation-related terms were significantly enriched, such as the toll-like receptor signaling pathway, Th17-cell differentiation, T-cell receptor signaling pathway, and NF-kappa B signaling pathway.

## 4. Discussion

Our main finding was that the treatment of dengue with CSJD might shorten both the time to defervescence and the disease course. The time to defervescence in the CSJD group was 0.8 days shorter than that in the control group. Similar to our results, the results of some previous studies [25–27] have suggested that traditional Chinese medicine could help relieve fever. Early in 1988, Liu Shichang and He Yangzhong [28] reported that treating dengue with a TCM herb formulation could help shorten the fever duration. The fever durations of the TCM group and the control group were 1.8 ± 1.1 days and 3.3 ± 2.0 days, respectively. The TCM formula used comprised mainly *Gypsum, Artemisia,* and *Radix Bupleuri*, and the amount of *Gypsum* ranged from 40 to 100 g. In recent years, a randomized controlled trial of TCM formulas including 160 dengue patients reported similar results, with fever durations of the TCM group and the control group being 1.90 ± 0.80 days and 3.50 ± 1.04 days, respectively [29]. The different formulas used in the above studies shared similar components, such as *Gypsum, Radix Bupleuri, Scutellariae Radix, Radix Puerariae, Artemisia,* and *Bubalus bubalis*, which were also widely used in the treatment of dengue.

The disease course in the CSJD group was 2 days shorter than that in the control group, which was of great significance for patients, not only by saving time but also reducing healthcare costs. Part of the reason for the shortened disease course in the CSJD group was that patients usually felt better after defervescence and were discharged from the hospital. The most frequent adverse events were level 2 digestive system discomfort, which was higher in the CSJD group than that in the control group with no significant difference, which could be related to the taste of CSJD. Usually, TCM decoctions are very bitter and smelly, which may cause nausea and vomiting. Chinese patent medicines that already on the market are usually made to taste sweet, but our preparation has not yet improved the taste. The AST level after treatment in the CSJD group was significantly higher than that in the control group (78.3 U/L vs. 59.4 U/L, *P* < 0.05). This finding could be related to the shorter disease course of the CSJD group. The present study showed that CSJD could help relieve fever and assist in disease recovery. However, the *in vitro* experiment showed that CSJD could not inhibit dengue virus replication in its safe dose, with an IC_50_ of 5954 *μ*g/mL and a therapeutic index of 0.6. Thus, we explored the underlying mechanisms of CSJD in treating dengue fever through network pharmacology.

CSJD had a strong influence on the immune response according to network pharmacological analysis. Many cytokines and immune regulation-related proteins, such as IL-1, IL-2, IL-4, IL-6, CXCL-8, CXCXL-11, MMP-9, INF-*γ*, TNF, TLR4, and MAPKs, were among the key proteins revealed by network pharmacology. The GO and KEGG pathway enrichment analyses showed that CSJD had effects on both the innate immune system and the adaptive immune system, including on cytokines, leukocytes, mononuclear cells, lymphocytes, T cells, and B cells. Specifically, response to tumor necrosis factor, cytokine-mediated signaling pathway, cytokine activity, and MAP kinase activity were among the top terms in the GO enrichment analysis (Figure 5(a)). KEGG enrichment analysis showed that CSJD affected many immune-regulating signaling pathways, such as those involving NF-kappa B, TNF, toll-like receptor, MAPK, T-cell receptor, and B-cell receptor (Figure 5(b)). Monocytes are the first to respond to dengue virus infection by using pattern recognition receptors (PRRs), such as toll-like receptors [30]. Highly purified recombinant NS1 was shown to activate human peripheral blood mononuclear cells via toll-like receptor 4 (TLR4), leading to the induction and release of proinflammatory cytokines and chemokines [31]. B cells mediate the neutralization of infection but can lead to a phenomenon termed “antibody-dependent enhancement of infection.” T cells target cell lysis, but cross-reactive T cells play a role in mediating the pathogenesis of dengue hemorrhagic fever (DHF) and enhancing the circulation of cytokines and chemokines. The release of cytokines, including TNF, IL-1*β*, IL-2, IL-4, IL-6, CXCL8 (IL-8), MMP, and IFN-*γ*, is considered a key factor contributing to DHF pathogenesis [32]. Generally, CSJD affects many steps of the immune response, from the entry of the virus to the end of the infection. The change in the immune response readily alters the course of the disease.

Changes in the immune response, cytokines, and chemokines influence body temperature. *Gypsum, Scutellariae Radix, Radix Bupleuri, Radix Puerariae,* and *Bubalus bubalis* might exert antipyretic effects together, among which *Gypsum* might be the most important one. One of the main indications for *Gypsum* is in treating febrile diseases caused by exogenous pathogens [33]. It is a component of the famous baihu decoction, which can be used as an antifebrile and anti-inflammatory drug for an extended period [34, 35]. *Gypsum* was reported to reduce the elevation of body temperature in rats and cats, and this effect was mediated by its influence on thermosensitive neurons in the PO/AH region [36, 37]; suppression of the NF-*κ*B signaling pathway; and reduction in the levels of TNF-*α*, interleukin-1 (IL-1), and interleukin-6 (IL-6) [38]. *Scutellariae Radix, Radix Bupleuri, Radix Puerariae,* and *Bubalus bubalis* are also antipyretic herbal medicines used in TCM, and their antipyretic effects have been confirmed in animal experiments [39–44]. The antipyretic effects of *Scutellariae Radix* were found to be associated with reducing the levels of TNF-*α*, IL-1*β*, IL-6, and other pyrogenic cytokines in the serum, hypothalamus, and cerebrospinal fluid [45], which may be mediated by the inhibition of the TLR4 signaling pathway and the downregulation of NF-*κ*B activation [46]. The *Radix Bupleuri* antipyretic mechanism is related to the regulation of the synthesis and secretion of cyclic adenosine monophosphate (cAMP) and arginine vasopressin (AVP) [43, 44]. The antipyretic mechanism of *Radix Puerariae* is due to the regulation of the synthesis and release of endogenous pyrogen via the inhibition of NF-*κ*B activation and the MAPK pathway [41]. *Radix Bupleuri* and *Radix Puerariae* could play an immunomodulatory role and reduce the levels of cytokines, such as IL-1, IL-6, IL-17, TNF-*α*, and IFN-*γ* [43, 44]. *Bubalus bubalis* has been shown to exert antipyretic activity by enhancing antioxidation enzyme activities and decreasing prostaglandin E2 (PGE2) production [40]. COX2, one of the potential therapeutic target proteins, is an inducible enzyme that regulates the biosynthesis of PGE2. Pogostone, an ingredient of *Pogostemon cablin (Blanco) Benth.*, can suppress the T-cell response and attenuate TNF-*α*-induced injury in A549 cells by inhibiting NF-kappa B and activating Nrf2 pathways [47, 48]. *Codonopsis Radix* [49] and *licorice* [50, 51] also have immune-regulating effects according to the results of previous publications.

Several oxidative stress-related pathways were identified in the GO and KEGG enrichment analyses. An imbalance of reactive oxygen species (ROS) triggers the sudden release of cytokines, which can aggravate existing cases of disease and lead to the apoptosis of microvascular endothelial cells [52, 53]. As described in the results of network pharmacology research, the response to metabolic processes involving oxidative stress, reactive oxygen species, and reactive nitrogen species are relevant to the oxidative stress response, indicating that CSJD might influence these processes.

The top 2 terms in GO enrichment were related to lipopolysaccharide (LPS). It is a potent stimulus for the production of platelet-activating factors (PAF) and other inflammatory cytokines from monocytes [54]. Serum LPS elevation was shown to be associated with the development of SD [55]. Since LPS is an endotoxin derived from the outer membrane of Gram-negative bacteria, these findings suggest that microbial translocation could occur in acute dengue and contribute to dengue disease severity [56]. Responses to lipopolysaccharide and molecules of bacterial origin were the first two terms in GO enrichment analysis, indicating that CSJD could contribute to controlling microbial translocation.

There were some limitations in this study. First, the study was a retrospective study rather than a prospective and randomized blind trial. Second, the conventional details of western treatment were not recorded or analyzed. Third, cytokines were not detected in the clinical or *in vitro* analyses. Nevertheless, we demonstrated the true effectiveness of CSJD in treating dengue fever and the potential underlying mechanisms, providing a basis for future research.

## 5. Conclusion

This multicenter real-world study showed that CSJD granules might shorten both the time to defervescence and the disease course of dengue fever. Based on the results of *in vitro* and network pharmacological analyses, the potential mechanisms were likely related to regulating immunity, moderating the oxidative stress response, and the response to lipopolysaccharide.

## Figures and Tables

**Table 1:** Demographic and clinical characteristics of the patients at baseline.

Variables	Before PSM			After PSM		
	CSJD (*n* = 183)	Control (*n* = 300)	*P* value	CSJD (*n* = 137)	Control (*n* = 137)	*P* value
Age (years)	36.3 ± 13.4	37.3 ± 13.2	0.454	37.6 ± 13.4	38.4 ± 13.9	0.640
Sex						
Male	113 (61.7%)	174 (58.0%)	0.416	82 (59.9%)	86 (62.8%)	0.620
Female	70 (38.3%)	126 (42.0%)		55 (40.1%)	51 (37.2%)	
Time from onset to presentation (days)	2.8 ± 1.9	4.3 ± 2.1	0.000	3.5 ± 1.7	3.5 ± 1.7	0.974
Comorbidity						
Hypertension	3 (1.6%)	9 (3.0%)	0.528	3 (2.2%)	6 (4.4%)	0.498
Diabetes	0 (0.0%)	1 (0.3%)	1.000	0 (0.0%)	1 (0.7%)	1.000
HBV	6 (3.3%)	4 (1.3%)	0.145	5 (3.6%)	2 (1.5%)	0.444
Others	8 (4.4%)	7 (2.3%)	0.210	5 (3.6%)	3 (2.2%)	0.720
Temperature at presentation (°C)	38.0 ± 0.9	37.5 ± 1.0	0.000	37.9 ± 0.9	37.7 ± 1.0	0.015
Serotypes			0.000			0.079
DENV-1	42 (23.0%)	129 (43.0%)		40 (29.2%)	31 (22.6%)	
DENV-2	1 (0.5)	24 (8.0%)		1 (0.7%)	6 (4.4%)	
DENV-3	4 (2.2%)	3 (1.0%)		4 (2.9%)	1 (0.7%)	
Unclassified	136 (74.3)	144 (48.0%)		92 (67.2%)	99 (72.3%)	
Secondary infection	1 (0.5%)	3 (1.0%)	0.987	1 (0.7%)	1 (0.7%)	1.000
WBC (10^9^/L)	4.2 ± 2.4	3.5 ± 2.1	0.001	3.8 ± 2.3	3.3 ± 1.7	0.388
HGB (g/L)	140.9 ± 17.8	138.6 ± 16.9	0.070	140.1 ± 17.8	137.7 ± 16.7	0.281
HCT (%)	41.6 ± 4.8	40.4 ± 4.4	0.006	41.4 ± 4.7	40.3 ± 4.4	0.041
PLT (10^9^/L)	140.3 ± 59.3	105.9 ± 55.8	0.000	127.0 ± 56.3	111.0 ± 55.0	0.020
ALT (U/L)	50.7 ± 44.7	60.0 ± 60.8	0.283	54.5 ± 48.9	64.4 ± 69.9	0.719
AST (U/L)	71.6 ± 72.1	75.8 ± 80.3	0.603	77.4 ± 79.9	77.0 ± 98.4	0.117
ALB (g/L)	41.4 ± 4.5	40.1 ± 3.7	0.027	40.5 ± 4.2	40.7 ± 3.5	0.640
LDH (U/L)	329.5 ± 156.2	352.4 ± 184.2	0.310	335.9 ± 157.9	342.0 ± 200.7	0.663
Cr (*μ*mol/l)	73.6 ± 16.6	69.9 ± 18.5	0.035	72.4 ± 17.2	74.3 ± 19.0	0.439

CSJD, Chai-Shi-Jie-Du granules. PSM, propensity score matching. HBV, hepatitis B virus infection. WBC, white blood cell. HGB, hemoglobin. HCT, hematocrit. PLT, platelet. ALT, alanine aminotransferase. AST, aspartate aminotransferase. ALB, albumin. LDH, lactate dehydrogenase. Cr, creatinine.

**Table 2:** Comparison of the laboratory data between the CSJD and control groups after treatment.

Variables	Before PSM			After PSM		
	CSJD (*n* = 183)	Control (*n* = 300)	*P* value	CSJD (*n* = 137)	Control (*n* = 137)	*P* value
WBC (10^9^/L)	4.7 ± 2.3	5.3 ± 2.0	0.003	5.1 ± 2.3	5.0 ± 1.9	0.818
HGB (g/L)	139.6 ± 20.1	134.6 ± 17.1	0.008	137.4 ± 20.8	134.4 ± 18.2	0.243
HCT (%)	40.7 ± 5.3	39.4 ± 4.5	0.007	40.1 ± 5.4	39.5 ± 4.8	0.309
PLT (10^9^/L)	143.9 ± 69.5	166.7 ± 83.7	0.009	144.2 ± 66.6	159.0 ± 87.0	0.430
ALT (U/L)	71.5 ± 54.3	69.0 ± 48.5	0.989	72.8 ± 57.0	69.2 ± 50.5	0.915
AST (U/L)	76.4 ± 55.2	57.7 ± 38.1	0.000	78.3 ± 57.9	59.4 ± 41.7	0.004
ALB (g/L)	38.6 ± 4.3	39.3 ± 3.4	0.122	38.4 ± 4.4	39.2 ± 3.5	0.259
LDH (U/L)	360.7 ± 122.7	362.0 ± 113.3	0.982	361.6 ± 122.4	354.3 ± 117.6	0.642
Cr (*μ*mol/l)	68.5 ± 26.7	66.4 ± 24.6	0.566	68.7 ± 28.4	70.2 ± 27.6	0.381

CSJD, Chai-Shi-Jie-Du granules. PSM, propensity score matching. WBC, white blood cell. HGB, hemoglobin. HCT, hematocrit. PLT, platelet. ALT, alanine aminotransferase. AST, aspartate aminotransferase. ALB, albumin. LDH, lactate dehydrogenase. Cr, creatinine.

**Table 3:** Comparison of the adverse events between the CSJD and control groups.

Grade	Variables	Total	CSJD (*n* = 137)	Control (*n* = 137)	*P* value
1-2	Digestive system	12 (4.4%)	9 (6.6%)	3 (2.2%)	0.077
3 or above	WBC	4 (1.5%)	4 (2.9%)	0 (0.0%)	0.131
3 or above	HGB	1 (0.4%)	0 (0.0%)	1 (0.7%)	1.000
3 or above	PLT	5 (1.8%)	1 (0.7%)	4 (2.9%)	0.362
3 or above	ALT	4 (1.5%)	3 (2.2%)	1 (0.7%)	0.614
3 or above	AST	2 (0.7%)	2 (1.5%)	0 (0.0%)	0.478
3 or above	Cr	1 (0.4%)	0 (0.0%)	1 (0.7%)	1.000

CSJD, Chai-Shi-Jie-Du granules. WBC, white blood cell. HGB, hemoglobin. PLT, platelet. ALT, alanine aminotransferase. AST, aspartate aminotransferase. Cr, creatinine.

## Data Availability

The data that support the findings of this study are available from the corresponding author on request.

## Supplementary Materials

Supplementary Materials Table S1: duration of symptoms and signs between the CSJD and control groups. Table S2: drug-active ingredient-target database. Table S3: the GO enrichment results for the 108 cotargets. Table S4: the KEGG enrichment results for the 108 cotargets. Figure S1: the PPI network of 108 potential therapeutic target proteins. Figure S2: the “drug-active compound-therapeutic target protein” network of the effect of CSJD on dengue fever. Red represents the most important potential therapeutic targets, and orange represents the less important potential therapeutic targets; other potential therapeutic targets are colored yellow. Green represents the active compound. Purple represents the constituent drugs of CSJD. The edges represent the relationship between constituent drugs, active compounds, and potential therapeutic target proteins.
