# Supplementary material for: Real-World Study on Chai-Shi-Jie-Du Granules for the Treatment of Dengue Fever and the Possible Mechanisms Based on Network Pharmacology
Source: Evid Based Complement Alternat Med. 2023 Aug 30;2023:9942842. doi: 10.1155/2023/9942842 (PMC10482559; doi:10.1155/2023/9942842)
Supplement: Supplementary Materials — Table S1: duration of symptoms and signs between the CSJD and control groups. Table S2: drug-active ingredient-target database. Table S3: the GO enrichment results for the 108 cotargets. Table S4: the KEGG enrichment results for the 108 cotargets. Figure S1: the PPI network of 108 potential therapeutic target proteins. Figure S2: the “drug-active compound-therapeutic target protein” network of the effect of CSJD on dengue fever. Red represents the most important potential therapeutic targets, and orange represents the less important potential therapeutic targets; other potential therapeutic targets are colored yellow. Green represents the active compound. Purple represents the constituent drugs of CSJD. The edges represent the relationship between constituent drugs, active compounds, and potential therapeutic target proteins. [file 9942842.f1.zip › supplementary materials(Table S1+Figure S1+Figure S2) (1).docx]

Table S1. Duration of symptoms and signs between the CSJD and control groups

| Variables  (days) | Before PSM | | | After PSM | | |
| --- | --- | --- | --- | --- | --- | --- |
|  | CSJD  (n=183) | Control (n=300) | *P* value | CSJD  (n=137) | Control  (n=137) | *P* value |
| Chills | 1 (1,2) | 1 (1,2) | 0.956 | 1 (1,2) | 2 (1,2) | 0.052 |
| Dizziness | 2.5 (1,3) | 1 (1,2.75) | 0.141 | 2.5 (1,3) | 2 (1,2.5) | 0.256 |
| Headache | 2 (1,3) | 2 (1,3) | 0.527 | 1.5 (1,3) | 2 (1,3) | 0.838 |
| Body ache | 2 (1,3) | 2 (1,3) | 0.223 | 2 (1,3) | 2 (1,3) | 0.407 |
| Fatigue | 3 (1,5) | 2 (1,3) | 0.302 | 3 (1,3) | 2 (1,4) | 0.928 |
| Anorexia | 2 (1,3) | 2 (1,3) | 0.589 | 2 (1,3) | 2 (1,3) | 0.586 |
| Nausea | 1 (1,2) | 1 (1,2) | 0.811 | 1.5 (1,2) | 1.5 (1,2) | 0.626 |
| Vomiting | 1 (1,1) | 1 (1,1) | 0.956 | 1 (1,1) | 1 (1,1.5) | 0.658 |
| Abdominal pain | 1 (0.75,1) | 1 (1,2) | 0.285 | 1 (1,1) | 1.5 (1,2.25) | 0.226 |
| Diarrhea | 1 (1,2) | 1 (1,2) | 0.708 | 1 (1,1.25) | 1 (1,2.25) | 0.233 |
| Minor bleeding | 1 (1,1.5) | 2 (1,2) | 0.163 | 1 (1,1.5) | 1 (1,2) | 0.35 |
| Rash | 3 (1,5) | 3 (2.25,4.75) | 0.396 | 3 (1,6) | 4 (3,4.25) | 0.375 |
| Petechia | 5 (3,5) | 3 (2,5) | 0.123 | 5 (3,5.25) | 3 (1,5) | 0.097 |

Table S2. Drugs-active ingredients-targets database

Table S3. The GO enrichment results for the 108 cotargets

Table S4. The KEGG enrichment results for the 108 cotargets


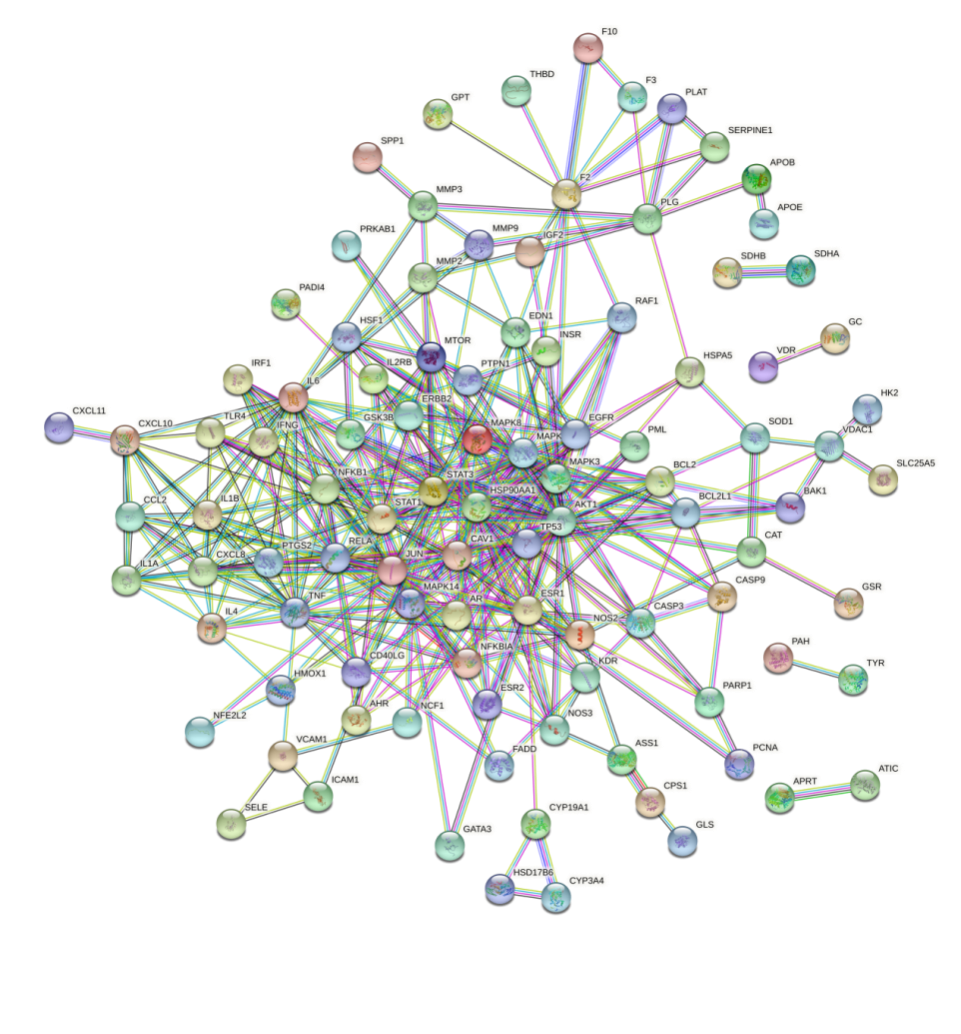


Figure S1. The PPI network of 108 potential therapeutic target proteins


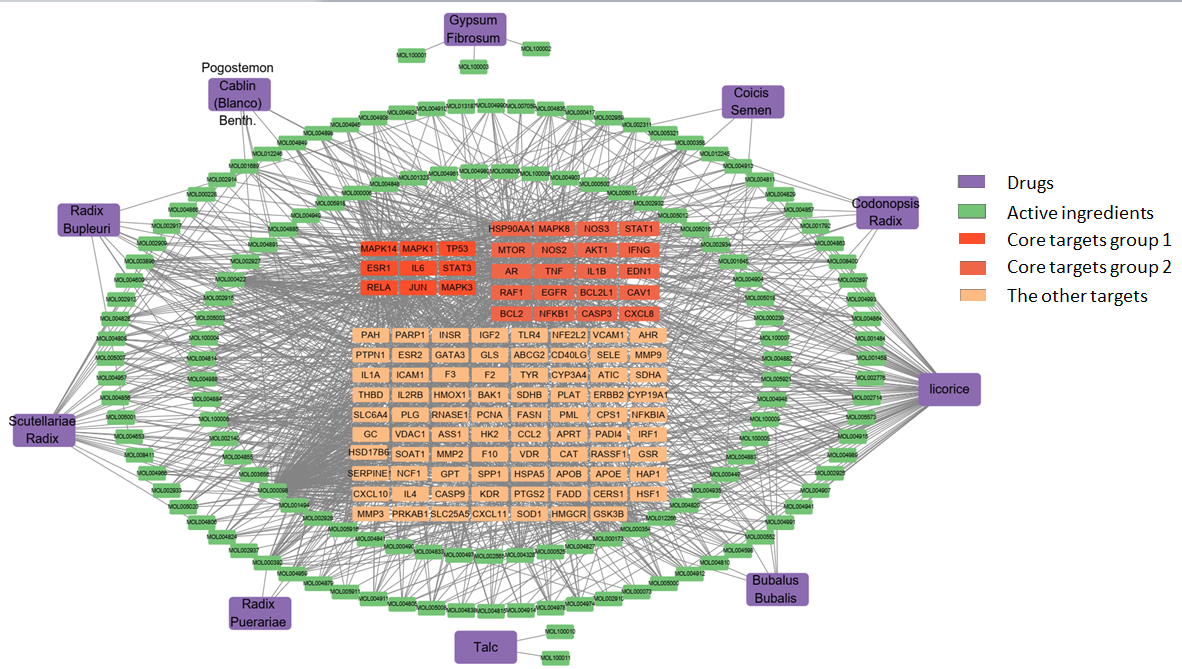


Figure S2. The “drugs-active compounds-therapeutic target proteins” network of the effect of CSJD on dengue fever. Red represents the most important potential therapeutic targets, and orange represents the less important potential therapeutic targets; other potential therapeutic targets are colored yellow. Green represents the active compound. Purple represents the constituent drugs of CSJD. The edges represent the relationship between constituent drugs, active compounds and potential therapeutic target proteins
