# Supplementary material for: Real-World Study on Chai-Shi-Jie-Du Granules for the Treatment of Dengue Fever and the Possible Mechanisms Based on Network Pharmacology
Source: Evid Based Complement Alternat Med. 2023 Aug 30;2023:9942842. doi: 10.1155/2023/9942842 (PMC10482559; doi:10.1155/2023/9942842)
Supplement: Supplementary Materials — Table S1: duration of symptoms and signs between the CSJD and control groups. Table S2: drug-active ingredient-target database. Table S3: the GO enrichment results for the 108 cotargets. Table S4: the KEGG enrichment results for the 108 cotargets. Figure S1: the PPI network of 108 potential therapeutic target proteins. Figure S2: the “drug-active compound-therapeutic target protein” network of the effect of CSJD on dengue fever. Red represents the most important potential therapeutic targets, and orange represents the less important potential therapeutic targets; other potential therapeutic targets are colored yellow. Green represents the active compound. Purple represents the constituent drugs of CSJD. The edges represent the relationship between constituent drugs, active compounds, and potential therapeutic target proteins. [file 9942842.f1.zip › Table S2. Drugs-active ingredients-targets database (1).pdf]

Drugs	MolId	MolName	Symbol	Potential
licorice	MOL004924	(-)-Medicocarpin	PTGS2	YES
licorice	MOL004924	(-)-Medicocarpin	ACHE	NO
Radix Bupleuri	MOL004653	(+)-Anomalin	F2	YES
Radix Bupleuri	MOL004653	(+)-Anomalin	KCNH2	NO
Radix Bupleuri	MOL004653	(+)-Anomalin	F10	YES
Radix Bupleuri	MOL004653	(+)-Anomalin	PTGS2	YES
Radix Bupleuri	MOL004653	(+)-Anomalin	TOP2A	NO
Radix Bupleuri	MOL004653	(+)-Anomalin	DPP4	NO
licorice	MOL004941	(2R)-7-hydroxy-2-(4-hydroxyp	PTGS1	NO
licorice	MOL004941	(2R)-7-hydroxy-2-(4-hydroxyp	ESR1	YES
licorice	MOL004941	(2R)-7-hydroxy-2-(4-hydroxyp	PTGS2	YES
licorice	MOL004941	(2R)-7-hydroxy-2-(4-hydroxyp	RXRA	NO
licorice	MOL004941	(2R)-7-hydroxy-2-(4-hydroxyp	PDE3A	NO
licorice	MOL004941	(2R)-7-hydroxy-2-(4-hydroxyp	ADRB2	NO
licorice	MOL004941	(2R)-7-hydroxy-2-(4-hydroxyp	HSP90AA1	YES
licorice	MOL004941	(2R)-7-hydroxy-2-(4-hydroxyp	DPEP1	NO
licorice	MOL004941	(2R)-7-hydroxy-2-(4-hydroxyp	MAOB	NO
licorice	MOL004941	(2R)-7-hydroxy-2-(4-hydroxyp	PRKACA	NO
licorice	MOL004941	(2R)-7-hydroxy-2-(4-hydroxyp	PKIA	NO
licorice	MOL004941	(2R)-7-hydroxy-2-(4-hydroxyp	CALM3	NO
licorice	MOL004941	(2R)-7-hydroxy-2-(4-hydroxyp	SLC6A4	YES
Scutellariae Radix	MOL000228	(2R)-7-hydroxy-5-methoxy-2-p	PTGS1	NO
Scutellariae Radix	MOL000228	(2R)-7-hydroxy-5-methoxy-2-p	DRD1	NO
Scutellariae Radix	MOL000228	(2R)-7-hydroxy-5-methoxy-2-p	CHRM3	NO
Scutellariae Radix	MOL000228	(2R)-7-hydroxy-5-methoxy-2-p	CHRM1	NO
Scutellariae Radix	MOL000228	(2R)-7-hydroxy-5-methoxy-2-p	ESR1	YES
Scutellariae Radix	MOL000228	(2R)-7-hydroxy-5-methoxy-2-p	SCN5A	NO
Scutellariae Radix	MOL000228	(2R)-7-hydroxy-5-methoxy-2-p	PTGS2	YES
Scutellariae Radix	MOL000228	(2R)-7-hydroxy-5-methoxy-2-p	RXRA	NO
Scutellariae Radix	MOL000228	(2R)-7-hydroxy-5-methoxy-2-p	PDE3A	NO
Scutellariae Radix	MOL000228	(2R)-7-hydroxy-5-methoxy-2-p	ADRA1A	NO
Scutellariae Radix	MOL000228	(2R)-7-hydroxy-5-methoxy-2-p	ADRA1B	NO
Scutellariae Radix	MOL000228	(2R)-7-hydroxy-5-methoxy-2-p	SLC6A3	NO
Scutellariae Radix	MOL000228	(2R)-7-hydroxy-5-methoxy-2-p	ADRB2	NO
Scutellariae Radix	MOL000228	(2R)-7-hydroxy-5-methoxy-2-p	SLC6A4	YES
Scutellariae Radix	MOL000228	(2R)-7-hydroxy-5-methoxy-2-p	HSP90AA1	YES
Scutellariae Radix	MOL000228	(2R)-7-hydroxy-5-methoxy-2-p	PRKACA	NO
Scutellariae Radix	MOL000228	(2R)-7-hydroxy-5-methoxy-2-p	PKIA	NO
Scutellariae Radix	MOL000228	(2R)-7-hydroxy-5-methoxy-2-p	CHRNA7	NO
Scutellariae Radix	MOL000228	(2R)-7-hydroxy-5-methoxy-2-p	MAOB	NO
Scutellariae Radix	MOL000228	(2R)-7-hydroxy-5-methoxy-2-p	CALM3	NO
licorice	MOL004805	(2S)-2-[4-hydroxy-3-(3-methy	NOS2	YES
licorice	MOL004805	(2S)-2-[4-hydroxy-3-(3-methy	KCNH2	NO
licorice	MOL004805	(2S)-2-[4-hydroxy-3-(3-methy	ESR1	YES
licorice	MOL004805	(2S)-2-[4-hydroxy-3-(3-methy	AR	YES
licorice	MOL004805	(2S)-2-[4-hydroxy-3-(3-methy	PPARG	NO
licorice	MOL004805	(2S)-2-[4-hydroxy-3-(3-methy	F10	YES
licorice	MOL004805	(2S)-2-[4-hydroxy-3-(3-methy	PTGS2	YES
licorice	MOL004805	(2S)-2-[4-hydroxy-3-(3-methy	ESR2	YES
licorice	MOL004805	(2S)-2-[4-hydroxy-3-(3-methy	MAPK14	YES
licorice	MOL004805	(2S)-2-[4-hydroxy-3-(3-methy	GSK3B	YES
licorice	MOL004805	(2S)-2-[4-hydroxy-3-(3-methy	CALM3	NO
licorice	MOL004824	(2S)-6-(2,4-dihydroxyphenyl)	NOS2	YES

licorice	MOL004824	(2S)-6-(2,4-dihydroxyphenyl)	F2	YES
licorice	MOL004824	(2S)-6-(2,4-dihydroxyphenyl)	ESR1	YES
licorice	MOL004824	(2S)-6-(2,4-dihydroxyphenyl)	AR	YES
licorice	MOL004824	(2S)-6-(2,4-dihydroxyphenyl)	PPARG	NO
licorice	MOL004824	(2S)-6-(2,4-dihydroxyphenyl)	F10	YES
licorice	MOL004824	(2S)-6-(2,4-dihydroxyphenyl)	PTGS2	YES
licorice	MOL004824	(2S)-6-(2,4-dihydroxyphenyl)	F7	NO
licorice	MOL004824	(2S)-6-(2,4-dihydroxyphenyl)	KDR	YES
licorice	MOL004824	(2S)-6-(2,4-dihydroxyphenyl)	ACHE	NO
licorice	MOL004824	(2S)-6-(2,4-dihydroxyphenyl)	TOP2A	NO
licorice	MOL004824	(2S)-6-(2,4-dihydroxyphenyl)	ESR2	YES
licorice	MOL004824	(2S)-6-(2,4-dihydroxyphenyl)	DPP4	NO
licorice	MOL004824	(2S)-6-(2,4-dihydroxyphenyl)	MAPK14	YES
licorice	MOL004824	(2S)-6-(2,4-dihydroxyphenyl)	GSK3B	YES
licorice	MOL004824	(2S)-6-(2,4-dihydroxyphenyl)	CDK2	NO
licorice	MOL004824	(2S)-6-(2,4-dihydroxyphenyl)	CHEK1	NO
licorice	MOL004824	(2S)-6-(2,4-dihydroxyphenyl)	PRSS1	NO
licorice	MOL004824	(2S)-6-(2,4-dihydroxyphenyl)	CCNA2	NO
licorice	MOL004824	(2S)-6-(2,4-dihydroxyphenyl)	CALM3	NO
licorice	MOL004945	(2S)-7-hydroxy-2-(4-hydroxyp	NOS2	YES
licorice	MOL004945	(2S)-7-hydroxy-2-(4-hydroxyp	PTGS1	NO
licorice	MOL004945	(2S)-7-hydroxy-2-(4-hydroxyp	ESR1	YES
licorice	MOL004945	(2S)-7-hydroxy-2-(4-hydroxyp	SCN5A	NO
licorice	MOL004945	(2S)-7-hydroxy-2-(4-hydroxyp	F10	YES
licorice	MOL004945	(2S)-7-hydroxy-2-(4-hydroxyp	PTGS2	YES
licorice	MOL004945	(2S)-7-hydroxy-2-(4-hydroxyp	PDE3A	NO
licorice	MOL004945	(2S)-7-hydroxy-2-(4-hydroxyp	ADRA1B	NO
licorice	MOL004945	(2S)-7-hydroxy-2-(4-hydroxyp	ADRB2	NO
licorice	MOL004945	(2S)-7-hydroxy-2-(4-hydroxyp	ESR2	YES
licorice	MOL004945	(2S)-7-hydroxy-2-(4-hydroxyp	HSP90AA1	YES
licorice	MOL004945	(2S)-7-hydroxy-2-(4-hydroxyp	CALM3	NO
Codonopsis Radix	MOL008407	(8S,9S,10R,13R,14S,17R)-17-[	PGR	NO
Codonopsis Radix	MOL008407	(8S,9S,10R,13R,14S,17R)-17-[	NR3C2	NO
licorice	MOL004815	(E)-1-(2,4-dihydroxyphenyl)-	NOS2	YES
licorice	MOL004815	(E)-1-(2,4-dihydroxyphenyl)-	PTGS1	NO
licorice	MOL004815	(E)-1-(2,4-dihydroxyphenyl)-	ESR1	YES
licorice	MOL004815	(E)-1-(2,4-dihydroxyphenyl)-	AR	YES
licorice	MOL004815	(E)-1-(2,4-dihydroxyphenyl)-	SCN5A	NO
licorice	MOL004815	(E)-1-(2,4-dihydroxyphenyl)-	PPARG	NO
licorice	MOL004815	(E)-1-(2,4-dihydroxyphenyl)-	F10	YES
licorice	MOL004815	(E)-1-(2,4-dihydroxyphenyl)-	PTGS2	YES
licorice	MOL004815	(E)-1-(2,4-dihydroxyphenyl)-	CA2	NO
licorice	MOL004815	(E)-1-(2,4-dihydroxyphenyl)-	RXRA	NO
licorice	MOL004815	(E)-1-(2,4-dihydroxyphenyl)-	ADRA1B	NO
licorice	MOL004815	(E)-1-(2,4-dihydroxyphenyl)-	ESR2	YES
licorice	MOL004815	(E)-1-(2,4-dihydroxyphenyl)-	MAPK14	YES
licorice	MOL004815	(E)-1-(2,4-dihydroxyphenyl)-	GSK3B	YES
licorice	MOL004815	(E)-1-(2,4-dihydroxyphenyl)-	CDK2	NO
licorice	MOL004815	(E)-1-(2,4-dihydroxyphenyl)-	CHEK1	NO
licorice	MOL004815	(E)-1-(2,4-dihydroxyphenyl)-	CCNA2	NO
licorice	MOL004815	(E)-1-(2,4-dihydroxyphenyl)-	NCOA2	NO
licorice	MOL004815	(E)-1-(2,4-dihydroxyphenyl)-	CALM3	NO
licorice	MOL004898	(E)-3-[3,4-dihydroxy-5-(3-me	ESR1	YES
licorice	MOL004898	(E)-3-[3,4-dihydroxy-5-(3-me	AR	YES

licorice	MOL004898	(E)-3-[3,4-dihydroxy-5-(3-me	PPARG	NO
licorice	MOL004898	(E)-3-[3,4-dihydroxy-5-(3-me	PTGS2	YES
licorice	MOL004898	(E)-3-[3,4-dihydroxy-5-(3-me	MAPK14	YES
licorice	MOL004898	(E)-3-[3,4-dihydroxy-5-(3-me	GSK3B	YES
licorice	MOL004898	(E)-3-[3,4-dihydroxy-5-(3-me	HSP90AA1	YES
licorice	MOL004898	(E)-3-[3,4-dihydroxy-5-(3-me	CDK2	NO
licorice	MOL004898	(E)-3-[3,4-dihydroxy-5-(3-me	CCNA2	NO
licorice	MOL004898	(E)-3-[3,4-dihydroxy-5-(3-me	NCOA2	NO
licorice	MOL004898	(E)-3-[3,4-dihydroxy-5-(3-me	CALM3	NO
licorice	MOL004914	1,3-dihydroxy-8,9-dimethoxy-	ESR1	YES
licorice	MOL004914	1,3-dihydroxy-8,9-dimethoxy-	AR	YES
licorice	MOL004914	1,3-dihydroxy-8,9-dimethoxy-	PPARG	NO
licorice	MOL004914	1,3-dihydroxy-8,9-dimethoxy-	MAPK14	YES
licorice	MOL004914	1,3-dihydroxy-8,9-dimethoxy-	GSK3B	YES
licorice	MOL004914	1,3-dihydroxy-8,9-dimethoxy-	HSP90AA1	YES
licorice	MOL004914	1,3-dihydroxy-8,9-dimethoxy-	CDK2	NO
licorice	MOL004914	1,3-dihydroxy-8,9-dimethoxy-	CHEK1	NO
licorice	MOL004914	1,3-dihydroxy-8,9-dimethoxy-	PRKACA	NO
licorice	MOL004913	1,3-dihydroxy-9-methoxy-6-be	ESR1	YES
licorice	MOL004913	1,3-dihydroxy-9-methoxy-6-be	PPARG	NO
licorice	MOL004913	1,3-dihydroxy-9-methoxy-6-be	ESR2	YES
licorice	MOL004913	1,3-dihydroxy-9-methoxy-6-be	MAPK14	YES
licorice	MOL004913	1,3-dihydroxy-9-methoxy-6-be	GSK3B	YES
licorice	MOL004913	1,3-dihydroxy-9-methoxy-6-be	HSP90AA1	YES
licorice	MOL004913	1,3-dihydroxy-9-methoxy-6-be	CDK2	NO
licorice	MOL004913	1,3-dihydroxy-9-methoxy-6-be	CHEK1	NO
licorice	MOL004913	1,3-dihydroxy-9-methoxy-6-be	PRKACA	NO
licorice	MOL004913	1,3-dihydroxy-9-methoxy-6-be	CCNA2	NO
Scutellariae Radix	MOL010415	11,13-Eicosadienoic acid, me	NCOA2	NO
Codonopsis Radix	MOL008411	11-Hydroxyrankinidine	ESR1	YES
Codonopsis Radix	MOL008411	11-Hydroxyrankinidine	SCN5A	NO
Codonopsis Radix	MOL008411	11-Hydroxyrankinidine	OPRM1	NO
Codonopsis Radix	MOL008411	11-Hydroxyrankinidine	CDK2	NO
licorice	MOL004959	1-Methoxyphaseollidin	NOS2	YES
licorice	MOL004959	1-Methoxyphaseollidin	PTGS1	NO
licorice	MOL004959	1-Methoxyphaseollidin	F2	YES
licorice	MOL004959	1-Methoxyphaseollidin	KCNH2	NO
licorice	MOL004959	1-Methoxyphaseollidin	ESR1	YES
licorice	MOL004959	1-Methoxyphaseollidin	AR	YES
licorice	MOL004959	1-Methoxyphaseollidin	SCN5A	NO
licorice	MOL004959	1-Methoxyphaseollidin	PPARG	NO
licorice	MOL004959	1-Methoxyphaseollidin	F10	YES
licorice	MOL004959	1-Methoxyphaseollidin	PTGS2	YES
licorice	MOL004959	1-Methoxyphaseollidin	KDR	YES
licorice	MOL004959	1-Methoxyphaseollidin	RXRA	NO
licorice	MOL004959	1-Methoxyphaseollidin	ADRA1B	NO
licorice	MOL004959	1-Methoxyphaseollidin	ADRB2	NO
licorice	MOL004959	1-Methoxyphaseollidin	ADRA1D	NO
licorice	MOL004959	1-Methoxyphaseollidin	TOP2A	NO
licorice	MOL004959	1-Methoxyphaseollidin	ESR2	YES
licorice	MOL004959	1-Methoxyphaseollidin	MAPK14	YES
licorice	MOL004959	1-Methoxyphaseollidin	GSK3B	YES
licorice	MOL004959	1-Methoxyphaseollidin	HSP90AA1	YES
licorice	MOL004959	1-Methoxyphaseollidin	CDK2	NO

licorice	MOL004959	1-Methoxyphaseollidin	PRSS1	NO
licorice	MOL004959	1-Methoxyphaseollidin	CCNA2	NO
licorice	MOL004959	1-Methoxyphaseollidin	NCOA2	NO
licorice	MOL004959	1-Methoxyphaseollidin	NCOA1	NO
licorice	MOL004959	1-Methoxyphaseollidin	CALM3	NO
licorice	MOL004866	2-(3,4-dihydroxyphenyl)-5,7-	F2	YES
licorice	MOL004866	2-(3,4-dihydroxyphenyl)-5,7-	AR	YES
licorice	MOL004866	2-(3,4-dihydroxyphenyl)-5,7-	SCN5A	NO
licorice	MOL004866	2-(3,4-dihydroxyphenyl)-5,7-	PPARG	NO
licorice	MOL004866	2-(3,4-dihydroxyphenyl)-5,7-	F10	YES
licorice	MOL004866	2-(3,4-dihydroxyphenyl)-5,7-	PTGS2	YES
licorice	MOL004866	2-(3,4-dihydroxyphenyl)-5,7-	F7	NO
licorice	MOL004866	2-(3,4-dihydroxyphenyl)-5,7-	ADRB2	NO
licorice	MOL004866	2-(3,4-dihydroxyphenyl)-5,7-	DPP4	NO
licorice	MOL004866	2-(3,4-dihydroxyphenyl)-5,7-	HSP90AA1	YES
licorice	MOL004866	2-(3,4-dihydroxyphenyl)-5,7-	CDK2	NO
licorice	MOL004866	2-(3,4-dihydroxyphenyl)-5,7-	CHEK1	NO
licorice	MOL004866	2-(3,4-dihydroxyphenyl)-5,7-	PRSS1	NO
licorice	MOL004866	2-(3,4-dihydroxyphenyl)-5,7-	CCNA2	NO
licorice	MOL004866	2-(3,4-dihydroxyphenyl)-5,7-	CALM3	NO
licorice	MOL004978	2-[(3R)-8,8-dimethyl-3,4-dih	NOS2	YES
licorice	MOL004978	2-[(3R)-8,8-dimethyl-3,4-dih	PTGS1	NO
licorice	MOL004978	2-[(3R)-8,8-dimethyl-3,4-dih	CHRM3	NO
licorice	MOL004978	2-[(3R)-8,8-dimethyl-3,4-dih	KCNH2	NO
licorice	MOL004978	2-[(3R)-8,8-dimethyl-3,4-dih	CHRM1	NO
licorice	MOL004978	2-[(3R)-8,8-dimethyl-3,4-dih	ESR1	YES
licorice	MOL004978	2-[(3R)-8,8-dimethyl-3,4-dih	AR	YES
licorice	MOL004978	2-[(3R)-8,8-dimethyl-3,4-dih	SCN5A	NO
licorice	MOL004978	2-[(3R)-8,8-dimethyl-3,4-dih	PPARG	NO
licorice	MOL004978	2-[(3R)-8,8-dimethyl-3,4-dih	F10	YES
licorice	MOL004978	2-[(3R)-8,8-dimethyl-3,4-dih	PTGS2	YES
licorice	MOL004978	2-[(3R)-8,8-dimethyl-3,4-dih	RXRA	NO
licorice	MOL004978	2-[(3R)-8,8-dimethyl-3,4-dih	ACHE	NO
licorice	MOL004978	2-[(3R)-8,8-dimethyl-3,4-dih	ADRA1B	NO
licorice	MOL004978	2-[(3R)-8,8-dimethyl-3,4-dih	SLC6A3	NO
licorice	MOL004978	2-[(3R)-8,8-dimethyl-3,4-dih	ADRB2	NO
licorice	MOL004978	2-[(3R)-8,8-dimethyl-3,4-dih	ESR2	YES
licorice	MOL004978	2-[(3R)-8,8-dimethyl-3,4-dih	MAPK14	YES
licorice	MOL004978	2-[(3R)-8,8-dimethyl-3,4-dih	GSK3B	YES
licorice	MOL004978	2-[(3R)-8,8-dimethyl-3,4-dih	CDK2	NO
licorice	MOL004978	2-[(3R)-8,8-dimethyl-3,4-dih	CHEK1	NO
licorice	MOL004978	2-[(3R)-8,8-dimethyl-3,4-dih	PRKACA	NO
licorice	MOL004978	2-[(3R)-8,8-dimethyl-3,4-dih	METTL1	NO
licorice	MOL004978	2-[(3R)-8,8-dimethyl-3,4-dih	PRSS1	NO
licorice	MOL004978	2-[(3R)-8,8-dimethyl-3,4-dih	CCNA2	NO
licorice	MOL004978	2-[(3R)-8,8-dimethyl-3,4-dih	NCOA2	NO
licorice	MOL004978	2-[(3R)-8,8-dimethyl-3,4-dih	NCOA1	NO
licorice	MOL004978	2-[(3R)-8,8-dimethyl-3,4-dih	CALM3	NO
Coicis Semen	MOL008121	2-Monoolein	NCOA2	NO
licorice	MOL004849	3-(2,4-dihydroxyphenyl)-8-(1	NOS2	YES
licorice	MOL004849	3-(2,4-dihydroxyphenyl)-8-(1	F2	YES
licorice	MOL004849	3-(2,4-dihydroxyphenyl)-8-(1	KCNH2	NO
licorice	MOL004849	3-(2,4-dihydroxyphenyl)-8-(1	ESR1	YES
licorice	MOL004849	3-(2,4-dihydroxyphenyl)-8-(1	AR	YES

licorice	MOL004849	3-(2,4-dihydroxyphenyl)-8-(1	PPARG	NO
licorice	MOL004849	3-(2,4-dihydroxyphenyl)-8-(1	F10	YES
licorice	MOL004849	3-(2,4-dihydroxyphenyl)-8-(1	PTGS2	YES
licorice	MOL004849	3-(2,4-dihydroxyphenyl)-8-(1	F7	NO
licorice	MOL004849	3-(2,4-dihydroxyphenyl)-8-(1	KDR	YES
licorice	MOL004849	3-(2,4-dihydroxyphenyl)-8-(1	TOP2A	NO
licorice	MOL004849	3-(2,4-dihydroxyphenyl)-8-(1	ESR2	YES
licorice	MOL004849	3-(2,4-dihydroxyphenyl)-8-(1	DPP4	NO
licorice	MOL004849	3-(2,4-dihydroxyphenyl)-8-(1	MAPK14	YES
licorice	MOL004849	3-(2,4-dihydroxyphenyl)-8-(1	GSK3B	YES
licorice	MOL004849	3-(2,4-dihydroxyphenyl)-8-(1	HSP90AA1	YES
licorice	MOL004849	3-(2,4-dihydroxyphenyl)-8-(1	CDK2	NO
licorice	MOL004849	3-(2,4-dihydroxyphenyl)-8-(1	CHEK1	NO
licorice	MOL004849	3-(2,4-dihydroxyphenyl)-8-(1	PRSS1	NO
licorice	MOL004849	3-(2,4-dihydroxyphenyl)-8-(1	NCOA2	NO
licorice	MOL004849	3-(2,4-dihydroxyphenyl)-8-(1	NCOA1	NO
licorice	MOL004849	3-(2,4-dihydroxyphenyl)-8-(1	CALM3	NO
licorice	MOL004863	3-(3,4-dihydroxyphenyl)-5,7-	NOS2	YES
licorice	MOL004863	3-(3,4-dihydroxyphenyl)-5,7-	F2	YES
licorice	MOL004863	3-(3,4-dihydroxyphenyl)-5,7-	ESR1	YES
licorice	MOL004863	3-(3,4-dihydroxyphenyl)-5,7-	AR	YES
licorice	MOL004863	3-(3,4-dihydroxyphenyl)-5,7-	PPARG	NO
licorice	MOL004863	3-(3,4-dihydroxyphenyl)-5,7-	F10	YES
licorice	MOL004863	3-(3,4-dihydroxyphenyl)-5,7-	PTGS2	YES
licorice	MOL004863	3-(3,4-dihydroxyphenyl)-5,7-	PTPN1	YES
licorice	MOL004863	3-(3,4-dihydroxyphenyl)-5,7-	MAPK14	YES
licorice	MOL004863	3-(3,4-dihydroxyphenyl)-5,7-	GSK3B	YES
licorice	MOL004863	3-(3,4-dihydroxyphenyl)-5,7-	HSP90AA1	YES
licorice	MOL004863	3-(3,4-dihydroxyphenyl)-5,7-	CDK2	NO
licorice	MOL004863	3-(3,4-dihydroxyphenyl)-5,7-	CHEK1	NO
licorice	MOL004863	3-(3,4-dihydroxyphenyl)-5,7-	PRSS1	NO
licorice	MOL004863	3-(3,4-dihydroxyphenyl)-5,7-	CCNA2	NO
licorice	MOL004863	3-(3,4-dihydroxyphenyl)-5,7-	NCOA2	NO
licorice	MOL004863	3-(3,4-dihydroxyphenyl)-5,7-	CALM3	NO
Pogostemon Cablin (	MOL005923	3,23-dihydroxy-12-oleanen-28	CA2	NO
Pogostemon Cablin (	MOL005923	3,23-dihydroxy-12-oleanen-28	TOP2A	NO
Radix Bupleuri	MOL004598	3,5,6,7-tetramethoxy-2-(3,4,	F2	YES
Radix Bupleuri	MOL004598	3,5,6,7-tetramethoxy-2-(3,4,	ESR1	YES
Radix Bupleuri	MOL004598	3,5,6,7-tetramethoxy-2-(3,4,	AR	YES
Radix Bupleuri	MOL004598	3,5,6,7-tetramethoxy-2-(3,4,	F10	YES
Radix Bupleuri	MOL004598	3,5,6,7-tetramethoxy-2-(3,4,	PTGS2	YES
Radix Bupleuri	MOL004598	3,5,6,7-tetramethoxy-2-(3,4,	F7	NO
Radix Bupleuri	MOL004598	3,5,6,7-tetramethoxy-2-(3,4,	ACHE	NO
Radix Bupleuri	MOL004598	3,5,6,7-tetramethoxy-2-(3,4,	TOP2A	NO
Radix Bupleuri	MOL004598	3,5,6,7-tetramethoxy-2-(3,4,	ESR2	YES
Radix Bupleuri	MOL004598	3,5,6,7-tetramethoxy-2-(3,4,	PRSS1	NO
Radix Bupleuri	MOL004598	3,5,6,7-tetramethoxy-2-(3,4,	NCOA2	NO
Radix Bupleuri	MOL004598	3,5,6,7-tetramethoxy-2-(3,4,	CALM3	NO
Codonopsis Radix	MOL007059	3-beta-Hydroxymethyllenetans	DRD1	NO
Codonopsis Radix	MOL007059	3-beta-Hydroxymethyllenetans	F2	YES
Codonopsis Radix	MOL007059	3-beta-Hydroxymethyllenetans	CHRM1	NO
Codonopsis Radix	MOL007059	3-beta-Hydroxymethyllenetans	PTGS2	YES
Codonopsis Radix	MOL007059	3-beta-Hydroxymethyllenetans	CA2	NO
Codonopsis Radix	MOL007059	3-beta-Hydroxymethyllenetans	RXRA	NO

Codonopsis Radix	MOL007059	3-beta-Hydroxymethyllenetans	OPRD1	NO
Codonopsis Radix	MOL007059	3-beta-Hydroxymethyllenetans	ACHE	NO
Codonopsis Radix	MOL007059	3-beta-Hydroxymethyllenetans	ADRA1A	NO
Codonopsis Radix	MOL007059	3-beta-Hydroxymethyllenetans	ADRB2	NO
Codonopsis Radix	MOL007059	3-beta-Hydroxymethyllenetans	OPRM1	NO
Codonopsis Radix	MOL007059	3-beta-Hydroxymethyllenetans	DPP4	NO
Codonopsis Radix	MOL007059	3-beta-Hydroxymethyllenetans	HSP90AA1	YES
Codonopsis Radix	MOL007059	3-beta-Hydroxymethyllenetans	CHRNA7	NO
Codonopsis Radix	MOL007059	3-beta-Hydroxymethyllenetans	IGHG1	NO
Codonopsis Radix	MOL007059	3-beta-Hydroxymethyllenetans	PRSS1	NO
Codonopsis Radix	MOL007059	3-beta-Hydroxymethyllenetans	NCOA1	NO
licorice	MOL004966	3'-Hydroxy-4'-O-Methylglabri	NOS2	YES
licorice	MOL004966	3'-Hydroxy-4'-O-Methylglabri	PTGS1	NO
licorice	MOL004966	3'-Hydroxy-4'-O-Methylglabri	KCNH2	NO
licorice	MOL004966	3'-Hydroxy-4'-O-Methylglabri	ESR1	YES
licorice	MOL004966	3'-Hydroxy-4'-O-Methylglabri	AR	YES
licorice	MOL004966	3'-Hydroxy-4'-O-Methylglabri	SCN5A	NO
licorice	MOL004966	3'-Hydroxy-4'-O-Methylglabri	PPARG	NO
licorice	MOL004966	3'-Hydroxy-4'-O-Methylglabri	F10	YES
licorice	MOL004966	3'-Hydroxy-4'-O-Methylglabri	PTGS2	YES
licorice	MOL004966	3'-Hydroxy-4'-O-Methylglabri	F7	NO
licorice	MOL004966	3'-Hydroxy-4'-O-Methylglabri	KDR	YES
licorice	MOL004966	3'-Hydroxy-4'-O-Methylglabri	ADRA1B	NO
licorice	MOL004966	3'-Hydroxy-4'-O-Methylglabri	ADRB2	NO
licorice	MOL004966	3'-Hydroxy-4'-O-Methylglabri	TOP2A	NO
licorice	MOL004966	3'-Hydroxy-4'-O-Methylglabri	ESR2	YES
licorice	MOL004966	3'-Hydroxy-4'-O-Methylglabri	MAPK14	YES
licorice	MOL004966	3'-Hydroxy-4'-O-Methylglabri	GSK3B	YES
licorice	MOL004966	3'-Hydroxy-4'-O-Methylglabri	HSP90AA1	YES
licorice	MOL004966	3'-Hydroxy-4'-O-Methylglabri	CDK2	NO
licorice	MOL004966	3'-Hydroxy-4'-O-Methylglabri	CHEK1	NO
licorice	MOL004966	3'-Hydroxy-4'-O-Methylglabri	PRKACA	NO
licorice	MOL004966	3'-Hydroxy-4'-O-Methylglabri	PRSS1	NO
licorice	MOL004966	3'-Hydroxy-4'-O-Methylglabri	CCNA2	NO
licorice	MOL004966	3'-Hydroxy-4'-O-Methylglabri	NCOA2	NO
licorice	MOL004966	3'-Hydroxy-4'-O-Methylglabri	NCOA1	NO
licorice	MOL004966	3'-Hydroxy-4'-O-Methylglabri	CALM3	NO
Radix Puerariae	MOL002959	3'-Methoxydaidzein	NOS2	YES
Radix Puerariae	MOL002959	3'-Methoxydaidzein	PTGS1	NO
Radix Puerariae	MOL002959	3'-Methoxydaidzein	ESR1	YES
Radix Puerariae	MOL002959	3'-Methoxydaidzein	AR	YES
Radix Puerariae	MOL002959	3'-Methoxydaidzein	PPARG	NO
Radix Puerariae	MOL002959	3'-Methoxydaidzein	PTGS2	YES
Radix Puerariae	MOL002959	3'-Methoxydaidzein	RXRA	NO
Radix Puerariae	MOL002959	3'-Methoxydaidzein	ESR2	YES
Radix Puerariae	MOL002959	3'-Methoxydaidzein	MAPK14	YES
Radix Puerariae	MOL002959	3'-Methoxydaidzein	GSK3B	YES
Radix Puerariae	MOL002959	3'-Methoxydaidzein	HSP90AA1	YES
Radix Puerariae	MOL002959	3'-Methoxydaidzein	CDK2	NO
Radix Puerariae	MOL002959	3'-Methoxydaidzein	CHEK1	NO
Radix Puerariae	MOL002959	3'-Methoxydaidzein	PRKACA	NO
Radix Puerariae	MOL002959	3'-Methoxydaidzein	PRSS1	NO
Radix Puerariae	MOL002959	3'-Methoxydaidzein	CCNA2	NO
Radix Puerariae	MOL002959	3'-Methoxydaidzein	NCOA1	NO

Radix Puerariae	MOL002959	3'-Methoxydaidzein	CALM3	NO
licorice	MOL004974	3'-Methoxyglabridin	NOS2	YES
licorice	MOL004974	3'-Methoxyglabridin	PTGS1	NO
licorice	MOL004974	3'-Methoxyglabridin	KCNH2	NO
licorice	MOL004974	3'-Methoxyglabridin	ESR1	YES
licorice	MOL004974	3'-Methoxyglabridin	AR	YES
licorice	MOL004974	3'-Methoxyglabridin	SCN5A	NO
licorice	MOL004974	3'-Methoxyglabridin	PPARG	NO
licorice	MOL004974	3'-Methoxyglabridin	F10	YES
licorice	MOL004974	3'-Methoxyglabridin	PTGS2	YES
licorice	MOL004974	3'-Methoxyglabridin	F7	NO
licorice	MOL004974	3'-Methoxyglabridin	RXRA	NO
licorice	MOL004974	3'-Methoxyglabridin	ACHE	NO
licorice	MOL004974	3'-Methoxyglabridin	ADRA1B	NO
licorice	MOL004974	3'-Methoxyglabridin	ADRB2	NO
licorice	MOL004974	3'-Methoxyglabridin	TOP2A	NO
licorice	MOL004974	3'-Methoxyglabridin	ESR2	YES
licorice	MOL004974	3'-Methoxyglabridin	MAPK14	YES
licorice	MOL004974	3'-Methoxyglabridin	GSK3B	YES
licorice	MOL004974	3'-Methoxyglabridin	HSP90AA1	YES
licorice	MOL004974	3'-Methoxyglabridin	CDK2	NO
licorice	MOL004974	3'-Methoxyglabridin	CHEK1	NO
licorice	MOL004974	3'-Methoxyglabridin	PRSS1	NO
licorice	MOL004974	3'-Methoxyglabridin	CCNA2	NO
licorice	MOL004974	3'-Methoxyglabridin	NCOA2	NO
licorice	MOL004974	3'-Methoxyglabridin	NCOA1	NO
licorice	MOL004974	3'-Methoxyglabridin	CALM3	NO
Scutellariae Radix	MOL002917	5,2',6'-Trihydroxy-7,8-dimet	NOS2	YES
Scutellariae Radix	MOL002917	5,2',6'-Trihydroxy-7,8-dimet	PTGS1	NO
Scutellariae Radix	MOL002917	5,2',6'-Trihydroxy-7,8-dimet	AR	YES
Scutellariae Radix	MOL002917	5,2',6'-Trihydroxy-7,8-dimet	SCN5A	NO
Scutellariae Radix	MOL002917	5,2',6'-Trihydroxy-7,8-dimet	F10	YES
Scutellariae Radix	MOL002917	5,2',6'-Trihydroxy-7,8-dimet	PTGS2	YES
Scutellariae Radix	MOL002917	5,2',6'-Trihydroxy-7,8-dimet	TOP2A	NO
Scutellariae Radix	MOL002917	5,2',6'-Trihydroxy-7,8-dimet	ESR2	YES
Scutellariae Radix	MOL002917	5,2',6'-Trihydroxy-7,8-dimet	DPP4	NO
Scutellariae Radix	MOL002917	5,2',6'-Trihydroxy-7,8-dimet	HSP90AA1	YES
Scutellariae Radix	MOL002917	5,2',6'-Trihydroxy-7,8-dimet	CDK2	NO
Scutellariae Radix	MOL002917	5,2',6'-Trihydroxy-7,8-dimet	CHEK1	NO
Scutellariae Radix	MOL002917	5,2',6'-Trihydroxy-7,8-dimet	PRSS1	NO
Scutellariae Radix	MOL002917	5,2',6'-Trihydroxy-7,8-dimet	NCOA2	NO
Scutellariae Radix	MOL002917	5,2',6'-Trihydroxy-7,8-dimet	CALM3	NO
Scutellariae Radix	MOL000552	5,2'-Dihydroxy-6,7,8-trimeth	NOS2	YES
Scutellariae Radix	MOL000552	5,2'-Dihydroxy-6,7,8-trimeth	PTGS1	NO
Scutellariae Radix	MOL000552	5,2'-Dihydroxy-6,7,8-trimeth	F2	YES
Scutellariae Radix	MOL000552	5,2'-Dihydroxy-6,7,8-trimeth	KCNH2	NO
Scutellariae Radix	MOL000552	5,2'-Dihydroxy-6,7,8-trimeth	AR	YES
Scutellariae Radix	MOL000552	5,2'-Dihydroxy-6,7,8-trimeth	SCN5A	NO
Scutellariae Radix	MOL000552	5,2'-Dihydroxy-6,7,8-trimeth	F10	YES
Scutellariae Radix	MOL000552	5,2'-Dihydroxy-6,7,8-trimeth	PTGS2	YES
Scutellariae Radix	MOL000552	5,2'-Dihydroxy-6,7,8-trimeth	F7	NO
Scutellariae Radix	MOL000552	5,2'-Dihydroxy-6,7,8-trimeth	TOP2A	NO
Scutellariae Radix	MOL000552	5,2'-Dihydroxy-6,7,8-trimeth	ESR2	YES
Scutellariae Radix	MOL000552	5,2'-Dihydroxy-6,7,8-trimeth	DPP4	NO

Scutellariae Radix	MOL000552	5,2'-Dihydroxy-6,7,8-trimeth	PPARD	NO
Scutellariae Radix	MOL000552	5,2'-Dihydroxy-6,7,8-trimeth	HSP90AA1	YES
Scutellariae Radix	MOL000552	5,2'-Dihydroxy-6,7,8-trimeth	PRSS1	NO
Scutellariae Radix	MOL000552	5,2'-Dihydroxy-6,7,8-trimeth	NCOA2	NO
Scutellariae Radix	MOL000552	5,2'-Dihydroxy-6,7,8-trimeth	CALM3	NO
Scutellariae Radix	MOL000552	5,2'-Dihydroxy-6,7,8-trimeth	KDR	YES
Scutellariae Radix	MOL000552	5,2'-Dihydroxy-6,7,8-trimeth	NCOA1	NO
Scutellariae Radix	MOL002909	5,7,2,5-tetrahydroxy-8,6-din	NOS2	YES
Scutellariae Radix	MOL002909	5,7,2,5-tetrahydroxy-8,6-din	F2	YES
Scutellariae Radix	MOL002909	5,7,2,5-tetrahydroxy-8,6-din	AR	YES
Scutellariae Radix	MOL002909	5,7,2,5-tetrahydroxy-8,6-din	F10	YES
Scutellariae Radix	MOL002909	5,7,2,5-tetrahydroxy-8,6-din	PTGS2	YES
Scutellariae Radix	MOL002909	5,7,2,5-tetrahydroxy-8,6-din	PTPN1	YES
Scutellariae Radix	MOL002909	5,7,2,5-tetrahydroxy-8,6-din	TOP2A	NO
Scutellariae Radix	MOL002909	5,7,2,5-tetrahydroxy-8,6-din	DPP4	NO
Scutellariae Radix	MOL002909	5,7,2,5-tetrahydroxy-8,6-din	PYGM	NO
Scutellariae Radix	MOL002909	5,7,2,5-tetrahydroxy-8,6-din	HSP90AA1	YES
Scutellariae Radix	MOL002909	5,7,2,5-tetrahydroxy-8,6-din	PRSS1	NO
Scutellariae Radix	MOL002909	5,7,2,5-tetrahydroxy-8,6-din	NCOA2	NO
Scutellariae Radix	MOL002925	5,7,2',6'-Tetrahydroxyflavon	PTGS1	NO
Scutellariae Radix	MOL002925	5,7,2',6'-Tetrahydroxyflavon	AR	YES
Scutellariae Radix	MOL002925	5,7,2',6'-Tetrahydroxyflavon	PTGS2	YES
Scutellariae Radix	MOL002925	5,7,2',6'-Tetrahydroxyflavon	DPP4	NO
Scutellariae Radix	MOL002925	5,7,2',6'-Tetrahydroxyflavon	HSP90AA1	YES
Scutellariae Radix	MOL002925	5,7,2',6'-Tetrahydroxyflavon	PRKACA	NO
Scutellariae Radix	MOL012245	5,7,4'-trihydroxy-6-methoxyf	PTGS1	NO
Scutellariae Radix	MOL012245	5,7,4'-trihydroxy-6-methoxyf	PTGS2	YES
Scutellariae Radix	MOL012245	5,7,4'-trihydroxy-6-methoxyf	CA2	NO
Scutellariae Radix	MOL012245	5,7,4'-trihydroxy-6-methoxyf	HSP90AA1	YES
Scutellariae Radix	MOL012245	5,7,4'-trihydroxy-6-methoxyf	PRKACA	NO
Scutellariae Radix	MOL012245	5,7,4'-trihydroxy-6-methoxyf	CALM3	NO
Scutellariae Radix	MOL012246	5,7,4'-trihydroxy-8-methoxyf	PTGS1	NO
Scutellariae Radix	MOL012246	5,7,4'-trihydroxy-8-methoxyf	PTGS2	YES
Scutellariae Radix	MOL012246	5,7,4'-trihydroxy-8-methoxyf	CA2	NO
Scutellariae Radix	MOL012246	5,7,4'-trihydroxy-8-methoxyf	HSP90AA1	YES
Scutellariae Radix	MOL012246	5,7,4'-trihydroxy-8-methoxyf	PRKACA	NO
Scutellariae Radix	MOL002933	5,7,4'-Trihydroxy-8-methoxyf	NOS2	YES
Scutellariae Radix	MOL002933	5,7,4'-Trihydroxy-8-methoxyf	PTGS1	NO
Scutellariae Radix	MOL002933	5,7,4'-Trihydroxy-8-methoxyf	ESR1	YES
Scutellariae Radix	MOL002933	5,7,4'-Trihydroxy-8-methoxyf	AR	YES
Scutellariae Radix	MOL002933	5,7,4'-Trihydroxy-8-methoxyf	PPARG	NO
Scutellariae Radix	MOL002933	5,7,4'-Trihydroxy-8-methoxyf	PTGS2	YES
Scutellariae Radix	MOL002933	5,7,4'-Trihydroxy-8-methoxyf	DPP4	NO
Scutellariae Radix	MOL002933	5,7,4'-Trihydroxy-8-methoxyf	PYGM	NO
Scutellariae Radix	MOL002933	5,7,4'-Trihydroxy-8-methoxyf	MAPK14	YES
Scutellariae Radix	MOL002933	5,7,4'-Trihydroxy-8-methoxyf	GSK3B	YES
Scutellariae Radix	MOL002933	5,7,4'-Trihydroxy-8-methoxyf	HSP90AA1	YES
Scutellariae Radix	MOL002933	5,7,4'-Trihydroxy-8-methoxyf	CDK2	NO
Scutellariae Radix	MOL002933	5,7,4'-Trihydroxy-8-methoxyf	CHEK1	NO
Scutellariae Radix	MOL002933	5,7,4'-Trihydroxy-8-methoxyf	PRSS1	NO
Scutellariae Radix	MOL002933	5,7,4'-Trihydroxy-8-methoxyf	NCOA2	NO
Scutellariae Radix	MOL002933	5,7,4'-Trihydroxy-8-methoxyf	CALM3	NO
Scutellariae Radix	MOL002933	5,7,4'-Trihydroxy-8-methoxyf	PRKACA	NO
licorice	MOL004864	5,7-dihydroxy-3-(4-methoxyph	NOS2	YES

licorice	MOL004864	5,7-dihydroxy-3-(4-methoxyph	KCNH2	NO
licorice	MOL004864	5,7-dihydroxy-3-(4-methoxyph	ESR1	YES
licorice	MOL004864	5,7-dihydroxy-3-(4-methoxyph	AR	YES
licorice	MOL004864	5,7-dihydroxy-3-(4-methoxyph	PPARG	NO
licorice	MOL004864	5,7-dihydroxy-3-(4-methoxyph	F10	YES
licorice	MOL004864	5,7-dihydroxy-3-(4-methoxyph	PTGS2	YES
licorice	MOL004864	5,7-dihydroxy-3-(4-methoxyph	TOP2A	NO
licorice	MOL004864	5,7-dihydroxy-3-(4-methoxyph	ESR2	YES
licorice	MOL004864	5,7-dihydroxy-3-(4-methoxyph	DPP4	NO
licorice	MOL004864	5,7-dihydroxy-3-(4-methoxyph	MAPK14	YES
licorice	MOL004864	5,7-dihydroxy-3-(4-methoxyph	GSK3B	YES
licorice	MOL004864	5,7-dihydroxy-3-(4-methoxyph	HSP90AA1	YES
licorice	MOL004864	5,7-dihydroxy-3-(4-methoxyph	CDK2	NO
licorice	MOL004864	5,7-dihydroxy-3-(4-methoxyph	CHEK1	NO
licorice	MOL004864	5,7-dihydroxy-3-(4-methoxyph	PRSS1	NO
licorice	MOL004864	5,7-dihydroxy-3-(4-methoxyph	CCNA2	NO
licorice	MOL004864	5,7-dihydroxy-3-(4-methoxyph	NCOA2	NO
licorice	MOL004864	5,7-dihydroxy-3-(4-methoxyph	CALM3	NO
Pogostemon Cablin (	MOL005911	5-Hydroxy-7,4’-dimethoxyflav	PTGS1	NO
Pogostemon Cablin (	MOL005911	5-Hydroxy-7,4’-dimethoxyflav	SCN5A	NO
Pogostemon Cablin (	MOL005911	5-Hydroxy-7,4’-dimethoxyflav	PTGS2	YES
Pogostemon Cablin (	MOL005911	5-Hydroxy-7,4’-dimethoxyflav	RXRA	NO
Pogostemon Cablin (	MOL005911	5-Hydroxy-7,4’-dimethoxyflav	ADRA1B	NO
Pogostemon Cablin (	MOL005911	5-Hydroxy-7,4’-dimethoxyflav	ADRB2	NO
Pogostemon Cablin (	MOL005911	5-Hydroxy-7,4’-dimethoxyflav	SLC6A4	YES
Pogostemon Cablin (	MOL005911	5-Hydroxy-7,4’-dimethoxyflav	HSP90AA1	YES
Pogostemon Cablin (	MOL005911	5-Hydroxy-7,4’-dimethoxyflav	LTA4H	NO
Pogostemon Cablin (	MOL005911	5-Hydroxy-7,4’-dimethoxyflav	CALM3	NO
Pogostemon Cablin (	MOL005911	5-Hydroxy-7,4’-dimethoxyflav	PDE3A	NO
licorice	MOL004989	6-prenylated eriodictyol	NOS2	YES
licorice	MOL004989	6-prenylated eriodictyol	ESR1	YES
licorice	MOL004989	6-prenylated eriodictyol	SCN5A	NO
licorice	MOL004989	6-prenylated eriodictyol	F10	YES
licorice	MOL004989	6-prenylated eriodictyol	PTGS2	YES
licorice	MOL004989	6-prenylated eriodictyol	F7	NO
licorice	MOL004989	6-prenylated eriodictyol	HSP90AA1	YES
licorice	MOL004989	6-prenylated eriodictyol	CALM3	NO
Codonopsis Radix	MOL008393	7-(beta-Xylosyl)cephalomanni	TUBB1	NO
licorice	MOL004990	7,2’,4’-trihydroxy—5-methox	NOS2	YES
licorice	MOL004990	7,2’,4’-trihydroxy—5-methox	PTGS1	NO
licorice	MOL004990	7,2’,4’-trihydroxy—5-methox	ESR1	YES
licorice	MOL004990	7,2’,4’-trihydroxy—5-methox	AR	YES
licorice	MOL004990	7,2’,4’-trihydroxy—5-methox	PPARG	NO
licorice	MOL004990	7,2’,4’-trihydroxy—5-methox	PTGS2	YES
licorice	MOL004990	7,2’,4’-trihydroxy—5-methox	ESR2	YES
licorice	MOL004990	7,2’,4’-trihydroxy—5-methox	DPP4	NO
licorice	MOL004990	7,2’,4’-trihydroxy—5-methox	MAPK14	YES
licorice	MOL004990	7,2’,4’-trihydroxy—5-methox	GSK3B	YES
licorice	MOL004990	7,2’,4’-trihydroxy—5-methox	HSP90AA1	YES
licorice	MOL004990	7,2’,4’-trihydroxy—5-methox	CDK2	NO
licorice	MOL004990	7,2’,4’-trihydroxy—5-methox	CHEK1	NO
licorice	MOL004990	7,2’,4’-trihydroxy—5-methox	PRKACA	NO
licorice	MOL004991	7-Acetoxy-2-methylisoflavone	NOS2	YES
licorice	MOL004991	7-Acetoxy-2-methylisoflavone	PTGS1	NO

licorice	MOL004991	7-Acetoxy-2-methylisoflavone	F2	YES
licorice	MOL004991	7-Acetoxy-2-methylisoflavone	ESR1	YES
licorice	MOL004991	7-Acetoxy-2-methylisoflavone	AR	YES
licorice	MOL004991	7-Acetoxy-2-methylisoflavone	SCN5A	NO
licorice	MOL004991	7-Acetoxy-2-methylisoflavone	PPARG	NO
licorice	MOL004991	7-Acetoxy-2-methylisoflavone	PTGS2	YES
licorice	MOL004991	7-Acetoxy-2-methylisoflavone	RXRA	NO
licorice	MOL004991	7-Acetoxy-2-methylisoflavone	ACHE	NO
licorice	MOL004991	7-Acetoxy-2-methylisoflavone	PDE3A	NO
licorice	MOL004991	7-Acetoxy-2-methylisoflavone	ADRA1B	NO
licorice	MOL004991	7-Acetoxy-2-methylisoflavone	ADRB2	NO
licorice	MOL004991	7-Acetoxy-2-methylisoflavone	ADRA1D	NO
licorice	MOL004991	7-Acetoxy-2-methylisoflavone	DPP4	NO
licorice	MOL004991	7-Acetoxy-2-methylisoflavone	MAPK14	YES
licorice	MOL004991	7-Acetoxy-2-methylisoflavone	GSK3B	YES
licorice	MOL004991	7-Acetoxy-2-methylisoflavone	HSP90AA1	YES
licorice	MOL004991	7-Acetoxy-2-methylisoflavone	CDK2	NO
licorice	MOL004991	7-Acetoxy-2-methylisoflavone	CHEK1	NO
licorice	MOL004991	7-Acetoxy-2-methylisoflavone	PRSS1	NO
licorice	MOL004991	7-Acetoxy-2-methylisoflavone	NCOA2	NO
licorice	MOL004991	7-Acetoxy-2-methylisoflavone	CALM3	NO
Codonopsis Radix	MOL003896	7-Methoxy-2-methyl isoflavon	NOS2	YES
Codonopsis Radix	MOL003896	7-Methoxy-2-methyl isoflavon	PTGS1	NO
Codonopsis Radix	MOL003896	7-Methoxy-2-methyl isoflavon	DRD1	NO
Codonopsis Radix	MOL003896	7-Methoxy-2-methyl isoflavon	CHRM3	NO
Codonopsis Radix	MOL003896	7-Methoxy-2-methyl isoflavon	F2	YES
Codonopsis Radix	MOL003896	7-Methoxy-2-methyl isoflavon	CHRM1	NO
Codonopsis Radix	MOL003896	7-Methoxy-2-methyl isoflavon	ESR1	YES
Codonopsis Radix	MOL003896	7-Methoxy-2-methyl isoflavon	AR	YES
Codonopsis Radix	MOL003896	7-Methoxy-2-methyl isoflavon	ADRB1	NO
Codonopsis Radix	MOL003896	7-Methoxy-2-methyl isoflavon	SCN5A	NO
Codonopsis Radix	MOL003896	7-Methoxy-2-methyl isoflavon	PPARG	NO
Codonopsis Radix	MOL003896	7-Methoxy-2-methyl isoflavon	PTGS2	YES
Codonopsis Radix	MOL003896	7-Methoxy-2-methyl isoflavon	RXRA	NO
Codonopsis Radix	MOL003896	7-Methoxy-2-methyl isoflavon	ACHE	NO
Codonopsis Radix	MOL003896	7-Methoxy-2-methyl isoflavon	PDE3A	NO
Codonopsis Radix	MOL003896	7-Methoxy-2-methyl isoflavon	ADRA1B	NO
Codonopsis Radix	MOL003896	7-Methoxy-2-methyl isoflavon	SLC6A3	NO
Codonopsis Radix	MOL003896	7-Methoxy-2-methyl isoflavon	ADRB2	NO
Codonopsis Radix	MOL003896	7-Methoxy-2-methyl isoflavon	ADRA1D	NO
Codonopsis Radix	MOL003896	7-Methoxy-2-methyl isoflavon	SLC6A4	YES
Codonopsis Radix	MOL003896	7-Methoxy-2-methyl isoflavon	ESR2	YES
Codonopsis Radix	MOL003896	7-Methoxy-2-methyl isoflavon	DPP4	NO
Codonopsis Radix	MOL003896	7-Methoxy-2-methyl isoflavon	MAPK14	YES
Codonopsis Radix	MOL003896	7-Methoxy-2-methyl isoflavon	GSK3B	YES
Codonopsis Radix	MOL003896	7-Methoxy-2-methyl isoflavon	HSP90AA1	YES
Codonopsis Radix	MOL003896	7-Methoxy-2-methyl isoflavon	CDK2	NO
Codonopsis Radix	MOL003896	7-Methoxy-2-methyl isoflavon	LTA4H	NO
Codonopsis Radix	MOL003896	7-Methoxy-2-methyl isoflavon	MAOB	NO
Codonopsis Radix	MOL003896	7-Methoxy-2-methyl isoflavon	CHRNA7	NO
Codonopsis Radix	MOL003896	7-Methoxy-2-methyl isoflavon	CHEK1	NO
Codonopsis Radix	MOL003896	7-Methoxy-2-methyl isoflavon	PRKACA	NO
Codonopsis Radix	MOL003896	7-Methoxy-2-methyl isoflavon	IGHG1	NO
Codonopsis Radix	MOL003896	7-Methoxy-2-methyl isoflavon	PRSS1	NO

Codonopsis Radix	MOL003896	7-Methoxy-2-methyl isoflavon	CCNA2	NO
Codonopsis Radix	MOL003896	7-Methoxy-2-methyl isoflavon	NCOA1	NO
Codonopsis Radix	MOL003896	7-Methoxy-2-methyl isoflavon	PKIA	NO
Codonopsis Radix	MOL003896	7-Methoxy-2-methyl isoflavon	CALM3	NO
Codonopsis Radix	MOL003896	7-Methoxy-2-methyl isoflavon	CHRM5	NO
Codonopsis Radix	MOL003896	7-Methoxy-2-methyl isoflavon	OPRM1	NO
Codonopsis Radix	MOL003896	7-Methoxy-2-methyl isoflavon	NCOA2	NO
Codonopsis Radix	MOL003896	7-Methoxy-2-methyl isoflavon	NOS2	YES
Codonopsis Radix	MOL003896	7-Methoxy-2-methyl isoflavon	PTGS1	NO
Codonopsis Radix	MOL003896	7-Methoxy-2-methyl isoflavon	DRD1	NO
Codonopsis Radix	MOL003896	7-Methoxy-2-methyl isoflavon	CHRM3	NO
Codonopsis Radix	MOL003896	7-Methoxy-2-methyl isoflavon	F2	YES
Codonopsis Radix	MOL003896	7-Methoxy-2-methyl isoflavon	CHRM1	NO
Codonopsis Radix	MOL003896	7-Methoxy-2-methyl isoflavon	ESR1	YES
Codonopsis Radix	MOL003896	7-Methoxy-2-methyl isoflavon	AR	YES
Codonopsis Radix	MOL003896	7-Methoxy-2-methyl isoflavon	ADRB1	NO
Codonopsis Radix	MOL003896	7-Methoxy-2-methyl isoflavon	SCN5A	NO
Codonopsis Radix	MOL003896	7-Methoxy-2-methyl isoflavon	PPARG	NO
Codonopsis Radix	MOL003896	7-Methoxy-2-methyl isoflavon	PTGS2	YES
Codonopsis Radix	MOL003896	7-Methoxy-2-methyl isoflavon	RXRA	NO
Codonopsis Radix	MOL003896	7-Methoxy-2-methyl isoflavon	ACHE	NO
Codonopsis Radix	MOL003896	7-Methoxy-2-methyl isoflavon	PDE3A	NO
Codonopsis Radix	MOL003896	7-Methoxy-2-methyl isoflavon	ADRA1B	NO
Codonopsis Radix	MOL003896	7-Methoxy-2-methyl isoflavon	SLC6A3	NO
Codonopsis Radix	MOL003896	7-Methoxy-2-methyl isoflavon	ADRB2	NO
Codonopsis Radix	MOL003896	7-Methoxy-2-methyl isoflavon	ADRA1D	NO
Codonopsis Radix	MOL003896	7-Methoxy-2-methyl isoflavon	SLC6A4	YES
Codonopsis Radix	MOL003896	7-Methoxy-2-methyl isoflavon	ESR2	YES
Codonopsis Radix	MOL003896	7-Methoxy-2-methyl isoflavon	DPP4	NO
Codonopsis Radix	MOL003896	7-Methoxy-2-methyl isoflavon	MAPK14	YES
Codonopsis Radix	MOL003896	7-Methoxy-2-methyl isoflavon	GSK3B	YES
Codonopsis Radix	MOL003896	7-Methoxy-2-methyl isoflavon	HSP90AA1	YES
Codonopsis Radix	MOL003896	7-Methoxy-2-methyl isoflavon	CDK2	NO
Codonopsis Radix	MOL003896	7-Methoxy-2-methyl isoflavon	LTA4H	NO
Codonopsis Radix	MOL003896	7-Methoxy-2-methyl isoflavon	MAOB	NO
Codonopsis Radix	MOL003896	7-Methoxy-2-methyl isoflavon	CHRNA7	NO
Codonopsis Radix	MOL003896	7-Methoxy-2-methyl isoflavon	CHEK1	NO
Codonopsis Radix	MOL003896	7-Methoxy-2-methyl isoflavon	PRKACA	NO
Codonopsis Radix	MOL003896	7-Methoxy-2-methyl isoflavon	IGHG1	NO
Codonopsis Radix	MOL003896	7-Methoxy-2-methyl isoflavon	PRSS1	NO
Codonopsis Radix	MOL003896	7-Methoxy-2-methyl isoflavon	CCNA2	NO
Codonopsis Radix	MOL003896	7-Methoxy-2-methyl isoflavon	NCOA1	NO
Codonopsis Radix	MOL003896	7-Methoxy-2-methyl isoflavon	PKIA	NO
Codonopsis Radix	MOL003896	7-Methoxy-2-methyl isoflavon	CALM3	NO
Codonopsis Radix	MOL003896	7-Methoxy-2-methyl isoflavon	CHRM5	NO
Codonopsis Radix	MOL003896	7-Methoxy-2-methyl isoflavon	OPRM1	NO
Codonopsis Radix	MOL003896	7-Methoxy-2-methyl isoflavon	NCOA2	NO
licorice	MOL004838	8-(6-hydroxy-2-benzofuranyl)	NOS2	YES
licorice	MOL004838	8-(6-hydroxy-2-benzofuranyl)	ESR1	YES
licorice	MOL004838	8-(6-hydroxy-2-benzofuranyl)	PTGS2	YES
licorice	MOL004838	8-(6-hydroxy-2-benzofuranyl)	RXRA	NO
licorice	MOL004838	8-(6-hydroxy-2-benzofuranyl)	HSP90AA1	YES
licorice	MOL004993	8-prenylated eriodictyol	ESR1	YES
licorice	MOL004993	8-prenylated eriodictyol	SCN5A	NO

licorice	MOL004993	8-prenylated eriodictyol	F10	YES
licorice	MOL004993	8-prenylated eriodictyol	PTGS2	YES
licorice	MOL004993	8-prenylated eriodictyol	F7	NO
licorice	MOL004993	8-prenylated eriodictyol	HSP90AA1	YES
licorice	MOL004993	8-prenylated eriodictyol	NCOA1	NO
licorice	MOL004993	8-prenylated eriodictyol	CALM3	NO
Scutellariae Radix	MOL001689	acacetin	NOS2	YES
Scutellariae Radix	MOL001689	acacetin	PTGS1	NO
Scutellariae Radix	MOL001689	acacetin	AR	YES
Scutellariae Radix	MOL001689	acacetin	PTGS2	YES
Scutellariae Radix	MOL001689	acacetin	DPP4	NO
Scutellariae Radix	MOL001689	acacetin	HSP90AA1	YES
Scutellariae Radix	MOL001689	acacetin	CDK2	NO
Scutellariae Radix	MOL001689	acacetin	PRKACA	NO
Scutellariae Radix	MOL001689	acacetin	PRSS1	NO
Scutellariae Radix	MOL001689	acacetin	NCOA2	NO
Scutellariae Radix	MOL001689	acacetin	NCOA1	NO
Scutellariae Radix	MOL001689	acacetin	CALM3	NO
Scutellariae Radix	MOL001689	acacetin	CHEK1	NO
Scutellariae Radix	MOL001689	acacetin	ADRB2	NO
Scutellariae Radix	MOL001689	acacetin	PDE3A	NO
Scutellariae Radix	MOL001689	acacetin	RELA	YES
Scutellariae Radix	MOL001689	acacetin	BCL2	YES
Scutellariae Radix	MOL001689	acacetin	CDKN1A	NO
Scutellariae Radix	MOL001689	acacetin	BAX	NO
Scutellariae Radix	MOL001689	acacetin	CASP3	YES
Scutellariae Radix	MOL001689	acacetin	TP53	YES
Scutellariae Radix	MOL001689	acacetin	CASP8	NO
Scutellariae Radix	MOL001689	acacetin	FASN	YES
Scutellariae Radix	MOL001689	acacetin	FASLG	NO
Scutellariae Radix	MOL001689	acacetin	CYP19A1	YES
Pogostemon Cablin (	MOL005922	Acanthoside B	TOP2A	NO
Radix Bupleuri	MOL004609	Areapillin	NOS2	YES
Radix Bupleuri	MOL004609	Areapillin	F2	YES
Radix Bupleuri	MOL004609	Areapillin	AR	YES
Radix Bupleuri	MOL004609	Areapillin	SCN5A	NO
Radix Bupleuri	MOL004609	Areapillin	F10	YES
Radix Bupleuri	MOL004609	Areapillin	PTGS2	YES
Radix Bupleuri	MOL004609	Areapillin	F7	NO
Radix Bupleuri	MOL004609	Areapillin	PTPN1	YES
Radix Bupleuri	MOL004609	Areapillin	TOP2A	NO
Radix Bupleuri	MOL004609	Areapillin	ESR2	YES
Radix Bupleuri	MOL004609	Areapillin	DPP4	NO
Radix Bupleuri	MOL004609	Areapillin	HSP90AA1	YES
Radix Bupleuri	MOL004609	Areapillin	IGHG1	NO
Radix Bupleuri	MOL004609	Areapillin	PRSS1	NO
Radix Bupleuri	MOL004609	Areapillin	NCOA2	NO
Radix Bupleuri	MOL004609	Areapillin	NCOA1	NO
Radix Bupleuri	MOL004609	Areapillin	CALM3	NO
Scutellariae Radix	MOL002714	baicalein	PTGS1	NO
Scutellariae Radix	MOL002714	baicalein	AR	YES
Scutellariae Radix	MOL002714	baicalein	PTGS2	YES
Scutellariae Radix	MOL002714	baicalein	HSP90AA1	YES
Scutellariae Radix	MOL002714	baicalein	PRKACA	NO

Scutellariae Radix	MOL002714	baicalein	DPP4	NO
Scutellariae Radix	MOL002714	baicalein	PDE3A	NO
Scutellariae Radix	MOL002714	baicalein	PRSS1	NO
Scutellariae Radix	MOL002714	baicalein	NCOA2	NO
Scutellariae Radix	MOL002714	baicalein	NCOA1	NO
Scutellariae Radix	MOL002714	baicalein	CALM3	NO
Scutellariae Radix	MOL002714	baicalein	RELA	YES
Scutellariae Radix	MOL002714	baicalein	AKT1	YES
Scutellariae Radix	MOL002714	baicalein	METTL1	NO
Scutellariae Radix	MOL002714	baicalein	BCL2	YES
Scutellariae Radix	MOL002714	baicalein	FOS	NO
Scutellariae Radix	MOL002714	baicalein	BAX	NO
Scutellariae Radix	MOL002714	baicalein	MMP9	YES
Scutellariae Radix	MOL002714	baicalein	CASP3	YES
Scutellariae Radix	MOL002714	baicalein	TP53	YES
Scutellariae Radix	MOL002714	baicalein	HIF1A	NO
Scutellariae Radix	MOL002714	baicalein	FOSL1	NO
Scutellariae Radix	MOL002714	baicalein	FOSL2	NO
Scutellariae Radix	MOL002714	baicalein	CDK1	NO
Scutellariae Radix	MOL002714	baicalein	CCNB1	NO
Scutellariae Radix	MOL002714	baicalein	MPO	NO
Scutellariae Radix	MOL002714	baicalein	AHR	YES
Scutellariae Radix	MOL002714	baicalein	IGF2	YES
Scutellariae Radix	MOL002714	baicalein	CYCS	NO
Scutellariae Radix	MOL002714	baicalein	NFATC1	NO
Scutellariae Radix	MOL002714	baicalein	TDRD7	NO
Scutellariae Radix	MOL002714	baicalein	EGLN1	NO
Scutellariae Radix	MOL002714	baicalein	NOX5	NO
Scutellariae Radix	MOL002714	baicalein	FABP5	NO
Scutellariae Radix	MOL002714	baicalein	APOD	NO
Radix Bupleuri	MOL002776	Baicalin	F10	YES
Radix Bupleuri	MOL002776	Baicalin	PTPN1	YES
Scutellariae Radix	MOL000358	beta-sitosterol	PGR	NO
Scutellariae Radix	MOL000358	beta-sitosterol	NCOA2	NO
Scutellariae Radix	MOL000358	beta-sitosterol	PTGS1	NO
Scutellariae Radix	MOL000358	beta-sitosterol	PTGS2	YES
Scutellariae Radix	MOL000358	beta-sitosterol	HSP90AA1	YES
Scutellariae Radix	MOL000358	beta-sitosterol	KCNH2	NO
Scutellariae Radix	MOL000358	beta-sitosterol	PRKACA	NO
Scutellariae Radix	MOL000358	beta-sitosterol	DRD1	NO
Scutellariae Radix	MOL000358	beta-sitosterol	CHRM3	NO
Scutellariae Radix	MOL000358	beta-sitosterol	CHRM1	NO
Scutellariae Radix	MOL000358	beta-sitosterol	SCN5A	NO
Scutellariae Radix	MOL000358	beta-sitosterol	CHRM4	NO
Scutellariae Radix	MOL000358	beta-sitosterol	PDE3A	NO
Scutellariae Radix	MOL000358	beta-sitosterol	ADRA1A	NO
Scutellariae Radix	MOL000358	beta-sitosterol	CHRM2	NO
Scutellariae Radix	MOL000358	beta-sitosterol	ADRA1B	NO
Scutellariae Radix	MOL000358	beta-sitosterol	ADRB2	NO
Scutellariae Radix	MOL000358	beta-sitosterol	CHRNA2	NO
Scutellariae Radix	MOL000358	beta-sitosterol	SLC6A4	YES
Scutellariae Radix	MOL000358	beta-sitosterol	OPRM1	NO
Scutellariae Radix	MOL000358	beta-sitosterol	CHRNA7	NO
Scutellariae Radix	MOL000358	beta-sitosterol	BCL2	YES

Scutellariae Radix	MOL000358	beta-sitosterol	BAX	NO
Scutellariae Radix	MOL000358	beta-sitosterol	CASP9	YES
Scutellariae Radix	MOL000358	beta-sitosterol	JUN	YES
Scutellariae Radix	MOL000358	beta-sitosterol	CASP3	YES
Scutellariae Radix	MOL000358	beta-sitosterol	CASP8	NO
Scutellariae Radix	MOL000358	beta-sitosterol	PRKCA	NO
Scutellariae Radix	MOL000358	beta-sitosterol	PON1	NO
Scutellariae Radix	MOL000358	beta-sitosterol	MAP2	NO
Scutellariae Radix	MOL000358	beta-sitosterol	PGR	NO
Scutellariae Radix	MOL000358	beta-sitosterol	NCOA2	NO
Scutellariae Radix	MOL000358	beta-sitosterol	PTGS1	NO
Scutellariae Radix	MOL000358	beta-sitosterol	PTGS2	YES
Scutellariae Radix	MOL000358	beta-sitosterol	HSP90AA1	YES
Scutellariae Radix	MOL000358	beta-sitosterol	KCNH2	NO
Scutellariae Radix	MOL000358	beta-sitosterol	PRKACA	NO
Scutellariae Radix	MOL000358	beta-sitosterol	DRD1	NO
Scutellariae Radix	MOL000358	beta-sitosterol	CHRM3	NO
Scutellariae Radix	MOL000358	beta-sitosterol	CHRM1	NO
Scutellariae Radix	MOL000358	beta-sitosterol	SCN5A	NO
Scutellariae Radix	MOL000358	beta-sitosterol	CHRM4	NO
Scutellariae Radix	MOL000358	beta-sitosterol	PDE3A	NO
Scutellariae Radix	MOL000358	beta-sitosterol	ADRA1A	NO
Scutellariae Radix	MOL000358	beta-sitosterol	CHRM2	NO
Scutellariae Radix	MOL000358	beta-sitosterol	ADRA1B	NO
Scutellariae Radix	MOL000358	beta-sitosterol	ADRB2	NO
Scutellariae Radix	MOL000358	beta-sitosterol	CHRNA2	NO
Scutellariae Radix	MOL000358	beta-sitosterol	SLC6A4	YES
Scutellariae Radix	MOL000358	beta-sitosterol	OPRM1	NO
Scutellariae Radix	MOL000358	beta-sitosterol	CHRNA7	NO
Scutellariae Radix	MOL000358	beta-sitosterol	BCL2	YES
Scutellariae Radix	MOL000358	beta-sitosterol	BAX	NO
Scutellariae Radix	MOL000358	beta-sitosterol	CASP9	YES
Scutellariae Radix	MOL000358	beta-sitosterol	JUN	YES
Scutellariae Radix	MOL000358	beta-sitosterol	CASP3	YES
Scutellariae Radix	MOL000358	beta-sitosterol	CASP8	NO
Scutellariae Radix	MOL000358	beta-sitosterol	PRKCA	NO
Scutellariae Radix	MOL000358	beta-sitosterol	PON1	NO
Scutellariae Radix	MOL000358	beta-sitosterol	MAP2	NO
Scutellariae Radix	MOL001490	bis[(2S)-2-ethylhexyl] benze	SCN5A	NO
licorice	MOL000417	Calycosin	NOS2	YES
licorice	MOL000417	Calycosin	PTGS1	NO
licorice	MOL000417	Calycosin	ESR1	YES
licorice	MOL000417	Calycosin	AR	YES
licorice	MOL000417	Calycosin	PPARG	NO
licorice	MOL000417	Calycosin	PTGS2	YES
licorice	MOL000417	Calycosin	RXRA	NO
licorice	MOL000417	Calycosin	PDE3A	NO
licorice	MOL000417	Calycosin	ESR2	YES
licorice	MOL000417	Calycosin	DPP4	NO
licorice	MOL000417	Calycosin	MAPK14	YES
licorice	MOL000417	Calycosin	GSK3B	YES
licorice	MOL000417	Calycosin	HSP90AA1	YES
licorice	MOL000417	Calycosin	CDK2	NO
licorice	MOL000417	Calycosin	CHEK1	NO

licorice	MOL000417	Calycosin	PRKACA	NO
licorice	MOL000417	Calycosin	PRSS1	NO
licorice	MOL000417	Calycosin	CCNA2	NO
licorice	MOL000417	Calycosin	NCOA2	NO
licorice	MOL000417	Calycosin	CALM3	NO
licorice	MOL000417	Calycosin	ADRB2	NO
Scutellariae Radix	MOL002910	Carthamidin	PTGS1	NO
Scutellariae Radix	MOL002910	Carthamidin	PTGS2	YES
Scutellariae Radix	MOL002910	Carthamidin	HSP90AA1	YES
Scutellariae Radix	MOL002910	Carthamidin	PRKACA	NO
Coicis Semen	MOL000953	CLR	PGR	NO
Coicis Semen	MOL000953	CLR	NR3C2	NO
Coicis Semen	MOL000953	CLR	NCOA2	NO
Scutellariae Radix	MOL001458	coptisine	NOS2	YES
Scutellariae Radix	MOL001458	coptisine	PTGS1	NO
Scutellariae Radix	MOL001458	coptisine	KCNH2	NO
Scutellariae Radix	MOL001458	coptisine	ESR1	YES
Scutellariae Radix	MOL001458	coptisine	AR	YES
Scutellariae Radix	MOL001458	coptisine	SCN5A	NO
Scutellariae Radix	MOL001458	coptisine	PTGS2	YES
Scutellariae Radix	MOL001458	coptisine	PRSS1	NO
Radix Bupleuri	MOL013187	Cubebin	PTGS1	NO
Radix Bupleuri	MOL013187	Cubebin	F10	YES
Radix Bupleuri	MOL013187	Cubebin	PTGS2	YES
Radix Bupleuri	MOL013187	Cubebin	ADRB2	NO
Radix Bupleuri	MOL013187	Cubebin	HSP90AA1	YES
Radix Puerariae	MOL003629	Daidzein-4,7-diglucoside	TOP2A	NO
Codonopsis Radix	MOL008397	Daturilin	NR3C1	NO
licorice	MOL005020	dehydroglyasperins C	NOS2	YES
licorice	MOL005020	dehydroglyasperins C	ESR1	YES
licorice	MOL005020	dehydroglyasperins C	AR	YES
licorice	MOL005020	dehydroglyasperins C	SCN5A	NO
licorice	MOL005020	dehydroglyasperins C	PPARG	NO
licorice	MOL005020	dehydroglyasperins C	F10	YES
licorice	MOL005020	dehydroglyasperins C	PTGS2	YES
licorice	MOL005020	dehydroglyasperins C	ADRB2	NO
licorice	MOL005020	dehydroglyasperins C	ESR2	YES
licorice	MOL005020	dehydroglyasperins C	MAPK14	YES
licorice	MOL005020	dehydroglyasperins C	HSP90AA1	YES
licorice	MOL005020	dehydroglyasperins C	CDK2	NO
licorice	MOL005020	dehydroglyasperins C	CHEK1	NO
licorice	MOL005020	dehydroglyasperins C	PRSS1	NO
licorice	MOL005020	dehydroglyasperins C	CCNA2	NO
licorice	MOL005020	dehydroglyasperins C	NCOA2	NO
licorice	MOL005020	dehydroglyasperins C	CALM3	NO
licorice	MOL001792	DFV	PTGS1	NO
licorice	MOL001792	DFV	ESR1	YES
licorice	MOL001792	DFV	PTGS2	YES
licorice	MOL001792	DFV	RXRA	NO
licorice	MOL001792	DFV	ADRB2	NO
licorice	MOL001792	DFV	HSP90AA1	YES
licorice	MOL001792	DFV	PRKACA	NO
licorice	MOL001792	DFV	DPEP1	NO
licorice	MOL001792	DFV	MAOB	NO

licorice	MOL001792	DFV	SLC6A4	YES
licorice	MOL001792	DFV	PKIA	NO
Scutellariae Radix	MOL002913	Dihydrobaicalin_qt	PTGS1	NO
Scutellariae Radix	MOL002913	Dihydrobaicalin_qt	PTGS2	YES
Scutellariae Radix	MOL002913	Dihydrobaicalin_qt	HSP90AA1	YES
Scutellariae Radix	MOL002913	Dihydrobaicalin_qt	PRKACA	NO
Scutellariae Radix	MOL002937	DIHYDROOROXYLIN	PTGS1	NO
Scutellariae Radix	MOL002937	DIHYDROOROXYLIN	SCN5A	NO
Scutellariae Radix	MOL002937	DIHYDROOROXYLIN	PTGS2	YES
Scutellariae Radix	MOL002937	DIHYDROOROXYLIN	RXRA	NO
Scutellariae Radix	MOL002937	DIHYDROOROXYLIN	PDE3A	NO
Scutellariae Radix	MOL002937	DIHYDROOROXYLIN	ADRA1B	NO
Scutellariae Radix	MOL002937	DIHYDROOROXYLIN	ADRB2	NO
Scutellariae Radix	MOL002937	DIHYDROOROXYLIN	HSP90AA1	YES
Scutellariae Radix	MOL002937	DIHYDROOROXYLIN	PRKACA	NO
Scutellariae Radix	MOL002937	DIHYDROOROXYLIN	CALM3	NO
Scutellariae Radix	MOL002937	DIHYDROOROXYLIN	NCOA1	NO
Scutellariae Radix	MOL002879	Diop	SCN5A	NO
Scutellariae Radix	MOL002879	Diop	ADRB2	NO
Scutellariae Radix	MOL002879	Diop	CHRM3	NO
Scutellariae Radix	MOL002879	Diop	SCN5A	NO
Scutellariae Radix	MOL002879	Diop	ADRB2	NO
Scutellariae Radix	MOL002879	Diop	CHRM3	NO
Scutellariae Radix	MOL002879	Diop	SCN5A	NO
Scutellariae Radix	MOL002879	Diop	ADRB2	NO
Scutellariae Radix	MOL002879	Diop	CHRM3	NO
Scutellariae Radix	MOL000073	ent-Epicatechin	PTGS1	NO
Scutellariae Radix	MOL000073	ent-Epicatechin	ESR1	YES
Scutellariae Radix	MOL000073	ent-Epicatechin	PTGS2	YES
Scutellariae Radix	MOL000073	ent-Epicatechin	HSP90AA1	YES
Scutellariae Radix	MOL000073	ent-Epicatechin	DPEP1	NO
Scutellariae Radix	MOL000073	ent-Epicatechin	PRKACA	NO
Scutellariae Radix	MOL002897	epiberberine	NOS2	YES
Scutellariae Radix	MOL002897	epiberberine	KCNH2	NO
Scutellariae Radix	MOL002897	epiberberine	ESR1	YES
Scutellariae Radix	MOL002897	epiberberine	AR	YES
Scutellariae Radix	MOL002897	epiberberine	PTGS2	YES
Scutellariae Radix	MOL002897	epiberberine	RXRA	NO
Scutellariae Radix	MOL002897	epiberberine	PRKACA	NO
Scutellariae Radix	MOL002897	epiberberine	PRSS1	NO
Scutellariae Radix	MOL002897	epiberberine	NCOA2	NO
Scutellariae Radix	MOL002897	epiberberine	PDE10A	NO
Scutellariae Radix	MOL002914	Eriodyctiol (flavanone)	PTGS1	NO
Scutellariae Radix	MOL002914	Eriodyctiol (flavanone)	PTGS2	YES
Scutellariae Radix	MOL002914	Eriodyctiol (flavanone)	HSP90AA1	YES
Scutellariae Radix	MOL002914	Eriodyctiol (flavanone)	PRKACA	NO
Scutellariae Radix	MOL002914	Eriodyctiol (flavanone)	NCOA2	NO
Scutellariae Radix	MOL002914	Eriodyctiol (flavanone)	PYGM	NO
Scutellariae Radix	MOL002914	Eriodyctiol (flavanone)	CALM3	NO
licorice	MOL004806	euchrenone	NOS2	YES
licorice	MOL004806	euchrenone	KCNH2	NO
licorice	MOL004806	euchrenone	ESR1	YES
licorice	MOL004806	euchrenone	SCN5A	NO
licorice	MOL004806	euchrenone	F10	YES

licorice	MOL004806	euchrenone	PTGS2	YES
licorice	MOL004806	euchrenone	ESR2	YES
licorice	MOL004806	euchrenone	BACE1	NO
licorice	MOL004806	euchrenone	CALM3	NO
licorice	MOL004915	Eurycarpin A	NOS2	YES
licorice	MOL004915	Eurycarpin A	F2	YES
licorice	MOL004915	Eurycarpin A	ESR1	YES
licorice	MOL004915	Eurycarpin A	AR	YES
licorice	MOL004915	Eurycarpin A	SCN5A	NO
licorice	MOL004915	Eurycarpin A	PPARG	NO
licorice	MOL004915	Eurycarpin A	F10	YES
licorice	MOL004915	Eurycarpin A	PTGS2	YES
licorice	MOL004915	Eurycarpin A	ESR2	YES
licorice	MOL004915	Eurycarpin A	DPP4	NO
licorice	MOL004915	Eurycarpin A	MAPK14	YES
licorice	MOL004915	Eurycarpin A	GSK3B	YES
licorice	MOL004915	Eurycarpin A	HSP90AA1	YES
licorice	MOL004915	Eurycarpin A	CDK2	NO
licorice	MOL004915	Eurycarpin A	CHEK1	NO
licorice	MOL004915	Eurycarpin A	PRSS1	NO
licorice	MOL004915	Eurycarpin A	CCNA2	NO
licorice	MOL004915	Eurycarpin A	CALM3	NO
Radix Puerariae	MOL000392	formononetin	NOS2	YES
Radix Puerariae	MOL000392	formononetin	PTGS1	NO
Radix Puerariae	MOL000392	formononetin	CHRM1	NO
Radix Puerariae	MOL000392	formononetin	ESR1	YES
Radix Puerariae	MOL000392	formononetin	AR	YES
Radix Puerariae	MOL000392	formononetin	PPARG	NO
Radix Puerariae	MOL000392	formononetin	PTGS2	YES
Radix Puerariae	MOL000392	formononetin	RXRA	NO
Radix Puerariae	MOL000392	formononetin	PDE3A	NO
Radix Puerariae	MOL000392	formononetin	ADRA1A	NO
Radix Puerariae	MOL000392	formononetin	SLC6A3	NO
Radix Puerariae	MOL000392	formononetin	ADRB2	NO
Radix Puerariae	MOL000392	formononetin	SLC6A4	YES
Radix Puerariae	MOL000392	formononetin	ESR2	YES
Radix Puerariae	MOL000392	formononetin	DPP4	NO
Radix Puerariae	MOL000392	formononetin	MAPK14	YES
Radix Puerariae	MOL000392	formononetin	GSK3B	YES
Radix Puerariae	MOL000392	formononetin	HSP90AA1	YES
Radix Puerariae	MOL000392	formononetin	CDK2	NO
Radix Puerariae	MOL000392	formononetin	MAOB	NO
Radix Puerariae	MOL000392	formononetin	CHEK1	NO
Radix Puerariae	MOL000392	formononetin	PRKACA	NO
Radix Puerariae	MOL000392	formononetin	PRSS1	NO
Radix Puerariae	MOL000392	formononetin	CCNA2	NO
Radix Puerariae	MOL000392	formononetin	CALM3	NO
Radix Puerariae	MOL000392	formononetin	PKIA	NO
Radix Puerariae	MOL000392	formononetin	F2	YES
Radix Puerariae	MOL000392	formononetin	ACHE	NO
Radix Puerariae	MOL000392	formononetin	DPEP1	NO
Radix Puerariae	MOL000392	formononetin	JUN	YES
Radix Puerariae	MOL000392	formononetin	PPARG	NO
Radix Puerariae	MOL000392	formononetin	IL4	YES

Radix Puerariae	MOL000392	formononetin	ATP5F1B	NO
Radix Puerariae	MOL000392	formononetin	HSD3B2	NO
Radix Puerariae	MOL000392	formononetin	HSD3B1	NO
Radix Puerariae	MOL000392	formononetin	NOS2	YES
Radix Puerariae	MOL000392	formononetin	PTGS1	NO
Radix Puerariae	MOL000392	formononetin	CHRM1	NO
Radix Puerariae	MOL000392	formononetin	ESR1	YES
Radix Puerariae	MOL000392	formononetin	AR	YES
Radix Puerariae	MOL000392	formononetin	PPARG	NO
Radix Puerariae	MOL000392	formononetin	PTGS2	YES
Radix Puerariae	MOL000392	formononetin	RXRA	NO
Radix Puerariae	MOL000392	formononetin	PDE3A	NO
Radix Puerariae	MOL000392	formononetin	ADRA1A	NO
Radix Puerariae	MOL000392	formononetin	SLC6A3	NO
Radix Puerariae	MOL000392	formononetin	ADRB2	NO
Radix Puerariae	MOL000392	formononetin	SLC6A4	YES
Radix Puerariae	MOL000392	formononetin	ESR2	YES
Radix Puerariae	MOL000392	formononetin	DPP4	NO
Radix Puerariae	MOL000392	formononetin	MAPK14	YES
Radix Puerariae	MOL000392	formononetin	GSK3B	YES
Radix Puerariae	MOL000392	formononetin	HSP90AA1	YES
Radix Puerariae	MOL000392	formononetin	CDK2	NO
Radix Puerariae	MOL000392	formononetin	MAOB	NO
Radix Puerariae	MOL000392	formononetin	CHEK1	NO
Radix Puerariae	MOL000392	formononetin	PRKACA	NO
Radix Puerariae	MOL000392	formononetin	PRSS1	NO
Radix Puerariae	MOL000392	formononetin	CCNA2	NO
Radix Puerariae	MOL000392	formononetin	CALM3	NO
Radix Puerariae	MOL000392	formononetin	PKIA	NO
Radix Puerariae	MOL000392	formononetin	F2	YES
Radix Puerariae	MOL000392	formononetin	ACHE	NO
Radix Puerariae	MOL000392	formononetin	DPEP1	NO
Radix Puerariae	MOL000392	formononetin	JUN	YES
Radix Puerariae	MOL000392	formononetin	PPARG	NO
Radix Puerariae	MOL000392	formononetin	IL4	YES
Radix Puerariae	MOL000392	formononetin	ATP5F1B	NO
Radix Puerariae	MOL000392	formononetin	HSD3B2	NO
Radix Puerariae	MOL000392	formononetin	HSD3B1	NO
Codonopsis Radix	MOL005321	Frutinone A	PTGS1	NO
Codonopsis Radix	MOL005321	Frutinone A	F2	YES
Codonopsis Radix	MOL005321	Frutinone A	AR	YES
Codonopsis Radix	MOL005321	Frutinone A	SCN5A	NO
Codonopsis Radix	MOL005321	Frutinone A	PPARG	NO
Codonopsis Radix	MOL005321	Frutinone A	PTGS2	YES
Codonopsis Radix	MOL005321	Frutinone A	RXRA	NO
Codonopsis Radix	MOL005321	Frutinone A	PDE3A	NO
Codonopsis Radix	MOL005321	Frutinone A	ADRB2	NO
Codonopsis Radix	MOL005321	Frutinone A	DPP4	NO
Codonopsis Radix	MOL005321	Frutinone A	HSP90AA1	YES
Codonopsis Radix	MOL005321	Frutinone A	CHRNA7	NO
Codonopsis Radix	MOL005321	Frutinone A	PRKACA	NO
Codonopsis Radix	MOL005321	Frutinone A	ACHE	NO
licorice	MOL004996	gadelaidic acid	NCOA2	NO
licorice	MOL004856	Gancaonin A	NOS2	YES

licorice	MOL004856	Gancaonin A	F2	YES
licorice	MOL004856	Gancaonin A	ESR1	YES
licorice	MOL004856	Gancaonin A	AR	YES
licorice	MOL004856	Gancaonin A	SCN5A	NO
licorice	MOL004856	Gancaonin A	PPARG	NO
licorice	MOL004856	Gancaonin A	F10	YES
licorice	MOL004856	Gancaonin A	PTGS2	YES
licorice	MOL004856	Gancaonin A	ACHE	NO
licorice	MOL004856	Gancaonin A	TOP2A	NO
licorice	MOL004856	Gancaonin A	ESR2	YES
licorice	MOL004856	Gancaonin A	DPP4	NO
licorice	MOL004856	Gancaonin A	GSK3B	YES
licorice	MOL004856	Gancaonin A	HSP90AA1	YES
licorice	MOL004856	Gancaonin A	CHEK1	NO
licorice	MOL004856	Gancaonin A	PRSS1	NO
licorice	MOL004856	Gancaonin A	CCNA2	NO
licorice	MOL004856	Gancaonin A	NCOA2	NO
licorice	MOL004856	Gancaonin A	CALM3	NO
licorice	MOL004857	Gancaonin B	NOS2	YES
licorice	MOL004857	Gancaonin B	F2	YES
licorice	MOL004857	Gancaonin B	ESR1	YES
licorice	MOL004857	Gancaonin B	AR	YES
licorice	MOL004857	Gancaonin B	PPARG	NO
licorice	MOL004857	Gancaonin B	F10	YES
licorice	MOL004857	Gancaonin B	PTGS2	YES
licorice	MOL004857	Gancaonin B	F7	NO
licorice	MOL004857	Gancaonin B	KDR	YES
licorice	MOL004857	Gancaonin B	ADRA1B	NO
licorice	MOL004857	Gancaonin B	ADRB2	NO
licorice	MOL004857	Gancaonin B	TOP2A	NO
licorice	MOL004857	Gancaonin B	ESR2	YES
licorice	MOL004857	Gancaonin B	DPP4	NO
licorice	MOL004857	Gancaonin B	GSK3B	YES
licorice	MOL004857	Gancaonin B	HSP90AA1	YES
licorice	MOL004857	Gancaonin B	CHEK1	NO
licorice	MOL004857	Gancaonin B	PRSS1	NO
licorice	MOL004857	Gancaonin B	CCNA2	NO
licorice	MOL004857	Gancaonin B	NCOA2	NO
licorice	MOL004857	Gancaonin B	CALM3	NO
licorice	MOL005000	Gancaonin G	NOS2	YES
licorice	MOL005000	Gancaonin G	F2	YES
licorice	MOL005000	Gancaonin G	ESR1	YES
licorice	MOL005000	Gancaonin G	AR	YES
licorice	MOL005000	Gancaonin G	PPARG	NO
licorice	MOL005000	Gancaonin G	F10	YES
licorice	MOL005000	Gancaonin G	PTGS2	YES
licorice	MOL005000	Gancaonin G	TOP2A	NO
licorice	MOL005000	Gancaonin G	ESR2	YES
licorice	MOL005000	Gancaonin G	DPP4	NO
licorice	MOL005000	Gancaonin G	MAPK14	YES
licorice	MOL005000	Gancaonin G	GSK3B	YES
licorice	MOL005000	Gancaonin G	HSP90AA1	YES
licorice	MOL005000	Gancaonin G	CHEK1	NO
licorice	MOL005000	Gancaonin G	PRSS1	NO

licorice	MOL005000	Gancaonin G	CCNA2	NO
licorice	MOL005000	Gancaonin G	NCOA2	NO
licorice	MOL005000	Gancaonin G	CALM3	NO
licorice	MOL005001	Gancaonin H	ESR1	YES
licorice	MOL005001	Gancaonin H	AR	YES
licorice	MOL005001	Gancaonin H	F10	YES
licorice	MOL005001	Gancaonin H	PTGS2	YES
licorice	MOL005001	Gancaonin H	KDR	YES
licorice	MOL005001	Gancaonin H	TOP2A	NO
licorice	MOL005001	Gancaonin H	HSP90AA1	YES
licorice	MOL005001	Gancaonin H	PRSS1	NO
licorice	MOL005001	Gancaonin H	CCNA2	NO
licorice	MOL005001	Gancaonin H	NCOA2	NO
licorice	MOL005001	Gancaonin H	CALM3	NO
Pogostemon Cablin (	MOL005573	Genkwanin	NOS2	YES
Pogostemon Cablin (	MOL005573	Genkwanin	PTGS1	NO
Pogostemon Cablin (	MOL005573	Genkwanin	AR	YES
Pogostemon Cablin (	MOL005573	Genkwanin	PTGS2	YES
Pogostemon Cablin (	MOL005573	Genkwanin	RXRA	NO
Pogostemon Cablin (	MOL005573	Genkwanin	ESR2	YES
Pogostemon Cablin (	MOL005573	Genkwanin	DPP4	NO
Pogostemon Cablin (	MOL005573	Genkwanin	HSP90AA1	YES
Pogostemon Cablin (	MOL005573	Genkwanin	PRKACA	NO
Pogostemon Cablin (	MOL005573	Genkwanin	PRSS1	NO
Pogostemon Cablin (	MOL005573	Genkwanin	NCOA2	NO
Pogostemon Cablin (	MOL005573	Genkwanin	NCOA1	NO
Pogostemon Cablin (	MOL005573	Genkwanin	CALM3	NO
Pogostemon Cablin (	MOL005573	Genkwanin	CHEK1	NO
licorice	MOL004910	Glabranin	NOS2	YES
licorice	MOL004910	Glabranin	PTGS1	NO
licorice	MOL004910	Glabranin	ESR1	YES
licorice	MOL004910	Glabranin	SCN5A	NO
licorice	MOL004910	Glabranin	F10	YES
licorice	MOL004910	Glabranin	PTGS2	YES
licorice	MOL004910	Glabranin	PDE3A	NO
licorice	MOL004910	Glabranin	HSP90AA1	YES
licorice	MOL004910	Glabranin	PRKACA	NO
licorice	MOL004910	Glabranin	CALM3	NO
licorice	MOL004911	Glabrene	NOS2	YES
licorice	MOL004911	Glabrene	PTGS1	NO
licorice	MOL004911	Glabrene	ESR1	YES
licorice	MOL004911	Glabrene	AR	YES
licorice	MOL004911	Glabrene	SCN5A	NO
licorice	MOL004911	Glabrene	PPARG	NO
licorice	MOL004911	Glabrene	F10	YES
licorice	MOL004911	Glabrene	PTGS2	YES
licorice	MOL004911	Glabrene	RXRA	NO
licorice	MOL004911	Glabrene	ADRB2	NO
licorice	MOL004911	Glabrene	ESR2	YES
licorice	MOL004911	Glabrene	MAPK14	YES
licorice	MOL004911	Glabrene	GSK3B	YES
licorice	MOL004911	Glabrene	HSP90AA1	YES
licorice	MOL004911	Glabrene	CDK2	NO
licorice	MOL004911	Glabrene	PRSS1	NO

licorice	MOL004911	Glabrene	NCOA2	NO
licorice	MOL004911	Glabrene	CALM3	NO
licorice	MOL004908	Glabridin	NOS2	YES
licorice	MOL004908	Glabridin	CHRM1	NO
licorice	MOL004908	Glabridin	ESR1	YES
licorice	MOL004908	Glabridin	AR	YES
licorice	MOL004908	Glabridin	SCN5A	NO
licorice	MOL004908	Glabridin	PPARG	NO
licorice	MOL004908	Glabridin	PTGS2	YES
licorice	MOL004908	Glabridin	RXRA	NO
licorice	MOL004908	Glabridin	ACHE	NO
licorice	MOL004908	Glabridin	ADRA1B	NO
licorice	MOL004908	Glabridin	ADRB2	NO
licorice	MOL004908	Glabridin	ESR2	YES
licorice	MOL004908	Glabridin	MAPK14	YES
licorice	MOL004908	Glabridin	GSK3B	YES
licorice	MOL004908	Glabridin	CDK2	NO
licorice	MOL004908	Glabridin	CHEK1	NO
licorice	MOL004908	Glabridin	PRKACA	NO
licorice	MOL004908	Glabridin	METTL1	NO
licorice	MOL004908	Glabridin	IGHG1	NO
licorice	MOL004908	Glabridin	PRSS1	NO
licorice	MOL004908	Glabridin	CCNA2	NO
licorice	MOL004908	Glabridin	NCOA2	NO
licorice	MOL004908	Glabridin	NCOA1	NO
licorice	MOL004908	Glabridin	CALM3	NO
licorice	MOL004912	Glabrone	NOS2	YES
licorice	MOL004912	Glabrone	PTGS1	NO
licorice	MOL004912	Glabrone	F2	YES
licorice	MOL004912	Glabrone	ESR1	YES
licorice	MOL004912	Glabrone	AR	YES
licorice	MOL004912	Glabrone	SCN5A	NO
licorice	MOL004912	Glabrone	PPARG	NO
licorice	MOL004912	Glabrone	F10	YES
licorice	MOL004912	Glabrone	PTGS2	YES
licorice	MOL004912	Glabrone	RXRA	NO
licorice	MOL004912	Glabrone	ACHE	NO
licorice	MOL004912	Glabrone	ESR2	YES
licorice	MOL004912	Glabrone	DPP4	NO
licorice	MOL004912	Glabrone	MAPK14	YES
licorice	MOL004912	Glabrone	GSK3B	YES
licorice	MOL004912	Glabrone	CDK2	NO
licorice	MOL004912	Glabrone	CHEK1	NO
licorice	MOL004912	Glabrone	PRSS1	NO
licorice	MOL004912	Glabrone	CCNA2	NO
licorice	MOL004912	Glabrone	CALM3	NO
licorice	MOL004828	Glepidotin A	NOS2	YES
licorice	MOL004828	Glepidotin A	PTGS1	NO
licorice	MOL004828	Glepidotin A	F2	YES
licorice	MOL004828	Glepidotin A	ESR1	YES
licorice	MOL004828	Glepidotin A	AR	YES
licorice	MOL004828	Glepidotin A	SCN5A	NO
licorice	MOL004828	Glepidotin A	PPARG	NO
licorice	MOL004828	Glepidotin A	F10	YES

licorice	MOL004828	Glepidotin A	PTGS2	YES
licorice	MOL004828	Glepidotin A	F7	NO
licorice	MOL004828	Glepidotin A	KDR	YES
licorice	MOL004828	Glepidotin A	RXRA	NO
licorice	MOL004828	Glepidotin A	PDE3A	NO
licorice	MOL004828	Glepidotin A	TOP2A	NO
licorice	MOL004828	Glepidotin A	DPP4	NO
licorice	MOL004828	Glepidotin A	MAPK14	YES
licorice	MOL004828	Glepidotin A	GSK3B	YES
licorice	MOL004828	Glepidotin A	HSP90AA1	YES
licorice	MOL004828	Glepidotin A	CDK2	NO
licorice	MOL004828	Glepidotin A	CHEK1	NO
licorice	MOL004828	Glepidotin A	IGHG1	NO
licorice	MOL004828	Glepidotin A	PRSS1	NO
licorice	MOL004828	Glepidotin A	CCNA2	NO
licorice	MOL004828	Glepidotin A	CALM3	NO
licorice	MOL004829	Glepidotin B	PTGS1	NO
licorice	MOL004829	Glepidotin B	ESR1	YES
licorice	MOL004829	Glepidotin B	SCN5A	NO
licorice	MOL004829	Glepidotin B	F10	YES
licorice	MOL004829	Glepidotin B	PTGS2	YES
licorice	MOL004829	Glepidotin B	F7	NO
licorice	MOL004829	Glepidotin B	RXRA	NO
licorice	MOL004829	Glepidotin B	PDE3A	NO
licorice	MOL004829	Glepidotin B	ADRA1B	NO
licorice	MOL004829	Glepidotin B	TOP2A	NO
licorice	MOL004829	Glepidotin B	HSP90AA1	YES
licorice	MOL004829	Glepidotin B	IGHG1	NO
licorice	MOL004829	Glepidotin B	NCOA1	NO
licorice	MOL004829	Glepidotin B	CALM3	NO
licorice	MOL004808	glyasperin B	NOS2	YES
licorice	MOL004808	glyasperin B	F2	YES
licorice	MOL004808	glyasperin B	ESR1	YES
licorice	MOL004808	glyasperin B	AR	YES
licorice	MOL004808	glyasperin B	PPARG	NO
licorice	MOL004808	glyasperin B	F10	YES
licorice	MOL004808	glyasperin B	PTGS2	YES
licorice	MOL004808	glyasperin B	F7	NO
licorice	MOL004808	glyasperin B	KDR	YES
licorice	MOL004808	glyasperin B	ACHE	NO
licorice	MOL004808	glyasperin B	TOP2A	NO
licorice	MOL004808	glyasperin B	ESR2	YES
licorice	MOL004808	glyasperin B	DPP4	NO
licorice	MOL004808	glyasperin B	GSK3B	YES
licorice	MOL004808	glyasperin B	HSP90AA1	YES
licorice	MOL004808	glyasperin B	CDK2	NO
licorice	MOL004808	glyasperin B	PRSS1	NO
licorice	MOL004808	glyasperin B	CCNA2	NO
licorice	MOL004808	glyasperin B	NCOA2	NO
licorice	MOL004808	glyasperin B	CALM3	NO
licorice	MOL004811	Glyasperin C	NOS2	YES
licorice	MOL004811	Glyasperin C	F2	YES
licorice	MOL004811	Glyasperin C	KCNH2	NO
licorice	MOL004811	Glyasperin C	ESR1	YES

licorice	MOL004811	Glyasperin C	AR	YES
licorice	MOL004811	Glyasperin C	SCN5A	NO
licorice	MOL004811	Glyasperin C	PPARG	NO
licorice	MOL004811	Glyasperin C	F10	YES
licorice	MOL004811	Glyasperin C	PTGS2	YES
licorice	MOL004811	Glyasperin C	RXRA	NO
licorice	MOL004811	Glyasperin C	ACHE	NO
licorice	MOL004811	Glyasperin C	TOP2A	NO
licorice	MOL004811	Glyasperin C	ESR2	YES
licorice	MOL004811	Glyasperin C	DPP4	NO
licorice	MOL004811	Glyasperin C	MAPK14	YES
licorice	MOL004811	Glyasperin C	GSK3B	YES
licorice	MOL004811	Glyasperin C	HSP90AA1	YES
licorice	MOL004811	Glyasperin C	CDK2	NO
licorice	MOL004811	Glyasperin C	CHEK1	NO
licorice	MOL004811	Glyasperin C	PRSS1	NO
licorice	MOL004811	Glyasperin C	CCNA2	NO
licorice	MOL004811	Glyasperin C	NCOA2	NO
licorice	MOL004811	Glyasperin C	CALM3	NO
licorice	MOL004810	glyasperin F	NOS2	YES
licorice	MOL004810	glyasperin F	PTGS1	NO
licorice	MOL004810	glyasperin F	ESR1	YES
licorice	MOL004810	glyasperin F	AR	YES
licorice	MOL004810	glyasperin F	SCN5A	NO
licorice	MOL004810	glyasperin F	PPARG	NO
licorice	MOL004810	glyasperin F	F10	YES
licorice	MOL004810	glyasperin F	PTGS2	YES
licorice	MOL004810	glyasperin F	TOP2A	NO
licorice	MOL004810	glyasperin F	ESR2	YES
licorice	MOL004810	glyasperin F	MAPK14	YES
licorice	MOL004810	glyasperin F	GSK3B	YES
licorice	MOL004810	glyasperin F	HSP90AA1	YES
licorice	MOL004810	glyasperin F	CDK2	NO
licorice	MOL004810	glyasperin F	PRSS1	NO
licorice	MOL004810	glyasperin F	CCNA2	NO
licorice	MOL004810	glyasperin F	CALM3	NO
licorice	MOL005007	Glyasperins M	NOS2	YES
licorice	MOL005007	Glyasperins M	PTGS1	NO
licorice	MOL005007	Glyasperins M	KCNH2	NO
licorice	MOL005007	Glyasperins M	ESR1	YES
licorice	MOL005007	Glyasperins M	AR	YES
licorice	MOL005007	Glyasperins M	SCN5A	NO
licorice	MOL005007	Glyasperins M	PPARG	NO
licorice	MOL005007	Glyasperins M	F10	YES
licorice	MOL005007	Glyasperins M	PTGS2	YES
licorice	MOL005007	Glyasperins M	F7	NO
licorice	MOL005007	Glyasperins M	KDR	YES
licorice	MOL005007	Glyasperins M	ACHE	NO
licorice	MOL005007	Glyasperins M	TOP2A	NO
licorice	MOL005007	Glyasperins M	ESR2	YES
licorice	MOL005007	Glyasperins M	PPARD	NO
licorice	MOL005007	Glyasperins M	GSK3B	YES
licorice	MOL005007	Glyasperins M	HSP90AA1	YES
licorice	MOL005007	Glyasperins M	CDK2	NO

licorice	MOL005007	Glyasperins M	PRKACA	NO
licorice	MOL005007	Glyasperins M	PRSS1	NO
licorice	MOL005007	Glyasperins M	CCNA2	NO
licorice	MOL005007	Glyasperins M	NCOA2	NO
licorice	MOL005007	Glyasperins M	NCOA1	NO
licorice	MOL005007	Glyasperins M	CALM3	NO
Codonopsis Radix	MOL008400	glycitein	PTGS1	NO
Codonopsis Radix	MOL008400	glycitein	ESR1	YES
Codonopsis Radix	MOL008400	glycitein	AR	YES
Codonopsis Radix	MOL008400	glycitein	PPARG	NO
Codonopsis Radix	MOL008400	glycitein	PTGS2	YES
Codonopsis Radix	MOL008400	glycitein	RXRA	NO
Codonopsis Radix	MOL008400	glycitein	PDE3A	NO
Codonopsis Radix	MOL008400	glycitein	ESR2	YES
Codonopsis Radix	MOL008400	glycitein	MAPK14	YES
Codonopsis Radix	MOL008400	glycitein	GSK3B	YES
Codonopsis Radix	MOL008400	glycitein	HSP90AA1	YES
Codonopsis Radix	MOL008400	glycitein	CDK2	NO
Codonopsis Radix	MOL008400	glycitein	CHEK1	NO
Codonopsis Radix	MOL008400	glycitein	PRSS1	NO
Codonopsis Radix	MOL008400	glycitein	CCNA2	NO
Codonopsis Radix	MOL008400	glycitein	CALM3	NO
Codonopsis Radix	MOL008400	glycitein	PRKACA	NO
Codonopsis Radix	MOL008400	glycitein	NCOA1	NO
Codonopsis Radix	MOL008400	glycitein	NOS2	YES
Codonopsis Radix	MOL008400	glycitein	APP	NO
Codonopsis Radix	MOL008400	glycitein	MMP13	NO
Codonopsis Radix	MOL008400	glycitein	MMP8	NO
licorice	MOL004879	Glycyrin	NOS2	YES
licorice	MOL004879	Glycyrin	F2	YES
licorice	MOL004879	Glycyrin	KCNH2	NO
licorice	MOL004879	Glycyrin	ESR1	YES
licorice	MOL004879	Glycyrin	AR	YES
licorice	MOL004879	Glycyrin	PPARG	NO
licorice	MOL004879	Glycyrin	F10	YES
licorice	MOL004879	Glycyrin	PTGS2	YES
licorice	MOL004879	Glycyrin	KDR	YES
licorice	MOL004879	Glycyrin	TOP2A	NO
licorice	MOL004879	Glycyrin	ESR2	YES
licorice	MOL004879	Glycyrin	DPP4	NO
licorice	MOL004879	Glycyrin	CHEK1	NO
licorice	MOL004879	Glycyrin	PRSS1	NO
licorice	MOL004879	Glycyrin	NCOA2	NO
licorice	MOL004879	Glycyrin	CALM3	NO
licorice	MOL002311	Glycyrol	NOS2	YES
licorice	MOL002311	Glycyrol	ESR1	YES
licorice	MOL002311	Glycyrol	PPARG	NO
licorice	MOL002311	Glycyrol	PTGS2	YES
licorice	MOL002311	Glycyrol	KDR	YES
licorice	MOL002311	Glycyrol	MAPK14	YES
licorice	MOL002311	Glycyrol	GSK3B	YES
licorice	MOL002311	Glycyrol	CHEK1	NO
licorice	MOL002311	Glycyrol	CCNA2	NO
licorice	MOL002311	Glycyrol	F2	YES

licorice	MOL005008	Glycyrrhiza flavonol A	NOS2	YES
licorice	MOL005008	Glycyrrhiza flavonol A	ESR1	YES
licorice	MOL005008	Glycyrrhiza flavonol A	AR	YES
licorice	MOL005008	Glycyrrhiza flavonol A	F10	YES
licorice	MOL005008	Glycyrrhiza flavonol A	PTGS2	YES
licorice	MOL005008	Glycyrrhiza flavonol A	F7	NO
licorice	MOL005008	Glycyrrhiza flavonol A	ACHE	NO
licorice	MOL005008	Glycyrrhiza flavonol A	TOP2A	NO
licorice	MOL005008	Glycyrrhiza flavonol A	ESR2	YES
licorice	MOL005008	Glycyrrhiza flavonol A	DPP4	NO
licorice	MOL005008	Glycyrrhiza flavonol A	GSK3B	YES
licorice	MOL005008	Glycyrrhiza flavonol A	HSP90AA1	YES
licorice	MOL005008	Glycyrrhiza flavonol A	CDK2	NO
licorice	MOL005008	Glycyrrhiza flavonol A	PRSS1	NO
licorice	MOL005008	Glycyrrhiza flavonol A	CCNA2	NO
licorice	MOL005008	Glycyrrhiza flavonol A	CALM3	NO
licorice	MOL004835	Glypallichalcone	NOS2	YES
licorice	MOL004835	Glypallichalcone	PTGS1	NO
licorice	MOL004835	Glypallichalcone	CHRM1	NO
licorice	MOL004835	Glypallichalcone	ESR1	YES
licorice	MOL004835	Glypallichalcone	AR	YES
licorice	MOL004835	Glypallichalcone	SCN5A	NO
licorice	MOL004835	Glypallichalcone	PPARG	NO
licorice	MOL004835	Glypallichalcone	PTGS2	YES
licorice	MOL004835	Glypallichalcone	CA2	NO
licorice	MOL004835	Glypallichalcone	PDE3A	NO
licorice	MOL004835	Glypallichalcone	ADRA1B	NO
licorice	MOL004835	Glypallichalcone	SLC6A3	NO
licorice	MOL004835	Glypallichalcone	ADRB2	NO
licorice	MOL004835	Glypallichalcone	SLC6A4	YES
licorice	MOL004835	Glypallichalcone	ESR2	YES
licorice	MOL004835	Glypallichalcone	MAPK14	YES
licorice	MOL004835	Glypallichalcone	GSK3B	YES
licorice	MOL004835	Glypallichalcone	HSP90AA1	YES
licorice	MOL004835	Glypallichalcone	CDK2	NO
licorice	MOL004835	Glypallichalcone	LTA4H	NO
licorice	MOL004835	Glypallichalcone	MAOB	NO
licorice	MOL004835	Glypallichalcone	CHEK1	NO
licorice	MOL004835	Glypallichalcone	PRKACA	NO
licorice	MOL004835	Glypallichalcone	CCNA2	NO
licorice	MOL004835	Glypallichalcone	NCOA1	NO
licorice	MOL004835	Glypallichalcone	PKIA	NO
licorice	MOL004835	Glypallichalcone	CALM3	NO
licorice	MOL004907	Glyzaglabrin	NOS2	YES
licorice	MOL004907	Glyzaglabrin	PTGS1	NO
licorice	MOL004907	Glyzaglabrin	ESR1	YES
licorice	MOL004907	Glyzaglabrin	AR	YES
licorice	MOL004907	Glyzaglabrin	PPARG	NO
licorice	MOL004907	Glyzaglabrin	PTGS2	YES
licorice	MOL004907	Glyzaglabrin	ESR2	YES
licorice	MOL004907	Glyzaglabrin	DPP4	NO
licorice	MOL004907	Glyzaglabrin	MAPK14	YES
licorice	MOL004907	Glyzaglabrin	GSK3B	YES
licorice	MOL004907	Glyzaglabrin	HSP90AA1	YES

licorice	MOL004907	Glyzaglabrin	CDK2	NO
licorice	MOL004907	Glyzaglabrin	CHEK1	NO
licorice	MOL004907	Glyzaglabrin	PRKACA	NO
licorice	MOL004907	Glyzaglabrin	PRSS1	NO
licorice	MOL004907	Glyzaglabrin	CCNA2	NO
licorice	MOL004957	HMO	NOS2	YES
licorice	MOL004957	HMO	PTGS1	NO
licorice	MOL004957	HMO	CHRM1	NO
licorice	MOL004957	HMO	ESR1	YES
licorice	MOL004957	HMO	AR	YES
licorice	MOL004957	HMO	SCN5A	NO
licorice	MOL004957	HMO	PPARG	NO
licorice	MOL004957	HMO	PTGS2	YES
licorice	MOL004957	HMO	RXRA	NO
licorice	MOL004957	HMO	PDE3A	NO
licorice	MOL004957	HMO	SLC6A3	NO
licorice	MOL004957	HMO	ADRB2	NO
licorice	MOL004957	HMO	SLC6A4	YES
licorice	MOL004957	HMO	ESR2	YES
licorice	MOL004957	HMO	DPP4	NO
licorice	MOL004957	HMO	MAPK14	YES
licorice	MOL004957	HMO	GSK3B	YES
licorice	MOL004957	HMO	CDK2	NO
licorice	MOL004957	HMO	MAOB	NO
licorice	MOL004957	HMO	CHEK1	NO
licorice	MOL004957	HMO	PRKACA	NO
licorice	MOL004957	HMO	IGHG1	NO
licorice	MOL004957	HMO	PRSS1	NO
licorice	MOL004957	HMO	CCNA2	NO
licorice	MOL004957	HMO	PKIA	NO
licorice	MOL004957	HMO	CALM3	NO
licorice	MOL004985	icos-5-enoic acid	NCOA2	NO
licorice	MOL001484	Inermine	PTGS1	NO
licorice	MOL001484	Inermine	CHRM3	NO
licorice	MOL001484	Inermine	SCN5A	NO
licorice	MOL001484	Inermine	PTGS2	YES
licorice	MOL001484	Inermine	RXRA	NO
licorice	MOL001484	Inermine	ADRA1B	NO
licorice	MOL001484	Inermine	ADRA1D	NO
licorice	MOL001484	Inermine	PRKACA	NO
licorice	MOL001484	Inermine	IGHG1	NO
licorice	MOL001484	Inermine	PRSS1	NO
licorice	MOL001484	Inermine	CALM3	NO
licorice	MOL001484	Inermine	CHRM1	NO
licorice	MOL001484	Inermine	ADRB2	NO
licorice	MOL001484	Inermine	OPRM1	NO
licorice	MOL001484	Inermine	HSP90AA1	YES
licorice	MOL004980	Inflacoumarin A	F2	YES
licorice	MOL004980	Inflacoumarin A	ESR1	YES
licorice	MOL004980	Inflacoumarin A	AR	YES
licorice	MOL004980	Inflacoumarin A	PPARG	NO
licorice	MOL004980	Inflacoumarin A	F10	YES
licorice	MOL004980	Inflacoumarin A	PTGS2	YES
licorice	MOL004980	Inflacoumarin A	ADRB2	NO

licorice	MOL004980	Inflacoumarin A	DPP4	NO
licorice	MOL004980	Inflacoumarin A	HSP90AA1	YES
licorice	MOL004980	Inflacoumarin A	PRSS1	NO
licorice	MOL004980	Inflacoumarin A	NCOA2	NO
licorice	MOL004980	Inflacoumarin A	CALM3	NO
licorice	MOL004980	Inflacoumarin A	PTGS1	NO
licorice	MOL004980	Inflacoumarin A	SCN5A	NO
Pogostemon Cablin (	MOL005916	irisolidone	NOS2	YES
Pogostemon Cablin (	MOL005916	irisolidone	PTGS1	NO
Pogostemon Cablin (	MOL005916	irisolidone	ESR1	YES
Pogostemon Cablin (	MOL005916	irisolidone	AR	YES
Pogostemon Cablin (	MOL005916	irisolidone	SCN5A	NO
Pogostemon Cablin (	MOL005916	irisolidone	PPARG	NO
Pogostemon Cablin (	MOL005916	irisolidone	PTGS2	YES
Pogostemon Cablin (	MOL005916	irisolidone	RXRA	NO
Pogostemon Cablin (	MOL005916	irisolidone	ACHE	NO
Pogostemon Cablin (	MOL005916	irisolidone	ADRB2	NO
Pogostemon Cablin (	MOL005916	irisolidone	ESR2	YES
Pogostemon Cablin (	MOL005916	irisolidone	DPP4	NO
Pogostemon Cablin (	MOL005916	irisolidone	MAPK14	YES
Pogostemon Cablin (	MOL005916	irisolidone	GSK3B	YES
Pogostemon Cablin (	MOL005916	irisolidone	CDK2	NO
Pogostemon Cablin (	MOL005916	irisolidone	CHEK1	NO
Pogostemon Cablin (	MOL005916	irisolidone	IGHG1	NO
Pogostemon Cablin (	MOL005916	irisolidone	PRSS1	NO
Pogostemon Cablin (	MOL005916	irisolidone	CCNA2	NO
Pogostemon Cablin (	MOL005916	irisolidone	NCOA2	NO
Pogostemon Cablin (	MOL005916	irisolidone	NCOA1	NO
Pogostemon Cablin (	MOL005916	irisolidone	CALM3	NO
Pogostemon Cablin (	MOL005916	irisolidone	HSP90AA1	YES
Pogostemon Cablin (	MOL005916	irisolidone	RELA	YES
Pogostemon Cablin (	MOL005916	irisolidone	MMP9	YES
Pogostemon Cablin (	MOL005916	irisolidone	MAPK1	YES
Pogostemon Cablin (	MOL005916	irisolidone	TNF	YES
Pogostemon Cablin (	MOL005916	irisolidone	JUN	YES
Pogostemon Cablin (	MOL005916	irisolidone	IL1B	YES
Pogostemon Cablin (	MOL005916	irisolidone	HMGCR	YES
licorice	MOL004948	Isoglycyrol	NOS2	YES
licorice	MOL004948	Isoglycyrol	ESR1	YES
licorice	MOL004948	Isoglycyrol	AR	YES
licorice	MOL004948	Isoglycyrol	PTGS2	YES
licorice	MOL004948	Isoglycyrol	DPP4	NO
licorice	MOL004948	Isoglycyrol	GSK3B	YES
licorice	MOL004949	Isolicoflavonol	NOS2	YES
licorice	MOL004949	Isolicoflavonol	F2	YES
licorice	MOL004949	Isolicoflavonol	ESR1	YES
licorice	MOL004949	Isolicoflavonol	AR	YES
licorice	MOL004949	Isolicoflavonol	PPARG	NO
licorice	MOL004949	Isolicoflavonol	F10	YES
licorice	MOL004949	Isolicoflavonol	PTGS2	YES
licorice	MOL004949	Isolicoflavonol	GSK3B	YES
licorice	MOL004949	Isolicoflavonol	HSP90AA1	YES
licorice	MOL004949	Isolicoflavonol	CDK2	NO
licorice	MOL004949	Isolicoflavonol	PRSS1	NO

licorice	MOL004949	Isolicoflavonol	CCNA2	NO
licorice	MOL004949	Isolicoflavonol	NCOA2	NO
licorice	MOL004949	Isolicoflavonol	CALM3	NO
Radix Bupleuri	MOL000354	isorhamnetin	NOS2	YES
Radix Bupleuri	MOL000354	isorhamnetin	PTGS1	NO
Radix Bupleuri	MOL000354	isorhamnetin	ESR1	YES
Radix Bupleuri	MOL000354	isorhamnetin	AR	YES
Radix Bupleuri	MOL000354	isorhamnetin	PPARG	NO
Radix Bupleuri	MOL000354	isorhamnetin	PTGS2	YES
Radix Bupleuri	MOL000354	isorhamnetin	PTPN1	YES
Radix Bupleuri	MOL000354	isorhamnetin	ESR2	YES
Radix Bupleuri	MOL000354	isorhamnetin	DPP4	NO
Radix Bupleuri	MOL000354	isorhamnetin	MAPK14	YES
Radix Bupleuri	MOL000354	isorhamnetin	GSK3B	YES
Radix Bupleuri	MOL000354	isorhamnetin	HSP90AA1	YES
Radix Bupleuri	MOL000354	isorhamnetin	CDK2	NO
Radix Bupleuri	MOL000354	isorhamnetin	PRKACA	NO
Radix Bupleuri	MOL000354	isorhamnetin	PRSS1	NO
Radix Bupleuri	MOL000354	isorhamnetin	CCNA2	NO
Radix Bupleuri	MOL000354	isorhamnetin	NCOA2	NO
Radix Bupleuri	MOL000354	isorhamnetin	CALM3	NO
Radix Bupleuri	MOL000354	isorhamnetin	PYGM	NO
Radix Bupleuri	MOL000354	isorhamnetin	PPARD	NO
Radix Bupleuri	MOL000354	isorhamnetin	CHEK1	NO
Radix Bupleuri	MOL000354	isorhamnetin	AKR1B1	NO
Radix Bupleuri	MOL000354	isorhamnetin	NCOA1	NO
Radix Bupleuri	MOL000354	isorhamnetin	F7	NO
Radix Bupleuri	MOL000354	isorhamnetin	F2	YES
Radix Bupleuri	MOL000354	isorhamnetin	ACHE	NO
Radix Bupleuri	MOL000354	isorhamnetin	MAOB	NO
Radix Bupleuri	MOL000354	isorhamnetin	GRIA2	NO
Radix Bupleuri	MOL000354	isorhamnetin	RELA	YES
Radix Bupleuri	MOL000354	isorhamnetin	NCF1	YES
Radix Bupleuri	MOL000354	isorhamnetin	OLR1	NO
Radix Bupleuri	MOL000354	isorhamnetin	NOS2	YES
Radix Bupleuri	MOL000354	isorhamnetin	PTGS1	NO
Radix Bupleuri	MOL000354	isorhamnetin	ESR1	YES
Radix Bupleuri	MOL000354	isorhamnetin	AR	YES
Radix Bupleuri	MOL000354	isorhamnetin	PPARG	NO
Radix Bupleuri	MOL000354	isorhamnetin	PTGS2	YES
Radix Bupleuri	MOL000354	isorhamnetin	PTPN1	YES
Radix Bupleuri	MOL000354	isorhamnetin	ESR2	YES
Radix Bupleuri	MOL000354	isorhamnetin	DPP4	NO
Radix Bupleuri	MOL000354	isorhamnetin	MAPK14	YES
Radix Bupleuri	MOL000354	isorhamnetin	GSK3B	YES
Radix Bupleuri	MOL000354	isorhamnetin	HSP90AA1	YES
Radix Bupleuri	MOL000354	isorhamnetin	CDK2	NO
Radix Bupleuri	MOL000354	isorhamnetin	PRKACA	NO
Radix Bupleuri	MOL000354	isorhamnetin	PRSS1	NO
Radix Bupleuri	MOL000354	isorhamnetin	CCNA2	NO
Radix Bupleuri	MOL000354	isorhamnetin	NCOA2	NO
Radix Bupleuri	MOL000354	isorhamnetin	CALM3	NO
Radix Bupleuri	MOL000354	isorhamnetin	PYGM	NO
Radix Bupleuri	MOL000354	isorhamnetin	PPARD	NO

Radix Bupleuri	MOL000354	isorhamnetin	CHEK1	NO
Radix Bupleuri	MOL000354	isorhamnetin	AKR1B1	NO
Radix Bupleuri	MOL000354	isorhamnetin	NCOA1	NO
Radix Bupleuri	MOL000354	isorhamnetin	F7	NO
Radix Bupleuri	MOL000354	isorhamnetin	F2	YES
Radix Bupleuri	MOL000354	isorhamnetin	ACHE	NO
Radix Bupleuri	MOL000354	isorhamnetin	MAOB	NO
Radix Bupleuri	MOL000354	isorhamnetin	GRIA2	NO
Radix Bupleuri	MOL000354	isorhamnetin	RELA	YES
Radix Bupleuri	MOL000354	isorhamnetin	NCF1	YES
Radix Bupleuri	MOL000354	isorhamnetin	OLR1	NO
licorice	MOL004814	Isotrifoliol	NOS2	YES
licorice	MOL004814	Isotrifoliol	ESR1	YES
licorice	MOL004814	Isotrifoliol	AR	YES
licorice	MOL004814	Isotrifoliol	PTGS2	YES
licorice	MOL004814	Isotrifoliol	ESR2	YES
licorice	MOL004814	Isotrifoliol	MAPK14	YES
licorice	MOL004814	Isotrifoliol	GSK3B	YES
licorice	MOL004814	Isotrifoliol	HSP90AA1	YES
licorice	MOL004814	Isotrifoliol	CDK2	NO
licorice	MOL004814	Isotrifoliol	CHEK1	NO
licorice	MOL004814	Isotrifoliol	PRKACA	NO
licorice	MOL004814	Isotrifoliol	CCNA2	NO
licorice	MOL000239	Jaranol	NOS2	YES
licorice	MOL000239	Jaranol	PTGS1	NO
licorice	MOL000239	Jaranol	AR	YES
licorice	MOL000239	Jaranol	SCN5A	NO
licorice	MOL000239	Jaranol	PTGS2	YES
licorice	MOL000239	Jaranol	ESR2	YES
licorice	MOL000239	Jaranol	DPP4	NO
licorice	MOL000239	Jaranol	HSP90AA1	YES
licorice	MOL000239	Jaranol	CDK2	NO
licorice	MOL000239	Jaranol	CHEK1	NO
licorice	MOL000239	Jaranol	PRSS1	NO
licorice	MOL000239	Jaranol	NCOA2	NO
licorice	MOL000239	Jaranol	CALM3	NO
Radix Bupleuri	MOL000422	kaempferol	NOS2	YES
Radix Bupleuri	MOL000422	kaempferol	PTGS1	NO
Radix Bupleuri	MOL000422	kaempferol	AR	YES
Radix Bupleuri	MOL000422	kaempferol	PPARG	NO
Radix Bupleuri	MOL000422	kaempferol	PTGS2	YES
Radix Bupleuri	MOL000422	kaempferol	HSP90AA1	YES
Radix Bupleuri	MOL000422	kaempferol	PRKACA	NO
Radix Bupleuri	MOL000422	kaempferol	NCOA2	NO
Radix Bupleuri	MOL000422	kaempferol	DPP4	NO
Radix Bupleuri	MOL000422	kaempferol	PRSS1	NO
Radix Bupleuri	MOL000422	kaempferol	PGR	NO
Radix Bupleuri	MOL000422	kaempferol	F2	YES
Radix Bupleuri	MOL000422	kaempferol	CHRM1	NO
Radix Bupleuri	MOL000422	kaempferol	ACHE	NO
Radix Bupleuri	MOL000422	kaempferol	SLC6A2	NO
Radix Bupleuri	MOL000422	kaempferol	CHRM2	NO
Radix Bupleuri	MOL000422	kaempferol	ADRA1B	NO
Radix Bupleuri	MOL000422	kaempferol	TOP2A	NO

Radix Bupleuri	MOL000422	kaempferol	F7	NO
Radix Bupleuri	MOL000422	kaempferol	CALM3	NO
Radix Bupleuri	MOL000422	kaempferol	RELA	YES
Radix Bupleuri	MOL000422	kaempferol	IKBKB	NO
Radix Bupleuri	MOL000422	kaempferol	AKT1	YES
Radix Bupleuri	MOL000422	kaempferol	BCL2	YES
Radix Bupleuri	MOL000422	kaempferol	BAX	NO
Radix Bupleuri	MOL000422	kaempferol	TNF	YES
Radix Bupleuri	MOL000422	kaempferol	JUN	YES
Radix Bupleuri	MOL000422	kaempferol	AHSA1	NO
Radix Bupleuri	MOL000422	kaempferol	CASP3	YES
Radix Bupleuri	MOL000422	kaempferol	MAPK8	YES
Radix Bupleuri	MOL000422	kaempferol	MMP1	NO
Radix Bupleuri	MOL000422	kaempferol	STAT1	YES
Radix Bupleuri	MOL000422	kaempferol	CDK1	NO
Radix Bupleuri	MOL000422	kaempferol	PPARG	NO
Radix Bupleuri	MOL000422	kaempferol	HMOX1	YES
Radix Bupleuri	MOL000422	kaempferol	CYP3A4	YES
Radix Bupleuri	MOL000422	kaempferol	METTL1	NO
Radix Bupleuri	MOL000422	kaempferol	CYP1A1	NO
Radix Bupleuri	MOL000422	kaempferol	ICAM1	YES
Radix Bupleuri	MOL000422	kaempferol	SELE	YES
Radix Bupleuri	MOL000422	kaempferol	VCAM1	YES
Radix Bupleuri	MOL000422	kaempferol	NR1I2	NO
Radix Bupleuri	MOL000422	kaempferol	CYP1B1	NO
Radix Bupleuri	MOL000422	kaempferol	ALOX5	NO
Radix Bupleuri	MOL000422	kaempferol	HAS2	NO
Radix Bupleuri	MOL000422	kaempferol	AHR	YES
Radix Bupleuri	MOL000422	kaempferol	PSMD3	NO
Radix Bupleuri	MOL000422	kaempferol	SLC2A4	NO
Radix Bupleuri	MOL000422	kaempferol	NR1I3	NO
Radix Bupleuri	MOL000422	kaempferol	INSR	YES
Radix Bupleuri	MOL000422	kaempferol	DIO1	NO
Radix Bupleuri	MOL000422	kaempferol	PPP3CA	NO
Radix Bupleuri	MOL000422	kaempferol	GSTM1	NO
Radix Bupleuri	MOL000422	kaempferol	GSTM2	NO
Radix Bupleuri	MOL000422	kaempferol	AKR1C3	NO
Radix Bupleuri	MOL000422	kaempferol	SLPI	NO
Radix Bupleuri	MOL000422	kaempferol	NOS2	YES
Radix Bupleuri	MOL000422	kaempferol	PTGS1	NO
Radix Bupleuri	MOL000422	kaempferol	AR	YES
Radix Bupleuri	MOL000422	kaempferol	PPARG	NO
Radix Bupleuri	MOL000422	kaempferol	PTGS2	YES
Radix Bupleuri	MOL000422	kaempferol	HSP90AA1	YES
Radix Bupleuri	MOL000422	kaempferol	PRKACA	NO
Radix Bupleuri	MOL000422	kaempferol	NCOA2	NO
Radix Bupleuri	MOL000422	kaempferol	DPP4	NO
Radix Bupleuri	MOL000422	kaempferol	PRSS1	NO
Radix Bupleuri	MOL000422	kaempferol	PGR	NO
Radix Bupleuri	MOL000422	kaempferol	F2	YES
Radix Bupleuri	MOL000422	kaempferol	CHRM1	NO
Radix Bupleuri	MOL000422	kaempferol	ACHE	NO
Radix Bupleuri	MOL000422	kaempferol	SLC6A2	NO
Radix Bupleuri	MOL000422	kaempferol	CHRM2	NO

Radix Bupleuri	MOL000422	kaempferol	ADRA1B	NO
Radix Bupleuri	MOL000422	kaempferol	TOP2A	NO
Radix Bupleuri	MOL000422	kaempferol	F7	NO
Radix Bupleuri	MOL000422	kaempferol	CALM3	NO
Radix Bupleuri	MOL000422	kaempferol	RELA	YES
Radix Bupleuri	MOL000422	kaempferol	IKBKB	NO
Radix Bupleuri	MOL000422	kaempferol	AKT1	YES
Radix Bupleuri	MOL000422	kaempferol	BCL2	YES
Radix Bupleuri	MOL000422	kaempferol	BAX	NO
Radix Bupleuri	MOL000422	kaempferol	TNF	YES
Radix Bupleuri	MOL000422	kaempferol	JUN	YES
Radix Bupleuri	MOL000422	kaempferol	AHSA1	NO
Radix Bupleuri	MOL000422	kaempferol	CASP3	YES
Radix Bupleuri	MOL000422	kaempferol	MAPK8	YES
Radix Bupleuri	MOL000422	kaempferol	MMP1	NO
Radix Bupleuri	MOL000422	kaempferol	STAT1	YES
Radix Bupleuri	MOL000422	kaempferol	CDK1	NO
Radix Bupleuri	MOL000422	kaempferol	PPARG	NO
Radix Bupleuri	MOL000422	kaempferol	HMOX1	YES
Radix Bupleuri	MOL000422	kaempferol	CYP3A4	YES
Radix Bupleuri	MOL000422	kaempferol	METTL1	NO
Radix Bupleuri	MOL000422	kaempferol	CYP1A1	NO
Radix Bupleuri	MOL000422	kaempferol	ICAM1	YES
Radix Bupleuri	MOL000422	kaempferol	SELE	YES
Radix Bupleuri	MOL000422	kaempferol	VCAM1	YES
Radix Bupleuri	MOL000422	kaempferol	NR1I2	NO
Radix Bupleuri	MOL000422	kaempferol	CYP1B1	NO
Radix Bupleuri	MOL000422	kaempferol	ALOX5	NO
Radix Bupleuri	MOL000422	kaempferol	HAS2	NO
Radix Bupleuri	MOL000422	kaempferol	AHR	YES
Radix Bupleuri	MOL000422	kaempferol	PSMD3	NO
Radix Bupleuri	MOL000422	kaempferol	SLC2A4	NO
Radix Bupleuri	MOL000422	kaempferol	NR1I3	NO
Radix Bupleuri	MOL000422	kaempferol	INSR	YES
Radix Bupleuri	MOL000422	kaempferol	DIO1	NO
Radix Bupleuri	MOL000422	kaempferol	PPP3CA	NO
Radix Bupleuri	MOL000422	kaempferol	GSTM1	NO
Radix Bupleuri	MOL000422	kaempferol	GSTM2	NO
Radix Bupleuri	MOL000422	kaempferol	AKR1C3	NO
Radix Bupleuri	MOL000422	kaempferol	SLPI	NO
licorice	MOL004988	Kanzonol F	ESR1	YES
licorice	MOL004988	Kanzonol F	AR	YES
licorice	MOL004988	Kanzonol F	F10	YES
licorice	MOL004988	Kanzonol F	PTGS2	YES
licorice	MOL004988	Kanzonol F	ESR2	YES
licorice	MOL004988	Kanzonol F	NCOA2	NO
licorice	MOL004988	Kanzonol F	CALM3	NO
licorice	MOL004820	kanzonols W	NOS2	YES
licorice	MOL004820	kanzonols W	PTGS1	NO
licorice	MOL004820	kanzonols W	ESR1	YES
licorice	MOL004820	kanzonols W	AR	YES
licorice	MOL004820	kanzonols W	SCN5A	NO
licorice	MOL004820	kanzonols W	PPARG	NO
licorice	MOL004820	kanzonols W	F10	YES

licorice	MOL004820	kanzonols W	PTGS2	YES
licorice	MOL004820	kanzonols W	RXRA	NO
licorice	MOL004820	kanzonols W	TOP2A	NO
licorice	MOL004820	kanzonols W	ESR2	YES
licorice	MOL004820	kanzonols W	MAPK14	YES
licorice	MOL004820	kanzonols W	GSK3B	YES
licorice	MOL004820	kanzonols W	CDK2	NO
licorice	MOL004820	kanzonols W	CHEK1	NO
licorice	MOL004820	kanzonols W	PRSS1	NO
licorice	MOL004820	kanzonols W	CCNA2	NO
licorice	MOL004820	kanzonols W	NCOA2	NO
licorice	MOL004820	kanzonols W	NCOA1	NO
licorice	MOL004820	kanzonols W	CALM3	NO
licorice	MOL005003	Licoagrocarpin	NOS2	YES
licorice	MOL005003	Licoagrocarpin	PTGS1	NO
licorice	MOL005003	Licoagrocarpin	CHRM3	NO
licorice	MOL005003	Licoagrocarpin	F2	YES
licorice	MOL005003	Licoagrocarpin	KCNH2	NO
licorice	MOL005003	Licoagrocarpin	CHRM1	NO
licorice	MOL005003	Licoagrocarpin	ESR1	YES
licorice	MOL005003	Licoagrocarpin	AR	YES
licorice	MOL005003	Licoagrocarpin	SCN5A	NO
licorice	MOL005003	Licoagrocarpin	PPARG	NO
licorice	MOL005003	Licoagrocarpin	F10	YES
licorice	MOL005003	Licoagrocarpin	CHRM5	NO
licorice	MOL005003	Licoagrocarpin	PTGS2	YES
licorice	MOL005003	Licoagrocarpin	RXRA	NO
licorice	MOL005003	Licoagrocarpin	ACHE	NO
licorice	MOL005003	Licoagrocarpin	ADRA1B	NO
licorice	MOL005003	Licoagrocarpin	ADRB2	NO
licorice	MOL005003	Licoagrocarpin	ESR2	YES
licorice	MOL005003	Licoagrocarpin	MAPK14	YES
licorice	MOL005003	Licoagrocarpin	GSK3B	YES
licorice	MOL005003	Licoagrocarpin	HSP90AA1	YES
licorice	MOL005003	Licoagrocarpin	CDK2	NO
licorice	MOL005003	Licoagrocarpin	METTL1	NO
licorice	MOL005003	Licoagrocarpin	PRSS1	NO
licorice	MOL005003	Licoagrocarpin	CCNA2	NO
licorice	MOL005003	Licoagrocarpin	NCOA2	NO
licorice	MOL005003	Licoagrocarpin	CALM3	NO
licorice	MOL005012	Licoagroisoflavone	NOS2	YES
licorice	MOL005012	Licoagroisoflavone	F2	YES
licorice	MOL005012	Licoagroisoflavone	ESR1	YES
licorice	MOL005012	Licoagroisoflavone	AR	YES
licorice	MOL005012	Licoagroisoflavone	SCN5A	NO
licorice	MOL005012	Licoagroisoflavone	PPARG	NO
licorice	MOL005012	Licoagroisoflavone	F10	YES
licorice	MOL005012	Licoagroisoflavone	PTGS2	YES
licorice	MOL005012	Licoagroisoflavone	ESR2	YES
licorice	MOL005012	Licoagroisoflavone	DPP4	NO
licorice	MOL005012	Licoagroisoflavone	MAPK14	YES
licorice	MOL005012	Licoagroisoflavone	GSK3B	YES
licorice	MOL005012	Licoagroisoflavone	CDK2	NO
licorice	MOL005012	Licoagroisoflavone	CHEK1	NO

licorice	MOL005012	Licoagroisoflavone	PRSS1	NO
licorice	MOL005012	Licoagroisoflavone	CCNA2	NO
licorice	MOL005012	Licoagroisoflavone	CALM3	NO
licorice	MOL000497	licochalcone a	NOS2	YES
licorice	MOL000497	licochalcone a	PTGS1	NO
licorice	MOL000497	licochalcone a	CHRM1	NO
licorice	MOL000497	licochalcone a	ESR1	YES
licorice	MOL000497	licochalcone a	AR	YES
licorice	MOL000497	licochalcone a	SCN5A	NO
licorice	MOL000497	licochalcone a	PPARG	NO
licorice	MOL000497	licochalcone a	F10	YES
licorice	MOL000497	licochalcone a	PTGS2	YES
licorice	MOL000497	licochalcone a	CA2	NO
licorice	MOL000497	licochalcone a	ADRA1B	NO
licorice	MOL000497	licochalcone a	SLC6A3	NO
licorice	MOL000497	licochalcone a	ESR2	YES
licorice	MOL000497	licochalcone a	MAPK14	YES
licorice	MOL000497	licochalcone a	GSK3B	YES
licorice	MOL000497	licochalcone a	HSP90AA1	YES
licorice	MOL000497	licochalcone a	CDK2	NO
licorice	MOL000497	licochalcone a	CHEK1	NO
licorice	MOL000497	licochalcone a	CCNA2	NO
licorice	MOL000497	licochalcone a	CALM3	NO
licorice	MOL000497	licochalcone a	ADRB2	NO
licorice	MOL000497	licochalcone a	NCOA2	NO
licorice	MOL000497	licochalcone a	RELA	YES
licorice	MOL000497	licochalcone a	STAT3	YES
licorice	MOL000497	licochalcone a	CCND1	NO
licorice	MOL000497	licochalcone a	BCL2	YES
licorice	MOL000497	licochalcone a	EIF6	NO
licorice	MOL000497	licochalcone a	MAPK1	YES
licorice	MOL000497	licochalcone a	RB1	NO
licorice	MOL000497	licochalcone a	CDK4	NO
licorice	MOL000497	licochalcone a	FOSL2	NO
licorice	MOL004841	Licochalcone B	NOS2	YES
licorice	MOL004841	Licochalcone B	PTGS1	NO
licorice	MOL004841	Licochalcone B	ESR1	YES
licorice	MOL004841	Licochalcone B	AR	YES
licorice	MOL004841	Licochalcone B	PPARG	NO
licorice	MOL004841	Licochalcone B	PTGS2	YES
licorice	MOL004841	Licochalcone B	CA2	NO
licorice	MOL004841	Licochalcone B	PDE3A	NO
licorice	MOL004841	Licochalcone B	ADRB2	NO
licorice	MOL004841	Licochalcone B	ESR2	YES
licorice	MOL004841	Licochalcone B	MAPK14	YES
licorice	MOL004841	Licochalcone B	GSK3B	YES
licorice	MOL004841	Licochalcone B	HSP90AA1	YES
licorice	MOL004841	Licochalcone B	CDK2	NO
licorice	MOL004841	Licochalcone B	CHEK1	NO
licorice	MOL004841	Licochalcone B	PRKACA	NO
licorice	MOL004841	Licochalcone B	CCNA2	NO
licorice	MOL004841	Licochalcone B	CALM3	NO
licorice	MOL004848	licochalcone G	NOS2	YES
licorice	MOL004848	licochalcone G	ESR1	YES

licorice	MOL004848	licochalcone G	AR	YES
licorice	MOL004848	licochalcone G	PPARG	NO
licorice	MOL004848	licochalcone G	F10	YES
licorice	MOL004848	licochalcone G	PTGS2	YES
licorice	MOL004848	licochalcone G	KDR	YES
licorice	MOL004848	licochalcone G	ESR2	YES
licorice	MOL004848	licochalcone G	MAPK14	YES
licorice	MOL004848	licochalcone G	GSK3B	YES
licorice	MOL004848	licochalcone G	HSP90AA1	YES
licorice	MOL004848	licochalcone G	CDK2	NO
licorice	MOL004848	licochalcone G	IGHG1	NO
licorice	MOL004848	licochalcone G	CCNA2	NO
licorice	MOL004848	licochalcone G	NCOA2	NO
licorice	MOL004848	licochalcone G	CALM3	NO
licorice	MOL004882	Licocoumarone	ESR1	YES
licorice	MOL004882	Licocoumarone	AR	YES
licorice	MOL004882	Licocoumarone	ESR2	YES
licorice	MOL004882	Licocoumarone	GSK3B	YES
licorice	MOL004882	Licocoumarone	HSP90AA1	YES
licorice	MOL004882	Licocoumarone	CDK2	NO
licorice	MOL004882	Licocoumarone	CCNA2	NO
licorice	MOL004885	licoisoflavanone	NOS2	YES
licorice	MOL004885	licoisoflavanone	PTGS1	NO
licorice	MOL004885	licoisoflavanone	ESR1	YES
licorice	MOL004885	licoisoflavanone	AR	YES
licorice	MOL004885	licoisoflavanone	SCN5A	NO
licorice	MOL004885	licoisoflavanone	PPARG	NO
licorice	MOL004885	licoisoflavanone	F10	YES
licorice	MOL004885	licoisoflavanone	PTGS2	YES
licorice	MOL004885	licoisoflavanone	F7	NO
licorice	MOL004885	licoisoflavanone	ACHE	NO
licorice	MOL004885	licoisoflavanone	TOP2A	NO
licorice	MOL004885	licoisoflavanone	ESR2	YES
licorice	MOL004885	licoisoflavanone	GSK3B	YES
licorice	MOL004885	licoisoflavanone	HSP90AA1	YES
licorice	MOL004885	licoisoflavanone	CDK2	NO
licorice	MOL004885	licoisoflavanone	PRSS1	NO
licorice	MOL004885	licoisoflavanone	CCNA2	NO
licorice	MOL004885	licoisoflavanone	NCOA1	NO
licorice	MOL004885	licoisoflavanone	CALM3	NO
licorice	MOL004883	Licoisoflavone	NOS2	YES
licorice	MOL004883	Licoisoflavone	F2	YES
licorice	MOL004883	Licoisoflavone	ESR1	YES
licorice	MOL004883	Licoisoflavone	AR	YES
licorice	MOL004883	Licoisoflavone	PPARG	NO
licorice	MOL004883	Licoisoflavone	F10	YES
licorice	MOL004883	Licoisoflavone	PTGS2	YES
licorice	MOL004883	Licoisoflavone	KDR	YES
licorice	MOL004883	Licoisoflavone	TOP2A	NO
licorice	MOL004883	Licoisoflavone	DPP4	NO
licorice	MOL004883	Licoisoflavone	MAPK14	YES
licorice	MOL004883	Licoisoflavone	HSP90AA1	YES
licorice	MOL004883	Licoisoflavone	CDK2	NO
licorice	MOL004883	Licoisoflavone	CHEK1	NO

licorice	MOL004883	Licoisoflavone	PRSS1	NO
licorice	MOL004883	Licoisoflavone	CCNA2	NO
licorice	MOL004883	Licoisoflavone	NCOA2	NO
licorice	MOL004883	Licoisoflavone	CALM3	NO
licorice	MOL004884	Licoisoflavone B	NOS2	YES
licorice	MOL004884	Licoisoflavone B	F2	YES
licorice	MOL004884	Licoisoflavone B	ESR1	YES
licorice	MOL004884	Licoisoflavone B	AR	YES
licorice	MOL004884	Licoisoflavone B	PPARG	NO
licorice	MOL004884	Licoisoflavone B	F10	YES
licorice	MOL004884	Licoisoflavone B	PTGS2	YES
licorice	MOL004884	Licoisoflavone B	ACHE	NO
licorice	MOL004884	Licoisoflavone B	TOP2A	NO
licorice	MOL004884	Licoisoflavone B	ESR2	YES
licorice	MOL004884	Licoisoflavone B	GSK3B	YES
licorice	MOL004884	Licoisoflavone B	CDK2	NO
licorice	MOL004884	Licoisoflavone B	CHEK1	NO
licorice	MOL004884	Licoisoflavone B	PRSS1	NO
licorice	MOL004884	Licoisoflavone B	CCNA2	NO
licorice	MOL004884	Licoisoflavone B	CALM3	NO
licorice	MOL004904	licopyranocoumarin	NOS2	YES
licorice	MOL004904	licopyranocoumarin	F2	YES
licorice	MOL004904	licopyranocoumarin	ESR1	YES
licorice	MOL004904	licopyranocoumarin	AR	YES
licorice	MOL004904	licopyranocoumarin	PPARG	NO
licorice	MOL004904	licopyranocoumarin	F10	YES
licorice	MOL004904	licopyranocoumarin	PTGS2	YES
licorice	MOL004904	licopyranocoumarin	F7	NO
licorice	MOL004904	licopyranocoumarin	KDR	YES
licorice	MOL004904	licopyranocoumarin	ACHE	NO
licorice	MOL004904	licopyranocoumarin	TOP2A	NO
licorice	MOL004904	licopyranocoumarin	CDK2	NO
licorice	MOL004904	licopyranocoumarin	PRSS1	NO
licorice	MOL004904	licopyranocoumarin	CCNA2	NO
licorice	MOL004904	licopyranocoumarin	CALM3	NO
licorice	MOL004855	Licoricone	NOS2	YES
licorice	MOL004855	Licoricone	F2	YES
licorice	MOL004855	Licoricone	KCNH2	NO
licorice	MOL004855	Licoricone	ESR1	YES
licorice	MOL004855	Licoricone	AR	YES
licorice	MOL004855	Licoricone	PPARG	NO
licorice	MOL004855	Licoricone	F10	YES
licorice	MOL004855	Licoricone	PTGS2	YES
licorice	MOL004855	Licoricone	KDR	YES
licorice	MOL004855	Licoricone	TOP2A	NO
licorice	MOL004855	Licoricone	CHEK1	NO
licorice	MOL004855	Licoricone	PRSS1	NO
licorice	MOL004855	Licoricone	NCOA2	NO
licorice	MOL004855	Licoricone	CALM3	NO
Radix Bupleuri	MOL001645	Linoleyl acetate	PTGS1	NO
Radix Bupleuri	MOL001645	Linoleyl acetate	PTGS2	YES
Radix Bupleuri	MOL001645	Linoleyl acetate	NCOA2	NO
Radix Bupleuri	MOL001645	Linoleyl acetate	RXRA	NO
licorice	MOL004903	liquiritin	F10	YES

licorice	MOL004903	liquiritin	F7	NO
licorice	MOL004903	liquiritin	CALM3	NO
licorice	MOL004903	liquiritin	PTGS2	YES
licorice	MOL004903	liquiritin	KDR	YES
licorice	MOL004903	liquiritin	SOD1	YES
Radix Bupleuri	MOL004624	Longikaurin A	CHRM1	NO
Radix Bupleuri	MOL004624	Longikaurin A	CHRM2	NO
Radix Bupleuri	MOL004624	Longikaurin A	PRSS1	NO
licorice	MOL003656	Lupiwighteone	NOS2	YES
licorice	MOL003656	Lupiwighteone	F2	YES
licorice	MOL003656	Lupiwighteone	ESR1	YES
licorice	MOL003656	Lupiwighteone	AR	YES
licorice	MOL003656	Lupiwighteone	SCN5A	NO
licorice	MOL003656	Lupiwighteone	PPARG	NO
licorice	MOL003656	Lupiwighteone	F10	YES
licorice	MOL003656	Lupiwighteone	PTGS2	YES
licorice	MOL003656	Lupiwighteone	TOP2A	NO
licorice	MOL003656	Lupiwighteone	ESR2	YES
licorice	MOL003656	Lupiwighteone	DPP4	NO
licorice	MOL003656	Lupiwighteone	MAPK14	YES
licorice	MOL003656	Lupiwighteone	GSK3B	YES
licorice	MOL003656	Lupiwighteone	HSP90AA1	YES
licorice	MOL003656	Lupiwighteone	CDK2	NO
licorice	MOL003656	Lupiwighteone	CHEK1	NO
licorice	MOL003656	Lupiwighteone	PRSS1	NO
licorice	MOL003656	Lupiwighteone	CCNA2	NO
licorice	MOL003656	Lupiwighteone	NCOA2	NO
licorice	MOL003656	Lupiwighteone	CALM3	NO
Codonopsis Radix	MOL000006	luteolin	PTGS1	NO
Codonopsis Radix	MOL000006	luteolin	AR	YES
Codonopsis Radix	MOL000006	luteolin	PTGS2	YES
Codonopsis Radix	MOL000006	luteolin	HSP90AA1	YES
Codonopsis Radix	MOL000006	luteolin	PRSS1	NO
Codonopsis Radix	MOL000006	luteolin	NCOA2	NO
Codonopsis Radix	MOL000006	luteolin	PRKACA	NO
Codonopsis Radix	MOL000006	luteolin	DPP4	NO
Codonopsis Radix	MOL000006	luteolin	RELA	YES
Codonopsis Radix	MOL000006	luteolin	EGFR	YES
Codonopsis Radix	MOL000006	luteolin	AKT1	YES
Codonopsis Radix	MOL000006	luteolin	METTL1	NO
Codonopsis Radix	MOL000006	luteolin	CCND1	NO
Codonopsis Radix	MOL000006	luteolin	BCL2L1	YES
Codonopsis Radix	MOL000006	luteolin	CDKN1A	NO
Codonopsis Radix	MOL000006	luteolin	CASP9	YES
Codonopsis Radix	MOL000006	luteolin	MMP2	YES
Codonopsis Radix	MOL000006	luteolin	MMP9	YES
Codonopsis Radix	MOL000006	luteolin	MAPK1	YES
Codonopsis Radix	MOL000006	luteolin	IL10RB	NO
Codonopsis Radix	MOL000006	luteolin	RB1	NO
Codonopsis Radix	MOL000006	luteolin	CDK4	NO
Codonopsis Radix	MOL000006	luteolin	TNF	YES
Codonopsis Radix	MOL000006	luteolin	JUN	YES
Codonopsis Radix	MOL000006	luteolin	IL6	YES
Codonopsis Radix	MOL000006	luteolin	CASP3	YES

Codonopsis Radix	MOL000006	luteolin	TP53	YES
Codonopsis Radix	MOL000006	luteolin	NFKBIA	YES
Codonopsis Radix	MOL000006	luteolin	TOP1	NO
Codonopsis Radix	MOL000006	luteolin	MDM2	NO
Codonopsis Radix	MOL000006	luteolin	APP	NO
Codonopsis Radix	MOL000006	luteolin	MMP1	NO
Codonopsis Radix	MOL000006	luteolin	PCNA	YES
Codonopsis Radix	MOL000006	luteolin	ERBB2	YES
Codonopsis Radix	MOL000006	luteolin	PPARG	NO
Codonopsis Radix	MOL000006	luteolin	HMOX1	YES
Codonopsis Radix	MOL000006	luteolin	CASP7	NO
Codonopsis Radix	MOL000006	luteolin	ICAM1	YES
Codonopsis Radix	MOL000006	luteolin	MCL1	NO
Codonopsis Radix	MOL000006	luteolin	BIRC5	NO
Codonopsis Radix	MOL000006	luteolin	IL2RB	YES
Codonopsis Radix	MOL000006	luteolin	CCNB1	NO
Codonopsis Radix	MOL000006	luteolin	TYR	YES
Codonopsis Radix	MOL000006	luteolin	IFNG	YES
Codonopsis Radix	MOL000006	luteolin	IL4	YES
Codonopsis Radix	MOL000006	luteolin	TOP2A	NO
Codonopsis Radix	MOL000006	luteolin	XIAP	NO
Codonopsis Radix	MOL000006	luteolin	SLC2A4	NO
Codonopsis Radix	MOL000006	luteolin	INSR	YES
Codonopsis Radix	MOL000006	luteolin	CD40LG	YES
Codonopsis Radix	MOL000006	luteolin	PTGES	NO
Codonopsis Radix	MOL000006	luteolin	NUF2	NO
Codonopsis Radix	MOL000006	luteolin	ADCY2	NO
Codonopsis Radix	MOL000006	luteolin	MET	NO
licorice	MOL000211	Mairin	PGR	NO
Coicis Semen	MOL001494	Mandenol	PTGS1	NO
Coicis Semen	MOL001494	Mandenol	PTGS2	YES
Coicis Semen	MOL001494	Mandenol	NCOA2	NO
licorice	MOL002565	Medicarpin	NOS2	YES
licorice	MOL002565	Medicarpin	PTGS1	NO
licorice	MOL002565	Medicarpin	DRD1	NO
licorice	MOL002565	Medicarpin	CHRM3	NO
licorice	MOL002565	Medicarpin	CHRM1	NO
licorice	MOL002565	Medicarpin	ESR1	YES
licorice	MOL002565	Medicarpin	SCN5A	NO
licorice	MOL002565	Medicarpin	CHRM5	NO
licorice	MOL002565	Medicarpin	PTGS2	YES
licorice	MOL002565	Medicarpin	CHRM4	NO
licorice	MOL002565	Medicarpin	RXRA	NO
licorice	MOL002565	Medicarpin	ADRA1A	NO
licorice	MOL002565	Medicarpin	CHRM2	NO
licorice	MOL002565	Medicarpin	ADRA1B	NO
licorice	MOL002565	Medicarpin	SLC6A3	NO
licorice	MOL002565	Medicarpin	ADRB2	NO
licorice	MOL002565	Medicarpin	SLC6A4	YES
licorice	MOL002565	Medicarpin	OPRM1	NO
licorice	MOL002565	Medicarpin	ESR2	YES
licorice	MOL002565	Medicarpin	DPP4	NO
licorice	MOL002565	Medicarpin	MAPK10	NO
licorice	MOL002565	Medicarpin	HSP90AA1	YES

licorice	MOL002565	Medicarpin	CDK2	NO
licorice	MOL002565	Medicarpin	CHRNA7	NO
licorice	MOL002565	Medicarpin	PRKACA	NO
licorice	MOL002565	Medicarpin	PRSS1	NO
licorice	MOL002565	Medicarpin	CCNA2	NO
licorice	MOL002565	Medicarpin	CALM3	NO
licorice	MOL002565	Medicarpin	OPRD1	NO
licorice	MOL002565	Medicarpin	PDE3A	NO
licorice	MOL002565	Medicarpin	ADRA1D	NO
Codonopsis Radix	MOL007514	methyl icosa-11,14-dienoate	NCOA2	NO
Scutellariae Radix	MOL008206	Moslosooflavone	NOS2	YES
Scutellariae Radix	MOL008206	Moslosooflavone	PTGS1	NO
Scutellariae Radix	MOL008206	Moslosooflavone	F2	YES
Scutellariae Radix	MOL008206	Moslosooflavone	AR	YES
Scutellariae Radix	MOL008206	Moslosooflavone	SCN5A	NO
Scutellariae Radix	MOL008206	Moslosooflavone	PPARG	NO
Scutellariae Radix	MOL008206	Moslosooflavone	PTGS2	YES
Scutellariae Radix	MOL008206	Moslosooflavone	RXRA	NO
Scutellariae Radix	MOL008206	Moslosooflavone	ESR2	YES
Scutellariae Radix	MOL008206	Moslosooflavone	DPP4	NO
Scutellariae Radix	MOL008206	Moslosooflavone	MAPK14	YES
Scutellariae Radix	MOL008206	Moslosooflavone	GSK3B	YES
Scutellariae Radix	MOL008206	Moslosooflavone	HSP90AA1	YES
Scutellariae Radix	MOL008206	Moslosooflavone	CDK2	NO
Scutellariae Radix	MOL008206	Moslosooflavone	CHEK1	NO
Scutellariae Radix	MOL008206	Moslosooflavone	PRKACA	NO
Scutellariae Radix	MOL008206	Moslosooflavone	PRSS1	NO
Scutellariae Radix	MOL008206	Moslosooflavone	NCOA1	NO
Scutellariae Radix	MOL008206	Moslosooflavone	CALM3	NO
Scutellariae Radix	MOL008206	Moslosooflavone	ADRA1B	NO
Scutellariae Radix	MOL008206	Moslosooflavone	ADRB2	NO
Scutellariae Radix	MOL008206	Moslosooflavone	CHRNA7	NO
licorice	MOL004328	naringenin	PTGS1	NO
licorice	MOL004328	naringenin	ESR1	YES
licorice	MOL004328	naringenin	PTGS2	YES
licorice	MOL004328	naringenin	HSP90AA1	YES
licorice	MOL004328	naringenin	DPEP1	NO
licorice	MOL004328	naringenin	PRKACA	NO
licorice	MOL004328	naringenin	RELA	YES
licorice	MOL004328	naringenin	AKT1	YES
licorice	MOL004328	naringenin	BCL2	YES
licorice	MOL004328	naringenin	MAPK3	YES
licorice	MOL004328	naringenin	MAPK1	YES
licorice	MOL004328	naringenin	CASP3	YES
licorice	MOL004328	naringenin	FASN	YES
licorice	MOL004328	naringenin	LDLR	NO
licorice	MOL004328	naringenin	BAD	NO
licorice	MOL004328	naringenin	SOD1	YES
licorice	MOL004328	naringenin	METTL1	NO
licorice	MOL004328	naringenin	PPARG	NO
licorice	MOL004328	naringenin	MTTP	NO
licorice	MOL004328	naringenin	APOB	YES
licorice	MOL004328	naringenin	PLB1	NO
licorice	MOL004328	naringenin	HMGCR	YES

licorice	MOL004328	naringenin	CYP19A1	YES
licorice	MOL004328	naringenin	UGT1A1	NO
licorice	MOL004328	naringenin	PPARA	NO
licorice	MOL004328	naringenin	SREBF1	NO
licorice	MOL004328	naringenin	GSR	YES
licorice	MOL004328	naringenin	ABCC1	NO
licorice	MOL004328	naringenin	ADIPOR2	NO
licorice	MOL004328	naringenin	SOAT2	NO
licorice	MOL004328	naringenin	AKR1C1	NO
licorice	MOL004328	naringenin	GOT1	NO
licorice	MOL004328	naringenin	ABAT	NO
licorice	MOL004328	naringenin	CES1	NO
licorice	MOL004328	naringenin	SOAT1	YES
Scutellariae Radix	MOL002934	NEOBAICALEIN	NOS2	YES
Scutellariae Radix	MOL002934	NEOBAICALEIN	F2	YES
Scutellariae Radix	MOL002934	NEOBAICALEIN	KCNH2	NO
Scutellariae Radix	MOL002934	NEOBAICALEIN	ESR1	YES
Scutellariae Radix	MOL002934	NEOBAICALEIN	AR	YES
Scutellariae Radix	MOL002934	NEOBAICALEIN	SCN5A	NO
Scutellariae Radix	MOL002934	NEOBAICALEIN	PPARG	NO
Scutellariae Radix	MOL002934	NEOBAICALEIN	F10	YES
Scutellariae Radix	MOL002934	NEOBAICALEIN	PTGS2	YES
Scutellariae Radix	MOL002934	NEOBAICALEIN	F7	NO
Scutellariae Radix	MOL002934	NEOBAICALEIN	PTPN1	YES
Scutellariae Radix	MOL002934	NEOBAICALEIN	TOP2A	NO
Scutellariae Radix	MOL002934	NEOBAICALEIN	ESR2	YES
Scutellariae Radix	MOL002934	NEOBAICALEIN	DPP4	NO
Scutellariae Radix	MOL002934	NEOBAICALEIN	PYGM	NO
Scutellariae Radix	MOL002934	NEOBAICALEIN	GSK3B	YES
Scutellariae Radix	MOL002934	NEOBAICALEIN	HSP90AA1	YES
Scutellariae Radix	MOL002934	NEOBAICALEIN	CHEK1	NO
Scutellariae Radix	MOL002934	NEOBAICALEIN	PRSS1	NO
Scutellariae Radix	MOL002934	NEOBAICALEIN	NCOA2	NO
Scutellariae Radix	MOL002934	NEOBAICALEIN	CALM3	NO
Scutellariae Radix	MOL000525	Norwogonin	NOS2	YES
Scutellariae Radix	MOL000525	Norwogonin	PTGS1	NO
Scutellariae Radix	MOL000525	Norwogonin	AR	YES
Scutellariae Radix	MOL000525	Norwogonin	PPARG	NO
Scutellariae Radix	MOL000525	Norwogonin	PTGS2	YES
Scutellariae Radix	MOL000525	Norwogonin	PDE3A	NO
Scutellariae Radix	MOL000525	Norwogonin	DPP4	NO
Scutellariae Radix	MOL000525	Norwogonin	HSP90AA1	YES
Scutellariae Radix	MOL000525	Norwogonin	CDK2	NO
Scutellariae Radix	MOL000525	Norwogonin	CHEK1	NO
Scutellariae Radix	MOL000525	Norwogonin	PRKACA	NO
licorice	MOL005016	Odoratin	NOS2	YES
licorice	MOL005016	Odoratin	PTGS1	NO
licorice	MOL005016	Odoratin	ESR1	YES
licorice	MOL005016	Odoratin	AR	YES
licorice	MOL005016	Odoratin	SCN5A	NO
licorice	MOL005016	Odoratin	PPARG	NO
licorice	MOL005016	Odoratin	PTGS2	YES
licorice	MOL005016	Odoratin	RXRA	NO
licorice	MOL005016	Odoratin	ESR2	YES

licorice	MOL005016	Odoratin	DPP4	NO
licorice	MOL005016	Odoratin	MAPK14	YES
licorice	MOL005016	Odoratin	GSK3B	YES
licorice	MOL005016	Odoratin	HSP90AA1	YES
licorice	MOL005016	Odoratin	CDK2	NO
licorice	MOL005016	Odoratin	CHEK1	NO
licorice	MOL005016	Odoratin	PRSS1	NO
licorice	MOL005016	Odoratin	CCNA2	NO
licorice	MOL005016	Odoratin	NCOA2	NO
licorice	MOL005016	Odoratin	CALM3	NO
Scutellariae Radix	MOL002928	oroxylin a	NOS2	YES
Scutellariae Radix	MOL002928	oroxylin a	PTGS1	NO
Scutellariae Radix	MOL002928	oroxylin a	AR	YES
Scutellariae Radix	MOL002928	oroxylin a	SCN5A	NO
Scutellariae Radix	MOL002928	oroxylin a	PTGS2	YES
Scutellariae Radix	MOL002928	oroxylin a	RXRA	NO
Scutellariae Radix	MOL002928	oroxylin a	PDE3A	NO
Scutellariae Radix	MOL002928	oroxylin a	ADRA1B	NO
Scutellariae Radix	MOL002928	oroxylin a	ADRB2	NO
Scutellariae Radix	MOL002928	oroxylin a	DPP4	NO
Scutellariae Radix	MOL002928	oroxylin a	HSP90AA1	YES
Scutellariae Radix	MOL002928	oroxylin a	PRKACA	NO
Scutellariae Radix	MOL002928	oroxylin a	PRSS1	NO
Scutellariae Radix	MOL002928	oroxylin a	NCOA1	NO
Scutellariae Radix	MOL002928	oroxylin a	CALM3	NO
Scutellariae Radix	MOL002928	oroxylin a	NCOA2	NO
Scutellariae Radix	MOL002928	oroxylin a	PKIA	NO
Scutellariae Radix	MOL002928	oroxylin a	BCL2	YES
Scutellariae Radix	MOL002928	oroxylin a	IL6	YES
Scutellariae Radix	MOL002928	oroxylin a	CASP3	YES
Scutellariae Radix	MOL002928	oroxylin a	CDK1	NO
Scutellariae Radix	MOL002928	oroxylin a	METTL1	NO
Scutellariae Radix	MOL002928	oroxylin a	CCNB1	NO
Scutellariae Radix	MOL002928	oroxylin a	CDK7	NO
Scutellariae Radix	MOL002928	oroxylin a	CYP2C9	NO
Scutellariae Radix	MOL002932	Panicolin	NOS2	YES
Scutellariae Radix	MOL002932	Panicolin	PTGS1	NO
Scutellariae Radix	MOL002932	Panicolin	AR	YES
Scutellariae Radix	MOL002932	Panicolin	SCN5A	NO
Scutellariae Radix	MOL002932	Panicolin	PTGS2	YES
Scutellariae Radix	MOL002932	Panicolin	ESR2	YES
Scutellariae Radix	MOL002932	Panicolin	DPP4	NO
Scutellariae Radix	MOL002932	Panicolin	HSP90AA1	YES
Scutellariae Radix	MOL002932	Panicolin	CDK2	NO
Scutellariae Radix	MOL002932	Panicolin	CHEK1	NO
Scutellariae Radix	MOL002932	Panicolin	PRSS1	NO
Scutellariae Radix	MOL002932	Panicolin	CALM3	NO
Scutellariae Radix	MOL002932	Panicolin	NCOA1	NO
Codonopsis Radix	MOL002140	Perlolyrine	F2	YES
Codonopsis Radix	MOL002140	Perlolyrine	PTGS2	YES
Codonopsis Radix	MOL002140	Perlolyrine	RXRA	NO
Codonopsis Radix	MOL002140	Perlolyrine	PRKACA	NO
Radix Bupleuri	MOL000490	petunidin	NOS2	YES
Radix Bupleuri	MOL000490	petunidin	PTGS1	NO

Radix Bupleuri	MOL000490	petunidin	PTGS2	YES
Radix Bupleuri	MOL000490	petunidin	ESR2	YES
Radix Bupleuri	MOL000490	petunidin	MAPK14	YES
Radix Bupleuri	MOL000490	petunidin	GSK3B	YES
Radix Bupleuri	MOL000490	petunidin	HSP90AA1	YES
Radix Bupleuri	MOL000490	petunidin	NCOA2	NO
licorice	MOL005017	Phaseol	F2	YES
licorice	MOL005017	Phaseol	ESR1	YES
licorice	MOL005017	Phaseol	AR	YES
licorice	MOL005017	Phaseol	PPARG	NO
licorice	MOL005017	Phaseol	PTGS2	YES
licorice	MOL005017	Phaseol	KDR	YES
licorice	MOL005017	Phaseol	MAPK14	YES
licorice	MOL005017	Phaseol	GSK3B	YES
licorice	MOL005017	Phaseol	HSP90AA1	YES
licorice	MOL005017	Phaseol	CDK2	NO
licorice	MOL005017	Phaseol	CHEK1	NO
licorice	MOL005017	Phaseol	PRKACA	NO
licorice	MOL005017	Phaseol	CCNA2	NO
licorice	MOL004833	Phaseolinisoflavan	NOS2	YES
licorice	MOL004833	Phaseolinisoflavan	CHRM1	NO
licorice	MOL004833	Phaseolinisoflavan	ESR1	YES
licorice	MOL004833	Phaseolinisoflavan	AR	YES
licorice	MOL004833	Phaseolinisoflavan	SCN5A	NO
licorice	MOL004833	Phaseolinisoflavan	PPARG	NO
licorice	MOL004833	Phaseolinisoflavan	F10	YES
licorice	MOL004833	Phaseolinisoflavan	PTGS2	YES
licorice	MOL004833	Phaseolinisoflavan	RXRA	NO
licorice	MOL004833	Phaseolinisoflavan	ACHE	NO
licorice	MOL004833	Phaseolinisoflavan	ADRA1B	NO
licorice	MOL004833	Phaseolinisoflavan	ADRB2	NO
licorice	MOL004833	Phaseolinisoflavan	ESR2	YES
licorice	MOL004833	Phaseolinisoflavan	MAPK14	YES
licorice	MOL004833	Phaseolinisoflavan	GSK3B	YES
licorice	MOL004833	Phaseolinisoflavan	CDK2	NO
licorice	MOL004833	Phaseolinisoflavan	CHEK1	NO
licorice	MOL004833	Phaseolinisoflavan	PRSS1	NO
licorice	MOL004833	Phaseolinisoflavan	CCNA2	NO
licorice	MOL004833	Phaseolinisoflavan	NCOA1	NO
licorice	MOL004833	Phaseolinisoflavan	CALM3	NO
Pogostemon Cablin (	MOL005918	phenanthrone	PTGS1	NO
Pogostemon Cablin (	MOL005918	phenanthrone	CHRM3	NO
Pogostemon Cablin (	MOL005918	phenanthrone	F2	YES
Pogostemon Cablin (	MOL005918	phenanthrone	CHRM1	NO
Pogostemon Cablin (	MOL005918	phenanthrone	AR	YES
Pogostemon Cablin (	MOL005918	phenanthrone	SCN5A	NO
Pogostemon Cablin (	MOL005918	phenanthrone	PTGS2	YES
Pogostemon Cablin (	MOL005918	phenanthrone	RXRA	NO
Pogostemon Cablin (	MOL005918	phenanthrone	ADRB2	NO
Pogostemon Cablin (	MOL005918	phenanthrone	OPRM1	NO
Pogostemon Cablin (	MOL005918	phenanthrone	DPP4	NO
Pogostemon Cablin (	MOL005918	phenanthrone	HSP90AA1	YES
Codonopsis Radix	MOL001006	poriferasta-7,22E-dien-3beta	PGR	NO
Codonopsis Radix	MOL001006	poriferasta-7,22E-dien-3beta	NCOA2	NO

Codonopsis Radix	MOL001006	poriferasta-7,22E-dien-3beta	NR3C2	NO
Pogostemon Cablin (	MOL000098	quercetin	PTGS1	NO
Pogostemon Cablin (	MOL000098	quercetin	AR	YES
Pogostemon Cablin (	MOL000098	quercetin	PPARG	NO
Pogostemon Cablin (	MOL000098	quercetin	PTGS2	YES
Pogostemon Cablin (	MOL000098	quercetin	HSP90AA1	YES
Pogostemon Cablin (	MOL000098	quercetin	NCOA2	NO
Pogostemon Cablin (	MOL000098	quercetin	DPP4	NO
Pogostemon Cablin (	MOL000098	quercetin	AKR1B1	NO
Pogostemon Cablin (	MOL000098	quercetin	PRSS1	NO
Pogostemon Cablin (	MOL000098	quercetin	TOP2A	NO
Pogostemon Cablin (	MOL000098	quercetin	F2	YES
Pogostemon Cablin (	MOL000098	quercetin	KCNH2	NO
Pogostemon Cablin (	MOL000098	quercetin	SCN5A	NO
Pogostemon Cablin (	MOL000098	quercetin	F10	YES
Pogostemon Cablin (	MOL000098	quercetin	ADRB2	NO
Pogostemon Cablin (	MOL000098	quercetin	MMP3	YES
Pogostemon Cablin (	MOL000098	quercetin	PRKACA	NO
Pogostemon Cablin (	MOL000098	quercetin	F7	NO
Pogostemon Cablin (	MOL000098	quercetin	RXRA	NO
Pogostemon Cablin (	MOL000098	quercetin	ACHE	NO
Pogostemon Cablin (	MOL000098	quercetin	MAOB	NO
Pogostemon Cablin (	MOL000098	quercetin	RELA	YES
Pogostemon Cablin (	MOL000098	quercetin	EGFR	YES
Pogostemon Cablin (	MOL000098	quercetin	AKT1	YES
Pogostemon Cablin (	MOL000098	quercetin	METTL1	NO
Pogostemon Cablin (	MOL000098	quercetin	CCND1	NO
Pogostemon Cablin (	MOL000098	quercetin	BCL2	YES
Pogostemon Cablin (	MOL000098	quercetin	BCL2L1	YES
Pogostemon Cablin (	MOL000098	quercetin	FOS	NO
Pogostemon Cablin (	MOL000098	quercetin	CDKN1A	NO
Pogostemon Cablin (	MOL000098	quercetin	EIF6	NO
Pogostemon Cablin (	MOL000098	quercetin	BAX	NO
Pogostemon Cablin (	MOL000098	quercetin	CASP9	YES
Pogostemon Cablin (	MOL000098	quercetin	PLAU	NO
Pogostemon Cablin (	MOL000098	quercetin	MMP2	YES
Pogostemon Cablin (	MOL000098	quercetin	MMP9	YES
Pogostemon Cablin (	MOL000098	quercetin	MAPK1	YES
Pogostemon Cablin (	MOL000098	quercetin	IL10RB	NO
Pogostemon Cablin (	MOL000098	quercetin	RB1	NO
Pogostemon Cablin (	MOL000098	quercetin	TNF	YES
Pogostemon Cablin (	MOL000098	quercetin	JUN	YES
Pogostemon Cablin (	MOL000098	quercetin	IL6	YES
Pogostemon Cablin (	MOL000098	quercetin	AHSA1	NO
Pogostemon Cablin (	MOL000098	quercetin	CASP3	YES
Pogostemon Cablin (	MOL000098	quercetin	TP53	YES
Pogostemon Cablin (	MOL000098	quercetin	ELK1	NO
Pogostemon Cablin (	MOL000098	quercetin	NFKBIA	YES
Pogostemon Cablin (	MOL000098	quercetin	ODC1	NO
Pogostemon Cablin (	MOL000098	quercetin	CASP8	NO
Pogostemon Cablin (	MOL000098	quercetin	TOP1	NO
Pogostemon Cablin (	MOL000098	quercetin	RAF1	YES
Pogostemon Cablin (	MOL000098	quercetin	SOD1	YES
Pogostemon Cablin (	MOL000098	quercetin	PRKCA	NO

Pogostemon Cablin (	MOL000098	quercetin	MMP1	NO
Pogostemon Cablin (	MOL000098	quercetin	HIF1A	NO
Pogostemon Cablin (	MOL000098	quercetin	STAT1	YES
Pogostemon Cablin (	MOL000098	quercetin	RUNX1T1	NO
Pogostemon Cablin (	MOL000098	quercetin	CDK1	NO
Pogostemon Cablin (	MOL000098	quercetin	HSPA5	YES
Pogostemon Cablin (	MOL000098	quercetin	ERBB2	YES
Pogostemon Cablin (	MOL000098	quercetin	PPARG	NO
Pogostemon Cablin (	MOL000098	quercetin	ACACA	NO
Pogostemon Cablin (	MOL000098	quercetin	HMOX1	YES
Pogostemon Cablin (	MOL000098	quercetin	CYP3A4	YES
Pogostemon Cablin (	MOL000098	quercetin	CAV1	YES
Pogostemon Cablin (	MOL000098	quercetin	MYC	NO
Pogostemon Cablin (	MOL000098	quercetin	F3	YES
Pogostemon Cablin (	MOL000098	quercetin	GJA1	NO
Pogostemon Cablin (	MOL000098	quercetin	CYP1A1	NO
Pogostemon Cablin (	MOL000098	quercetin	ICAM1	YES
Pogostemon Cablin (	MOL000098	quercetin	IL1B	YES
Pogostemon Cablin (	MOL000098	quercetin	CCL2	YES
Pogostemon Cablin (	MOL000098	quercetin	SELE	YES
Pogostemon Cablin (	MOL000098	quercetin	VCAM1	YES
Pogostemon Cablin (	MOL000098	quercetin	CXCL8	YES
Pogostemon Cablin (	MOL000098	quercetin	PRKCB	NO
Pogostemon Cablin (	MOL000098	quercetin	BIRC5	NO
Pogostemon Cablin (	MOL000098	quercetin	DUOX2	NO
Pogostemon Cablin (	MOL000098	quercetin	NOS3	YES
Pogostemon Cablin (	MOL000098	quercetin	HSPB1	NO
Pogostemon Cablin (	MOL000098	quercetin	IL2RB	YES
Pogostemon Cablin (	MOL000098	quercetin	NR1I2	NO
Pogostemon Cablin (	MOL000098	quercetin	CYP1B1	NO
Pogostemon Cablin (	MOL000098	quercetin	CCNB1	NO
Pogostemon Cablin (	MOL000098	quercetin	PLAT	YES
Pogostemon Cablin (	MOL000098	quercetin	THBD	YES
Pogostemon Cablin (	MOL000098	quercetin	SERPINE1	YES
Pogostemon Cablin (	MOL000098	quercetin	IFNG	YES
Pogostemon Cablin (	MOL000098	quercetin	ALOX5	NO
Pogostemon Cablin (	MOL000098	quercetin	IL1A	YES
Pogostemon Cablin (	MOL000098	quercetin	MPO	NO
Pogostemon Cablin (	MOL000098	quercetin	TOP2A	NO
Pogostemon Cablin (	MOL000098	quercetin	NCF1	YES
Pogostemon Cablin (	MOL000098	quercetin	ABCG2	YES
Pogostemon Cablin (	MOL000098	quercetin	HAS2	NO
Pogostemon Cablin (	MOL000098	quercetin	NFE2L2	YES
Pogostemon Cablin (	MOL000098	quercetin	NQO1	NO
Pogostemon Cablin (	MOL000098	quercetin	PARP1	YES
Pogostemon Cablin (	MOL000098	quercetin	AHR	YES
Pogostemon Cablin (	MOL000098	quercetin	PSMD3	NO
Pogostemon Cablin (	MOL000098	quercetin	SLC2A4	NO
Pogostemon Cablin (	MOL000098	quercetin	COL3A1	NO
Pogostemon Cablin (	MOL000098	quercetin	CXCL11	YES
Pogostemon Cablin (	MOL000098	quercetin	CXCL2	NO
Pogostemon Cablin (	MOL000098	quercetin	DCAF5	NO
Pogostemon Cablin (	MOL000098	quercetin	NR1I3	NO
Pogostemon Cablin (	MOL000098	quercetin	CHEK2	NO

Pogostemon Cablin (	MOL000098	quercetin	INSR	YES
Pogostemon Cablin (	MOL000098	quercetin	CLDN4	NO
Pogostemon Cablin (	MOL000098	quercetin	PPARA	NO
Pogostemon Cablin (	MOL000098	quercetin	PPARD	NO
Pogostemon Cablin (	MOL000098	quercetin	HSF1	YES
Pogostemon Cablin (	MOL000098	quercetin	CXCL10	YES
Pogostemon Cablin (	MOL000098	quercetin	CHUK	NO
Pogostemon Cablin (	MOL000098	quercetin	SPP1	YES
Pogostemon Cablin (	MOL000098	quercetin	RUNX2	NO
Pogostemon Cablin (	MOL000098	quercetin	RASSF1	YES
Pogostemon Cablin (	MOL000098	quercetin	E2F1	NO
Pogostemon Cablin (	MOL000098	quercetin	E2F2	NO
Pogostemon Cablin (	MOL000098	quercetin	ACP3	NO
Pogostemon Cablin (	MOL000098	quercetin	CTSD	NO
Pogostemon Cablin (	MOL000098	quercetin	IGFBP3	NO
Pogostemon Cablin (	MOL000098	quercetin	IGF2	YES
Pogostemon Cablin (	MOL000098	quercetin	CD40LG	YES
Pogostemon Cablin (	MOL000098	quercetin	IRF1	YES
Pogostemon Cablin (	MOL000098	quercetin	ERBB3	NO
Pogostemon Cablin (	MOL000098	quercetin	PON1	NO
Pogostemon Cablin (	MOL000098	quercetin	DIO1	NO
Pogostemon Cablin (	MOL000098	quercetin	PCOLCE	NO
Pogostemon Cablin (	MOL000098	quercetin	NPEPPS	NO
Pogostemon Cablin (	MOL000098	quercetin	HK2	YES
Pogostemon Cablin (	MOL000098	quercetin	RASA1	NO
Pogostemon Cablin (	MOL000098	quercetin	GSTM1	NO
Pogostemon Cablin (	MOL000098	quercetin	GSTM2	NO
Pogostemon Cablin (	MOL000098	quercetin	PTGS1	NO
Pogostemon Cablin (	MOL000098	quercetin	AR	YES
Pogostemon Cablin (	MOL000098	quercetin	PPARG	NO
Pogostemon Cablin (	MOL000098	quercetin	PTGS2	YES
Pogostemon Cablin (	MOL000098	quercetin	HSP90AA1	YES
Pogostemon Cablin (	MOL000098	quercetin	NCOA2	NO
Pogostemon Cablin (	MOL000098	quercetin	DPP4	NO
Pogostemon Cablin (	MOL000098	quercetin	AKR1B1	NO
Pogostemon Cablin (	MOL000098	quercetin	PRSS1	NO
Pogostemon Cablin (	MOL000098	quercetin	TOP2A	NO
Pogostemon Cablin (	MOL000098	quercetin	F2	YES
Pogostemon Cablin (	MOL000098	quercetin	KCNH2	NO
Pogostemon Cablin (	MOL000098	quercetin	SCN5A	NO
Pogostemon Cablin (	MOL000098	quercetin	F10	YES
Pogostemon Cablin (	MOL000098	quercetin	ADRB2	NO
Pogostemon Cablin (	MOL000098	quercetin	MMP3	YES
Pogostemon Cablin (	MOL000098	quercetin	PRKACA	NO
Pogostemon Cablin (	MOL000098	quercetin	F7	NO
Pogostemon Cablin (	MOL000098	quercetin	RXRA	NO
Pogostemon Cablin (	MOL000098	quercetin	ACHE	NO
Pogostemon Cablin (	MOL000098	quercetin	MAOB	NO
Pogostemon Cablin (	MOL000098	quercetin	RELA	YES
Pogostemon Cablin (	MOL000098	quercetin	EGFR	YES
Pogostemon Cablin (	MOL000098	quercetin	AKT1	YES
Pogostemon Cablin (	MOL000098	quercetin	METTL1	NO
Pogostemon Cablin (	MOL000098	quercetin	CCND1	NO
Pogostemon Cablin (	MOL000098	quercetin	BCL2	YES

Pogostemon Cablin (	MOL000098	quercetin	BCL2L1	YES
Pogostemon Cablin (	MOL000098	quercetin	FOS	NO
Pogostemon Cablin (	MOL000098	quercetin	CDKN1A	NO
Pogostemon Cablin (	MOL000098	quercetin	EIF6	NO
Pogostemon Cablin (	MOL000098	quercetin	BAX	NO
Pogostemon Cablin (	MOL000098	quercetin	CASP9	YES
Pogostemon Cablin (	MOL000098	quercetin	PLAU	NO
Pogostemon Cablin (	MOL000098	quercetin	MMP2	YES
Pogostemon Cablin (	MOL000098	quercetin	MMP9	YES
Pogostemon Cablin (	MOL000098	quercetin	MAPK1	YES
Pogostemon Cablin (	MOL000098	quercetin	IL10RB	NO
Pogostemon Cablin (	MOL000098	quercetin	RB1	NO
Pogostemon Cablin (	MOL000098	quercetin	TNF	YES
Pogostemon Cablin (	MOL000098	quercetin	JUN	YES
Pogostemon Cablin (	MOL000098	quercetin	IL6	YES
Pogostemon Cablin (	MOL000098	quercetin	AHSA1	NO
Pogostemon Cablin (	MOL000098	quercetin	CASP3	YES
Pogostemon Cablin (	MOL000098	quercetin	TP53	YES
Pogostemon Cablin (	MOL000098	quercetin	ELK1	NO
Pogostemon Cablin (	MOL000098	quercetin	NFKBIA	YES
Pogostemon Cablin (	MOL000098	quercetin	ODC1	NO
Pogostemon Cablin (	MOL000098	quercetin	CASP8	NO
Pogostemon Cablin (	MOL000098	quercetin	TOP1	NO
Pogostemon Cablin (	MOL000098	quercetin	RAF1	YES
Pogostemon Cablin (	MOL000098	quercetin	SOD1	YES
Pogostemon Cablin (	MOL000098	quercetin	PRKCA	NO
Pogostemon Cablin (	MOL000098	quercetin	MMP1	NO
Pogostemon Cablin (	MOL000098	quercetin	HIF1A	NO
Pogostemon Cablin (	MOL000098	quercetin	STAT1	YES
Pogostemon Cablin (	MOL000098	quercetin	RUNX1T1	NO
Pogostemon Cablin (	MOL000098	quercetin	CDK1	NO
Pogostemon Cablin (	MOL000098	quercetin	HSPA5	YES
Pogostemon Cablin (	MOL000098	quercetin	ERBB2	YES
Pogostemon Cablin (	MOL000098	quercetin	PPARG	NO
Pogostemon Cablin (	MOL000098	quercetin	ACACA	NO
Pogostemon Cablin (	MOL000098	quercetin	HMOX1	YES
Pogostemon Cablin (	MOL000098	quercetin	CYP3A4	YES
Pogostemon Cablin (	MOL000098	quercetin	CAV1	YES
Pogostemon Cablin (	MOL000098	quercetin	MYC	NO
Pogostemon Cablin (	MOL000098	quercetin	F3	YES
Pogostemon Cablin (	MOL000098	quercetin	GJA1	NO
Pogostemon Cablin (	MOL000098	quercetin	CYP1A1	NO
Pogostemon Cablin (	MOL000098	quercetin	ICAM1	YES
Pogostemon Cablin (	MOL000098	quercetin	IL1B	YES
Pogostemon Cablin (	MOL000098	quercetin	CCL2	YES
Pogostemon Cablin (	MOL000098	quercetin	SELE	YES
Pogostemon Cablin (	MOL000098	quercetin	VCAM1	YES
Pogostemon Cablin (	MOL000098	quercetin	CXCL8	YES
Pogostemon Cablin (	MOL000098	quercetin	PRKCB	NO
Pogostemon Cablin (	MOL000098	quercetin	BIRC5	NO
Pogostemon Cablin (	MOL000098	quercetin	DUOX2	NO
Pogostemon Cablin (	MOL000098	quercetin	NOS3	YES
Pogostemon Cablin (	MOL000098	quercetin	HSPB1	NO
Pogostemon Cablin (	MOL000098	quercetin	IL2RB	YES

Pogostemon Cablin (	MOL000098	quercetin	NR1I2	NO
Pogostemon Cablin (	MOL000098	quercetin	CYP1B1	NO
Pogostemon Cablin (	MOL000098	quercetin	CCNB1	NO
Pogostemon Cablin (	MOL000098	quercetin	PLAT	YES
Pogostemon Cablin (	MOL000098	quercetin	THBD	YES
Pogostemon Cablin (	MOL000098	quercetin	SERPINE1	YES
Pogostemon Cablin (	MOL000098	quercetin	IFNG	YES
Pogostemon Cablin (	MOL000098	quercetin	ALOX5	NO
Pogostemon Cablin (	MOL000098	quercetin	IL1A	YES
Pogostemon Cablin (	MOL000098	quercetin	MPO	NO
Pogostemon Cablin (	MOL000098	quercetin	TOP2A	NO
Pogostemon Cablin (	MOL000098	quercetin	NCF1	YES
Pogostemon Cablin (	MOL000098	quercetin	ABCG2	YES
Pogostemon Cablin (	MOL000098	quercetin	HAS2	NO
Pogostemon Cablin (	MOL000098	quercetin	NFE2L2	YES
Pogostemon Cablin (	MOL000098	quercetin	NQO1	NO
Pogostemon Cablin (	MOL000098	quercetin	PARP1	YES
Pogostemon Cablin (	MOL000098	quercetin	AHR	YES
Pogostemon Cablin (	MOL000098	quercetin	PSMD3	NO
Pogostemon Cablin (	MOL000098	quercetin	SLC2A4	NO
Pogostemon Cablin (	MOL000098	quercetin	COL3A1	NO
Pogostemon Cablin (	MOL000098	quercetin	CXCL11	YES
Pogostemon Cablin (	MOL000098	quercetin	CXCL2	NO
Pogostemon Cablin (	MOL000098	quercetin	DCAF5	NO
Pogostemon Cablin (	MOL000098	quercetin	NR1I3	NO
Pogostemon Cablin (	MOL000098	quercetin	CHEK2	NO
Pogostemon Cablin (	MOL000098	quercetin	INSR	YES
Pogostemon Cablin (	MOL000098	quercetin	CLDN4	NO
Pogostemon Cablin (	MOL000098	quercetin	PPARA	NO
Pogostemon Cablin (	MOL000098	quercetin	PPARD	NO
Pogostemon Cablin (	MOL000098	quercetin	HSF1	YES
Pogostemon Cablin (	MOL000098	quercetin	CXCL10	YES
Pogostemon Cablin (	MOL000098	quercetin	CHUK	NO
Pogostemon Cablin (	MOL000098	quercetin	SPP1	YES
Pogostemon Cablin (	MOL000098	quercetin	RUNX2	NO
Pogostemon Cablin (	MOL000098	quercetin	RASSF1	YES
Pogostemon Cablin (	MOL000098	quercetin	E2F1	NO
Pogostemon Cablin (	MOL000098	quercetin	E2F2	NO
Pogostemon Cablin (	MOL000098	quercetin	ACP3	NO
Pogostemon Cablin (	MOL000098	quercetin	CTSD	NO
Pogostemon Cablin (	MOL000098	quercetin	IGFBP3	NO
Pogostemon Cablin (	MOL000098	quercetin	IGF2	YES
Pogostemon Cablin (	MOL000098	quercetin	CD40LG	YES
Pogostemon Cablin (	MOL000098	quercetin	IRF1	YES
Pogostemon Cablin (	MOL000098	quercetin	ERBB3	NO
Pogostemon Cablin (	MOL000098	quercetin	PON1	NO
Pogostemon Cablin (	MOL000098	quercetin	DIO1	NO
Pogostemon Cablin (	MOL000098	quercetin	PCOLCE	NO
Pogostemon Cablin (	MOL000098	quercetin	NPEPPS	NO
Pogostemon Cablin (	MOL000098	quercetin	HK2	YES
Pogostemon Cablin (	MOL000098	quercetin	RASA1	NO
Pogostemon Cablin (	MOL000098	quercetin	GSTM1	NO
Pogostemon Cablin (	MOL000098	quercetin	GSTM2	NO
Pogostemon Cablin (	MOL000098	quercetin	PTGS1	NO

Pogostemon Cablin (	MOL000098	quercetin	AR	YES
Pogostemon Cablin (	MOL000098	quercetin	PPARG	NO
Pogostemon Cablin (	MOL000098	quercetin	PTGS2	YES
Pogostemon Cablin (	MOL000098	quercetin	HSP90AA1	YES
Pogostemon Cablin (	MOL000098	quercetin	NCOA2	NO
Pogostemon Cablin (	MOL000098	quercetin	DPP4	NO
Pogostemon Cablin (	MOL000098	quercetin	AKR1B1	NO
Pogostemon Cablin (	MOL000098	quercetin	PRSS1	NO
Pogostemon Cablin (	MOL000098	quercetin	TOP2A	NO
Pogostemon Cablin (	MOL000098	quercetin	F2	YES
Pogostemon Cablin (	MOL000098	quercetin	KCNH2	NO
Pogostemon Cablin (	MOL000098	quercetin	SCN5A	NO
Pogostemon Cablin (	MOL000098	quercetin	F10	YES
Pogostemon Cablin (	MOL000098	quercetin	ADRB2	NO
Pogostemon Cablin (	MOL000098	quercetin	MMP3	YES
Pogostemon Cablin (	MOL000098	quercetin	PRKACA	NO
Pogostemon Cablin (	MOL000098	quercetin	F7	NO
Pogostemon Cablin (	MOL000098	quercetin	RXRA	NO
Pogostemon Cablin (	MOL000098	quercetin	ACHE	NO
Pogostemon Cablin (	MOL000098	quercetin	MAOB	NO
Pogostemon Cablin (	MOL000098	quercetin	RELA	YES
Pogostemon Cablin (	MOL000098	quercetin	EGFR	YES
Pogostemon Cablin (	MOL000098	quercetin	AKT1	YES
Pogostemon Cablin (	MOL000098	quercetin	METTL1	NO
Pogostemon Cablin (	MOL000098	quercetin	CCND1	NO
Pogostemon Cablin (	MOL000098	quercetin	BCL2	YES
Pogostemon Cablin (	MOL000098	quercetin	BCL2L1	YES
Pogostemon Cablin (	MOL000098	quercetin	FOS	NO
Pogostemon Cablin (	MOL000098	quercetin	CDKN1A	NO
Pogostemon Cablin (	MOL000098	quercetin	EIF6	NO
Pogostemon Cablin (	MOL000098	quercetin	BAX	NO
Pogostemon Cablin (	MOL000098	quercetin	CASP9	YES
Pogostemon Cablin (	MOL000098	quercetin	PLAU	NO
Pogostemon Cablin (	MOL000098	quercetin	MMP2	YES
Pogostemon Cablin (	MOL000098	quercetin	MMP9	YES
Pogostemon Cablin (	MOL000098	quercetin	MAPK1	YES
Pogostemon Cablin (	MOL000098	quercetin	IL10RB	NO
Pogostemon Cablin (	MOL000098	quercetin	RB1	NO
Pogostemon Cablin (	MOL000098	quercetin	TNF	YES
Pogostemon Cablin (	MOL000098	quercetin	JUN	YES
Pogostemon Cablin (	MOL000098	quercetin	IL6	YES
Pogostemon Cablin (	MOL000098	quercetin	AHSA1	NO
Pogostemon Cablin (	MOL000098	quercetin	CASP3	YES
Pogostemon Cablin (	MOL000098	quercetin	TP53	YES
Pogostemon Cablin (	MOL000098	quercetin	ELK1	NO
Pogostemon Cablin (	MOL000098	quercetin	NFKBIA	YES
Pogostemon Cablin (	MOL000098	quercetin	ODC1	NO
Pogostemon Cablin (	MOL000098	quercetin	CASP8	NO
Pogostemon Cablin (	MOL000098	quercetin	TOP1	NO
Pogostemon Cablin (	MOL000098	quercetin	RAF1	YES
Pogostemon Cablin (	MOL000098	quercetin	SOD1	YES
Pogostemon Cablin (	MOL000098	quercetin	PRKCA	NO
Pogostemon Cablin (	MOL000098	quercetin	MMP1	NO
Pogostemon Cablin (	MOL000098	quercetin	HIF1A	NO

Pogostemon Cablin (	MOL000098	quercetin	STAT1	YES
Pogostemon Cablin (	MOL000098	quercetin	RUNX1T1	NO
Pogostemon Cablin (	MOL000098	quercetin	CDK1	NO
Pogostemon Cablin (	MOL000098	quercetin	HSPA5	YES
Pogostemon Cablin (	MOL000098	quercetin	ERBB2	YES
Pogostemon Cablin (	MOL000098	quercetin	PPARG	NO
Pogostemon Cablin (	MOL000098	quercetin	ACACA	NO
Pogostemon Cablin (	MOL000098	quercetin	HMOX1	YES
Pogostemon Cablin (	MOL000098	quercetin	CYP3A4	YES
Pogostemon Cablin (	MOL000098	quercetin	CAV1	YES
Pogostemon Cablin (	MOL000098	quercetin	MYC	NO
Pogostemon Cablin (	MOL000098	quercetin	F3	YES
Pogostemon Cablin (	MOL000098	quercetin	GJA1	NO
Pogostemon Cablin (	MOL000098	quercetin	CYP1A1	NO
Pogostemon Cablin (	MOL000098	quercetin	ICAM1	YES
Pogostemon Cablin (	MOL000098	quercetin	IL1B	YES
Pogostemon Cablin (	MOL000098	quercetin	CCL2	YES
Pogostemon Cablin (	MOL000098	quercetin	SELE	YES
Pogostemon Cablin (	MOL000098	quercetin	VCAM1	YES
Pogostemon Cablin (	MOL000098	quercetin	CXCL8	YES
Pogostemon Cablin (	MOL000098	quercetin	PRKCB	NO
Pogostemon Cablin (	MOL000098	quercetin	BIRC5	NO
Pogostemon Cablin (	MOL000098	quercetin	DUOX2	NO
Pogostemon Cablin (	MOL000098	quercetin	NOS3	YES
Pogostemon Cablin (	MOL000098	quercetin	HSPB1	NO
Pogostemon Cablin (	MOL000098	quercetin	IL2RB	YES
Pogostemon Cablin (	MOL000098	quercetin	NR1I2	NO
Pogostemon Cablin (	MOL000098	quercetin	CYP1B1	NO
Pogostemon Cablin (	MOL000098	quercetin	CCNB1	NO
Pogostemon Cablin (	MOL000098	quercetin	PLAT	YES
Pogostemon Cablin (	MOL000098	quercetin	THBD	YES
Pogostemon Cablin (	MOL000098	quercetin	SERPINE1	YES
Pogostemon Cablin (	MOL000098	quercetin	IFNG	YES
Pogostemon Cablin (	MOL000098	quercetin	ALOX5	NO
Pogostemon Cablin (	MOL000098	quercetin	IL1A	YES
Pogostemon Cablin (	MOL000098	quercetin	MPO	NO
Pogostemon Cablin (	MOL000098	quercetin	TOP2A	NO
Pogostemon Cablin (	MOL000098	quercetin	NCF1	YES
Pogostemon Cablin (	MOL000098	quercetin	ABCG2	YES
Pogostemon Cablin (	MOL000098	quercetin	HAS2	NO
Pogostemon Cablin (	MOL000098	quercetin	NFE2L2	YES
Pogostemon Cablin (	MOL000098	quercetin	NQO1	NO
Pogostemon Cablin (	MOL000098	quercetin	PARP1	YES
Pogostemon Cablin (	MOL000098	quercetin	AHR	YES
Pogostemon Cablin (	MOL000098	quercetin	PSMD3	NO
Pogostemon Cablin (	MOL000098	quercetin	SLC2A4	NO
Pogostemon Cablin (	MOL000098	quercetin	COL3A1	NO
Pogostemon Cablin (	MOL000098	quercetin	CXCL11	YES
Pogostemon Cablin (	MOL000098	quercetin	CXCL2	NO
Pogostemon Cablin (	MOL000098	quercetin	DCAF5	NO
Pogostemon Cablin (	MOL000098	quercetin	NR1I3	NO
Pogostemon Cablin (	MOL000098	quercetin	CHEK2	NO
Pogostemon Cablin (	MOL000098	quercetin	INSR	YES
Pogostemon Cablin (	MOL000098	quercetin	CLDN4	NO

Pogostemon Cablin (	MOL000098	quercetin	PPARA	NO
Pogostemon Cablin (	MOL000098	quercetin	PPARD	NO
Pogostemon Cablin (	MOL000098	quercetin	HSF1	YES
Pogostemon Cablin (	MOL000098	quercetin	CXCL10	YES
Pogostemon Cablin (	MOL000098	quercetin	CHUK	NO
Pogostemon Cablin (	MOL000098	quercetin	SPP1	YES
Pogostemon Cablin (	MOL000098	quercetin	RUNX2	NO
Pogostemon Cablin (	MOL000098	quercetin	RASSF1	YES
Pogostemon Cablin (	MOL000098	quercetin	E2F1	NO
Pogostemon Cablin (	MOL000098	quercetin	E2F2	NO
Pogostemon Cablin (	MOL000098	quercetin	ACP3	NO
Pogostemon Cablin (	MOL000098	quercetin	CTSD	NO
Pogostemon Cablin (	MOL000098	quercetin	IGFBP3	NO
Pogostemon Cablin (	MOL000098	quercetin	IGF2	YES
Pogostemon Cablin (	MOL000098	quercetin	CD40LG	YES
Pogostemon Cablin (	MOL000098	quercetin	IRF1	YES
Pogostemon Cablin (	MOL000098	quercetin	ERBB3	NO
Pogostemon Cablin (	MOL000098	quercetin	PON1	NO
Pogostemon Cablin (	MOL000098	quercetin	DIO1	NO
Pogostemon Cablin (	MOL000098	quercetin	PCOLCE	NO
Pogostemon Cablin (	MOL000098	quercetin	NPEPPS	NO
Pogostemon Cablin (	MOL000098	quercetin	HK2	YES
Pogostemon Cablin (	MOL000098	quercetin	RASA1	NO
Pogostemon Cablin (	MOL000098	quercetin	GSTM1	NO
Pogostemon Cablin (	MOL000098	quercetin	GSTM2	NO
Pogostemon Cablin (	MOL005921	quercetin 7-O-β-D-glucoside	PTGS1	NO
Pogostemon Cablin (	MOL005921	quercetin 7-O-β-D-glucoside	ESR1	YES
Pogostemon Cablin (	MOL005921	quercetin 7-O-β-D-glucoside	AR	YES
Pogostemon Cablin (	MOL005921	quercetin 7-O-β-D-glucoside	PPARG	NO
Pogostemon Cablin (	MOL005921	quercetin 7-O-β-D-glucoside	PTGS2	YES
Pogostemon Cablin (	MOL005921	quercetin 7-O-β-D-glucoside	CA2	NO
Pogostemon Cablin (	MOL005921	quercetin 7-O-β-D-glucoside	HSP90AA1	YES
Pogostemon Cablin (	MOL005921	quercetin 7-O-β-D-glucoside	DPEP1	NO
Pogostemon Cablin (	MOL005921	quercetin 7-O-β-D-glucoside	NCOA2	NO
licorice	MOL004961	Quercetin der.	NOS2	YES
licorice	MOL004961	Quercetin der.	PTGS1	NO
licorice	MOL004961	Quercetin der.	ESR1	YES
licorice	MOL004961	Quercetin der.	AR	YES
licorice	MOL004961	Quercetin der.	SCN5A	NO
licorice	MOL004961	Quercetin der.	PPARG	NO
licorice	MOL004961	Quercetin der.	PTGS2	YES
licorice	MOL004961	Quercetin der.	PTPN1	YES
licorice	MOL004961	Quercetin der.	ESR2	YES
licorice	MOL004961	Quercetin der.	DPP4	NO
licorice	MOL004961	Quercetin der.	MAPK14	YES
licorice	MOL004961	Quercetin der.	GSK3B	YES
licorice	MOL004961	Quercetin der.	HSP90AA1	YES
licorice	MOL004961	Quercetin der.	CDK2	NO
licorice	MOL004961	Quercetin der.	PRSS1	NO
licorice	MOL004961	Quercetin der.	NCOA2	NO
licorice	MOL004961	Quercetin der.	CALM3	NO
Scutellariae Radix	MOL012266	rivularin	NOS2	YES
Scutellariae Radix	MOL012266	rivularin	PTGS1	NO
Scutellariae Radix	MOL012266	rivularin	F2	YES

Scutellariae Radix	MOL012266	rivularin	KCNH2	NO
Scutellariae Radix	MOL012266	rivularin	AR	YES
Scutellariae Radix	MOL012266	rivularin	SCN5A	NO
Scutellariae Radix	MOL012266	rivularin	F10	YES
Scutellariae Radix	MOL012266	rivularin	PTGS2	YES
Scutellariae Radix	MOL012266	rivularin	CA2	NO
Scutellariae Radix	MOL012266	rivularin	F7	NO
Scutellariae Radix	MOL012266	rivularin	KDR	YES
Scutellariae Radix	MOL012266	rivularin	RXRA	NO
Scutellariae Radix	MOL012266	rivularin	TOP2A	NO
Scutellariae Radix	MOL012266	rivularin	ESR2	YES
Scutellariae Radix	MOL012266	rivularin	DPP4	NO
Scutellariae Radix	MOL012266	rivularin	HSP90AA1	YES
Scutellariae Radix	MOL012266	rivularin	PRSS1	NO
Scutellariae Radix	MOL012266	rivularin	NCOA2	NO
Scutellariae Radix	MOL012266	rivularin	NCOA1	NO
Scutellariae Radix	MOL012266	rivularin	CALM3	NO
Scutellariae Radix	MOL002915	Salvigenin	NOS2	YES
Scutellariae Radix	MOL002915	Salvigenin	PTGS1	NO
Scutellariae Radix	MOL002915	Salvigenin	F2	YES
Scutellariae Radix	MOL002915	Salvigenin	SCN5A	NO
Scutellariae Radix	MOL002915	Salvigenin	F10	YES
Scutellariae Radix	MOL002915	Salvigenin	PTGS2	YES
Scutellariae Radix	MOL002915	Salvigenin	RXRA	NO
Scutellariae Radix	MOL002915	Salvigenin	ACHE	NO
Scutellariae Radix	MOL002915	Salvigenin	ADRA1B	NO
Scutellariae Radix	MOL002915	Salvigenin	ADRB2	NO
Scutellariae Radix	MOL002915	Salvigenin	DPP4	NO
Scutellariae Radix	MOL002915	Salvigenin	HSP90AA1	YES
Scutellariae Radix	MOL002915	Salvigenin	IGHG1	NO
Scutellariae Radix	MOL002915	Salvigenin	PRSS1	NO
Scutellariae Radix	MOL002915	Salvigenin	NCOA2	NO
Scutellariae Radix	MOL002915	Salvigenin	CALM3	NO
Scutellariae Radix	MOL002915	Salvigenin	F7	NO
licorice	MOL004827	Semilicoisoflavone B	NOS2	YES
licorice	MOL004827	Semilicoisoflavone B	F2	YES
licorice	MOL004827	Semilicoisoflavone B	ESR1	YES
licorice	MOL004827	Semilicoisoflavone B	AR	YES
licorice	MOL004827	Semilicoisoflavone B	SCN5A	NO
licorice	MOL004827	Semilicoisoflavone B	PPARG	NO
licorice	MOL004827	Semilicoisoflavone B	F10	YES
licorice	MOL004827	Semilicoisoflavone B	PTGS2	YES
licorice	MOL004827	Semilicoisoflavone B	F7	NO
licorice	MOL004827	Semilicoisoflavone B	ACHE	NO
licorice	MOL004827	Semilicoisoflavone B	TOP2A	NO
licorice	MOL004827	Semilicoisoflavone B	GSK3B	YES
licorice	MOL004827	Semilicoisoflavone B	HSP90AA1	YES
licorice	MOL004827	Semilicoisoflavone B	CDK2	NO
licorice	MOL004827	Semilicoisoflavone B	CHEK1	NO
licorice	MOL004827	Semilicoisoflavone B	PRSS1	NO
licorice	MOL004827	Semilicoisoflavone B	CALM3	NO
licorice	MOL004891	shinpterocarpin	NOS2	YES
licorice	MOL004891	shinpterocarpin	PTGS1	NO
licorice	MOL004891	shinpterocarpin	CHRM3	NO

licorice	MOL004891	shinpterocarpin	KCNH2	NO
licorice	MOL004891	shinpterocarpin	CHRM1	NO
licorice	MOL004891	shinpterocarpin	ESR1	YES
licorice	MOL004891	shinpterocarpin	AR	YES
licorice	MOL004891	shinpterocarpin	SCN5A	NO
licorice	MOL004891	shinpterocarpin	PPARG	NO
licorice	MOL004891	shinpterocarpin	PTGS2	YES
licorice	MOL004891	shinpterocarpin	RXRA	NO
licorice	MOL004891	shinpterocarpin	OPRD1	NO
licorice	MOL004891	shinpterocarpin	ADRA1B	NO
licorice	MOL004891	shinpterocarpin	ADRB2	NO
licorice	MOL004891	shinpterocarpin	ADRA1D	NO
licorice	MOL004891	shinpterocarpin	OPRM1	NO
licorice	MOL004891	shinpterocarpin	ESR2	YES
licorice	MOL004891	shinpterocarpin	MAPK14	YES
licorice	MOL004891	shinpterocarpin	GSK3B	YES
licorice	MOL004891	shinpterocarpin	CDK2	NO
licorice	MOL004891	shinpterocarpin	CHRNA7	NO
licorice	MOL004891	shinpterocarpin	PRKACA	NO
licorice	MOL004891	shinpterocarpin	METTL1	NO
licorice	MOL004891	shinpterocarpin	PRSS1	NO
licorice	MOL004891	shinpterocarpin	CCNA2	NO
licorice	MOL004891	shinpterocarpin	NCOA1	NO
licorice	MOL004891	shinpterocarpin	CALM3	NO
licorice	MOL004935	Sigmoidin-B	ESR1	YES
licorice	MOL004935	Sigmoidin-B	F10	YES
licorice	MOL004935	Sigmoidin-B	PTGS2	YES
licorice	MOL004935	Sigmoidin-B	KDR	YES
licorice	MOL004935	Sigmoidin-B	HSP90AA1	YES
licorice	MOL004935	Sigmoidin-B	CALM3	NO
Scutellariae Radix	MOL000359	sitosterol	PGR	NO
Scutellariae Radix	MOL000359	sitosterol	NCOA2	NO
Scutellariae Radix	MOL000359	sitosterol	NR3C2	NO
Scutellariae Radix	MOL000359	sitosterol	PGR	NO
Scutellariae Radix	MOL000359	sitosterol	NCOA2	NO
Scutellariae Radix	MOL000359	sitosterol	NR3C2	NO
Scutellariae Radix	MOL000359	sitosterol	PGR	NO
Scutellariae Radix	MOL000359	sitosterol	NCOA2	NO
Scutellariae Radix	MOL000359	sitosterol	NR3C2	NO
Coicis Semen	MOL001323	Sitosterol alpha1	PGR	NO
Coicis Semen	MOL001323	Sitosterol alpha1	PTGS2	YES
Coicis Semen	MOL001323	Sitosterol alpha1	METTL1	NO
Coicis Semen	MOL001323	Sitosterol alpha1	NR3C2	NO
Scutellariae Radix	MOL002927	Skullcapflavone II	NOS2	YES
Scutellariae Radix	MOL002927	Skullcapflavone II	PTGS1	NO
Scutellariae Radix	MOL002927	Skullcapflavone II	F2	YES
Scutellariae Radix	MOL002927	Skullcapflavone II	KCNH2	NO
Scutellariae Radix	MOL002927	Skullcapflavone II	AR	YES
Scutellariae Radix	MOL002927	Skullcapflavone II	SCN5A	NO
Scutellariae Radix	MOL002927	Skullcapflavone II	F10	YES
Scutellariae Radix	MOL002927	Skullcapflavone II	PTGS2	YES
Scutellariae Radix	MOL002927	Skullcapflavone II	F7	NO
Scutellariae Radix	MOL002927	Skullcapflavone II	KDR	YES
Scutellariae Radix	MOL002927	Skullcapflavone II	CACNA2D1	NO

Scutellariae Radix	MOL002927	Skullcapflavone II	TOP2A	NO
Scutellariae Radix	MOL002927	Skullcapflavone II	DPP4	NO
Scutellariae Radix	MOL002927	Skullcapflavone II	HSP90AA1	YES
Scutellariae Radix	MOL002927	Skullcapflavone II	IGHG1	NO
Scutellariae Radix	MOL002927	Skullcapflavone II	PRSS1	NO
Scutellariae Radix	MOL002927	Skullcapflavone II	NCOA2	NO
Scutellariae Radix	MOL002927	Skullcapflavone II	NCOA1	NO
Scutellariae Radix	MOL002927	Skullcapflavone II	CALM3	NO
Codonopsis Radix	MOL004355	Spinasterol	PGR	NO
Codonopsis Radix	MOL004355	Spinasterol	NR3C2	NO
Codonopsis Radix	MOL004355	Spinasterol	NCOA2	NO
Codonopsis Radix	MOL006774	stigmast-7-enol	PGR	NO
Codonopsis Radix	MOL006774	stigmast-7-enol	NCOA2	NO
Scutellariae Radix	MOL000449	Stigmasterol	PGR	NO
Scutellariae Radix	MOL000449	Stigmasterol	NR3C2	NO
Scutellariae Radix	MOL000449	Stigmasterol	NCOA2	NO
Scutellariae Radix	MOL000449	Stigmasterol	METTL1	NO
Scutellariae Radix	MOL000449	Stigmasterol	IGHG1	NO
Scutellariae Radix	MOL000449	Stigmasterol	RXRA	NO
Scutellariae Radix	MOL000449	Stigmasterol	NCOA1	NO
Scutellariae Radix	MOL000449	Stigmasterol	PTGS1	NO
Scutellariae Radix	MOL000449	Stigmasterol	PTGS2	YES
Scutellariae Radix	MOL000449	Stigmasterol	ADRA2A	NO
Scutellariae Radix	MOL000449	Stigmasterol	SLC6A2	NO
Scutellariae Radix	MOL000449	Stigmasterol	SLC6A3	NO
Scutellariae Radix	MOL000449	Stigmasterol	ADRB2	NO
Scutellariae Radix	MOL000449	Stigmasterol	AKR1B1	NO
Scutellariae Radix	MOL000449	Stigmasterol	PLAU	NO
Scutellariae Radix	MOL000449	Stigmasterol	LTA4H	NO
Scutellariae Radix	MOL000449	Stigmasterol	MAOB	NO
Scutellariae Radix	MOL000449	Stigmasterol	MAOA	NO
Scutellariae Radix	MOL000449	Stigmasterol	PRKACA	NO
Scutellariae Radix	MOL000449	Stigmasterol	CTRB1	NO
Scutellariae Radix	MOL000449	Stigmasterol	CHRM3	NO
Scutellariae Radix	MOL000449	Stigmasterol	CHRM1	NO
Scutellariae Radix	MOL000449	Stigmasterol	ADRB1	NO
Scutellariae Radix	MOL000449	Stigmasterol	SCN5A	NO
Scutellariae Radix	MOL000449	Stigmasterol	ADRA1A	NO
Scutellariae Radix	MOL000449	Stigmasterol	CHRM2	NO
Scutellariae Radix	MOL000449	Stigmasterol	ADRA1B	NO
Scutellariae Radix	MOL000449	Stigmasterol	CHRNA7	NO
Scutellariae Radix	MOL000449	Stigmasterol	PGR	NO
Scutellariae Radix	MOL000449	Stigmasterol	NR3C2	NO
Scutellariae Radix	MOL000449	Stigmasterol	NCOA2	NO
Scutellariae Radix	MOL000449	Stigmasterol	METTL1	NO
Scutellariae Radix	MOL000449	Stigmasterol	IGHG1	NO
Scutellariae Radix	MOL000449	Stigmasterol	RXRA	NO
Scutellariae Radix	MOL000449	Stigmasterol	NCOA1	NO
Scutellariae Radix	MOL000449	Stigmasterol	PTGS1	NO
Scutellariae Radix	MOL000449	Stigmasterol	PTGS2	YES
Scutellariae Radix	MOL000449	Stigmasterol	ADRA2A	NO
Scutellariae Radix	MOL000449	Stigmasterol	SLC6A2	NO
Scutellariae Radix	MOL000449	Stigmasterol	SLC6A3	NO
Scutellariae Radix	MOL000449	Stigmasterol	ADRB2	NO

Scutellariae Radix	MOL000449	Stigmasterol	AKR1B1	NO
Scutellariae Radix	MOL000449	Stigmasterol	PLAU	NO
Scutellariae Radix	MOL000449	Stigmasterol	LTA4H	NO
Scutellariae Radix	MOL000449	Stigmasterol	MAOB	NO
Scutellariae Radix	MOL000449	Stigmasterol	MAOA	NO
Scutellariae Radix	MOL000449	Stigmasterol	PRKACA	NO
Scutellariae Radix	MOL000449	Stigmasterol	CTRB1	NO
Scutellariae Radix	MOL000449	Stigmasterol	CHRM3	NO
Scutellariae Radix	MOL000449	Stigmasterol	CHRM1	NO
Scutellariae Radix	MOL000449	Stigmasterol	ADRB1	NO
Scutellariae Radix	MOL000449	Stigmasterol	SCN5A	NO
Scutellariae Radix	MOL000449	Stigmasterol	ADRA1A	NO
Scutellariae Radix	MOL000449	Stigmasterol	CHRM2	NO
Scutellariae Radix	MOL000449	Stigmasterol	ADRA1B	NO
Scutellariae Radix	MOL000449	Stigmasterol	CHRNA7	NO
Scutellariae Radix	MOL000449	Stigmasterol	PGR	NO
Scutellariae Radix	MOL000449	Stigmasterol	NR3C2	NO
Scutellariae Radix	MOL000449	Stigmasterol	NCOA2	NO
Scutellariae Radix	MOL000449	Stigmasterol	METTL1	NO
Scutellariae Radix	MOL000449	Stigmasterol	IGHG1	NO
Scutellariae Radix	MOL000449	Stigmasterol	RXRA	NO
Scutellariae Radix	MOL000449	Stigmasterol	NCOA1	NO
Scutellariae Radix	MOL000449	Stigmasterol	PTGS1	NO
Scutellariae Radix	MOL000449	Stigmasterol	PTGS2	YES
Scutellariae Radix	MOL000449	Stigmasterol	ADRA2A	NO
Scutellariae Radix	MOL000449	Stigmasterol	SLC6A2	NO
Scutellariae Radix	MOL000449	Stigmasterol	SLC6A3	NO
Scutellariae Radix	MOL000449	Stigmasterol	ADRB2	NO
Scutellariae Radix	MOL000449	Stigmasterol	AKR1B1	NO
Scutellariae Radix	MOL000449	Stigmasterol	PLAU	NO
Scutellariae Radix	MOL000449	Stigmasterol	LTA4H	NO
Scutellariae Radix	MOL000449	Stigmasterol	MAOB	NO
Scutellariae Radix	MOL000449	Stigmasterol	MAOA	NO
Scutellariae Radix	MOL000449	Stigmasterol	PRKACA	NO
Scutellariae Radix	MOL000449	Stigmasterol	CTRB1	NO
Scutellariae Radix	MOL000449	Stigmasterol	CHRM3	NO
Scutellariae Radix	MOL000449	Stigmasterol	CHRM1	NO
Scutellariae Radix	MOL000449	Stigmasterol	ADRB1	NO
Scutellariae Radix	MOL000449	Stigmasterol	SCN5A	NO
Scutellariae Radix	MOL000449	Stigmasterol	ADRA1A	NO
Scutellariae Radix	MOL000449	Stigmasterol	CHRM2	NO
Scutellariae Radix	MOL000449	Stigmasterol	ADRA1B	NO
Scutellariae Radix	MOL000449	Stigmasterol	CHRNA7	NO
Scutellariae Radix	MOL000449	Stigmasterol	PGR	NO
Scutellariae Radix	MOL000449	Stigmasterol	NR3C2	NO
Scutellariae Radix	MOL000449	Stigmasterol	NCOA2	NO
Scutellariae Radix	MOL000449	Stigmasterol	METTL1	NO
Scutellariae Radix	MOL000449	Stigmasterol	IGHG1	NO
Scutellariae Radix	MOL000449	Stigmasterol	RXRA	NO
Scutellariae Radix	MOL000449	Stigmasterol	NCOA1	NO
Scutellariae Radix	MOL000449	Stigmasterol	PTGS1	NO
Scutellariae Radix	MOL000449	Stigmasterol	PTGS2	YES
Scutellariae Radix	MOL000449	Stigmasterol	ADRA2A	NO
Scutellariae Radix	MOL000449	Stigmasterol	SLC6A2	NO

Scutellariae Radix	MOL000449	Stigmasterol	SLC6A3	NO
Scutellariae Radix	MOL000449	Stigmasterol	ADRB2	NO
Scutellariae Radix	MOL000449	Stigmasterol	AKR1B1	NO
Scutellariae Radix	MOL000449	Stigmasterol	PLAU	NO
Scutellariae Radix	MOL000449	Stigmasterol	LTA4H	NO
Scutellariae Radix	MOL000449	Stigmasterol	MAOB	NO
Scutellariae Radix	MOL000449	Stigmasterol	MAOA	NO
Scutellariae Radix	MOL000449	Stigmasterol	PRKACA	NO
Scutellariae Radix	MOL000449	Stigmasterol	CTRB1	NO
Scutellariae Radix	MOL000449	Stigmasterol	CHRM3	NO
Scutellariae Radix	MOL000449	Stigmasterol	CHRM1	NO
Scutellariae Radix	MOL000449	Stigmasterol	ADRB1	NO
Scutellariae Radix	MOL000449	Stigmasterol	SCN5A	NO
Scutellariae Radix	MOL000449	Stigmasterol	ADRA1A	NO
Scutellariae Radix	MOL000449	Stigmasterol	CHRM2	NO
Scutellariae Radix	MOL000449	Stigmasterol	ADRA1B	NO
Scutellariae Radix	MOL000449	Stigmasterol	CHRNA7	NO
licorice	MOL000500	Vestitol	NOS2	YES
licorice	MOL000500	Vestitol	PTGS1	NO
licorice	MOL000500	Vestitol	CHRM1	NO
licorice	MOL000500	Vestitol	ESR1	YES
licorice	MOL000500	Vestitol	AR	YES
licorice	MOL000500	Vestitol	SCN5A	NO
licorice	MOL000500	Vestitol	PPARG	NO
licorice	MOL000500	Vestitol	PTGS2	YES
licorice	MOL000500	Vestitol	CHRM4	NO
licorice	MOL000500	Vestitol	RXRA	NO
licorice	MOL000500	Vestitol	PDE3A	NO
licorice	MOL000500	Vestitol	ADRA1A	NO
licorice	MOL000500	Vestitol	ADRA1B	NO
licorice	MOL000500	Vestitol	SLC6A3	NO
licorice	MOL000500	Vestitol	ADRB2	NO
licorice	MOL000500	Vestitol	SLC6A4	YES
licorice	MOL000500	Vestitol	ESR2	YES
licorice	MOL000500	Vestitol	DPP4	NO
licorice	MOL000500	Vestitol	MAPK14	YES
licorice	MOL000500	Vestitol	GSK3B	YES
licorice	MOL000500	Vestitol	HSP90AA1	YES
licorice	MOL000500	Vestitol	CDK2	NO
licorice	MOL000500	Vestitol	CHEK1	NO
licorice	MOL000500	Vestitol	PRKACA	NO
licorice	MOL000500	Vestitol	PRSS1	NO
licorice	MOL000500	Vestitol	CCNA2	NO
licorice	MOL000500	Vestitol	PKIA	NO
licorice	MOL000500	Vestitol	CALM3	NO
Scutellariae Radix	MOL000173	wogonin	NOS2	YES
Scutellariae Radix	MOL000173	wogonin	PTGS1	NO
Scutellariae Radix	MOL000173	wogonin	ESR1	YES
Scutellariae Radix	MOL000173	wogonin	AR	YES
Scutellariae Radix	MOL000173	wogonin	SCN5A	NO
Scutellariae Radix	MOL000173	wogonin	PPARG	NO
Scutellariae Radix	MOL000173	wogonin	PTGS2	YES
Scutellariae Radix	MOL000173	wogonin	RXRA	NO
Scutellariae Radix	MOL000173	wogonin	PDE3A	NO

Scutellariae Radix	MOL000173	wogonin	DPP4	NO
Scutellariae Radix	MOL000173	wogonin	MAPK14	YES
Scutellariae Radix	MOL000173	wogonin	GSK3B	YES
Scutellariae Radix	MOL000173	wogonin	HSP90AA1	YES
Scutellariae Radix	MOL000173	wogonin	CDK2	NO
Scutellariae Radix	MOL000173	wogonin	CHEK1	NO
Scutellariae Radix	MOL000173	wogonin	PRKACA	NO
Scutellariae Radix	MOL000173	wogonin	PRSS1	NO
Scutellariae Radix	MOL000173	wogonin	CALM3	NO
Scutellariae Radix	MOL000173	wogonin	ADRB2	NO
Scutellariae Radix	MOL000173	wogonin	RELA	YES
Scutellariae Radix	MOL000173	wogonin	AKT1	YES
Scutellariae Radix	MOL000173	wogonin	CCND1	NO
Scutellariae Radix	MOL000173	wogonin	BCL2	YES
Scutellariae Radix	MOL000173	wogonin	CDKN1A	NO
Scutellariae Radix	MOL000173	wogonin	EIF6	NO
Scutellariae Radix	MOL000173	wogonin	BAX	NO
Scutellariae Radix	MOL000173	wogonin	CASP9	YES
Scutellariae Radix	MOL000173	wogonin	KDR	YES
Scutellariae Radix	MOL000173	wogonin	TNF	YES
Scutellariae Radix	MOL000173	wogonin	JUN	YES
Scutellariae Radix	MOL000173	wogonin	IL6	YES
Scutellariae Radix	MOL000173	wogonin	AHSA1	NO
Scutellariae Radix	MOL000173	wogonin	CASP3	YES
Scutellariae Radix	MOL000173	wogonin	TP53	YES
Scutellariae Radix	MOL000173	wogonin	TEP1	NO
Scutellariae Radix	MOL000173	wogonin	MMP1	NO
Scutellariae Radix	MOL000173	wogonin	CCL2	YES
Scutellariae Radix	MOL000173	wogonin	PRKCD	NO
Scutellariae Radix	MOL000173	wogonin	FNDC3B	NO
Scutellariae Radix	MOL000173	wogonin	CXCL8	YES
Scutellariae Radix	MOL000173	wogonin	MCL1	NO
licorice	MOL005018	Xambioona	NOS2	YES
licorice	MOL005018	Xambioona	ESR1	YES
licorice	MOL005018	Xambioona	F10	YES
licorice	MOL005018	Xambioona	PTGS2	YES
licorice	MOL005018	Xambioona	ESR2	YES
licorice	MOL005018	Xambioona	NCOA2	NO
licorice	MOL005018	Xambioona	CALM3	NO
Codonopsis Radix	MOL003036	ZINC03978781	PGR	NO
Codonopsis Radix	MOL003036	ZINC03978781	NCOA2	NO
Codonopsis Radix	MOL003036	ZINC03978781	NR3C2	NO
Radix Bupleuri	MOL004718	α-spinasterol	PGR	NO
Radix Bupleuri	MOL004718	α-spinasterol	NR3C2	NO
Radix Bupleuri	MOL004718	α-spinasterol	NCOA2	NO
Florisil	MOL100010	Alumina	PHF20	NO
Florisil	MOL100010	Alumina	DESI2	NO
Gypsum	MOL100002	Calcium sulfate dihydrate	TUBGCP6	NO
Gypsum	MOL100003	Calcium sulfate hemihydrate	TUBGCP6	NO
Gypsum	MOL100002	Calcium sulfate dihydrate	TUBGCP4	NO
Gypsum	MOL100003	Calcium sulfate hemihydrate	TUBGCP4	NO
Gypsum	MOL100002	Calcium sulfate dihydrate	TUBGCP3	NO
Gypsum	MOL100003	Calcium sulfate hemihydrate	TUBGCP3	NO
Gypsum	MOL100001	Calcium sulfate	AS3MT	NO

Bubalus Bubalis	MOL100004	Arginine	AZIN2	NO
Bubalus Bubalis	MOL100004	Arginine	DDAH1	NO
Bubalus Bubalis	MOL100004	Arginine	SLC7A4	NO
Bubalus Bubalis	MOL100004	Arginine	NOS1	NO
Bubalus Bubalis	MOL100004	Arginine	ASL	NO
Bubalus Bubalis	MOL100004	Arginine	SLC7A3	NO
Bubalus Bubalis	MOL100004	Arginine	PADI4	YES
Bubalus Bubalis	MOL100004	Arginine	PADI6	NO
Bubalus Bubalis	MOL100004	Arginine	PADI3	NO
Bubalus Bubalis	MOL100004	Arginine	ASS1	YES
Bubalus Bubalis	MOL100004	Arginine	NOS3	YES
Bubalus Bubalis	MOL100004	Arginine	PADI1	NO
Bubalus Bubalis	MOL100004	Arginine	PADI2	NO
Bubalus Bubalis	MOL100004	Arginine	SLC7A1	NO
Bubalus Bubalis	MOL100004	Arginine	OTC	NO
Bubalus Bubalis	MOL100004	Arginine	NOS2	YES
Bubalus Bubalis	MOL100004	Arginine	DDAH2	NO
Bubalus Bubalis	MOL100004	Arginine	ARG2	NO
Bubalus Bubalis	MOL100005	Aspartic Acid	GOT2	NO
Bubalus Bubalis	MOL100005	Aspartic Acid	ASNS	NO
Bubalus Bubalis	MOL100005	Aspartic Acid	DARS	NO
Bubalus Bubalis	MOL100005	Aspartic Acid	CAD	NO
Bubalus Bubalis	MOL100005	Aspartic Acid	SUCLG2	NO
Bubalus Bubalis	MOL100005	Aspartic Acid	GABRB1	NO
Bubalus Bubalis	MOL100005	Aspartic Acid	TMLHE	NO
Bubalus Bubalis	MOL100005	Aspartic Acid	PLOD1	NO
Bubalus Bubalis	MOL100005	Aspartic Acid	ALDH5A1	NO
Bubalus Bubalis	MOL100005	Aspartic Acid	TARS2	NO
Bubalus Bubalis	MOL100005	Aspartic Acid	ACADSB	NO
Bubalus Bubalis	MOL100005	Aspartic Acid	ALAD	NO
Bubalus Bubalis	MOL100005	Aspartic Acid	LCMT2	NO
Bubalus Bubalis	MOL100005	Aspartic Acid	SLC1A1	NO
Bubalus Bubalis	MOL100005	Aspartic Acid	DARS2	NO
Bubalus Bubalis	MOL100005	Aspartic Acid	ASRGL1	NO
Bubalus Bubalis	MOL100005	Aspartic Acid	ACY1	NO
Bubalus Bubalis	MOL100005	Aspartic Acid	SLC13A2	NO
Bubalus Bubalis	MOL100005	Aspartic Acid	SLC25A10	NO
Bubalus Bubalis	MOL100005	Aspartic Acid	BCAT1	NO
Bubalus Bubalis	MOL100005	Aspartic Acid	THNSL1	NO
Bubalus Bubalis	MOL100005	Aspartic Acid	BBOX1	NO
Bubalus Bubalis	MOL100005	Aspartic Acid	LARS2	NO
Bubalus Bubalis	MOL100005	Aspartic Acid	PCCB	NO
Bubalus Bubalis	MOL100005	Aspartic Acid	P4HA2	NO
Bubalus Bubalis	MOL100005	Aspartic Acid	P3H1	NO
Bubalus Bubalis	MOL100005	Aspartic Acid	ACY3	NO
Bubalus Bubalis	MOL100005	Aspartic Acid	GOT1	NO
Bubalus Bubalis	MOL100005	Aspartic Acid	RNASE1	YES
Bubalus Bubalis	MOL100005	Aspartic Acid	SLC25A13	NO
Bubalus Bubalis	MOL100005	Aspartic Acid	SLC13A1	NO
Bubalus Bubalis	MOL100005	Aspartic Acid	P3H3	NO
Bubalus Bubalis	MOL100005	Aspartic Acid	VARS	NO
Bubalus Bubalis	MOL100005	Aspartic Acid	SLC52A2	NO
Bubalus Bubalis	MOL100005	Aspartic Acid	SDHB	YES
Bubalus Bubalis	MOL100005	Aspartic Acid	PLOD3	NO

Bubalus Bubalis	MOL100005	Aspartic Acid	P3H2	NO
Bubalus Bubalis	MOL100005	Aspartic Acid	SUCLA2	NO
Bubalus Bubalis	MOL100005	Aspartic Acid	ASPA	NO
Bubalus Bubalis	MOL100005	Aspartic Acid	ASPH	NO
Bubalus Bubalis	MOL100005	Aspartic Acid	ASS1	YES
Bubalus Bubalis	MOL100005	Aspartic Acid	LYZ	NO
Bubalus Bubalis	MOL100005	Aspartic Acid	SUCLG1	NO
Bubalus Bubalis	MOL100005	Aspartic Acid	OXCT2	NO
Bubalus Bubalis	MOL100005	Aspartic Acid	TARS	NO
Bubalus Bubalis	MOL100005	Aspartic Acid	SUCNR1	NO
Bubalus Bubalis	MOL100005	Aspartic Acid	LARS	NO
Bubalus Bubalis	MOL100005	Aspartic Acid	P4HA1	NO
Bubalus Bubalis	MOL100005	Aspartic Acid	SDHA	YES
Bubalus Bubalis	MOL100005	Aspartic Acid	BCAT2	NO
Bubalus Bubalis	MOL100005	Aspartic Acid	ADSSL1	NO
Bubalus Bubalis	MOL100005	Aspartic Acid	SLC25A12	NO
Bubalus Bubalis	MOL100005	Aspartic Acid	ADSS	NO
Bubalus Bubalis	MOL100005	Aspartic Acid	PAICS	NO
Bubalus Bubalis	MOL100005	Aspartic Acid	HSD17B6	YES
Bubalus Bubalis	MOL100005	Aspartic Acid	IARS2	NO
Bubalus Bubalis	MOL100005	Aspartic Acid	LCMT1	NO
Bubalus Bubalis	MOL100005	Aspartic Acid	SLC13A3	NO
Bubalus Bubalis	MOL100005	Aspartic Acid	OXCT1	NO
Bubalus Bubalis	MOL100005	Aspartic Acid	SDHC	NO
Bubalus Bubalis	MOL100005	Aspartic Acid	IARS	NO
Bubalus Bubalis	MOL100005	Aspartic Acid	SDHD	NO
Bubalus Bubalis	MOL100006	Alanine	ABAT	NO
Bubalus Bubalis	MOL100006	Alanine	PHYKPL	NO
Bubalus Bubalis	MOL100006	Alanine	AGXT2	NO
Bubalus Bubalis	MOL100006	Alanine	SPTLC2	NO
Bubalus Bubalis	MOL100006	Alanine	GOT2	NO
Bubalus Bubalis	MOL100006	Alanine	SLC7A2	NO
Bubalus Bubalis	MOL100006	Alanine	IARS2	NO
Bubalus Bubalis	MOL100006	Alanine	SLC38A3	NO
Bubalus Bubalis	MOL100006	Alanine	GSS	NO
Bubalus Bubalis	MOL100006	Alanine	LCMT1	NO
Bubalus Bubalis	MOL100006	Alanine	GLYAT	NO
Bubalus Bubalis	MOL100006	Alanine	GLYATL1	NO
Bubalus Bubalis	MOL100006	Alanine	TNNC1	NO
Bubalus Bubalis	MOL100006	Alanine	VDAC2	NO
Bubalus Bubalis	MOL100006	Alanine	SHMT2	NO
Bubalus Bubalis	MOL100006	Alanine	ASRGL1	NO
Bubalus Bubalis	MOL100006	Alanine	LARS2	NO
Bubalus Bubalis	MOL100006	Alanine	ACADSB	NO
Bubalus Bubalis	MOL100006	Alanine	OTC	NO
Bubalus Bubalis	MOL100006	Alanine	ACY1	NO
Bubalus Bubalis	MOL100006	Alanine	PAICS	NO
Bubalus Bubalis	MOL100006	Alanine	ASPG	NO
Bubalus Bubalis	MOL100006	Alanine	ASNSD1	NO
Bubalus Bubalis	MOL100006	Alanine	HCN3	NO
Bubalus Bubalis	MOL100006	Alanine	TYW5	NO
Bubalus Bubalis	MOL100006	Alanine	CTPS2	NO
Bubalus Bubalis	MOL100006	Alanine	VARS2	NO
Bubalus Bubalis	MOL100006	Alanine	CSAD	NO

Bubalus Bubalis	MOL100006	Alanine	CARS	NO
Bubalus Bubalis	MOL100006	Alanine	SLC19A3	NO
Bubalus Bubalis	MOL100006	Alanine	CACNA1A	NO
Bubalus Bubalis	MOL100006	Alanine	DGKG	NO
Bubalus Bubalis	MOL100006	Alanine	MTR	NO
Bubalus Bubalis	MOL100006	Alanine	CACNA1B	NO
Bubalus Bubalis	MOL100006	Alanine	GRIN2B	NO
Bubalus Bubalis	MOL100006	Alanine	SMPD3	NO
Bubalus Bubalis	MOL100006	Alanine	GRIN2D	NO
Bubalus Bubalis	MOL100006	Alanine	SCARB1	NO
Bubalus Bubalis	MOL100006	Alanine	FBP1	NO
Bubalus Bubalis	MOL100006	Alanine	CACNA1H	NO
Bubalus Bubalis	MOL100006	Alanine	ARV1	NO
Bubalus Bubalis	MOL100006	Alanine	MUT	NO
Bubalus Bubalis	MOL100006	Alanine	AVPR1A	NO
Bubalus Bubalis	MOL100006	Alanine	HOGA1	NO
Bubalus Bubalis	MOL100006	Alanine	PRLR	NO
Bubalus Bubalis	MOL100006	Alanine	ZP4	NO
Bubalus Bubalis	MOL100006	Alanine	TK1	NO
Bubalus Bubalis	MOL100006	Alanine	PAOX	NO
Bubalus Bubalis	MOL100006	Alanine	RNASE4	NO
Bubalus Bubalis	MOL100006	Alanine	RCAN3	NO
Bubalus Bubalis	MOL100006	Alanine	PSPH	NO
Bubalus Bubalis	MOL100006	Alanine	ENOPH1	NO
Bubalus Bubalis	MOL100006	Alanine	SLC13A3	NO
Bubalus Bubalis	MOL100006	Alanine	ALDH18A1	NO
Bubalus Bubalis	MOL100006	Alanine	MOCS3	NO
Bubalus Bubalis	MOL100006	Alanine	BBOX1	NO
Bubalus Bubalis	MOL100006	Alanine	QPRT	NO
Bubalus Bubalis	MOL100006	Alanine	OPLAH	NO
Bubalus Bubalis	MOL100006	Alanine	AARSD1	NO
Bubalus Bubalis	MOL100006	Alanine	LEP	NO
Bubalus Bubalis	MOL100006	Alanine	SLIT3	NO
Bubalus Bubalis	MOL100006	Alanine	GAD1	NO
Bubalus Bubalis	MOL100006	Alanine	GLS	YES
Bubalus Bubalis	MOL100006	Alanine	AFMID	NO
Bubalus Bubalis	MOL100006	Alanine	PSAT1	NO
Bubalus Bubalis	MOL100006	Alanine	EARS2	NO
Bubalus Bubalis	MOL100006	Alanine	DLST	NO
Bubalus Bubalis	MOL100006	Alanine	NUDT3	NO
Bubalus Bubalis	MOL100006	Alanine	TNNT3	NO
Bubalus Bubalis	MOL100006	Alanine	LRRK2	NO
Bubalus Bubalis	MOL100006	Alanine	PI4K2A	NO
Bubalus Bubalis	MOL100006	Alanine	CTNS	NO
Bubalus Bubalis	MOL100006	Alanine	TMEM110	NO
Bubalus Bubalis	MOL100006	Alanine	KMO	NO
Bubalus Bubalis	MOL100006	Alanine	MMAA	NO
Bubalus Bubalis	MOL100006	Alanine	RNASE2	NO
Bubalus Bubalis	MOL100006	Alanine	PARK2	NO
Bubalus Bubalis	MOL100006	Alanine	HPRT1	NO
Bubalus Bubalis	MOL100006	Alanine	NARFL	NO
Bubalus Bubalis	MOL100006	Alanine	SLC25A4	NO
Bubalus Bubalis	MOL100006	Alanine	GPT2	NO
Bubalus Bubalis	MOL100006	Alanine	AARS2	NO

Bubalus Bubalis	MOL100006	Alanine	SLC36A1	NO
Bubalus Bubalis	MOL100006	Alanine	CBSL	NO
Bubalus Bubalis	MOL100006	Alanine	SLC1A5	NO
Bubalus Bubalis	MOL100006	Alanine	SLC1A1	NO
Bubalus Bubalis	MOL100006	Alanine	ASPA	NO
Bubalus Bubalis	MOL100006	Alanine	GCAT	NO
Bubalus Bubalis	MOL100006	Alanine	VDAC3	NO
Bubalus Bubalis	MOL100006	Alanine	GRIN3B	NO
Bubalus Bubalis	MOL100006	Alanine	GLRB	NO
Bubalus Bubalis	MOL100006	Alanine	SLC25A12	NO
Bubalus Bubalis	MOL100006	Alanine	NARS2	NO
Bubalus Bubalis	MOL100006	Alanine	GLDC	NO
Bubalus Bubalis	MOL100006	Alanine	GARS	NO
Bubalus Bubalis	MOL100006	Alanine	SLC7A3	NO
Bubalus Bubalis	MOL100006	Alanine	KARS	NO
Bubalus Bubalis	MOL100006	Alanine	SLC7A1	NO
Bubalus Bubalis	MOL100006	Alanine	PCCB	NO
Bubalus Bubalis	MOL100006	Alanine	BCAT2	NO
Bubalus Bubalis	MOL100006	Alanine	LCMT2	NO
Bubalus Bubalis	MOL100006	Alanine	PCCA	NO
Bubalus Bubalis	MOL100006	Alanine	TLR4	YES
Bubalus Bubalis	MOL100006	Alanine	TFAP2B	NO
Bubalus Bubalis	MOL100006	Alanine	AMT	NO
Bubalus Bubalis	MOL100006	Alanine	SLC38A7	NO
Bubalus Bubalis	MOL100006	Alanine	ACOT8	NO
Bubalus Bubalis	MOL100006	Alanine	TYMS	NO
Bubalus Bubalis	MOL100006	Alanine	THNSL1	NO
Bubalus Bubalis	MOL100006	Alanine	TARS2	NO
Bubalus Bubalis	MOL100006	Alanine	BHMT2	NO
Bubalus Bubalis	MOL100006	Alanine	CACNA2D2	NO
Bubalus Bubalis	MOL100006	Alanine	PTDSS1	NO
Bubalus Bubalis	MOL100006	Alanine	PAH	YES
Bubalus Bubalis	MOL100006	Alanine	DDAH1	NO
Bubalus Bubalis	MOL100006	Alanine	GRIN3A	NO
Bubalus Bubalis	MOL100006	Alanine	GSTP1	NO
Bubalus Bubalis	MOL100006	Alanine	PADI1	NO
Bubalus Bubalis	MOL100006	Alanine	ABCC2	NO
Bubalus Bubalis	MOL100006	Alanine	GPHN	NO
Bubalus Bubalis	MOL100006	Alanine	AUH	NO
Bubalus Bubalis	MOL100006	Alanine	GAA	NO
Bubalus Bubalis	MOL100006	Alanine	ADRB2	NO
Bubalus Bubalis	MOL100006	Alanine	NRXN1	NO
Bubalus Bubalis	MOL100006	Alanine	GART	NO
Bubalus Bubalis	MOL100006	Alanine	AADAT	NO
Bubalus Bubalis	MOL100006	Alanine	SLC7A10	NO
Bubalus Bubalis	MOL100006	Alanine	TOMM40	NO
Bubalus Bubalis	MOL100006	Alanine	NTSR1	NO
Bubalus Bubalis	MOL100006	Alanine	IL1B	YES
Bubalus Bubalis	MOL100006	Alanine	CYP11A1	NO
Bubalus Bubalis	MOL100006	Alanine	DHODH	NO
Bubalus Bubalis	MOL100006	Alanine	MYLK2	NO
Bubalus Bubalis	MOL100006	Alanine	MCCC1	NO
Bubalus Bubalis	MOL100006	Alanine	TNNI3	NO
Bubalus Bubalis	MOL100006	Alanine	MCCC2	NO

Bubalus Bubalis	MOL100006	Alanine	TAF7	NO
Bubalus Bubalis	MOL100006	Alanine	SLC19A2	NO
Bubalus Bubalis	MOL100006	Alanine	STIM1	NO
Bubalus Bubalis	MOL100006	Alanine	SLC25A5	YES
Bubalus Bubalis	MOL100006	Alanine	GLYATL3	NO
Bubalus Bubalis	MOL100006	Alanine	PRODH	NO
Bubalus Bubalis	MOL100006	Alanine	SLC6A13	NO
Bubalus Bubalis	MOL100006	Alanine	ANK3	NO
Bubalus Bubalis	MOL100006	Alanine	PCMT1	NO
Bubalus Bubalis	MOL100006	Alanine	EZR	NO
Bubalus Bubalis	MOL100006	Alanine	STAR	NO
Bubalus Bubalis	MOL100006	Alanine	CRACR2A	NO
Bubalus Bubalis	MOL100006	Alanine	UROS	NO
Bubalus Bubalis	MOL100006	Alanine	GLRA4	NO
Bubalus Bubalis	MOL100006	Alanine	SLC7A11	NO
Bubalus Bubalis	MOL100006	Alanine	SMOX	NO
Bubalus Bubalis	MOL100006	Alanine	GHR	NO
Bubalus Bubalis	MOL100006	Alanine	EGLN1	NO
Bubalus Bubalis	MOL100006	Alanine	PLCB1	NO
Bubalus Bubalis	MOL100006	Alanine	CERS1	YES
Bubalus Bubalis	MOL100006	Alanine	YARS2	NO
Bubalus Bubalis	MOL100006	Alanine	HAP1	YES
Bubalus Bubalis	MOL100006	Alanine	DTYMK	NO
Bubalus Bubalis	MOL100006	Alanine	ATP8A2	NO
Bubalus Bubalis	MOL100006	Alanine	SLC7A8	NO
Bubalus Bubalis	MOL100006	Alanine	GPT	YES
Bubalus Bubalis	MOL100006	Alanine	KYNU	NO
Bubalus Bubalis	MOL100006	Alanine	SARS	NO
Bubalus Bubalis	MOL100006	Alanine	BAAT	NO
Bubalus Bubalis	MOL100006	Alanine	GCSH	NO
Bubalus Bubalis	MOL100006	Alanine	GLRA2	NO
Bubalus Bubalis	MOL100006	Alanine	VARS	NO
Bubalus Bubalis	MOL100006	Alanine	ASNS	NO
Bubalus Bubalis	MOL100006	Alanine	GRIN2A	NO
Bubalus Bubalis	MOL100006	Alanine	ASPH	NO
Bubalus Bubalis	MOL100006	Alanine	GATM	NO
Bubalus Bubalis	MOL100006	Alanine	DARS	NO
Bubalus Bubalis	MOL100006	Alanine	OAZ2	NO
Bubalus Bubalis	MOL100006	Alanine	ALAS2	NO
Bubalus Bubalis	MOL100006	Alanine	GLYATL2	NO
Bubalus Bubalis	MOL100006	Alanine	RNASE1	YES
Bubalus Bubalis	MOL100006	Alanine	SLC6A5	NO
Bubalus Bubalis	MOL100006	Alanine	GLUL	NO
Bubalus Bubalis	MOL100006	Alanine	SLC25A13	NO
Bubalus Bubalis	MOL100006	Alanine	NARS	NO
Bubalus Bubalis	MOL100006	Alanine	AZIN2	NO
Bubalus Bubalis	MOL100006	Alanine	SLC26A6	NO
Bubalus Bubalis	MOL100006	Alanine	ALDH5A1	NO
Bubalus Bubalis	MOL100006	Alanine	HLCS	NO
Bubalus Bubalis	MOL100006	Alanine	HIF1AN	NO
Bubalus Bubalis	MOL100006	Alanine	TNNT2	NO
Bubalus Bubalis	MOL100006	Alanine	CDO1	NO
Bubalus Bubalis	MOL100006	Alanine	GCLM	NO
Bubalus Bubalis	MOL100006	Alanine	MGMT	NO

Bubalus Bubalis	MOL100006	Alanine	PADI3	NO
Bubalus Bubalis	MOL100006	Alanine	METAP2	NO
Bubalus Bubalis	MOL100006	Alanine	TAT	NO
Bubalus Bubalis	MOL100006	Alanine	FARSA	NO
Bubalus Bubalis	MOL100006	Alanine	PTDSS2	NO
Bubalus Bubalis	MOL100006	Alanine	DDAH2	NO
Bubalus Bubalis	MOL100006	Alanine	ADORA1	NO
Bubalus Bubalis	MOL100006	Alanine	SMPD4	NO
Bubalus Bubalis	MOL100006	Alanine	DGAT2	NO
Bubalus Bubalis	MOL100006	Alanine	SLC6A1	NO
Bubalus Bubalis	MOL100006	Alanine	ARPIN	NO
Bubalus Bubalis	MOL100006	Alanine	OSBPL8	NO
Bubalus Bubalis	MOL100006	Alanine	IVD	NO
Bubalus Bubalis	MOL100006	Alanine	GOT1L1	NO
Bubalus Bubalis	MOL100006	Alanine	PFAS	NO
Bubalus Bubalis	MOL100006	Alanine	SLC6A11	NO
Bubalus Bubalis	MOL100006	Alanine	STIM2	NO
Bubalus Bubalis	MOL100006	Alanine	CD101	NO
Bubalus Bubalis	MOL100006	Alanine	ACACA	NO
Bubalus Bubalis	MOL100006	Alanine	ADI1	NO
Bubalus Bubalis	MOL100006	Alanine	MTNR1B	NO
Bubalus Bubalis	MOL100006	Alanine	CCBL2	NO
Bubalus Bubalis	MOL100006	Alanine	CAT	YES
Bubalus Bubalis	MOL100006	Alanine	HMBS	NO
Bubalus Bubalis	MOL100006	Alanine	RNASE8	NO
Bubalus Bubalis	MOL100006	Alanine	APRT	YES
Bubalus Bubalis	MOL100006	Alanine	WNT7B	NO
Bubalus Bubalis	MOL100006	Alanine	CMPK2	NO
Bubalus Bubalis	MOL100006	Alanine	MOCS2	NO
Bubalus Bubalis	MOL100006	Alanine	MRI1	NO
Bubalus Bubalis	MOL100006	Alanine	PYCR1	NO
Bubalus Bubalis	MOL100006	Alanine	PKLR	NO
Bubalus Bubalis	MOL100006	Alanine	SLC11A1	NO
Bubalus Bubalis	MOL100006	Alanine	SLC3A1	NO
Bubalus Bubalis	MOL100006	Alanine	TNNT1	NO
Bubalus Bubalis	MOL100006	Alanine	HAAO	NO
Bubalus Bubalis	MOL100006	Alanine	ATP1A2	NO
Bubalus Bubalis	MOL100006	Alanine	CMPK1	NO
Bubalus Bubalis	MOL100006	Alanine	RYR3	NO
Bubalus Bubalis	MOL100006	Alanine	C2orf83	NO
Bubalus Bubalis	MOL100006	Alanine	SOD2	NO
Bubalus Bubalis	MOL100006	Alanine	LCN2	NO
Bubalus Bubalis	MOL100006	Alanine	ADSL	NO
Bubalus Bubalis	MOL100006	Alanine	PYCRL	NO
Bubalus Bubalis	MOL100006	Alanine	NAGS	NO
Bubalus Bubalis	MOL100006	Alanine	MYBPC3	NO
Bubalus Bubalis	MOL100006	Alanine	FAH	NO
Bubalus Bubalis	MOL100006	Alanine	ALDH4A1	NO
Bubalus Bubalis	MOL100006	Alanine	TNNI2	NO
Bubalus Bubalis	MOL100006	Alanine	MCEE	NO
Bubalus Bubalis	MOL100006	Alanine	NFS1	NO
Bubalus Bubalis	MOL100006	Alanine	AARS	NO
Bubalus Bubalis	MOL100006	Alanine	CBS	NO
Bubalus Bubalis	MOL100006	Alanine	SDS	NO

Bubalus Bubalis	MOL100006	Alanine	OAZ3	NO
Bubalus Bubalis	MOL100006	Alanine	PPAT	NO
Bubalus Bubalis	MOL100006	Alanine	ALAS1	NO
Bubalus Bubalis	MOL100006	Alanine	SLC25A15	NO
Bubalus Bubalis	MOL100006	Alanine	GLRA3	NO
Bubalus Bubalis	MOL100006	Alanine	GOT1	NO
Bubalus Bubalis	MOL100006	Alanine	GPR18	NO
Bubalus Bubalis	MOL100006	Alanine	LARS	NO
Bubalus Bubalis	MOL100006	Alanine	GRIN2C	NO
Bubalus Bubalis	MOL100006	Alanine	PIPOX	NO
Bubalus Bubalis	MOL100006	Alanine	VDAC1	YES
Bubalus Bubalis	MOL100006	Alanine	SLC32A1	NO
Bubalus Bubalis	MOL100006	Alanine	ASS1	YES
Bubalus Bubalis	MOL100006	Alanine	SLC7A4	NO
Bubalus Bubalis	MOL100006	Alanine	ARG2	NO
Bubalus Bubalis	MOL100006	Alanine	GLRA1	NO
Bubalus Bubalis	MOL100006	Alanine	PCK1	NO
Bubalus Bubalis	MOL100006	Alanine	GAMT	NO
Bubalus Bubalis	MOL100006	Alanine	ISCU	NO
Bubalus Bubalis	MOL100006	Alanine	BACE1	NO
Bubalus Bubalis	MOL100006	Alanine	SLC1A3	NO
Bubalus Bubalis	MOL100006	Alanine	CPS1	YES
Bubalus Bubalis	MOL100006	Alanine	PISD	NO
Bubalus Bubalis	MOL100006	Alanine	GCLC	NO
Bubalus Bubalis	MOL100006	Alanine	CARS2	NO
Bubalus Bubalis	MOL100006	Alanine	CACNA2D1	NO
Bubalus Bubalis	MOL100006	Alanine	MTRR	NO
Bubalus Bubalis	MOL100006	Alanine	PADI2	NO
Bubalus Bubalis	MOL100006	Alanine	PLG	YES
Bubalus Bubalis	MOL100006	Alanine	PRKCA	NO
Bubalus Bubalis	MOL100006	Alanine	ATP8A1	NO
Bubalus Bubalis	MOL100006	Alanine	PADI4	YES
Bubalus Bubalis	MOL100006	Alanine	NOS1	NO
Bubalus Bubalis	MOL100006	Alanine	GRIN1	NO
Bubalus Bubalis	MOL100006	Alanine	ACAT2	NO
Bubalus Bubalis	MOL100006	Alanine	TBPL1	NO
Bubalus Bubalis	MOL100006	Alanine	RARS	NO
Bubalus Bubalis	MOL100006	Alanine	PCMTD2	NO
Bubalus Bubalis	MOL100006	Alanine	ACACB	NO
Bubalus Bubalis	MOL100006	Alanine	TMLHE	NO
Bubalus Bubalis	MOL100006	Alanine	SLC7A9	NO
Bubalus Bubalis	MOL100006	Alanine	MOCOS	NO
Bubalus Bubalis	MOL100006	Alanine	PKD2	NO
Bubalus Bubalis	MOL100006	Alanine	ALDH9A1	NO
Bubalus Bubalis	MOL100006	Alanine	ARMT1	NO
Bubalus Bubalis	MOL100006	Alanine	NT5M	NO
Bubalus Bubalis	MOL100006	Alanine	BAK1	YES
Bubalus Bubalis	MOL100006	Alanine	TOMM40L	NO
Bubalus Bubalis	MOL100006	Alanine	ACAT1	NO
Bubalus Bubalis	MOL100006	Alanine	SLC36A2	NO
Bubalus Bubalis	MOL100006	Alanine	DAO	NO
Bubalus Bubalis	MOL100006	Alanine	ACADS	NO
Bubalus Bubalis	MOL100006	Alanine	TRPM1	NO
Bubalus Bubalis	MOL100006	Alanine	SLC28A3	NO

Bubalus Bubalis	MOL100006	Alanine	JUN	YES
Bubalus Bubalis	MOL100006	Alanine	AKR1B1	NO
Bubalus Bubalis	MOL100006	Alanine	HADHA	NO
Bubalus Bubalis	MOL100006	Alanine	NIT2	NO
Bubalus Bubalis	MOL100006	Alanine	ATIC	YES
Bubalus Bubalis	MOL100006	Alanine	HIF1A	NO
Bubalus Bubalis	MOL100006	Alanine	MTOR	YES
Bubalus Bubalis	MOL100006	Alanine	DHFR	NO
Bubalus Bubalis	MOL100006	Alanine	ATP13A2	NO
Bubalus Bubalis	MOL100006	Alanine	MYO5A	NO
Bubalus Bubalis	MOL100006	Alanine	EPRS	NO
Bubalus Bubalis	MOL100006	Alanine	RAB8B	NO
Bubalus Bubalis	MOL100006	Alanine	ACADM	NO
Bubalus Bubalis	MOL100006	Alanine	DPYSL2	NO
Bubalus Bubalis	MOL100006	Alanine	STAT5B	NO
Bubalus Bubalis	MOL100006	Alanine	ZP3	NO
Bubalus Bubalis	MOL100006	Alanine	GAD2	NO
Bubalus Bubalis	MOL100006	Alanine	KDM6B	NO
Bubalus Bubalis	MOL100006	Alanine	SLC36A3	NO
Bubalus Bubalis	MOL100006	Alanine	MOCS1	NO
Bubalus Bubalis	MOL100006	Alanine	GLS2	NO
Bubalus Bubalis	MOL100006	Alanine	SLC1A4	NO
Bubalus Bubalis	MOL100006	Alanine	AGXT	NO
Bubalus Bubalis	MOL100006	Alanine	SPTLC1	NO
Bubalus Bubalis	MOL100006	Alanine	SRR	NO
Bubalus Bubalis	MOL100006	Alanine	SHMT1	NO
Bubalus Bubalis	MOL100006	Alanine	ACY3	NO
Bubalus Bubalis	MOL100006	Alanine	BCAT1	NO
Bubalus Bubalis	MOL100006	Alanine	ADSSL1	NO
Bubalus Bubalis	MOL100006	Alanine	DARS2	NO
Bubalus Bubalis	MOL100006	Alanine	GNMT	NO
Bubalus Bubalis	MOL100006	Alanine	CTPS1	NO
Bubalus Bubalis	MOL100006	Alanine	ARG1	NO
Bubalus Bubalis	MOL100006	Alanine	OAT	NO
Bubalus Bubalis	MOL100006	Alanine	SLC25A2	NO
Bubalus Bubalis	MOL100006	Alanine	SLC6A9	NO
Bubalus Bubalis	MOL100006	Alanine	OAZ1	NO
Bubalus Bubalis	MOL100006	Alanine	ADSS	NO
Bubalus Bubalis	MOL100006	Alanine	CAD	NO
Bubalus Bubalis	MOL100006	Alanine	IARS	NO
Bubalus Bubalis	MOL100006	Alanine	LYZ	NO
Bubalus Bubalis	MOL100006	Alanine	CCBL1	NO
Bubalus Bubalis	MOL100006	Alanine	ASL	NO
Bubalus Bubalis	MOL100006	Alanine	SLC1A2	NO
Bubalus Bubalis	MOL100006	Alanine	ICMT	NO
Bubalus Bubalis	MOL100006	Alanine	TDO2	NO
Bubalus Bubalis	MOL100006	Alanine	LGSN	NO
Bubalus Bubalis	MOL100006	Alanine	FXN	NO
Bubalus Bubalis	MOL100006	Alanine	TARS	NO
Bubalus Bubalis	MOL100006	Alanine	CTH	NO
Bubalus Bubalis	MOL100006	Alanine	PLAT	YES
Bubalus Bubalis	MOL100006	Alanine	TH	NO
Bubalus Bubalis	MOL100006	Alanine	NOS2	YES
Bubalus Bubalis	MOL100006	Alanine	FARS2	NO

Bubalus Bubalis	MOL100006	Alanine	BHMT	NO
Bubalus Bubalis	MOL100006	Alanine	FARSB	NO
Bubalus Bubalis	MOL100006	Alanine	NOS3	YES
Bubalus Bubalis	MOL100006	Alanine	PADI6	NO
Bubalus Bubalis	MOL100006	Alanine	DGKD	NO
Bubalus Bubalis	MOL100006	Alanine	AIMP1	NO
Bubalus Bubalis	MOL100006	Alanine	APIP	NO
Bubalus Bubalis	MOL100006	Alanine	KLF9	NO
Bubalus Bubalis	MOL100006	Alanine	ACMSD	NO
Bubalus Bubalis	MOL100006	Alanine	MTAP	NO
Bubalus Bubalis	MOL100006	Alanine	GRM6	NO
Bubalus Bubalis	MOL100006	Alanine	STAT5A	NO
Bubalus Bubalis	MOL100006	Alanine	HTR2B	NO
Bubalus Bubalis	MOL100006	Alanine	SLC22A6	NO
Bubalus Bubalis	MOL100006	Alanine	AMDHD1	NO
Bubalus Bubalis	MOL100006	Alanine	KCNA1	NO
Bubalus Bubalis	MOL100006	Alanine	SLIT2	NO
Bubalus Bubalis	MOL100006	Alanine	SDSL	NO
Bubalus Bubalis	MOL100006	Alanine	HMGCL	NO
Bubalus Bubalis	MOL100006	Alanine	PQLC2	NO
Bubalus Bubalis	MOL100006	Alanine	PLCG2	NO
Bubalus Bubalis	MOL100006	Alanine	PHGDH	NO
Bubalus Bubalis	MOL100006	Alanine	TNNI3K	NO
Bubalus Bubalis	MOL100006	Alanine	SLC28A1	NO
Bubalus Bubalis	MOL100006	Alanine	PCMTD1	NO
Bubalus Bubalis	MOL100006	Alanine	DPYS	NO
Bubalus Bubalis	MOL100006	Alanine	AVP	NO
Bubalus Bubalis	MOL100006	Alanine	DDO	NO
Bubalus Bubalis	MOL100006	Alanine	DUT	NO
Bubalus Bubalis	MOL100006	Alanine	HCRT	NO
Bubalus Bubalis	MOL100006	Alanine	UROD	NO
Bubalus Bubalis	MOL100006	Alanine	NFKBIE	NO
Bubalus Bubalis	MOL100006	Alanine	AQP9	NO
Bubalus Bubalis	MOL100006	Alanine	SLC7A7	NO
Bubalus Bubalis	MOL100006	Alanine	HTT	NO
Bubalus Bubalis	MOL100006	Alanine	UMPS	NO
Bubalus Bubalis	MOL100006	Alanine	SLC1A6	NO
Bubalus Bubalis	MOL100006	Alanine	FH	NO
Bubalus Bubalis	MOL100006	Alanine	PYCR2	NO
Bubalus Bubalis	MOL100006	Alanine	AZIN1	NO
Bubalus Bubalis	MOL100006	Alanine	SLC6A12	NO
Bubalus Bubalis	MOL100006	Alanine	FAHD1	NO
Bubalus Bubalis	MOL100006	Alanine	AASS	NO
Bubalus Bubalis	MOL100006	Alanine	THNSL2	NO
Bubalus Bubalis	MOL100006	Alanine	GRM7	NO
Bubalus Bubalis	MOL100006	Alanine	PER2	NO
Bubalus Bubalis	MOL100007	4-Guanidino-1-Butanol	RDH11	NO
Bubalus Bubalis	MOL100007	4-Guanidino-1-Butanol	RDH5	NO
Bubalus Bubalis	MOL100007	4-Guanidino-1-Butanol	ALDH1A1	NO
Bubalus Bubalis	MOL100007	4-Guanidino-1-Butanol	ALDH1A2	NO
Bubalus Bubalis	MOL100007	4-Guanidino-1-Butanol	OPN4	NO
Bubalus Bubalis	MOL100007	4-Guanidino-1-Butanol	HSD17B7	NO
Bubalus Bubalis	MOL100007	4-Guanidino-1-Butanol	GATA3	YES
Bubalus Bubalis	MOL100007	4-Guanidino-1-Butanol	CRYM	NO

Bubalus Bubalis	MOL100007	4-Guanidino-1-Butanol	CYP1A1	NO
Bubalus Bubalis	MOL100007	4-Guanidino-1-Butanol	RBP3	NO
Bubalus Bubalis	MOL100007	4-Guanidino-1-Butanol	ALDH1A3	NO
Bubalus Bubalis	MOL100007	4-Guanidino-1-Butanol	RLBP1	NO
Bubalus Bubalis	MOL100007	4-Guanidino-1-Butanol	ALDH3A1	NO
Bubalus Bubalis	MOL100007	4-Guanidino-1-Butanol	RDH10	NO
Bubalus Bubalis	MOL100007	4-Guanidino-1-Butanol	AR	YES
Bubalus Bubalis	MOL100007	4-Guanidino-1-Butanol	ASCL1	NO
Bubalus Bubalis	MOL100007	4-Guanidino-1-Butanol	TSPO	NO
Bubalus Bubalis	MOL100007	4-Guanidino-1-Butanol	RETSAT	NO
Bubalus Bubalis	MOL100007	4-Guanidino-1-Butanol	RDH12	NO
Bubalus Bubalis	MOL100007	4-Guanidino-1-Butanol	RDH14	NO
Bubalus Bubalis	MOL100007	4-Guanidino-1-Butanol	RHO	NO
Bubalus Bubalis	MOL100007	4-Guanidino-1-Butanol	ALDH8A1	NO
Bubalus Bubalis	MOL100007	4-Guanidino-1-Butanol	THRA	NO
Bubalus Bubalis	MOL100007	4-Guanidino-1-Butanol	DLX5	NO
Bubalus Bubalis	MOL100007	4-Guanidino-1-Butanol	OPN5	NO
Bubalus Bubalis	MOL100007	4-Guanidino-1-Butanol	RDH13	NO
Bubalus Bubalis	MOL100007	4-Guanidino-1-Butanol	DHRS4	NO
Bubalus Bubalis	MOL100007	4-Guanidino-1-Butanol	RDH8	NO
Bubalus Bubalis	MOL100007	4-Guanidino-1-Butanol	RS1	NO
Bubalus Bubalis	MOL100007	4-Guanidino-1-Butanol	SHBG	NO
Bubalus Bubalis	MOL100007	4-Guanidino-1-Butanol	PAX2	NO
Bubalus Bubalis	MOL100007	4-Guanidino-1-Butanol	RET	NO
Bubalus Bubalis	MOL100007	4-Guanidino-1-Butanol	DHRS9	NO
Bubalus Bubalis	MOL100007	4-Guanidino-1-Butanol	DHRS3	NO
Bubalus Bubalis	MOL100007	4-Guanidino-1-Butanol	RBP1	NO
Bubalus Bubalis	MOL100007	4-Guanidino-1-Butanol	LRAT	NO
Bubalus Bubalis	MOL100007	4-Guanidino-1-Butanol	CTSH	NO
Bubalus Bubalis	MOL100007	4-Guanidino-1-Butanol	DHRS2	NO
Bubalus Bubalis	MOL100007	4-Guanidino-1-Butanol	CBR3	NO
Bubalus Bubalis	MOL100007	4-Guanidino-1-Butanol	THRB	NO
Bubalus Bubalis	MOL100007	4-Guanidino-1-Butanol	CBR1	NO
Bubalus Bubalis	MOL100008	Guanidine	GAMT	NO
Bubalus Bubalis	MOL100008	Guanidine	KCNA3	NO
Bubalus Bubalis	MOL100008	Guanidine	KCNA1	NO
Bubalus Bubalis	MOL100008	Guanidine	ADH1B	NO
Bubalus Bubalis	MOL100008	Guanidine	KCNA5	NO
Bubalus Bubalis	MOL100008	Guanidine	KCND3	NO
Bubalus Bubalis	MOL100008	Guanidine	KCNQ1	NO
Bubalus Bubalis	MOL100008	Guanidine	CYGB	NO
Bubalus Bubalis	MOL100008	Guanidine	IGF1	NO
Bubalus Bubalis	MOL100008	Guanidine	EDN1	YES
Bubalus Bubalis	MOL100008	Guanidine	CNTNAP4	NO
Bubalus Bubalis	MOL100008	Guanidine	GRIN2A	NO
Bubalus Bubalis	MOL100008	Guanidine	SPARC	NO
Bubalus Bubalis	MOL100008	Guanidine	CRLF1	NO
Bubalus Bubalis	MOL100008	Guanidine	ALDH1B1	NO
Bubalus Bubalis	MOL100008	Guanidine	DARS	NO
Bubalus Bubalis	MOL100008	Guanidine	MIP	NO
Bubalus Bubalis	MOL100008	Guanidine	STX1A	NO
Bubalus Bubalis	MOL100008	Guanidine	NRXN2	NO
Bubalus Bubalis	MOL100008	Guanidine	DAB2IP	NO
Bubalus Bubalis	MOL100008	Guanidine	DLG4	NO

Bubalus Bubalis	MOL100008	Guanidine	PRKAB1	YES
Bubalus Bubalis	MOL100008	Guanidine	KCNA2	NO
Bubalus Bubalis	MOL100008	Guanidine	KCNC2	NO
Bubalus Bubalis	MOL100008	Guanidine	KCND2	NO
Bubalus Bubalis	MOL100008	Guanidine	GUCY1B3	NO
Bubalus Bubalis	MOL100008	Guanidine	FBP1	NO
Bubalus Bubalis	MOL100008	Guanidine	NRXN3	NO
Bubalus Bubalis	MOL100008	Guanidine	SHANK3	NO
Bubalus Bubalis	MOL100008	Guanidine	OXT	NO
Bubalus Bubalis	MOL100008	Guanidine	RNASE4	NO
Bubalus Bubalis	MOL100008	Guanidine	SLC17A7	NO
Bubalus Bubalis	MOL100008	Guanidine	SORCS3	NO
Bubalus Bubalis	MOL100008	Guanidine	FADD	YES
Bubalus Bubalis	MOL100008	Guanidine	LEP	NO
Bubalus Bubalis	MOL100008	Guanidine	ANK3	NO
Bubalus Bubalis	MOL100008	Guanidine	RYR3	NO
Bubalus Bubalis	MOL100008	Guanidine	PAXBP1	NO
Bubalus Bubalis	MOL100008	Guanidine	ADORA1	NO
Bubalus Bubalis	MOL100008	Guanidine	CYP2E1	NO
Bubalus Bubalis	MOL100008	Guanidine	RNASE1	YES
Bubalus Bubalis	MOL100008	Guanidine	ADH1A	NO
Bubalus Bubalis	MOL100008	Guanidine	TPO	NO
Bubalus Bubalis	MOL100008	Guanidine	KCNC1	NO
Bubalus Bubalis	MOL100008	Guanidine	KCNA4	NO
Bubalus Bubalis	MOL100008	Guanidine	GATM	NO
Bubalus Bubalis	MOL100008	Guanidine	DGKI	NO
Bubalus Bubalis	MOL100008	Guanidine	LRRC4B	NO
Bubalus Bubalis	MOL100008	Guanidine	ACY3	NO
Bubalus Bubalis	MOL100008	Guanidine	MAGI2	NO
Bubalus Bubalis	MOL100008	Guanidine	RAB3A	NO
Bubalus Bubalis	MOL100008	Guanidine	CYP11A1	NO
Bubalus Bubalis	MOL100008	Guanidine	RNASE8	NO
Bubalus Bubalis	MOL100008	Guanidine	SNTG2	NO
Bubalus Bubalis	MOL100008	Guanidine	NLGN1	NO
Bubalus Bubalis	MOL100008	Guanidine	KCNE5	NO
Bubalus Bubalis	MOL100008	Guanidine	ZPR1	NO
Bubalus Bubalis	MOL100008	Guanidine	PAX7	NO
Bubalus Bubalis	MOL100008	Guanidine	ADH4	NO
Bubalus Bubalis	MOL100008	Guanidine	ACY1	NO
Bubalus Bubalis	MOL100008	Guanidine	ALDH2	NO
Bubalus Bubalis	MOL100008	Guanidine	KCNA10	NO
Bubalus Bubalis	MOL100008	Guanidine	CAT	YES
Bubalus Bubalis	MOL100008	Guanidine	ADH1C	NO
Bubalus Bubalis	MOL100008	Guanidine	KCNA7	NO
Bubalus Bubalis	MOL100008	Guanidine	KCNK4	NO
Bubalus Bubalis	MOL100008	Guanidine	ADH7	NO
Bubalus Bubalis	MOL100008	Guanidine	AQP8	NO
Bubalus Bubalis	MOL100008	Guanidine	ASPA	NO
Bubalus Bubalis	MOL100008	Guanidine	APOE	YES
Bubalus Bubalis	MOL100008	Guanidine	MC4R	NO
Bubalus Bubalis	MOL100008	Guanidine	SCN5A	NO
Bubalus Bubalis	MOL100008	Guanidine	KCNIP2	NO
Bubalus Bubalis	MOL100008	Guanidine	SCGB1A1	NO
Bubalus Bubalis	MOL100008	Guanidine	CACNA1D	NO

Bubalus Bubalis	MOL100008	Guanidine	PPARD	NO
Bubalus Bubalis	MOL100008	Guanidine	ALDH3B1	NO
Bubalus Bubalis	MOL100008	Guanidine	ALDH3B2	NO
Bubalus Bubalis	MOL100008	Guanidine	GJA5	NO
Bubalus Bubalis	MOL100008	Guanidine	KCND1	NO
Bubalus Bubalis	MOL100008	Guanidine	KCNC3	NO
Bubalus Bubalis	MOL100008	Guanidine	KCNB1	NO
Bubalus Bubalis	MOL100008	Guanidine	KCNB2	NO
Bubalus Bubalis	MOL100008	Guanidine	KCNA6	NO
Bubalus Bubalis	MOL100008	Guanidine	IYD	NO
Bubalus Bubalis	MOL100008	Guanidine	NFIB	NO
Bubalus Bubalis	MOL100008	Guanidine	ARX	NO
Bubalus Bubalis	MOL100008	Guanidine	NRXN1	NO
Bubalus Bubalis	MOL100008	Guanidine	SOX15	NO
Bubalus Bubalis	MOL100008	Guanidine	IL1B	YES
Bubalus Bubalis	MOL100008	Guanidine	SCN10A	NO
Bubalus Bubalis	MOL100008	Guanidine	GPX7	NO
Bubalus Bubalis	MOL100008	Guanidine	OXTR	NO
Bubalus Bubalis	MOL100008	Guanidine	RAPGEF2	NO
Bubalus Bubalis	MOL100008	Guanidine	NKX2-1	NO
Bubalus Bubalis	MOL100008	Guanidine	NPPA	NO
Bubalus Bubalis	MOL100008	Guanidine	RNASE2	NO
Bubalus Bubalis	MOL100008	Guanidine	FAS	NO
Bubalus Bubalis	MOL100009	Cholesterol	VDR	YES
Bubalus Bubalis	MOL100009	Cholesterol	PGR	NO
Bubalus Bubalis	MOL100009	Cholesterol	SNAI2	NO
Bubalus Bubalis	MOL100009	Cholesterol	FGF23	NO
Bubalus Bubalis	MOL100009	Cholesterol	BAX	NO
Bubalus Bubalis	MOL100009	Cholesterol	S100G	NO
Bubalus Bubalis	MOL100009	Cholesterol	NR1H4	NO
Bubalus Bubalis	MOL100009	Cholesterol	CYP27B1	NO
Bubalus Bubalis	MOL100009	Cholesterol	NFKB1	YES
Bubalus Bubalis	MOL100009	Cholesterol	MED1	NO
Bubalus Bubalis	MOL100009	Cholesterol	GFI1	NO
Bubalus Bubalis	MOL100009	Cholesterol	KL	NO
Bubalus Bubalis	MOL100009	Cholesterol	CYP27A1	NO
Bubalus Bubalis	MOL100009	Cholesterol	TRIM24	NO
Bubalus Bubalis	MOL100009	Cholesterol	GC	YES
Bubalus Bubalis	MOL100009	Cholesterol	AKR1C3	NO
Bubalus Bubalis	MOL100009	Cholesterol	SNAI1	NO
Bubalus Bubalis	MOL100009	Cholesterol	LANCL2	NO
Bubalus Bubalis	MOL100009	Cholesterol	CYP2R1	NO
Bubalus Bubalis	MOL100009	Cholesterol	KANK2	NO
Bubalus Bubalis	MOL100009	Cholesterol	SNW1	NO
Bubalus Bubalis	MOL100009	Cholesterol	CYP24A1	NO
Bubalus Bubalis	MOL100009	Cholesterol	CYP3A4	YES
Bubalus Bubalis	MOL100009	Cholesterol	WNT4	NO
Bubalus Bubalis	MOL100009	Cholesterol	PML	YES
Bubalus Bubalis	MOL100009	Cholesterol	IRX5	NO
Bubalus Bubalis	MOL100009	Cholesterol	ESR1	YES
Bubalus Bubalis	MOL100009	Cholesterol	GPBAR1	NO
Bubalus Bubalis	MOL100009	Cholesterol	CALB1	NO
Bubalus Bubalis	MOL100009	Cholesterol	TCF3	NO
Bubalus Bubalis	MOL100009	Cholesterol	B4GALT1	NO

Bubalus Bubalis	MOL100009	Cholesterol	RXRA	NO

Therapeutic Target Proteins
