# Supplementary material for: Real-World Study on Chai-Shi-Jie-Du Granules for the Treatment of Dengue Fever and the Possible Mechanisms Based on Network Pharmacology
Source: Evid Based Complement Alternat Med. 2023 Aug 30;2023:9942842. doi: 10.1155/2023/9942842 (PMC10482559; doi:10.1155/2023/9942842)
Supplement: Supplementary Materials — Table S1: duration of symptoms and signs between the CSJD and control groups. Table S2: drug-active ingredient-target database. Table S3: the GO enrichment results for the 108 cotargets. Table S4: the KEGG enrichment results for the 108 cotargets. Figure S1: the PPI network of 108 potential therapeutic target proteins. Figure S2: the “drug-active compound-therapeutic target protein” network of the effect of CSJD on dengue fever. Red represents the most important potential therapeutic targets, and orange represents the less important potential therapeutic targets; other potential therapeutic targets are colored yellow. Green represents the active compound. Purple represents the constituent drugs of CSJD. The edges represent the relationship between constituent drugs, active compounds, and potential therapeutic target proteins. [file 9942842.f1.zip › Table S3. The GO enrichment results for the 108 cotargets.pdf]

ONTOLOGY	ID	Description
BP	GO:0032496	response to lipopolysaccharide
BP	GO:0002237	response to molecule of bacterial origin
BP	GO:0006979	response to oxidative stress
BP	GO:0009314	response to radiation
BP	GO:0071216	cellular response to biotic stimulus
BP	GO:0000302	response to reactive oxygen species
BP	GO:0062197	cellular response to chemical stress
BP	GO:0031667	response to nutrient levels
BP	GO:0010038	response to metal ion
BP	GO:0071222	cellular response to lipopolysaccharide
BP	GO:2001233	regulation of apoptotic signaling pathway
BP	GO:0034599	cellular response to oxidative stress
BP	GO:0071219	cellular response to molecule of bacterial origin
BP	GO:0071214	cellular response to abiotic stimulus
BP	GO:0104004	cellular response to environmental stimulus
BP	GO:0046209	nitric oxide metabolic process
BP	GO:2001057	reactive nitrogen species metabolic process
BP	GO:0009411	response to UV
BP	GO:0034612	response to tumor necrosis factor
BP	GO:0042060	wound healing
BP	GO:0007568	aging
BP	GO:0048732	gland development
BP	GO:0006809	nitric oxide biosynthetic process
BP	GO:2001236	regulation of extrinsic apoptotic signaling pathway
BP	GO:0009410	response to xenobiotic stimulus
BP	GO:0097191	extrinsic apoptotic signaling pathway
BP	GO:2001234	negative regulation of apoptotic signaling pathway
BP	GO:0045429	positive regulation of nitric oxide biosynthetic process
BP	GO:1904407	positive regulation of nitric oxide metabolic process
BP	GO:1901653	cellular response to peptide
BP	GO:0071496	cellular response to external stimulus
BP	GO:2001237	negative regulation of extrinsic apoptotic signaling pathway
BP	GO:0009612	response to mechanical stimulus
BP	GO:0045428	regulation of nitric oxide biosynthetic process
BP	GO:0080164	regulation of nitric oxide metabolic process
BP	GO:0071356	cellular response to tumor necrosis factor
BP	GO:0001666	response to hypoxia
BP	GO:0046686	response to cadmium ion
BP	GO:0070997	neuron death
BP	GO:0072593	reactive oxygen species metabolic process
BP	GO:0009416	response to light stimulus
BP	GO:0036293	response to decreased oxygen levels
BP	GO:0043434	response to peptide hormone
BP	GO:0033002	muscle cell proliferation
BP	GO:0071276	cellular response to cadmium ion
BP	GO:0062012	regulation of small molecule metabolic process
BP	GO:1903037	regulation of leukocyte cell-cell adhesion
BP	GO:0034614	cellular response to reactive oxygen species
BP	GO:0070482	response to oxygen levels
BP	GO:0022407	regulation of cell-cell adhesion
BP	GO:0048661	positive regulation of smooth muscle cell proliferation
BP	GO:1901214	regulation of neuron death
BP	GO:0007159	leukocyte cell-cell adhesion

BP	GO:0019221	cytokine-mediated signaling pathway
BP	GO:0032103	positive regulation of response to external stimulus
BP	GO:0007584	response to nutrient
BP	GO:0071375	cellular response to peptide hormone stimulus
BP	GO:0050727	regulation of inflammatory response
BP	GO:0048660	regulation of smooth muscle cell proliferation
BP	GO:0010212	response to ionizing radiation
BP	GO:0044706	multi-multicellular organism process
BP	GO:0048659	smooth muscle cell proliferation
BP	GO:0071241	cellular response to inorganic substance
BP	GO:1901654	response to ketone
BP	GO:0001819	positive regulation of cytokine production
BP	GO:0071248	cellular response to metal ion
BP	GO:1903039	positive regulation of leukocyte cell-cell adhesion
BP	GO:0061041	regulation of wound healing
BP	GO:1903034	regulation of response to wounding
BP	GO:0050878	regulation of body fluid levels
BP	GO:0030099	myeloid cell differentiation
BP	GO:0042110	T cell activation
BP	GO:0048545	response to steroid hormone
BP	GO:0032355	response to estradiol
BP	GO:0002526	acute inflammatory response
BP	GO:0030193	regulation of blood coagulation
BP	GO:0007596	blood coagulation
BP	GO:0009636	response to toxic substance
BP	GO:1900046	regulation of hemostasis
BP	GO:0007599	hemostasis
BP	GO:0050817	coagulation
BP	GO:0071478	cellular response to radiation
BP	GO:1904019	epithelial cell apoptotic process
BP	GO:0050818	regulation of coagulation
BP	GO:1904035	regulation of epithelial cell apoptotic process
BP	GO:0050900	leukocyte migration
BP	GO:2000377	regulation of reactive oxygen species metabolic process
BP	GO:0007565	female pregnancy
BP	GO:1903131	mononuclear cell differentiation
BP	GO:0031331	positive regulation of cellular catabolic process
BP	GO:0022409	positive regulation of cell-cell adhesion
BP	GO:0045785	positive regulation of cell adhesion
BP	GO:0050673	epithelial cell proliferation
BP	GO:0097193	intrinsic apoptotic signaling pathway
BP	GO:0051090	regulation of DNA-binding transcription factor activity
BP	GO:0010332	response to gamma radiation
BP	GO:0070663	regulation of leukocyte proliferation
BP	GO:0018209	peptidyl-serine modification
BP	GO:0002685	regulation of leukocyte migration
BP	GO:0071260	cellular response to mechanical stimulus
BP	GO:0032768	regulation of monooxygenase activity
BP	GO:0051091	positive regulation of DNA-binding transcription factor acti
BP	GO:0032757	positive regulation of interleukin-8 production
BP	GO:0042542	response to hydrogen peroxide
BP	GO:0043200	response to amino acid
BP	GO:0018105	peptidyl-serine phosphorylation
BP	GO:0070661	leukocyte proliferation

BP	GO:0038034	signal transduction in absence of ligand
BP	GO:0097192	extrinsic apoptotic signaling pathway in absence of ligand
BP	GO:0043410	positive regulation of MAPK cascade
BP	GO:0048608	reproductive structure development
BP	GO:0061458	reproductive system development
BP	GO:0030098	lymphocyte differentiation
BP	GO:0035094	response to nicotine
BP	GO:0051235	maintenance of location
BP	GO:0009896	positive regulation of catabolic process
BP	GO:0050863	regulation of T cell activation
BP	GO:1903035	negative regulation of response to wounding
BP	GO:0045765	regulation of angiogenesis
BP	GO:0051402	neuron apoptotic process
BP	GO:0046683	response to organophosphorus
BP	GO:0031960	response to corticosteroid
BP	GO:1901342	regulation of vasculature development
BP	GO:0042063	gliogenesis
BP	GO:0001101	response to acid chemical
BP	GO:0061045	negative regulation of wound healing
BP	GO:0090559	regulation of membrane permeability
BP	GO:0008637	apoptotic mitochondrial changes
BP	GO:0051341	regulation of oxidoreductase activity
BP	GO:0016241	regulation of macroautophagy
BP	GO:0035296	regulation of tube diameter
BP	GO:0097746	blood vessel diameter maintenance
BP	GO:0035150	regulation of tube size
BP	GO:0009615	response to virus
BP	GO:0062013	positive regulation of small molecule metabolic process
BP	GO:0009408	response to heat
BP	GO:0010506	regulation of autophagy
BP	GO:0008625	extrinsic apoptotic signaling pathway via death domain recep
BP	GO:0008202	steroid metabolic process
BP	GO:0010001	glial cell differentiation
BP	GO:0050670	regulation of lymphocyte proliferation
BP	GO:0018108	peptidyl-tyrosine phosphorylation
BP	GO:2000351	regulation of endothelial cell apoptotic process
BP	GO:0032944	regulation of mononuclear cell proliferation
BP	GO:0014074	response to purine-containing compound
BP	GO:0051384	response to glucocorticoid
BP	GO:0018212	peptidyl-tyrosine modification
BP	GO:0031663	lipopolysaccharide-mediated signaling pathway
BP	GO:0050678	regulation of epithelial cell proliferation
BP	GO:0070665	positive regulation of leukocyte proliferation
BP	GO:0060135	maternal process involved in female pregnancy
BP	GO:0046902	regulation of mitochondrial membrane permeability
BP	GO:0070141	response to UV-A
BP	GO:0034644	cellular response to UV
BP	GO:0071674	mononuclear cell migration
BP	GO:0046651	lymphocyte proliferation
BP	GO:0072577	endothelial cell apoptotic process
BP	GO:0032943	mononuclear cell proliferation
BP	GO:0150076	neuroinflammatory response
BP	GO:0048538	thymus development
BP	GO:0048511	rhythmic process

BP	GO:0002573	myeloid leukocyte differentiation
BP	GO:1901216	positive regulation of neuron death
BP	GO:0030217	T cell differentiation
BP	GO:0043491	protein kinase B signaling
BP	GO:0043523	regulation of neuron apoptotic process
BP	GO:0032102	negative regulation of response to external stimulus
BP	GO:1903706	regulation of hemopoiesis
BP	GO:0002687	positive regulation of leukocyte migration
BP	GO:0006953	acute-phase response
BP	GO:0030195	negative regulation of blood coagulation
BP	GO:0050870	positive regulation of T cell activation
BP	GO:0003018	vascular process in circulatory system
BP	GO:0032677	regulation of interleukin-8 production
BP	GO:0032868	response to insulin
BP	GO:0006091	generation of precursor metabolites and energy
BP	GO:0032637	interleukin-8 production
BP	GO:0030879	mammary gland development
BP	GO:0010822	positive regulation of mitochondrion organization
BP	GO:1902042	negative regulation of extrinsic apoptotic signaling pathway
BP	GO:1900047	negative regulation of hemostasis
BP	GO:1904036	negative regulation of epithelial cell apoptotic process
BP	GO:0009266	response to temperature stimulus
BP	GO:0001936	regulation of endothelial cell proliferation
BP	GO:0048872	homeostasis of number of cells
BP	GO:1905952	regulation of lipid localization
BP	GO:0033138	positive regulation of peptidyl-serine phosphorylation
BP	GO:0050819	negative regulation of coagulation
BP	GO:0010821	regulation of mitochondrion organization
BP	GO:0014065	phosphatidylinositol 3-kinase signaling
BP	GO:0033135	regulation of peptidyl-serine phosphorylation
BP	GO:0048638	regulation of developmental growth
BP	GO:0048708	astrocyte differentiation
BP	GO:1904645	response to amyloid-beta
BP	GO:0001935	endothelial cell proliferation
BP	GO:0007006	mitochondrial membrane organization
BP	GO:0010632	regulation of epithelial cell migration
BP	GO:0033674	positive regulation of kinase activity
BP	GO:0071346	cellular response to interferon-gamma
BP	GO:0051767	nitric-oxide synthase biosynthetic process
BP	GO:0051769	regulation of nitric-oxide synthase biosynthetic process
BP	GO:0042098	T cell proliferation
BP	GO:0031668	cellular response to extracellular stimulus
BP	GO:0019915	lipid storage
BP	GO:0071900	regulation of protein serine/threonine kinase activity
BP	GO:0071548	response to dexamethasone
BP	GO:0071482	cellular response to light stimulus
BP	GO:0051251	positive regulation of lymphocyte activation
BP	GO:0051924	regulation of calcium ion transport
BP	GO:2001242	regulation of intrinsic apoptotic signaling pathway
BP	GO:0150077	regulation of neuroinflammatory response
BP	GO:0030194	positive regulation of blood coagulation
BP	GO:0035994	response to muscle stretch
BP	GO:1900048	positive regulation of hemostasis
BP	GO:0043254	regulation of protein-containing complex assembly

BP	GO:0045637	regulation of myeloid cell differentiation
BP	GO:0051591	response to cAMP
BP	GO:0045862	positive regulation of proteolysis
BP	GO:0002532	production of molecular mediator involved in inflammatory re
BP	GO:0050820	positive regulation of coagulation
BP	GO:0043467	regulation of generation of precursor metabolites and energy
BP	GO:0050730	regulation of peptidyl-tyrosine phosphorylation
BP	GO:0031669	cellular response to nutrient levels
BP	GO:0042129	regulation of T cell proliferation
BP	GO:0009895	negative regulation of catabolic process
BP	GO:1903798	regulation of production of miRNAs involved in gene silencin
BP	GO:0048771	tissue remodeling
BP	GO:0050999	regulation of nitric-oxide synthase activity
BP	GO:0010634	positive regulation of epithelial cell migration
BP	GO:0034605	cellular response to heat
BP	GO:0045860	positive regulation of protein kinase activity
BP	GO:0043405	regulation of MAP kinase activity
BP	GO:0035265	organ growth
BP	GO:0048015	phosphatidylinositol-mediated signaling
BP	GO:0050671	positive regulation of lymphocyte proliferation
BP	GO:0070920	regulation of production of small RNA involved in gene silen
BP	GO:0032946	positive regulation of mononuclear cell proliferation
BP	GO:0045766	positive regulation of angiogenesis
BP	GO:1904018	positive regulation of vasculature development
BP	GO:0048017	inositol lipid-mediated signaling
BP	GO:0034341	response to interferon-gamma
BP	GO:0050921	positive regulation of chemotaxis
BP	GO:0002697	regulation of immune effector process
BP	GO:0070555	response to interleukin-1
BP	GO:0006801	superoxide metabolic process
BP	GO:1990776	response to angiotensin
BP	GO:0033077	T cell differentiation in thymus
BP	GO:1902041	regulation of extrinsic apoptotic signaling pathway via deat
BP	GO:0001503	ossification
BP	GO:0002696	positive regulation of leukocyte activation
BP	GO:0051403	stress-activated MAPK cascade
BP	GO:0010827	regulation of glucose transmembrane transport
BP	GO:1905954	positive regulation of lipid localization
BP	GO:0050731	positive regulation of peptidyl-tyrosine phosphorylation
BP	GO:0016032	viral process
BP	GO:1903799	negative regulation of production of miRNAs involved in gene
BP	GO:0010631	epithelial cell migration
BP	GO:0051092	positive regulation of NF-kappaB transcription factor activi
BP	GO:0071347	cellular response to interleukin-1
BP	GO:0050867	positive regulation of cell activation
BP	GO:0042594	response to starvation
BP	GO:2000378	negative regulation of reactive oxygen species metabolic pro
BP	GO:0051052	regulation of DNA metabolic process
BP	GO:0031098	stress-activated protein kinase signaling cascade
BP	GO:0046660	female sex differentiation
BP	GO:0090132	epithelium migration
BP	GO:0071675	regulation of mononuclear cell migration
BP	GO:0043620	regulation of DNA-templated transcription in response to str
BP	GO:0071902	positive regulation of protein serine/threonine kinase activ

BP	GO:0051098	regulation of binding
BP	GO:0051054	positive regulation of DNA metabolic process
BP	GO:0090130	tissue migration
BP	GO:0051770	positive regulation of nitric-oxide synthase biosynthetic pro
BP	GO:0002262	myeloid cell homeostasis
BP	GO:0097305	response to alcohol
BP	GO:0032869	cellular response to insulin stimulus
BP	GO:0006839	mitochondrial transport
BP	GO:0042310	vasoconstriction
BP	GO:0034976	response to endoplasmic reticulum stress
BP	GO:0019932	second-messenger-mediated signaling
BP	GO:0017038	protein import
BP	GO:0051897	positive regulation of protein kinase B signaling
BP	GO:1901099	negative regulation of signal transduction in absence of liga
BP	GO:2001240	negative regulation of extrinsic apoptotic signaling pathway
BP	GO:0034250	positive regulation of cellular amide metabolic process
BP	GO:0045936	negative regulation of phosphate metabolic process
BP	GO:0002688	regulation of leukocyte chemotaxis
BP	GO:0007623	circadian rhythm
BP	GO:0010563	negative regulation of phosphorus metabolic process
BP	GO:0032770	positive regulation of monooxygenase activity
BP	GO:2000352	negative regulation of endothelial cell apoptotic process
BP	GO:0010508	positive regulation of autophagy
BP	GO:0097237	cellular response to toxic substance
BP	GO:0051607	defense response to virus
BP	GO:0140546	defense response to symbiont
BP	GO:0001893	maternal placenta development
BP	GO:0045907	positive regulation of vasoconstriction
BP	GO:0001836	release of cytochrome c from mitochondria
BP	GO:0051353	positive regulation of oxidoreductase activity
BP	GO:0090303	positive regulation of wound healing
BP	GO:0007259	receptor signaling pathway via JAK-STAT
BP	GO:0046890	regulation of lipid biosynthetic process
BP	GO:0042176	regulation of protein catabolic process
BP	GO:0070371	ERK1 and ERK2 cascade
BP	GO:0006694	steroid biosynthetic process
BP	GO:0043270	positive regulation of ion transport
BP	GO:1900221	regulation of amyloid-beta clearance
BP	GO:0032755	positive regulation of interleukin-6 production
BP	GO:0033273	response to vitamin
BP	GO:0032885	regulation of polysaccharide biosynthetic process
BP	GO:0001659	temperature homeostasis
BP	GO:1903829	positive regulation of cellular protein localization
BP	GO:0032615	interleukin-12 production
BP	GO:0032655	regulation of interleukin-12 production
BP	GO:0050920	regulation of chemotaxis
BP	GO:0002690	positive regulation of leukocyte chemotaxis
BP	GO:0007569	cell aging
BP	GO:0031349	positive regulation of defense response
BP	GO:0016239	positive regulation of macroautophagy
BP	GO:1902105	regulation of leukocyte differentiation
BP	GO:0008585	female gonad development
BP	GO:0036473	cell death in response to oxidative stress
BP	GO:0097242	amyloid-beta clearance

BP	GO:0002703	regulation of leukocyte mediated immunity
BP	GO:0071453	cellular response to oxygen levels
BP	GO:0071466	cellular response to xenobiotic stimulus
BP	GO:0006109	regulation of carbohydrate metabolic process
BP	GO:0051051	negative regulation of transport
BP	GO:1901655	cellular response to ketone
BP	GO:0042359	vitamin D metabolic process
BP	GO:0060252	positive regulation of glial cell proliferation
BP	GO:0010959	regulation of metal ion transport
BP	GO:0001667	ameboidal-type cell migration
BP	GO:0097696	receptor signaling pathway via STAT
BP	GO:0060401	cytosolic calcium ion transport
BP	GO:0019229	regulation of vasoconstriction
BP	GO:0008630	intrinsic apoptotic signaling pathway in response to DNA dama
BP	GO:0010543	regulation of platelet activation
BP	GO:0034504	protein localization to nucleus
BP	GO:0046545	development of primary female sexual characteristics
BP	GO:2000116	regulation of cysteine-type endopeptidase activity
BP	GO:0061180	mammary gland epithelium development
BP	GO:0016236	macroautophagy
BP	GO:0032735	positive regulation of interleukin-12 production
BP	GO:0042102	positive regulation of T cell proliferation
BP	GO:0031334	positive regulation of protein-containing complex assembly
BP	GO:1901617	organic hydroxy compound biosynthetic process
BP	GO:0010522	regulation of calcium ion transport into cytosol
BP	GO:0050729	positive regulation of inflammatory response
BP	GO:0046879	hormone secretion
BP	GO:0050890	cognition
BP	GO:0031281	positive regulation of cyclase activity
BP	GO:0060965	negative regulation of gene silencing by miRNA
BP	GO:1902895	positive regulation of pri-miRNA transcription by RNA polyme
BP	GO:0045639	positive regulation of myeloid cell differentiation
BP	GO:0006816	calcium ion transport
BP	GO:0001890	placenta development
BP	GO:0045981	positive regulation of nucleotide metabolic process
BP	GO:1900544	positive regulation of purine nucleotide metabolic process
BP	GO:2001239	regulation of extrinsic apoptotic signaling pathway in absen
BP	GO:0046620	regulation of organ growth
BP	GO:0051222	positive regulation of protein transport
BP	GO:0002285	lymphocyte activation involved in immune response
BP	GO:0002534	cytokine production involved in inflammatory response
BP	GO:1900015	regulation of cytokine production involved in inflammatory r
BP	GO:1903036	positive regulation of response to wounding
BP	GO:0032881	regulation of polysaccharide metabolic process
BP	GO:0006006	glucose metabolic process
BP	GO:0032368	regulation of lipid transport
BP	GO:0009914	hormone transport
BP	GO:0042368	vitamin D biosynthetic process
BP	GO:0051918	negative regulation of fibrinolysis
BP	GO:1901524	regulation of mitophagy
BP	GO:0008631	intrinsic apoptotic signaling pathway in response to oxidati
BP	GO:0070372	regulation of ERK1 and ERK2 cascade
BP	GO:0071456	cellular response to hypoxia
BP	GO:0060149	negative regulation of posttranscriptional gene silencing

BP	GO:0060967	negative regulation of gene silencing by RNA
BP	GO:0051881	regulation of mitochondrial membrane potential
BP	GO:0060326	cell chemotaxis
BP	GO:1904659	glucose transmembrane transport
BP	GO:0016125	sterol metabolic process
BP	GO:0022411	cellular component disassembly
BP	GO:0007611	learning or memory
BP	GO:0030856	regulation of epithelial cell differentiation
BP	GO:0043406	positive regulation of MAP kinase activity
BP	GO:0046632	alpha-beta T cell differentiation
BP	GO:2000379	positive regulation of reactive oxygen species metabolic pro[illegible]
BP	GO:0006606	protein import into nucleus
BP	GO:0042730	fibrinolysis
BP	GO:1904385	cellular response to angiotensin
BP	GO:1904996	positive regulation of leukocyte adhesion to vascular endoth[illegible]
BP	GO:0043618	regulation of transcription from RNA polymerase II promoter [illegible]
BP	GO:0046677	response to antibiotic
BP	GO:0010876	lipid localization
BP	GO:0009267	cellular response to starvation
BP	GO:0045927	positive regulation of growth
BP	GO:0015980	energy derivation by oxidation of organic compounds
BP	GO:0002286	T cell activation involved in immune response
BP	GO:0008645	hexose transmembrane transport
BP	GO:1904951	positive regulation of establishment of protein localization
BP	GO:1902003	regulation of amyloid-beta formation
BP	GO:0050679	positive regulation of epithelial cell proliferation
BP	GO:0001660	fever generation
BP	GO:0071492	cellular response to UV-A
BP	GO:1901215	negative regulation of neuron death
BP	GO:0006706	steroid catabolic process
BP	GO:0046697	decidualization
BP	GO:0051170	import into nucleus
BP	GO:0015749	monosaccharide transmembrane transport
BP	GO:1990748	cellular detoxification
BP	GO:0043281	regulation of cysteine-type endopeptidase activity involved [illegible]
BP	GO:0060964	regulation of gene silencing by miRNA
BP	GO:0030522	intracellular receptor signaling pathway
BP	GO:0036294	cellular response to decreased oxygen levels
BP	GO:0022612	gland morphogenesis
BP	GO:0034219	carbohydrate transmembrane transport
BP	GO:0051101	regulation of DNA binding
BP	GO:0052547	regulation of peptidase activity
BP	GO:0051348	negative regulation of transferase activity
BP	GO:0023061	signal release
BP	GO:0051651	maintenance of location in cell
BP	GO:0002761	regulation of myeloid leukocyte differentiation
BP	GO:0046889	positive regulation of lipid biosynthetic process
BP	GO:0019216	regulation of lipid metabolic process
BP	GO:0035196	production of miRNAs involved in gene silencing by miRNA
BP	GO:0060147	regulation of posttranscriptional gene silencing
BP	GO:0010660	regulation of muscle cell apoptotic process
BP	GO:0032370	positive regulation of lipid transport
BP	GO:1900542	regulation of purine nucleotide metabolic process
BP	GO:0002366	leukocyte activation involved in immune response

BP	GO:0034764	positive regulation of transmembrane transport
BP	GO:0030168	platelet activation
BP	GO:0007548	sex differentiation
BP	GO:0097529	myeloid leukocyte migration
BP	GO:0007566	embryo implantation
BP	GO:0031279	regulation of cyclase activity
BP	GO:0060966	regulation of gene silencing by RNA
BP	GO:0097194	execution phase of apoptosis
BP	GO:0008406	gonad development
BP	GO:0002360	T cell lineage commitment
BP	GO:0005979	regulation of glycogen biosynthetic process
BP	GO:0010962	regulation of glucan biosynthetic process
BP	GO:0060969	negative regulation of gene silencing
BP	GO:0002263	cell activation involved in immune response
BP	GO:0071621	granulocyte chemotaxis
BP	GO:0006140	regulation of nucleotide metabolic process
BP	GO:0010524	positive regulation of calcium ion transport into cytosol
BP	GO:0031050	dsRNA processing
BP	GO:0070918	production of small RNA involved in gene silencing by RNA
BP	GO:1902893	regulation of pri-miRNA transcription by RNA polymerase II
BP	GO:1900182	positive regulation of protein localization to nucleus
BP	GO:0016049	cell growth
BP	GO:0035270	endocrine system development
BP	GO:0042362	fat-soluble vitamin biosynthetic process
BP	GO:0061614	pri-miRNA transcription by RNA polymerase II
BP	GO:1902991	regulation of amyloid precursor protein catabolic process
BP	GO:0008209	androgen metabolic process
BP	GO:0001558	regulation of cell growth
BP	GO:0045137	development of primary sexual characteristics
BP	GO:0010657	muscle cell apoptotic process
BP	GO:0046622	positive regulation of organ growth
BP	GO:0030595	leukocyte chemotaxis
BP	GO:0034205	amyloid-beta formation
BP	GO:0061756	leukocyte adhesion to vascular endothelial cell
BP	GO:0044403	biological process involved in symbiotic interaction
BP	GO:0010594	regulation of endothelial cell migration
BP	GO:0072594	establishment of protein localization to organelle
BP	GO:0060485	mesenchyme development
BP	GO:0051917	regulation of fibrinolysis
BP	GO:0002460	adaptive immune response based on somatic recombination of i[illegible]
BP	GO:0002931	response to ischemia
BP	GO:0010595	positive regulation of endothelial cell migration
BP	GO:0002699	positive regulation of immune effector process
BP	GO:0048762	mesenchymal cell differentiation
BP	GO:0046324	regulation of glucose import
BP	GO:0019318	hexose metabolic process
BP	GO:1900180	regulation of protein localization to nucleus
BP	GO:0052548	regulation of endopeptidase activity
BP	GO:0051896	regulation of protein kinase B signaling
BP	GO:0042982	amyloid precursor protein metabolic process
BP	GO:0008203	cholesterol metabolic process
BP	GO:0045471	response to ethanol
BP	GO:0006913	nucleocytoplasmic transport
BP	GO:0051169	nuclear transport

BP	GO:0002718	regulation of cytokine production involved in immune respons[illegible]
BP	GO:0043255	regulation of carbohydrate biosynthetic process
BP	GO:0050804	modulation of chemical synaptic transmission
BP	GO:0046824	positive regulation of nucleocytoplasmic transport
BP	GO:0002443	leukocyte mediated immunity
BP	GO:0099177	regulation of trans-synaptic signaling
BP	GO:0002367	cytokine production involved in immune response
BP	GO:2001243	negative regulation of intrinsic apoptotic signaling pathway
BP	GO:0030183	B cell differentiation
BP	GO:0033280	response to vitamin D
BP	GO:0034405	response to fluid shear stress
BP	GO:0070059	intrinsic apoptotic signaling pathway in response to endopla[illegible]
BP	GO:0031341	regulation of cell killing
BP	GO:0032872	regulation of stress-activated MAPK cascade
BP	GO:0038061	NIK/NF-kappaB signaling
BP	GO:0044282	small molecule catabolic process
BP	GO:0046883	regulation of hormone secretion
BP	GO:0051044	positive regulation of membrane protein ectodomain proteolys[illegible]
BP	GO:0060251	regulation of glial cell proliferation
BP	GO:0070873	regulation of glycogen metabolic process
BP	GO:0098869	cellular oxidant detoxification
BP	GO:0070302	regulation of stress-activated protein kinase signaling casc[illegible]
BP	GO:0062014	negative regulation of small molecule metabolic process
BP	GO:0010675	regulation of cellular carbohydrate metabolic process
BP	GO:0010952	positive regulation of peptidase activity
BP	GO:1902652	secondary alcohol metabolic process
BP	GO:0060416	response to growth hormone
BP	GO:0014013	regulation of gliogenesis
BP	GO:0014015	positive regulation of gliogenesis
BP	GO:0050766	positive regulation of phagocytosis
BP	GO:0006959	humoral immune response
BP	GO:0097530	granulocyte migration
BP	GO:0007346	regulation of mitotic cell cycle
BP	GO:0034349	glial cell apoptotic process
BP	GO:0005996	monosaccharide metabolic process
BP	GO:0045834	positive regulation of lipid metabolic process
BP	GO:0050435	amyloid-beta metabolic process
BP	GO:0016042	lipid catabolic process
BP	GO:0010742	macrophage derived foam cell differentiation
BP	GO:0090077	foam cell differentiation
BP	GO:0019722	calcium-mediated signaling
BP	GO:0002294	CD4-positive, alpha-beta T cell differentiation involved in [illegible]
BP	GO:0042987	amyloid precursor protein catabolic process
BP	GO:0043535	regulation of blood vessel endothelial cell migration
BP	GO:0048754	branching morphogenesis of an epithelial tube
BP	GO:0071887	leukocyte apoptotic process
BP	GO:0008643	carbohydrate transport
BP	GO:0098754	detoxification
BP	GO:0071383	cellular response to steroid hormone stimulus
BP	GO:0060562	epithelial tube morphogenesis
BP	GO:0002287	alpha-beta T cell activation involved in immune response
BP	GO:0002293	alpha-beta T cell differentiation involved in immune respons[illegible]
BP	GO:0050810	regulation of steroid biosynthetic process
BP	GO:1901224	positive regulation of NIK/NF-kappaB signaling

BP	GO:0002831	regulation of response to biotic stimulus
BP	GO:0031649	heat generation
BP	GO:0035743	CD4-positive, alpha-beta T cell cytokine production
BP	GO:0006163	purine nucleotide metabolic process
BP	GO:0045123	cellular extravasation
BP	GO:0006869	lipid transport
BP	GO:0002456	T cell mediated immunity
BP	GO:1904994	regulation of leukocyte adhesion to vascular endothelial cel
BP	GO:0046631	alpha-beta T cell activation
BP	GO:0032722	positive regulation of chemokine production
BP	GO:0071230	cellular response to amino acid stimulus
BP	GO:0050708	regulation of protein secretion
BP	GO:0050866	negative regulation of cell activation
BP	GO:0001938	positive regulation of endothelial cell proliferation
BP	GO:0014066	regulation of phosphatidylinositol 3-kinase signaling
BP	GO:0042113	B cell activation
BP	GO:0000271	polysaccharide biosynthetic process
BP	GO:0071479	cellular response to ionizing radiation
BP	GO:0032733	positive regulation of interleukin-10 production
BP	GO:1900371	regulation of purine nucleotide biosynthetic process
BP	GO:0010544	negative regulation of platelet activation
BP	GO:1901222	regulation of NIK/NF-kappaB signaling
BP	GO:0032386	regulation of intracellular transport
BP	GO:0043271	negative regulation of ion transport
BP	GO:0060402	calcium ion transport into cytosol
BP	GO:0090316	positive regulation of intracellular protein transport
BP	GO:0030808	regulation of nucleotide biosynthetic process
BP	GO:0046688	response to copper ion
BP	GO:0046323	glucose import
BP	GO:1903201	regulation of oxidative stress-induced cell death
BP	GO:0051100	negative regulation of binding
BP	GO:0043279	response to alkaloid
BP	GO:0070374	positive regulation of ERK1 and ERK2 cascade
BP	GO:0002292	T cell differentiation involved in immune response
BP	GO:0043627	response to estrogen
BP	GO:0045913	positive regulation of carbohydrate metabolic process
BP	GO:0014002	astrocyte development
BP	GO:0045840	positive regulation of mitotic nuclear division
BP	GO:0060443	mammary gland morphogenesis
BP	GO:0140353	lipid export from cell
BP	GO:0009110	vitamin biosynthetic process
BP	GO:1902004	positive regulation of amyloid-beta formation
BP	GO:0009117	nucleotide metabolic process
BP	GO:0008286	insulin receptor signaling pathway
BP	GO:0021782	glial cell development
BP	GO:0002700	regulation of production of molecular mediator of immune res[illegible]
BP	GO:0050680	negative regulation of epithelial cell proliferation
BP	GO:0050806	positive regulation of synaptic transmission
BP	GO:0043542	endothelial cell migration
BP	GO:2001020	regulation of response to DNA damage stimulus
BP	GO:0072521	purine-containing compound metabolic process
BP	GO:0031100	animal organ regeneration
BP	GO:0043536	positive regulation of blood vessel endothelial cell migrati[illegible]
BP	GO:0032635	interleukin-6 production

BP	GO:0032675	regulation of interleukin-6 production
BP	GO:0072676	lymphocyte migration
BP	GO:0002064	epithelial cell development
BP	GO:0007249	I-kappaB kinase/NF-kappaB signaling
BP	GO:0005978	glycogen biosynthetic process
BP	GO:0006775	fat-soluble vitamin metabolic process
BP	GO:0009250	glucan biosynthetic process
BP	GO:0042554	superoxide anion generation
BP	GO:1903532	positive regulation of secretion by cell
BP	GO:1901361	organic cyclic compound catabolic process
BP	GO:0007254	JNK cascade
BP	GO:0006753	nucleoside phosphate metabolic process
BP	GO:0010906	regulation of glucose metabolic process
BP	GO:0038083	peptidyl-tyrosine autophosphorylation
BP	GO:0071354	cellular response to interleukin-6
BP	GO:1904646	cellular response to amyloid-beta
BP	GO:0071901	negative regulation of protein serine/threonine kinase activ
BP	GO:0014068	positive regulation of phosphatidylinositol 3-kinase signali
BP	GO:0006984	ER-nucleus signaling pathway
BP	GO:0046777	protein autophosphorylation
BP	GO:0051928	positive regulation of calcium ion transport
BP	GO:0071229	cellular response to acid chemical
BP	GO:0060444	branching involved in mammary gland duct morphogenesis
BP	GO:0033157	regulation of intracellular protein transport
BP	GO:0009306	protein secretion
BP	GO:2000146	negative regulation of cell motility
BP	GO:0051099	positive regulation of binding
BP	GO:0060968	regulation of gene silencing
BP	GO:0042311	vasodilation
BP	GO:0051972	regulation of telomerase activity
BP	GO:0035592	establishment of protein localization to extracellular regio
BP	GO:0048639	positive regulation of developmental growth
BP	GO:0045732	positive regulation of protein catabolic process
BP	GO:0002683	negative regulation of immune system process
BP	GO:1905897	regulation of response to endoplasmic reticulum stress
BP	GO:1905475	regulation of protein localization to membrane
BP	GO:0002673	regulation of acute inflammatory response
BP	GO:0090199	regulation of release of cytochrome c from mitochondria
BP	GO:0043534	blood vessel endothelial cell migration
BP	GO:0050767	regulation of neurogenesis
BP	GO:0043367	CD4-positive, alpha-beta T cell differentiation
BP	GO:0051899	membrane depolarization
BP	GO:0002363	alpha-beta T cell lineage commitment
BP	GO:0043369	CD4-positive or CD8-positive, alpha-beta T cell lineage comm
BP	GO:0051271	negative regulation of cellular component movement
BP	GO:0030225	macrophage differentiation
BP	GO:0070741	response to interleukin-6
BP	GO:0070849	response to epidermal growth factor
BP	GO:1904707	positive regulation of vascular associated smooth muscle cel
BP	GO:0030072	peptide hormone secretion
BP	GO:0071692	protein localization to extracellular region
BP	GO:0010950	positive regulation of endopeptidase activity
BP	GO:0051960	regulation of nervous system development
BP	GO:0042886	amide transport

BP	GO:0002709	regulation of T cell mediated immunity
BP	GO:0010507	negative regulation of autophagy
BP	GO:0042509	regulation of tyrosine phosphorylation of STAT protein
BP	GO:0034101	erythrocyte homeostasis
BP	GO:0045058	T cell selection
BP	GO:0002726	positive regulation of T cell cytokine production
BP	GO:0030810	positive regulation of nucleotide biosynthetic process
BP	GO:0036003	positive regulation of transcription from RNA polymerase II p
BP	GO:0051043	regulation of membrane protein ectodomain proteolysis
BP	GO:1900373	positive regulation of purine nucleotide biosynthetic process
BP	GO:0061138	morphogenesis of a branching epithelium
BP	GO:0006874	cellular calcium ion homeostasis
BP	GO:0002819	regulation of adaptive immune response
BP	GO:0010656	negative regulation of muscle cell apoptotic process
BP	GO:0014009	glial cell proliferation
BP	GO:0071622	regulation of granulocyte chemotaxis
BP	GO:0001776	leukocyte homeostasis
BP	GO:1903578	regulation of ATP metabolic process
BP	GO:0002790	peptide secretion
BP	GO:0035264	multicellular organism growth
BP	GO:0007260	tyrosine phosphorylation of STAT protein
BP	GO:0048143	astrocyte activation
BP	GO:1902993	positive regulation of amyloid precursor protein catabolic pr
BP	GO:0051047	positive regulation of secretion
BP	GO:0002705	positive regulation of leukocyte mediated immunity
BP	GO:1900407	regulation of cellular response to oxidative stress
BP	GO:0001906	cell killing
BP	GO:0001541	ovarian follicle development
BP	GO:1904705	regulation of vascular associated smooth muscle cell prolifer
BP	GO:0046394	carboxylic acid biosynthetic process
BP	GO:0055074	calcium ion homeostasis
BP	GO:0042326	negative regulation of phosphorylation
BP	GO:1900017	positive regulation of cytokine production involved in inflam
BP	GO:1990874	vascular associated smooth muscle cell proliferation
BP	GO:0071560	cellular response to transforming growth factor beta stimulus
BP	GO:0055123	digestive system development
BP	GO:0016053	organic acid biosynthetic process
BP	GO:0006631	fatty acid metabolic process
BP	GO:0007204	positive regulation of cytosolic calcium ion concentration
BP	GO:0045598	regulation of fat cell differentiation
BP	GO:0043331	response to dsRNA
BP	GO:0040013	negative regulation of locomotion
BP	GO:0007589	body fluid secretion
BP	GO:0007263	nitric oxide mediated signal transduction
BP	GO:0060333	interferon-gamma-mediated signaling pathway
BP	GO:0030316	osteoclast differentiation
BP	GO:0045185	maintenance of protein location
BP	GO:1901605	alpha-amino acid metabolic process
BP	GO:0071559	response to transforming growth factor beta
BP	GO:0002711	positive regulation of T cell mediated immunity
BP	GO:0071385	cellular response to glucocorticoid stimulus
BP	GO:0001763	morphogenesis of a branching structure
BP	GO:0043393	regulation of protein binding
BP	GO:0050764	regulation of phagocytosis

BP	GO:0045165	cell fate commitment
BP	GO:0034763	negative regulation of transmembrane transport
BP	GO:0010883	regulation of lipid storage
BP	GO:0051785	positive regulation of nuclear division
BP	GO:0000423	mitophagy
BP	GO:0002675	positive regulation of acute inflammatory response
BP	GO:0010575	positive regulation of vascular endothelial growth factor pr[illegible]
BP	GO:0031099	regeneration
BP	GO:0002763	positive regulation of myeloid leukocyte differentiation
BP	GO:0042743	hydrogen peroxide metabolic process
BP	GO:0043525	positive regulation of neuron apoptotic process
BP	GO:0033080	immature T cell proliferation in thymus
BP	GO:0045348	positive regulation of MHC class II biosynthetic process
BP	GO:0070391	response to lipoteichoic acid
BP	GO:0071223	cellular response to lipoteichoic acid
BP	GO:0032642	regulation of chemokine production
BP	GO:1902882	regulation of response to oxidative stress
BP	GO:0071549	cellular response to dexamethasone stimulus
BP	GO:1902175	regulation of oxidative stress-induced intrinsic apoptotic s[illegible]
BP	GO:0015833	peptide transport
BP	GO:0032388	positive regulation of intracellular transport
BP	GO:0043407	negative regulation of MAP kinase activity
BP	GO:0001889	liver development
BP	GO:0032602	chemokine production
BP	GO:0042100	B cell proliferation
BP	GO:0072503	cellular divalent inorganic cation homeostasis
BP	GO:2001056	positive regulation of cysteine-type endopeptidase activity
BP	GO:0019218	regulation of steroid metabolic process
BP	GO:0060603	mammary gland duct morphogenesis
BP	GO:0070498	interleukin-1-mediated signaling pathway
BP	GO:2000637	positive regulation of gene silencing by miRNA
BP	GO:0001655	urogenital system development
BP	GO:0061008	hepaticobiliary system development
BP	GO:0071384	cellular response to corticosteroid stimulus
BP	GO:0035710	CD4-positive, alpha-beta T cell activation
BP	GO:0045833	negative regulation of lipid metabolic process
BP	GO:0001933	negative regulation of protein phosphorylation
BP	GO:0002260	lymphocyte homeostasis
BP	GO:0010573	vascular endothelial growth factor production
BP	GO:0032613	interleukin-10 production
BP	GO:0032653	regulation of interleukin-10 production
BP	GO:0032731	positive regulation of interleukin-1 beta production
BP	GO:0019233	sensory perception of pain
BP	GO:0032760	positive regulation of tumor necrosis factor production
BP	GO:0046425	regulation of receptor signaling pathway via JAK-STAT
BP	GO:0060148	positive regulation of posttranscriptional gene silencing
BP	GO:0071480	cellular response to gamma radiation
BP	GO:0030656	regulation of vitamin metabolic process
BP	GO:0031652	positive regulation of heat generation
BP	GO:0033083	regulation of immature T cell proliferation
BP	GO:0042447	hormone catabolic process
BP	GO:1900222	negative regulation of amyloid-beta clearance
BP	GO:0030336	negative regulation of cell migration
BP	GO:1903076	regulation of protein localization to plasma membrane

BP	GO:0022617	extracellular matrix disassembly
BP	GO:0006469	negative regulation of protein kinase activity
BP	GO:0030888	regulation of B cell proliferation
BP	GO:0045670	regulation of osteoclast differentiation
BP	GO:0070542	response to fatty acid
BP	GO:0035821	modulation of process of other organism
BP	GO:0046822	regulation of nucleocytoplasmic transport
BP	GO:1905477	positive regulation of protein localization to membrane
BP	GO:2000278	regulation of DNA biosynthetic process
BP	GO:1902107	positive regulation of leukocyte differentiation
BP	GO:1903708	positive regulation of hemopoiesis
BP	GO:0045861	negative regulation of proteolysis
BP	GO:0002824	positive regulation of adaptive immune response based on soma
BP	GO:0005976	polysaccharide metabolic process
BP	GO:1903557	positive regulation of tumor necrosis factor superfamily cyto
BP	GO:0048568	embryonic organ development
BP	GO:0002720	positive regulation of cytokine production involved in immune
BP	GO:0032507	maintenance of protein location in cell
BP	GO:0033692	cellular polysaccharide biosynthetic process
BP	GO:0071677	positive regulation of mononuclear cell migration
BP	GO:1905330	regulation of morphogenesis of an epithelium
BP	GO:0051480	regulation of cytosolic calcium ion concentration
BP	GO:0010165	response to X-ray
BP	GO:0071353	cellular response to interleukin-4
BP	GO:1903146	regulation of autophagy of mitochondrion
BP	GO:0044262	cellular carbohydrate metabolic process
BP	GO:0051146	striated muscle cell differentiation
BP	GO:0042093	T-helper cell differentiation
BP	GO:0072678	T cell migration
BP	GO:1905953	negative regulation of lipid localization
BP	GO:0015850	organic hydroxy compound transport
BP	GO:0033079	immature T cell proliferation
BP	GO:0072584	caveolin-mediated endocytosis
BP	GO:0072683	T cell extravasation
BP	GO:1903599	positive regulation of autophagy of mitochondrion
BP	GO:1903800	positive regulation of production of miRNAs involved in gene
BP	GO:0001818	negative regulation of cytokine production
BP	GO:1904062	regulation of cation transmembrane transport
BP	GO:0042391	regulation of membrane potential
BP	GO:0032611	interleukin-1 beta production
BP	GO:0032651	regulation of interleukin-1 beta production
BP	GO:0009112	nucleobase metabolic process
BP	GO:0016242	negative regulation of macroautophagy
BP	GO:0043276	anoikis
BP	GO:0042531	positive regulation of tyrosine phosphorylation of STAT prote
BP	GO:0042698	ovulation cycle
BP	GO:0051926	negative regulation of calcium ion transport
BP	GO:0061912	selective autophagy
BP	GO:0002821	positive regulation of adaptive immune response
BP	GO:0045454	cell redox homeostasis
BP	GO:0097421	liver regeneration
BP	GO:1905898	positive regulation of response to endoplasmic reticulum stre
BP	GO:0050769	positive regulation of neurogenesis
BP	GO:0033344	cholesterol efflux

BP	GO:0002708	positive regulation of lymphocyte mediated immunity
BP	GO:0030307	positive regulation of cell growth
BP	GO:0006983	ER overload response
BP	GO:0007494	midgut development
BP	GO:0035745	T-helper 2 cell cytokine production
BP	GO:0051709	regulation of killing of cells of other organism
BP	GO:2000551	regulation of T-helper 2 cell cytokine production
BP	GO:1904892	regulation of receptor signaling pathway via STAT
BP	GO:0009150	purine ribonucleotide metabolic process
BP	GO:0070670	response to interleukin-4
BP	GO:0090322	regulation of superoxide metabolic process
BP	GO:0002706	regulation of lymphocyte mediated immunity
BP	GO:0002822	regulation of adaptive immune response based on somatic recoı
BP	GO:0001649	osteoblast differentiation
BP	GO:0018107	peptidyl-threonine phosphorylation
BP	GO:0010720	positive regulation of cell development
BP	GO:0030301	cholesterol transport
BP	GO:0002369	T cell cytokine production
BP	GO:0002724	regulation of T cell cytokine production
BP	GO:0043029	T cell homeostasis
BP	GO:0043368	positive T cell selection
BP	GO:0005977	glycogen metabolic process
BP	GO:0006073	cellular glucan metabolic process
BP	GO:0032732	positive regulation of interleukin-1 production
BP	GO:0035924	cellular response to vascular endothelial growth factor stimı
BP	GO:0044042	glucan metabolic process
BP	GO:0010469	regulation of signaling receptor activity
BP	GO:0031650	regulation of heat generation
BP	GO:1903894	regulation of IRE1-mediated unfolded protein response
BP	GO:0071392	cellular response to estradiol stimulus
BP	GO:1903580	positive regulation of ATP metabolic process
BP	GO:0030218	erythrocyte differentiation
BP	GO:0033673	negative regulation of kinase activity
BP	GO:0038127	ERBB signaling pathway
BP	GO:0043500	muscle adaptation
BP	GO:0046326	positive regulation of glucose import
BP	GO:0001937	negative regulation of endothelial cell proliferation
BP	GO:0014823	response to activity
BP	GO:0009259	ribonucleotide metabolic process
BP	GO:0045786	negative regulation of cell cycle
BP	GO:0032310	prostaglandin secretion
BP	GO:0045346	regulation of MHC class II biosynthetic process
BP	GO:0045725	positive regulation of glycogen biosynthetic process
BP	GO:0090594	inflammatory response to wounding
BP	GO:0051281	positive regulation of release of sequestered calcium ion in
BP	GO:0070266	necroptotic process
BP	GO:0031016	pancreas development
BP	GO:0010721	negative regulation of cell development
BP	GO:0043409	negative regulation of MAPK cascade
BP	GO:0045787	positive regulation of cell cycle
BP	GO:0032640	tumor necrosis factor production
BP	GO:0032680	regulation of tumor necrosis factor production
BP	GO:0018210	peptidyl-threonine modification
BP	GO:1904375	regulation of protein localization to cell periphery

BP	GO:0006919	activation of cysteine-type endopeptidase activity involved
BP	GO:0009064	glutamine family amino acid metabolic process
BP	GO:0034637	cellular carbohydrate biosynthetic process
BP	GO:0043154	negative regulation of cysteine-type endopeptidase activity
BP	GO:0010907	positive regulation of glucose metabolic process
BP	GO:0042307	positive regulation of protein import into nucleus
BP	GO:0055023	positive regulation of cardiac muscle tissue growth
BP	GO:2001235	positive regulation of apoptotic signaling pathway
BP	GO:0019058	viral life cycle
BP	GO:0048565	digestive tract development
BP	GO:0043122	regulation of I-kappaB kinase/NF-kappaB signaling
BP	GO:0019693	ribose phosphate metabolic process
BP	GO:0032612	interleukin-1 production
BP	GO:0032652	regulation of interleukin-1 production
BP	GO:0002830	positive regulation of type 2 immune response
BP	GO:0006206	pyrimidine nucleobase metabolic process
BP	GO:0009299	mRNA transcription
BP	GO:0045342	MHC class II biosynthetic process
BP	GO:0070431	nucleotide-binding oligomerization domain containing 2 signa
BP	GO:1902931	negative regulation of alcohol biosynthetic process
BP	GO:0033574	response to testosterone
BP	GO:0072595	maintenance of protein localization in organelle
BP	GO:0009791	post-embryonic development
BP	GO:0008217	regulation of blood pressure
BP	GO:0071706	tumor necrosis factor superfamily cytokine production
BP	GO:1903555	regulation of tumor necrosis factor superfamily cytokine pro
BP	GO:0043280	positive regulation of cysteine-type endopeptidase activity
BP	GO:0000422	autophagy of mitochondrion
BP	GO:0061726	mitochondrion disassembly
BP	GO:0015918	sterol transport
BP	GO:0006509	membrane protein ectodomain proteolysis
BP	GO:0035794	positive regulation of mitochondrial membrane permeability
BP	GO:1904591	positive regulation of protein import
BP	GO:0000723	telomere maintenance
BP	GO:0009165	nucleotide biosynthetic process
BP	GO:0090596	sensory organ morphogenesis
BP	GO:1901293	nucleoside phosphate biosynthetic process
BP	GO:0046427	positive regulation of receptor signaling pathway via JAK-ST
BP	GO:0060324	face development
BP	GO:0070875	positive regulation of glycogen metabolic process
BP	GO:1900034	regulation of cellular response to heat
BP	GO:2000696	regulation of epithelial cell differentiation involved in ki
BP	GO:0006164	purine nucleotide biosynthetic process
BP	GO:0046328	regulation of JNK cascade
BP	GO:0006112	energy reserve metabolic process
BP	GO:0006970	response to osmotic stress
BP	GO:0098586	cellular response to virus
BP	GO:0042593	glucose homeostasis
BP	GO:0034765	regulation of ion transmembrane transport
BP	GO:0033500	carbohydrate homeostasis
BP	GO:0060421	positive regulation of heart growth
BP	GO:1902108	regulation of mitochondrial membrane permeability involved i
BP	GO:2000273	positive regulation of signaling receptor activity
BP	GO:0032092	positive regulation of protein binding

BP	GO:0030073	insulin secretion
BP	GO:0003158	endothelium development
BP	GO:0045727	positive regulation of translation
BP	GO:0031330	negative regulation of cellular catabolic process
BP	GO:2000117	negative regulation of cysteine-type endopeptidase activity
BP	GO:0022408	negative regulation of cell-cell adhesion
BP	GO:0090276	regulation of peptide hormone secretion
BP	GO:0010828	positive regulation of glucose transmembrane transport
BP	GO:0031018	endocrine pancreas development
BP	GO:0031294	lymphocyte costimulation
BP	GO:0034198	cellular response to amino acid starvation
BP	GO:0061028	establishment of endothelial barrier
BP	GO:0006986	response to unfolded protein
BP	GO:0002295	T-helper cell lineage commitment
BP	GO:0002544	chronic inflammatory response
BP	GO:0015732	prostaglandin transport
BP	GO:0036499	PERK-mediated unfolded protein response
BP	GO:0046112	nucleobase biosynthetic process
BP	GO:0048643	positive regulation of skeletal muscle tissue development
BP	GO:0150078	positive regulation of neuroinflammatory response
BP	GO:1902074	response to salt
BP	GO:1903209	positive regulation of oxidative stress-induced cell death
BP	GO:1901606	alpha-amino acid catabolic process
BP	GO:0007179	transforming growth factor beta receptor signaling pathway
BP	GO:0001774	microglial cell activation
BP	GO:0007595	lactation
BP	GO:0097300	programmed necrotic cell death
BP	GO:0050829	defense response to Gram-negative bacterium
BP	GO:0070098	chemokine-mediated signaling pathway
BP	GO:0002791	regulation of peptide secretion
BP	GO:0072522	purine-containing compound biosynthetic process
BP	GO:0046165	alcohol biosynthetic process
BP	GO:0050768	negative regulation of neurogenesis
BP	GO:0001894	tissue homeostasis
BP	GO:0008584	male gonad development
BP	GO:1903038	negative regulation of leukocyte cell-cell adhesion
BP	GO:0042149	cellular response to glucose starvation
BP	GO:1905710	positive regulation of membrane permeability
BP	GO:2001238	positive regulation of extrinsic apoptotic signaling pathway
BP	GO:0036498	IRE1-mediated unfolded protein response
BP	GO:1901739	regulation of myoblast fusion
BP	GO:0016051	carbohydrate biosynthetic process
BP	GO:0090087	regulation of peptide transport
BP	GO:0046546	development of primary male sexual characteristics
BP	GO:2000045	regulation of G1/S transition of mitotic cell cycle
BP	GO:0051701	biological process involved in interaction with host
BP	GO:0051962	positive regulation of nervous system development
BP	GO:0010823	negative regulation of mitochondrion organization
BP	GO:0014075	response to amine
BP	GO:0030857	negative regulation of epithelial cell differentiation
BP	GO:0043330	response to exogenous dsRNA
BP	GO:0043457	regulation of cellular respiration
BP	GO:1904894	positive regulation of receptor signaling pathway via STAT
BP	GO:1990928	response to amino acid starvation

BP	GO:0007612	learning
BP	GO:0048863	stem cell differentiation
BP	GO:0002449	lymphocyte mediated immunity
BP	GO:0043524	negative regulation of neuron apoptotic process
BP	GO:0051961	negative regulation of nervous system development
BP	GO:0010665	regulation of cardiac muscle cell apoptotic process
BP	GO:0070231	T cell apoptotic process
BP	GO:1902930	regulation of alcohol biosynthetic process
BP	GO:0006525	arginine metabolic process
BP	GO:0032495	response to muramyl dipeptide
BP	GO:0045844	positive regulation of striated muscle tissue development
BP	GO:0048636	positive regulation of muscle organ development
BP	GO:0051900	regulation of mitochondrial depolarization
BP	GO:0045580	regulation of T cell differentiation
BP	GO:0019217	regulation of fatty acid metabolic process
BP	GO:0046034	ATP metabolic process
BP	GO:0006066	alcohol metabolic process
BP	GO:0032091	negative regulation of protein binding
BP	GO:0007178	transmembrane receptor protein serine/threonine kinase signa
BP	GO:0030100	regulation of endocytosis
BP	GO:0042180	cellular ketone metabolic process
BP	GO:0010662	regulation of striated muscle cell apoptotic process
BP	GO:0048260	positive regulation of receptor-mediated endocytosis
BP	GO:0007252	I-kappaB phosphorylation
BP	GO:0043373	CD4-positive, alpha-beta T cell lineage commitment
BP	GO:0071498	cellular response to fluid shear stress
BP	GO:1901028	regulation of mitochondrial outer membrane permeabilization
BP	GO:1901863	positive regulation of muscle tissue development
BP	GO:0034620	cellular response to unfolded protein
BP	GO:0042632	cholesterol homeostasis
BP	GO:0043648	dicarboxylic acid metabolic process
BP	GO:0044264	cellular polysaccharide metabolic process
BP	GO:0006520	cellular amino acid metabolic process
BP	GO:0030902	hindbrain development
BP	GO:1904064	positive regulation of cation transmembrane transport
BP	GO:0014909	smooth muscle cell migration
BP	GO:0055092	sterol homeostasis
BP	GO:1990868	response to chemokine
BP	GO:1990869	cellular response to chemokine
BP	GO:0034381	plasma lipoprotein particle clearance
BP	GO:0071320	cellular response to cAMP
BP	GO:0031056	regulation of histone modification
BP	GO:0070301	cellular response to hydrogen peroxide
BP	GO:0010659	cardiac muscle cell apoptotic process
BP	GO:0030520	intracellular estrogen receptor signaling pathway
BP	GO:0045540	regulation of cholesterol biosynthetic process
BP	GO:0061042	vascular wound healing
BP	GO:0106118	regulation of sterol biosynthetic process
BP	GO:0140467	integrated stress response signaling
BP	GO:1901522	positive regulation of transcription from RNA polymerase II
BP	GO:0033209	tumor necrosis factor-mediated signaling pathway
BP	GO:0042445	hormone metabolic process
BP	GO:0045088	regulation of innate immune response
BP	GO:0001701	in utero embryonic development

BP	GO:1903364	positive regulation of cellular protein catabolic process
BP	GO:0006641	triglyceride metabolic process
BP	GO:0045807	positive regulation of endocytosis
BP	GO:0010676	positive regulation of cellular carbohydrate metabolic proces
BP	GO:0048016	inositol phosphate-mediated signaling
BP	GO:0035051	cardiocyte differentiation
BP	GO:0001822	kidney development
BP	GO:1990845	adaptive thermogenesis
BP	GO:0010888	negative regulation of lipid storage
BP	GO:0019430	removal of superoxide radicals
BP	GO:0051882	mitochondrial depolarization
BP	GO:0060353	regulation of cell adhesion molecule production
BP	GO:0090335	regulation of brown fat cell differentiation
BP	GO:2001169	regulation of ATP biosynthetic process
BP	GO:0010658	striated muscle cell apoptotic process
BP	GO:0043388	positive regulation of DNA binding
BP	GO:0043470	regulation of carbohydrate catabolic process
BP	GO:0002274	myeloid leukocyte activation
BP	GO:0032200	telomere organization
BP	GO:0035966	response to topologically incorrect protein
BP	GO:1903169	regulation of calcium ion transmembrane transport
BP	GO:0030593	neutrophil chemotaxis
BP	GO:0006695	cholesterol biosynthetic process
BP	GO:0033619	membrane protein proteolysis
BP	GO:0051155	positive regulation of striated muscle cell differentiation
BP	GO:1902653	secondary alcohol biosynthetic process
BP	GO:0016485	protein processing
BP	GO:0032963	collagen metabolic process
BP	GO:0030878	thyroid gland development
BP	GO:0032928	regulation of superoxide anion generation
BP	GO:0048011	neurotrophin TRK receptor signaling pathway
BP	GO:0048641	regulation of skeletal muscle tissue development
BP	GO:0051000	positive regulation of nitric-oxide synthase activity
BP	GO:2000679	positive regulation of transcription regulatory region DNA b
BP	GO:0010043	response to zinc ion
BP	GO:0010574	regulation of vascular endothelial growth factor production
BP	GO:0032481	positive regulation of type I interferon production
BP	GO:0060759	regulation of response to cytokine stimulus
BP	GO:0072001	renal system development
BP	GO:0006633	fatty acid biosynthetic process
BP	GO:0045444	fat cell differentiation
BP	GO:0006090	pyruvate metabolic process
BP	GO:0006766	vitamin metabolic process
BP	GO:0042116	macrophage activation
BP	GO:0007162	negative regulation of cell adhesion
BP	GO:0048010	vascular endothelial growth factor receptor signaling pathwa
BP	GO:0045333	cellular respiration
BP	GO:0002764	immune response-regulating signaling pathway
BP	GO:0042692	muscle cell differentiation
BP	GO:0042303	molting cycle
BP	GO:0042633	hair cycle
BP	GO:0046661	male sex differentiation
BP	GO:0050796	regulation of insulin secretion
BP	GO:0032469	endoplasmic reticulum calcium ion homeostasis

BP	GO:0050927	positive regulation of positive chemotaxis
BP	GO:0050995	negative regulation of lipid catabolic process
BP	GO:0070423	nucleotide-binding oligomerization domain containing signali[illegible]
BP	GO:0071450	cellular response to oxygen radical
BP	GO:0071451	cellular response to superoxide
BP	GO:0031638	zymogen activation
BP	GO:0042306	regulation of protein import into nucleus
BP	GO:0043112	receptor metabolic process
BP	GO:0060538	skeletal muscle organ development
BP	GO:0007173	epidermal growth factor receptor signaling pathway
BP	GO:0022037	metencephalon development
BP	GO:0002440	production of molecular mediator of immune response
BP	GO:0034767	positive regulation of ion transmembrane transport
BP	GO:0034766	negative regulation of ion transmembrane transport
BP	GO:0019748	secondary metabolic process
BP	GO:0040014	regulation of multicellular organism growth
BP	GO:0050994	regulation of lipid catabolic process
BP	GO:2000401	regulation of lymphocyte migration
BP	GO:1902806	regulation of cell cycle G1/S phase transition
BP	GO:0046395	carboxylic acid catabolic process
BP	GO:0090068	positive regulation of cell cycle process
BP	GO:0007088	regulation of mitotic nuclear division
BP	GO:0009063	cellular amino acid catabolic process
BP	GO:0014812	muscle cell migration
BP	GO:0010875	positive regulation of cholesterol efflux
BP	GO:0033598	mammary gland epithelial cell proliferation
BP	GO:0035872	nucleotide-binding domain, leucine rich repeat containing re[illegible]
BP	GO:0050926	regulation of positive chemotaxis
BP	GO:0060352	cell adhesion molecule production
BP	GO:0070265	necrotic cell death
BP	GO:1903078	positive regulation of protein localization to plasma membra[illegible]
BP	GO:0060249	anatomical structure homeostasis
BP	GO:0032609	interferon-gamma production
BP	GO:0032649	regulation of interferon-gamma production
BP	GO:0031343	positive regulation of cell killing
BP	GO:1904589	regulation of protein import
BP	GO:0016054	organic acid catabolic process
BP	GO:0002221	pattern recognition receptor signaling pathway
BP	GO:0010894	negative regulation of steroid biosynthetic process
BP	GO:0034377	plasma lipoprotein particle assembly
BP	GO:0060142	regulation of syncytium formation by plasma membrane fusion
BP	GO:0071624	positive regulation of granulocyte chemotaxis
BP	GO:0001885	endothelial cell development
BP	GO:0006940	regulation of smooth muscle contraction
BP	GO:0016126	sterol biosynthetic process
BP	GO:1900076	regulation of cellular response to insulin stimulus
BP	GO:1903409	reactive oxygen species biosynthetic process
BP	GO:0045619	regulation of lymphocyte differentiation
BP	GO:0002244	hematopoietic progenitor cell differentiation
BP	GO:1903008	organelle disassembly
BP	GO:0022900	electron transport chain
BP	GO:0006898	receptor-mediated endocytosis
BP	GO:0042471	ear morphogenesis
BP	GO:0050728	negative regulation of inflammatory response

BP	GO:0000303	response to superoxide
BP	GO:0009065	glutamine family amino acid catabolic process
BP	GO:0090200	positive regulation of release of cytochrome c from mitochon
BP	GO:0035967	cellular response to topologically incorrect protein
BP	GO:0046626	regulation of insulin receptor signaling pathway
BP	GO:0050918	positive chemotaxis
BP	GO:2000573	positive regulation of DNA biosynthetic process
BP	GO:0002702	positive regulation of production of molecular mediator of in
BP	GO:0014902	myotube differentiation
BP	GO:0007613	memory
BP	GO:0045446	endothelial cell differentiation
BP	GO:0000305	response to oxygen radical
BP	GO:0002068	glandular epithelial cell development
BP	GO:0035066	positive regulation of histone acetylation
BP	GO:0062098	regulation of programmed necrotic cell death
BP	GO:0072539	T-helper 17 cell differentiation
BP	GO:0071897	DNA biosynthetic process
BP	GO:0008361	regulation of cell size
BP	GO:0031640	killing of cells of other organism
BP	GO:1901607	alpha-amino acid biosynthetic process
BP	GO:0090257	regulation of muscle system process
BP	GO:0009743	response to carbohydrate
BP	GO:0034694	response to prostaglandin
BP	GO:0045939	negative regulation of steroid metabolic process
BP	GO:0045940	positive regulation of steroid metabolic process
BP	GO:0051968	positive regulation of synaptic transmission, glutamatergic
BP	GO:1900101	regulation of endoplasmic reticulum unfolded protein respons
BP	GO:1903579	negative regulation of ATP metabolic process
BP	GO:0051966	regulation of synaptic transmission, glutamatergic
BP	GO:1904377	positive regulation of protein localization to cell periphe
BP	GO:0002224	toll-like receptor signaling pathway
BP	GO:0042177	negative regulation of protein catabolic process
BP	GO:0042752	regulation of circadian rhythm
BP	GO:1903362	regulation of cellular protein catabolic process
BP	GO:1990266	neutrophil migration
BP	GO:1903522	regulation of blood circulation
BP	GO:0002548	monocyte chemotaxis
BP	GO:0001516	prostaglandin biosynthetic process
BP	GO:0001782	B cell homeostasis
BP	GO:0002230	positive regulation of defense response to virus by host
BP	GO:0002828	regulation of type 2 immune response
BP	GO:0030212	hyaluronan metabolic process
BP	GO:0046457	prostanoid biosynthetic process
BP	GO:0060055	angiogenesis involved in wound healing
BP	GO:0065005	protein-lipid complex assembly
BP	GO:2000144	positive regulation of DNA-templated transcription, initiati
BP	GO:0006575	cellular modified amino acid metabolic process
BP	GO:0051053	negative regulation of DNA metabolic process
BP	GO:0032729	positive regulation of interferon-gamma production
BP	GO:0055021	regulation of cardiac muscle tissue growth
BP	GO:0070227	lymphocyte apoptotic process
BP	GO:0032874	positive regulation of stress-activated MAPK cascade
BP	GO:0010743	regulation of macrophage derived foam cell differentiation
BP	GO:0097345	mitochondrial outer membrane permeabilization

BP	GO:2000191	regulation of fatty acid transport
BP	GO:0002637	regulation of immunoglobulin production
BP	GO:0042058	regulation of epidermal growth factor receptor signaling patl
BP	GO:1904427	positive regulation of calcium ion transmembrane transport
BP	GO:0006639	acylglycerol metabolic process
BP	GO:0070304	positive regulation of stress-activated protein kinase signa
BP	GO:0002437	inflammatory response to antigenic stimulus
BP	GO:0030968	endoplasmic reticulum unfolded protein response
BP	GO:0045648	positive regulation of erythrocyte differentiation
BP	GO:0050901	leukocyte tethering or rolling
BP	GO:0110110	positive regulation of animal organ morphogenesis
BP	GO:0006638	neutral lipid metabolic process
BP	GO:0006282	regulation of DNA repair
BP	GO:0008652	cellular amino acid biosynthetic process
BP	GO:0045685	regulation of glial cell differentiation
BP	GO:0001662	behavioral fear response
BP	GO:0010661	positive regulation of muscle cell apoptotic process
BP	GO:0014072	response to isoquinoline alkaloid
BP	GO:0034383	low-density lipoprotein particle clearance
BP	GO:0043278	response to morphine
BP	GO:0051973	positive regulation of telomerase activity
BP	GO:0055094	response to lipoprotein particle
BP	GO:0070884	regulation of calcineurin-NFAT signaling cascade
BP	GO:2000758	positive regulation of peptidyl-lysine acetylation
BP	GO:0050864	regulation of B cell activation
BP	GO:1901983	regulation of protein acetylation
BP	GO:0010565	regulation of cellular ketone metabolic process
BP	GO:0002209	behavioral defense response
BP	GO:0010664	negative regulation of striated muscle cell apoptotic proces
BP	GO:0046627	negative regulation of insulin receptor signaling pathway
BP	GO:0090050	positive regulation of cell migration involved in sprouting
BP	GO:0090181	regulation of cholesterol metabolic process
BP	GO:0106056	regulation of calcineurin-mediated signaling
BP	GO:0032371	regulation of sterol transport
BP	GO:0032374	regulation of cholesterol transport
BP	GO:0051279	regulation of release of sequestered calcium ion into cytoso
BP	GO:0060420	regulation of heart growth
BP	GO:0061844	antimicrobial humoral immune response mediated by antimicrob
BP	GO:1901184	regulation of ERBB signaling pathway
BP	GO:0002691	regulation of cellular extravasation
BP	GO:0038179	neurotrophin signaling pathway
BP	GO:0042092	type 2 immune response
BP	GO:0045742	positive regulation of epidermal growth factor receptor sign
BP	GO:0071402	cellular response to lipoprotein particle stimulus
BP	GO:0007422	peripheral nervous system development
BP	GO:0032204	regulation of telomere maintenance
BP	GO:0034754	cellular hormone metabolic process
BP	GO:0003012	muscle system process
BP	GO:0001942	hair follicle development
BP	GO:0006096	glycolytic process
BP	GO:0010833	telomere maintenance via telomere lengthening
BP	GO:0051149	positive regulation of muscle cell differentiation
BP	GO:0051817	modulation of process of other organism involved in symbioti
BP	GO:0016202	regulation of striated muscle tissue development

BP	GO:1900077	negative regulation of cellular response to insulin stimulus
BP	GO:0051783	regulation of nuclear division
BP	GO:0002312	B cell activation involved in immune response
BP	GO:0006757	ATP generation from ADP
BP	GO:0050886	endocrine process
BP	GO:0010613	positive regulation of cardiac muscle hypertrophy
BP	GO:0032373	positive regulation of sterol transport
BP	GO:0032376	positive regulation of cholesterol transport
BP	GO:0042596	fear response
BP	GO:0048246	macrophage chemotaxis
BP	GO:0071398	cellular response to fatty acid
BP	GO:0072538	T-helper 17 type immune response
BP	GO:1901186	positive regulation of ERBB signaling pathway
BP	GO:1902110	positive regulation of mitochondrial membrane permeability i
BP	GO:0010921	regulation of phosphatase activity
BP	GO:0022404	molting cycle process
BP	GO:0022405	hair cycle process
BP	GO:0042440	pigment metabolic process
BP	GO:0045445	myoblast differentiation
BP	GO:2000134	negative regulation of G1/S transition of mitotic cell cycle
BP	GO:0000050	urea cycle
BP	GO:0014041	regulation of neuron maturation
BP	GO:0031284	positive regulation of guanylate cyclase activity
BP	GO:0032025	response to cobalt ion
BP	GO:0032070	regulation of deoxyribonuclease activity
BP	GO:0045792	negative regulation of cell size
BP	GO:0045898	regulation of RNA polymerase II transcription preinitiation
BP	GO:0051974	negative regulation of telomerase activity
BP	GO:0060068	vagina development
BP	GO:0060439	trachea morphogenesis
BP	GO:0060736	prostate gland growth
BP	GO:0030258	lipid modification
BP	GO:0014742	positive regulation of muscle hypertrophy
BP	GO:0032728	positive regulation of interferon-beta production
BP	GO:1901861	regulation of muscle tissue development
BP	GO:0043583	ear development
BP	GO:0097006	regulation of plasma lipoprotein particle levels
BP	GO:0098773	skin epidermis development
BP	GO:0106106	cold-induced thermogenesis
BP	GO:0120161	regulation of cold-induced thermogenesis
BP	GO:0000082	G1/S transition of mitotic cell cycle
BP	GO:0072330	monocarboxylic acid biosynthetic process
BP	GO:0014031	mesenchymal cell development
BP	GO:0015908	fatty acid transport
BP	GO:0034103	regulation of tissue remodeling
BP	GO:0010863	positive regulation of phospholipase C activity
BP	GO:0014037	Schwann cell differentiation
BP	GO:0032094	response to food
BP	GO:0045740	positive regulation of DNA replication
BP	GO:1902686	mitochondrial outer membrane permeabilization involved in pr
BP	GO:2000142	regulation of DNA-templated transcription, initiation
BP	GO:0051604	protein maturation
BP	GO:1903531	negative regulation of secretion by cell
BP	GO:0002251	organ or tissue specific immune response

BP	GO:0048634	regulation of muscle organ development
BP	GO:0050691	regulation of defense response to virus by host
BP	GO:0014706	striated muscle tissue development
BP	GO:0016525	negative regulation of angiogenesis
BP	GO:0006188	IMP biosynthetic process
BP	GO:0006527	arginine catabolic process
BP	GO:0019048	modulation by virus of host process
BP	GO:0032308	positive regulation of prostaglandin secretion
BP	GO:0033148	positive regulation of intracellular estrogen receptor signa
BP	GO:0034379	very-low-density lipoprotein particle assembly
BP	GO:0035865	cellular response to potassium ion
BP	GO:0043619	regulation of transcription from RNA polymerase II promoter
BP	GO:0045945	positive regulation of transcription by RNA polymerase III
BP	GO:0071377	cellular response to glucagon stimulus
BP	GO:1901030	positive regulation of mitochondrial outer membrane permeabi
BP	GO:1902510	regulation of apoptotic DNA fragmentation
BP	GO:1903897	regulation of PERK-mediated unfolded protein response
BP	GO:1904181	positive regulation of membrane depolarization
BP	GO:1990440	positive regulation of transcription from RNA polymerase II
BP	GO:2000343	positive regulation of chemokine (C-X-C motif) ligand 2 produ
BP	GO:2000644	regulation of receptor catabolic process
BP	GO:0014910	regulation of smooth muscle cell migration
BP	GO:0046330	positive regulation of JNK cascade
BP	GO:0051781	positive regulation of cell division
BP	GO:0001959	regulation of cytokine-mediated signaling pathway
BP	GO:1903050	regulation of proteolysis involved in cellular protein catabo
BP	GO:0030198	extracellular matrix organization
BP	GO:0007520	myoblast fusion
BP	GO:0010939	regulation of necrotic cell death
BP	GO:0030574	collagen catabolic process
BP	GO:0030890	positive regulation of B cell proliferation
BP	GO:0043507	positive regulation of JUN kinase activity
BP	GO:0071312	cellular response to alkaloid
BP	GO:1900274	regulation of phospholipase C activity
BP	GO:1904037	positive regulation of epithelial cell apoptotic process
BP	GO:0055067	monovalent inorganic cation homeostasis
BP	GO:2000181	negative regulation of blood vessel morphogenesis
BP	GO:0032436	positive regulation of proteasomal ubiquitin-dependent prote
BP	GO:0046031	ADP metabolic process
BP	GO:0043062	extracellular structure organization
BP	GO:0060070	canonical Wnt signaling pathway
BP	GO:1901343	negative regulation of vasculature development
BP	GO:0002065	columnar/cuboidal epithelial cell differentiation
BP	GO:0006885	regulation of pH
BP	GO:0045229	external encapsulating structure organization
BP	GO:0090287	regulation of cellular response to growth factor stimulus
BP	GO:0034284	response to monosaccharide
BP	GO:0033173	calcineurin-NFAT signaling cascade
BP	GO:0042088	T-helper 1 type immune response
BP	GO:0042771	intrinsic apoptotic signaling pathway in response to DNA dama
BP	GO:0031058	positive regulation of histone modification
BP	GO:0035249	synaptic transmission, glutamatergic
BP	GO:0016052	carbohydrate catabolic process
BP	GO:0007519	skeletal muscle tissue development

BP	GO:0051147	regulation of muscle cell differentiation
BP	GO:0006909	phagocytosis
BP	GO:0090398	cellular senescence
BP	GO:1901796	regulation of signal transduction by p53 class mediator
BP	GO:1902807	negative regulation of cell cycle G1/S phase transition
BP	GO:0002066	columnar/cuboidal epithelial cell development
BP	GO:0031295	T cell costimulation
BP	GO:0032620	interleukin-17 production
BP	GO:0032660	regulation of interleukin-17 production
BP	GO:0045124	regulation of bone resorption
BP	GO:0046006	regulation of activated T cell proliferation
BP	GO:0048806	genitalia development
BP	GO:1903573	negative regulation of response to endoplasmic reticulum str
BP	GO:0010623	programmed cell death involved in cell development
BP	GO:0019627	urea metabolic process
BP	GO:0032306	regulation of prostaglandin secretion
BP	GO:0032736	positive regulation of interleukin-13 production
BP	GO:0033127	regulation of histone phosphorylation
BP	GO:0051549	positive regulation of keratinocyte migration
BP	GO:0061051	positive regulation of cell growth involved in cardiac muscl
BP	GO:0061307	cardiac neural crest cell differentiation involved in heart
BP	GO:0061308	cardiac neural crest cell development involved in heart deve
BP	GO:0061517	macrophage proliferation
BP	GO:0071941	nitrogen cycle metabolic process
BP	GO:0072182	regulation of nephron tubule epithelial cell differentiation
BP	GO:0090557	establishment of endothelial intestinal barrier
BP	GO:0140052	cellular response to oxidised low-density lipoprotein partic
BP	GO:1903624	regulation of DNA catabolic process
BP	GO:2000628	regulation of miRNA metabolic process
BP	GO:0002042	cell migration involved in sprouting angiogenesis
BP	GO:0051702	biological process involved in interaction with symbiont
BP	GO:1990830	cellular response to leukemia inhibitory factor
BP	GO:0001837	epithelial to mesenchymal transition
BP	GO:0003254	regulation of membrane depolarization
BP	GO:0006110	regulation of glycolytic process
BP	GO:0022602	ovulation cycle process
BP	GO:0032309	icosanoid secretion
BP	GO:0046189	phenol-containing compound biosynthetic process
BP	GO:0071364	cellular response to epidermal growth factor stimulus
BP	GO:1901985	positive regulation of protein acetylation
BP	GO:0008593	regulation of Notch signaling pathway
BP	GO:0032479	regulation of type I interferon production
BP	GO:0032606	type I interferon production
BP	GO:1903321	negative regulation of protein modification by small protein
BP	GO:1990823	response to leukemia inhibitory factor
BP	GO:0055017	cardiac muscle tissue growth
BP	GO:0060537	muscle tissue development
BP	GO:0007157	heterophilic cell-cell adhesion via plasma membrane cell adh
BP	GO:0010830	regulation of myotube differentiation
BP	GO:0071827	plasma lipoprotein particle organization
BP	GO:0048588	developmental cell growth
BP	GO:0120162	positive regulation of cold-induced thermogenesis
BP	GO:0045930	negative regulation of mitotic cell cycle
BP	GO:0014745	negative regulation of muscle adaptation

BP	GO:0031053	primary miRNA processing
BP	GO:0031282	regulation of guanylate cyclase activity
BP	GO:0031392	regulation of prostaglandin biosynthetic process
BP	GO:0032042	mitochondrial DNA metabolic process
BP	GO:0032494	response to peptidoglycan
BP	GO:0033145	positive regulation of intracellular steroid hormone recepto[illegible]
BP	GO:0061418	regulation of transcription from RNA polymerase II promoter [illegible]
BP	GO:1900103	positive regulation of endoplasmic reticulum unfolded protei[illegible]
BP	GO:1902947	regulation of tau-protein kinase activity
BP	GO:0043502	regulation of muscle adaptation
BP	GO:0035850	epithelial cell differentiation involved in kidney developme[illegible]
BP	GO:0045581	negative regulation of T cell differentiation
BP	GO:0045646	regulation of erythrocyte differentiation
BP	GO:0050798	activated T cell proliferation
BP	GO:0090279	regulation of calcium ion import
BP	GO:0101023	vascular endothelial cell proliferation
BP	GO:1905562	regulation of vascular endothelial cell proliferation
BP	GO:0072331	signal transduction by p53 class mediator
BP	GO:0002444	myeloid leukocyte mediated immunity
BP	GO:0006165	nucleoside diphosphate phosphorylation
BP	GO:0010717	regulation of epithelial to mesenchymal transition
BP	GO:0021549	cerebellum development
BP	GO:0051153	regulation of striated muscle cell differentiation
BP	GO:0050792	regulation of viral process
BP	GO:2000058	regulation of ubiquitin-dependent protein catabolic process
BP	GO:0002686	negative regulation of leukocyte migration
BP	GO:0045912	negative regulation of carbohydrate metabolic process
BP	GO:0090311	regulation of protein deacetylation
BP	GO:0097720	calcineurin-mediated signaling
BP	GO:0008544	epidermis development
BP	GO:0044843	cell cycle G1/S phase transition
BP	GO:0046939	nucleotide phosphorylation
BP	GO:1903320	regulation of protein modification by small protein conjugat[illegible]
BP	GO:0055088	lipid homeostasis
BP	GO:0006692	prostanoid metabolic process
BP	GO:0006693	prostaglandin metabolic process
BP	GO:0009409	response to cold
BP	GO:0030213	hyaluronan biosynthetic process
BP	GO:0034616	response to laminar fluid shear stress
BP	GO:0036444	calcium import into the mitochondrion
BP	GO:0038183	bile acid signaling pathway
BP	GO:0043374	CD8-positive, alpha-beta T cell differentiation
BP	GO:0051547	regulation of keratinocyte migration
BP	GO:0061043	regulation of vascular wound healing
BP	GO:0071236	cellular response to antibiotic
BP	GO:0072160	nephron tubule epithelial cell differentiation
BP	GO:0072540	T-helper 17 cell lineage commitment
BP	GO:0090281	negative regulation of calcium ion import
BP	GO:1901857	positive regulation of cellular respiration
BP	GO:1904294	positive regulation of ERAD pathway
BP	GO:2001171	positive regulation of ATP biosynthetic process
BP	GO:0007517	muscle organ development
BP	GO:1902106	negative regulation of leukocyte differentiation
BP	GO:1904063	negative regulation of cation transmembrane transport

BP	GO:0009152	purine ribonucleotide biosynthetic process
BP	GO:0009135	purine nucleoside diphosphate metabolic process
BP	GO:0009179	purine ribonucleoside diphosphate metabolic process
BP	GO:0030004	cellular monovalent inorganic cation homeostasis
BP	GO:0050873	brown fat cell differentiation
BP	GO:0060337	type I interferon signaling pathway
BP	GO:0060425	lung morphogenesis
BP	GO:0060688	regulation of morphogenesis of a branching structure
BP	GO:0071825	protein-lipid complex subunit organization
BP	GO:2000677	regulation of transcription regulatory region DNA binding
BP	GO:0046634	regulation of alpha-beta T cell activation
BP	GO:0060419	heart growth
BP	GO:0051048	negative regulation of secretion
BP	GO:0006636	unsaturated fatty acid biosynthetic process
BP	GO:0010874	regulation of cholesterol efflux
BP	GO:0043370	regulation of CD4-positive, alpha-beta T cell differentiatio
BP	GO:0045661	regulation of myoblast differentiation
BP	GO:0046850	regulation of bone remodeling
BP	GO:1990778	protein localization to cell periphery
BP	GO:0062207	regulation of pattern recognition receptor signaling pathway
BP	GO:0045926	negative regulation of growth
BP	GO:0006570	tyrosine metabolic process
BP	GO:0042159	lipoprotein catabolic process
BP	GO:0045064	T-helper 2 cell differentiation
BP	GO:0045986	negative regulation of smooth muscle contraction
BP	GO:0048308	organelle inheritance
BP	GO:0048313	Golgi inheritance
BP	GO:0070885	negative regulation of calcineurin-NFAT signaling cascade
BP	GO:0106057	negative regulation of calcineurin-mediated signaling
BP	GO:1900102	negative regulation of endoplasmic reticulum unfolded protei
BP	GO:1901741	positive regulation of myoblast fusion
BP	GO:2000402	negative regulation of lymphocyte migration
BP	GO:2001279	regulation of unsaturated fatty acid biosynthetic process
BP	GO:0009185	ribonucleoside diphosphate metabolic process
BP	GO:1903707	negative regulation of hemopoiesis
BP	GO:0010718	positive regulation of epithelial to mesenchymal transition
BP	GO:0032206	positive regulation of telomere maintenance
BP	GO:0043392	negative regulation of DNA binding
BP	GO:0071357	cellular response to type I interferon
BP	GO:0010951	negative regulation of endopeptidase activity
BP	GO:0006275	regulation of DNA replication
BP	GO:2000060	positive regulation of ubiquitin-dependent protein catabolic
BP	GO:0052126	movement in host environment
BP	GO:0060828	regulation of canonical Wnt signaling pathway
BP	GO:0071715	icosanoid transport
BP	GO:0051302	regulation of cell division
BP	GO:0035065	regulation of histone acetylation
BP	GO:0002693	positive regulation of cellular extravasation
BP	GO:0010225	response to UV-C
BP	GO:0030540	female genitalia development
BP	GO:0034116	positive regulation of heterotypic cell-cell adhesion
BP	GO:0034374	low-density lipoprotein particle remodeling
BP	GO:0035864	response to potassium ion
BP	GO:0045651	positive regulation of macrophage differentiation

BP	GO:0070242	thymocyte apoptotic process
BP	GO:0071391	cellular response to estrogen stimulus
BP	GO:0090336	positive regulation of brown fat cell differentiation
BP	GO:0097202	activation of cysteine-type endopeptidase activity
BP	GO:0002698	negative regulation of immune effector process
BP	GO:0006939	smooth muscle contraction
BP	GO:0018958	phenol-containing compound metabolic process
BP	GO:0048259	regulation of receptor-mediated endocytosis
BP	GO:0031348	negative regulation of defense response
BP	GO:0015718	monocarboxylic acid transport
BP	GO:0000768	syncytium formation by plasma membrane fusion
BP	GO:0002067	glandular epithelial cell differentiation
BP	GO:0003179	heart valve morphogenesis
BP	GO:0038066	p38MAPK cascade
BP	GO:0042220	response to cocaine
BP	GO:0045620	negative regulation of lymphocyte differentiation
BP	GO:0140253	cell-cell fusion
BP	GO:1905517	macrophage migration
BP	GO:0090150	establishment of protein localization to membrane
BP	GO:0009260	ribonucleotide biosynthetic process
BP	GO:0010466	negative regulation of peptidase activity
BP	GO:0010518	positive regulation of phospholipase activity
BP	GO:0031529	ruffle organization
BP	GO:0032608	interferon-beta production
BP	GO:0032648	regulation of interferon-beta production
BP	GO:0045599	negative regulation of fat cell differentiation
BP	GO:0046456	icosanoid biosynthetic process
BP	GO:0048008	platelet-derived growth factor receptor signaling pathway
BP	GO:0031623	receptor internalization
BP	GO:0044344	cellular response to fibroblast growth factor stimulus
BP	GO:0043588	skin development
BP	GO:0009084	glutamine family amino acid biosynthetic process
BP	GO:0010224	response to UV-B
BP	GO:0010831	positive regulation of myotube differentiation
BP	GO:0016264	gap junction assembly
BP	GO:0046827	positive regulation of protein export from nucleus
BP	GO:0060263	regulation of respiratory burst
BP	GO:0090399	replicative senescence
BP	GO:1901550	regulation of endothelial cell development
BP	GO:1903140	regulation of establishment of endothelial barrier
BP	GO:1903358	regulation of Golgi organization
BP	GO:1904355	positive regulation of telomere capping
BP	GO:2000811	negative regulation of anoikis
BP	GO:0010975	regulation of neuron projection development
BP	GO:2001257	regulation of cation channel activity
BP	GO:1901800	positive regulation of proteasomal protein catabolic process
BP	GO:0006754	ATP biosynthetic process
BP	GO:0006949	syncytium formation
BP	GO:0051205	protein insertion into membrane
BP	GO:0060760	positive regulation of response to cytokine stimulus
BP	GO:0061005	cell differentiation involved in kidney development
BP	GO:0030278	regulation of ossification
BP	GO:0051209	release of sequestered calcium ion into cytosol
BP	GO:0043123	positive regulation of I-kappaB kinase/NF-kappaB signaling

BP	GO:0030518	intracellular steroid hormone receptor signaling pathway
BP	GO:0033559	unsaturated fatty acid metabolic process
BP	GO:0051283	negative regulation of sequestering of calcium ion
BP	GO:0034340	response to type I interferon
BP	GO:0043506	regulation of JUN kinase activity
BP	GO:0043666	regulation of phosphoprotein phosphatase activity
BP	GO:2001244	positive regulation of intrinsic apoptotic signaling pathway
BP	GO:0002695	negative regulation of leukocyte activation
BP	GO:0061136	regulation of proteasomal protein catabolic process
BP	GO:0030308	negative regulation of cell growth
BP	GO:0009404	toxin metabolic process
BP	GO:0010663	positive regulation of striated muscle cell apoptotic process
BP	GO:0010666	positive regulation of cardiac muscle cell apoptotic process
BP	GO:0030730	sequestering of triglyceride
BP	GO:0032305	positive regulation of icosanoid secretion
BP	GO:0032769	negative regulation of monooxygenase activity
BP	GO:0034433	steroid esterification
BP	GO:0034434	sterol esterification
BP	GO:0034435	cholesterol esterification
BP	GO:0042136	neurotransmitter biosynthetic process
BP	GO:0043217	myelin maintenance
BP	GO:0046040	IMP metabolic process
BP	GO:0050665	hydrogen peroxide biosynthetic process
BP	GO:0060438	trachea development
BP	GO:0060546	negative regulation of necroptotic process
BP	GO:0060716	labyrinthine layer blood vessel development
BP	GO:0060749	mammary gland alveolus development
BP	GO:0061377	mammary gland lobule development
BP	GO:0071318	cellular response to ATP
BP	GO:2000641	regulation of early endosome to late endosome transport
BP	GO:0051055	negative regulation of lipid biosynthetic process
BP	GO:0051282	regulation of sequestering of calcium ion
BP	GO:0046390	ribose phosphate biosynthetic process
BP	GO:0002753	cytoplasmic pattern recognition receptor signaling pathway
BP	GO:0071774	response to fibroblast growth factor
BP	GO:0072655	establishment of protein localization to mitochondrion
BP	GO:0034113	heterotypic cell-cell adhesion
BP	GO:0043030	regulation of macrophage activation
BP	GO:1904356	regulation of telomere maintenance via telomere lengthening
BP	GO:0006007	glucose catabolic process
BP	GO:0006309	apoptotic DNA fragmentation
BP	GO:0006977	DNA damage response, signal transduction by p53 class mediat
BP	GO:0009168	purine ribonucleoside monophosphate biosynthetic process
BP	GO:0010744	positive regulation of macrophage derived foam cell differen
BP	GO:0010759	positive regulation of macrophage chemotaxis
BP	GO:0010829	negative regulation of glucose transmembrane transport
BP	GO:0030220	platelet formation
BP	GO:0032616	interleukin-13 production
BP	GO:0032656	regulation of interleukin-13 production
BP	GO:0036344	platelet morphogenesis
BP	GO:0044003	modulation by symbiont of host process
BP	GO:0045780	positive regulation of bone resorption
BP	GO:0045821	positive regulation of glycolytic process
BP	GO:0048245	eosinophil chemotaxis

BP	GO:0051546	keratinocyte migration
BP	GO:0060065	uterus development
BP	GO:0062099	negative regulation of programmed necrotic cell death
BP	GO:0019730	antimicrobial humoral response
BP	GO:0050868	negative regulation of T cell activation
BP	GO:0051208	sequestering of calcium ion
BP	GO:0022898	regulation of transmembrane transporter activity
BP	GO:0007588	excretion
BP	GO:0032623	interleukin-2 production
BP	GO:0032663	regulation of interleukin-2 production
BP	GO:0006690	icosanoid metabolic process
BP	GO:0061564	axon development
BP	GO:0006417	regulation of translation
BP	GO:2000756	regulation of peptidyl-lysine acetylation
BP	GO:0001909	leukocyte mediated cytotoxicity
BP	GO:0009132	nucleoside diphosphate metabolic process
BP	GO:0006851	mitochondrial calcium ion transmembrane transport
BP	GO:0009713	catechol-containing compound biosynthetic process
BP	GO:0010042	response to manganese ion
BP	GO:0010893	positive regulation of steroid biosynthetic process
BP	GO:0032930	positive regulation of superoxide anion generation
BP	GO:0042423	catecholamine biosynthetic process
BP	GO:0060231	mesenchymal to epithelial transition
BP	GO:0071243	cellular response to arsenic-containing substance
BP	GO:0071379	cellular response to prostaglandin stimulus
BP	GO:0072567	chemokine (C-X-C motif) ligand 2 production
BP	GO:0090201	negative regulation of release of cytochrome c from mitochon
BP	GO:1902176	negative regulation of oxidative stress-induced intrinsic ap
BP	GO:1902236	negative regulation of endoplasmic reticulum stress-induced
BP	GO:1904292	regulation of ERAD pathway
BP	GO:2000010	positive regulation of protein localization to cell surface
BP	GO:2000341	regulation of chemokine (C-X-C motif) ligand 2 production
BP	GO:0070585	protein localization to mitochondrion
BP	GO:2000027	regulation of animal organ morphogenesis
BP	GO:0006749	glutathione metabolic process
BP	GO:0046888	negative regulation of hormone secretion
BP	GO:0048247	lymphocyte chemotaxis
BP	GO:0002253	activation of immune response
BP	GO:0072659	protein localization to plasma membrane
BP	GO:0003170	heart valve development
BP	GO:0045453	bone resorption
BP	GO:0048645	animal organ formation
BP	GO:0017157	regulation of exocytosis
BP	GO:0051346	negative regulation of hydrolase activity
BP	GO:0000002	mitochondrial genome maintenance
BP	GO:0008090	retrograde axonal transport
BP	GO:0009127	purine nucleoside monophosphate biosynthetic process
BP	GO:0010878	cholesterol storage
BP	GO:0031998	regulation of fatty acid beta-oxidation
BP	GO:0032303	regulation of icosanoid secretion
BP	GO:0033762	response to glucagon
BP	GO:0050849	negative regulation of calcium-mediated signaling
BP	GO:0072111	cell proliferation involved in kidney development
BP	GO:0090026	positive regulation of monocyte chemotaxis

BP	GO:0097062	dendritic spine maintenance
BP	GO:1903429	regulation of cell maturation
BP	GO:2000178	negative regulation of neural precursor cell proliferation
BP	GO:0060541	respiratory system development
BP	GO:0001952	regulation of cell-matrix adhesion
BP	GO:0035303	regulation of dephosphorylation
BP	GO:0042446	hormone biosynthetic process
BP	GO:0045600	positive regulation of fat cell differentiation
BP	GO:0032535	regulation of cellular component size
BP	GO:0006879	cellular iron ion homeostasis
BP	GO:0010517	regulation of phospholipase activity
BP	GO:0032890	regulation of organic acid transport
BP	GO:2000514	regulation of CD4-positive, alpha-beta T cell activation
BP	GO:0019079	viral genome replication
BP	GO:1903844	regulation of cellular response to transforming growth facto
BP	GO:0002320	lymphoid progenitor cell differentiation
BP	GO:0006359	regulation of transcription by RNA polymerase III
BP	GO:0010310	regulation of hydrogen peroxide metabolic process
BP	GO:0032069	regulation of nuclease activity
BP	GO:0032700	negative regulation of interleukin-17 production
BP	GO:0032727	positive regulation of interferon-alpha production
BP	GO:0035584	calcium-mediated signaling using intracellular calcium sourc
BP	GO:0044346	fibroblast apoptotic process
BP	GO:0055093	response to hyperoxia
BP	GO:0071359	cellular response to dsRNA
BP	GO:1900543	negative regulation of purine nucleotide metabolic process
BP	GO:2000193	positive regulation of fatty acid transport
BP	GO:0009206	purine ribonucleoside triphosphate biosynthetic process
BP	GO:0046637	regulation of alpha-beta T cell differentiation
BP	GO:0046887	positive regulation of hormone secretion
BP	GO:1903052	positive regulation of proteolysis involved in cellular prot
BP	GO:0007004	telomere maintenance via telomerase
BP	GO:0009145	purine nucleoside triphosphate biosynthetic process
BP	GO:0010611	regulation of cardiac muscle hypertrophy
BP	GO:0050688	regulation of defense response to virus
BP	GO:0050805	negative regulation of synaptic transmission
BP	GO:0060193	positive regulation of lipase activity
BP	GO:0031396	regulation of protein ubiquitination
BP	GO:1901987	regulation of cell cycle phase transition
BP	GO:0032434	regulation of proteasomal ubiquitin-dependent protein catabo
BP	GO:0042552	myelination
BP	GO:0003323	type B pancreatic cell development
BP	GO:0006541	glutamine metabolic process
BP	GO:0033081	regulation of T cell differentiation in thymus
BP	GO:0035162	embryonic hemopoiesis
BP	GO:0042026	protein refolding
BP	GO:0045662	negative regulation of myoblast differentiation
BP	GO:0045723	positive regulation of fatty acid biosynthetic process
BP	GO:0045932	negative regulation of muscle contraction
BP	GO:0045943	positive regulation of transcription by RNA polymerase I
BP	GO:0045980	negative regulation of nucleotide metabolic process
BP	GO:0051349	positive regulation of lyase activity
BP	GO:0060143	positive regulation of syncytium formation by plasma membran
BP	GO:0060396	growth hormone receptor signaling pathway

BP	GO:0060547	negative regulation of necrotic cell death
BP	GO:0061050	regulation of cell growth involved in cardiac muscle cell dev
BP	GO:0072677	eosinophil migration
BP	GO:2000737	negative regulation of stem cell differentiation
BP	GO:1901990	regulation of mitotic cell cycle phase transition
BP	GO:0007272	ensheathment of neurons
BP	GO:0008366	axon ensheathment
BP	GO:0043401	steroid hormone mediated signaling pathway
BP	GO:0072073	kidney epithelium development
BP	GO:0033555	multicellular organismal response to stress
BP	GO:0050795	regulation of behavior
BP	GO:0090090	negative regulation of canonical Wnt signaling pathway
BP	GO:0014743	regulation of muscle hypertrophy
BP	GO:0043903	regulation of biological process involved in symbiotic intera
BP	GO:0050709	negative regulation of protein secretion
BP	GO:0002438	acute inflammatory response to antigenic stimulus
BP	GO:0002755	MyD88-dependent toll-like receptor signaling pathway
BP	GO:0002922	positive regulation of humoral immune response
BP	GO:0006582	melanin metabolic process
BP	GO:0010226	response to lithium ion
BP	GO:0010288	response to lead ion
BP	GO:0031639	plasminogen activation
BP	GO:0035458	cellular response to interferon-beta
BP	GO:0044321	response to leptin
BP	GO:0045649	regulation of macrophage differentiation
BP	GO:0051123	RNA polymerase II preinitiation complex assembly
BP	GO:0051156	glucose 6-phosphate metabolic process
BP	GO:0060561	apoptotic process involved in morphogenesis
BP	GO:0071378	cellular response to growth hormone stimulus
BP	GO:0071676	negative regulation of mononuclear cell migration
BP	GO:0090023	positive regulation of neutrophil chemotaxis
BP	GO:2000209	regulation of anoikis
BP	GO:2000310	regulation of NMDA receptor activity
BP	GO:2000353	positive regulation of endothelial cell apoptotic process
BP	GO:0045739	positive regulation of DNA repair
BP	GO:0050848	regulation of calcium-mediated signaling
BP	GO:0072088	nephron epithelium morphogenesis
BP	GO:0009746	response to hexose
BP	GO:0009201	ribonucleoside triphosphate biosynthetic process
BP	GO:0031333	negative regulation of protein-containing complex assembly
BP	GO:0032753	positive regulation of interleukin-4 production
BP	GO:0051204	protein insertion into mitochondrial membrane
BP	GO:0060261	positive regulation of transcription initiation from RNA pol
BP	GO:0060740	prostate gland epithelium morphogenesis
BP	GO:0090312	positive regulation of protein deacetylation
BP	GO:1901623	regulation of lymphocyte chemotaxis
BP	GO:1905564	positive regulation of vascular endothelial cell proliferati
BP	GO:0010810	regulation of cell-substrate adhesion
BP	GO:0032409	regulation of transporter activity
BP	GO:0007009	plasma membrane organization
BP	GO:0097553	calcium ion transmembrane import into cytosol
BP	GO:0006278	RNA-dependent DNA biosynthetic process
BP	GO:0034121	regulation of toll-like receptor signaling pathway
BP	GO:0048662	negative regulation of smooth muscle cell proliferation

BP	GO:2001259	positive regulation of cation channel activity
BP	GO:0070588	calcium ion transmembrane transport
BP	GO:0014855	striated muscle cell proliferation
BP	GO:0072028	nephron morphogenesis
BP	GO:0072332	intrinsic apoptotic signaling pathway by p53 class mediator
BP	GO:0001963	synaptic transmission, dopaminergic
BP	GO:0002092	positive regulation of receptor internalization
BP	GO:0032515	negative regulation of phosphoprotein phosphatase activity
BP	GO:0034114	regulation of heterotypic cell-cell adhesion
BP	GO:0045672	positive regulation of osteoclast differentiation
BP	GO:0060544	regulation of necroptotic process
BP	GO:0070102	interleukin-6-mediated signaling pathway
BP	GO:1904353	regulation of telomere capping
BP	GO:1905523	positive regulation of macrophage migration
BP	GO:0090049	regulation of cell migration involved in sprouting angiogenes
BP	GO:1901616	organic hydroxy compound catabolic process
BP	GO:0000737	DNA catabolic process, endonucleolytic
BP	GO:0002227	innate immune response in mucosa
BP	GO:0002719	negative regulation of cytokine production involved in immun
BP	GO:0009651	response to salt stress
BP	GO:0010586	miRNA metabolic process
BP	GO:0010758	regulation of macrophage chemotaxis
BP	GO:0036037	CD8-positive, alpha-beta T cell activation
BP	GO:0042104	positive regulation of activated T cell proliferation
BP	GO:0048873	homeostasis of number of cells within a tissue
BP	GO:0060512	prostate gland morphogenesis
BP	GO:1903203	regulation of oxidative stress-induced neuron death
BP	GO:1903900	regulation of viral life cycle
BP	GO:0007409	axonogenesis
BP	GO:0051592	response to calcium ion
BP	GO:0048145	regulation of fibroblast proliferation
BP	GO:0010818	T cell chemotaxis
BP	GO:0010884	positive regulation of lipid storage
BP	GO:0030262	apoptotic nuclear changes
BP	GO:0032607	interferon-alpha production
BP	GO:0032647	regulation of interferon-alpha production
BP	GO:0032801	receptor catabolic process
BP	GO:0045589	regulation of regulatory T cell differentiation
BP	GO:0045671	negative regulation of osteoclast differentiation
BP	GO:0060259	regulation of feeding behavior
BP	GO:0061082	myeloid leukocyte cytokine production
BP	GO:0071280	cellular response to copper ion
BP	GO:1902624	positive regulation of neutrophil migration
BP	GO:1903649	regulation of cytoplasmic transport
BP	GO:0007160	cell-matrix adhesion
BP	GO:0044409	entry into host
BP	GO:0014032	neural crest cell development
BP	GO:0030512	negative regulation of transforming growth factor beta recep
BP	GO:0030641	regulation of cellular pH
BP	GO:0048144	fibroblast proliferation
BP	GO:2001021	negative regulation of response to DNA damage stimulus
BP	GO:0060560	developmental growth involved in morphogenesis
BP	GO:0050871	positive regulation of B cell activation
BP	GO:0001892	embryonic placenta development

BP	GO:0001910	regulation of leukocyte mediated cytotoxicity
BP	GO:0009205	purine ribonucleoside triphosphate metabolic process
BP	GO:0072527	pyrimidine-containing compound metabolic process
BP	GO:2001252	positive regulation of chromosome organization
BP	GO:0030111	regulation of Wnt signaling pathway
BP	GO:0003309	type B pancreatic cell differentiation
BP	GO:0006734	NADH metabolic process
BP	GO:0019433	triglyceride catabolic process
BP	GO:0022011	myelination in peripheral nervous system
BP	GO:0032292	peripheral nervous system axon ensheathment
BP	GO:0033028	myeloid cell apoptotic process
BP	GO:0040018	positive regulation of multicellular organism growth
BP	GO:0043153	entrainment of circadian clock by photoperiod
BP	GO:0045737	positive regulation of cyclin-dependent protein serine/threo
BP	GO:0090025	regulation of monocyte chemotaxis
BP	GO:1900117	regulation of execution phase of apoptosis
BP	GO:1902230	negative regulation of intrinsic apoptotic signaling pathway
BP	GO:1902253	regulation of intrinsic apoptotic signaling pathway by p53 c
BP	GO:1902430	negative regulation of amyloid-beta formation
BP	GO:2000406	positive regulation of T cell migration
BP	GO:0008360	regulation of cell shape
BP	GO:0031397	negative regulation of protein ubiquitination
BP	GO:0048678	response to axon injury
BP	GO:0006099	tricarboxylic acid cycle
BP	GO:0006921	cellular component disassembly involved in execution phase o
BP	GO:0007202	activation of phospholipase C activity
BP	GO:0009072	aromatic amino acid family metabolic process
BP	GO:0019674	NAD metabolic process
BP	GO:0031571	mitotic G1 DNA damage checkpoint signaling
BP	GO:0033137	negative regulation of peptidyl-serine phosphorylation
BP	GO:0034368	protein-lipid complex remodeling
BP	GO:0034369	plasma lipoprotein particle remodeling
BP	GO:0034390	smooth muscle cell apoptotic process
BP	GO:0034391	regulation of smooth muscle cell apoptotic process
BP	GO:0036296	response to increased oxygen levels
BP	GO:0043304	regulation of mast cell degranulation
BP	GO:0045948	positive regulation of translational initiation
BP	GO:0046825	regulation of protein export from nucleus
BP	GO:0060218	hematopoietic stem cell differentiation
BP	GO:0060674	placenta blood vessel development
BP	GO:0090151	establishment of protein localization to mitochondrial membra
BP	GO:0051250	negative regulation of lymphocyte activation
BP	GO:0001656	metanephros development
BP	GO:0009142	nucleoside triphosphate biosynthetic process
BP	GO:0030433	ubiquitin-dependent ERAD pathway
BP	GO:0045069	regulation of viral genome replication
BP	GO:0055072	iron ion homeostasis
BP	GO:2000779	regulation of double-strand break repair
BP	GO:0048864	stem cell development
BP	GO:1903510	mucopolysaccharide metabolic process
BP	GO:0031063	regulation of histone deacetylation
BP	GO:0032148	activation of protein kinase B activity
BP	GO:0033006	regulation of mast cell activation involved in immune respons
BP	GO:0036475	neuron death in response to oxidative stress

BP	GO:0042133	neurotransmitter metabolic process
BP	GO:0044819	mitotic G1/S transition checkpoint signaling
BP	GO:0045066	regulatory T cell differentiation
BP	GO:0045987	positive regulation of smooth muscle contraction
BP	GO:0048566	embryonic digestive tract development
BP	GO:0048710	regulation of astrocyte differentiation
BP	GO:0060045	positive regulation of cardiac muscle cell proliferation
BP	GO:0070633	transepithelial transport
BP	GO:0090183	regulation of kidney development
BP	GO:1900027	regulation of ruffle assembly
BP	GO:0044070	regulation of anion transport
BP	GO:0150104	transport across blood-brain barrier
BP	GO:0003007	heart morphogenesis
BP	GO:0009144	purine nucleoside triphosphate metabolic process
BP	GO:0010232	vascular transport
BP	GO:0016055	Wnt signaling pathway
BP	GO:0003180	aortic valve morphogenesis
BP	GO:0006308	DNA catabolic process
BP	GO:0010039	response to iron ion
BP	GO:0010667	negative regulation of cardiac muscle cell apoptotic process
BP	GO:0014044	Schwann cell development
BP	GO:0034367	protein-containing complex remodeling
BP	GO:0035456	response to interferon-beta
BP	GO:0035883	enteroendocrine cell differentiation
BP	GO:0090022	regulation of neutrophil chemotaxis
BP	GO:1900745	positive regulation of p38MAPK cascade
BP	GO:1901889	negative regulation of cell junction assembly
BP	GO:1902235	regulation of endoplasmic reticulum stress-induced intrinsic
BP	GO:0046700	heterocycle catabolic process
BP	GO:1901988	negative regulation of cell cycle phase transition
BP	GO:0010976	positive regulation of neuron projection development
BP	GO:0198738	cell-cell signaling by wnt
BP	GO:0009199	ribonucleoside triphosphate metabolic process
BP	GO:0035304	regulation of protein dephosphorylation
BP	GO:0045638	negative regulation of myeloid cell differentiation
BP	GO:0046849	bone remodeling
BP	GO:0061097	regulation of protein tyrosine kinase activity
BP	GO:0001975	response to amphetamine
BP	GO:0009156	ribonucleoside monophosphate biosynthetic process
BP	GO:0009648	photoperiodism
BP	GO:0010259	multicellular organism aging
BP	GO:0030947	regulation of vascular endothelial growth factor receptor si[illegible]
BP	GO:0032633	interleukin-4 production
BP	GO:0032673	regulation of interleukin-4 production
BP	GO:0033198	response to ATP
BP	GO:0046320	regulation of fatty acid oxidation
BP	GO:0046685	response to arsenic-containing substance
BP	GO:0060260	regulation of transcription initiation from RNA polymerase I[illegible]
BP	GO:1902992	negative regulation of amyloid precursor protein catabolic p[illegible]
BP	GO:1904031	positive regulation of cyclin-dependent protein kinase activ[illegible]
BP	GO:0043087	regulation of GTPase activity
BP	GO:0044270	cellular nitrogen compound catabolic process
BP	GO:0001657	ureteric bud development
BP	GO:0002275	myeloid cell activation involved in immune response

BP	GO:0014033	neural crest cell differentiation
BP	GO:0045582	positive regulation of T cell differentiation
BP	GO:0060191	regulation of lipase activity
BP	GO:0072080	nephron tubule development
BP	GO:0042742	defense response to bacterium
BP	GO:0048525	negative regulation of viral process
BP	GO:0070509	calcium ion import
BP	GO:0072163	mesonephric epithelium development
BP	GO:0072164	mesonephric tubule development
BP	GO:1903351	cellular response to dopamine
BP	GO:0002446	neutrophil mediated immunity
BP	GO:0006356	regulation of transcription by RNA polymerase I
BP	GO:0007616	long-term memory
BP	GO:0009303	rRNA transcription
BP	GO:0009649	entrainment of circadian clock
BP	GO:0010922	positive regulation of phosphatase activity
BP	GO:0019320	hexose catabolic process
BP	GO:0032212	positive regulation of telomere maintenance via telomerase
BP	GO:0032743	positive regulation of interleukin-2 production
BP	GO:0035308	negative regulation of protein dephosphorylation
BP	GO:0048536	spleen development
BP	GO:0070232	regulation of T cell apoptotic process
BP	GO:0006937	regulation of muscle contraction
BP	GO:0060993	kidney morphogenesis
BP	GO:0106027	neuron projection organization
BP	GO:1903350	response to dopamine
BP	GO:0030178	negative regulation of Wnt signaling pathway
BP	GO:0000079	regulation of cyclin-dependent protein serine/threonine kina
BP	GO:0061326	renal tubule development
BP	GO:0001569	branching involved in blood vessel morphogenesis
BP	GO:0003298	physiological muscle hypertrophy
BP	GO:0003301	physiological cardiac muscle hypertrophy
BP	GO:0006471	protein ADP-ribosylation
BP	GO:0032350	regulation of hormone metabolic process
BP	GO:0033146	regulation of intracellular estrogen receptor signaling path
BP	GO:0035633	maintenance of blood-brain barrier
BP	GO:0043243	positive regulation of protein-containing complex disassembl
BP	GO:0061049	cell growth involved in cardiac muscle cell development
BP	GO:0072528	pyrimidine-containing compound biosynthetic process
BP	GO:2000403	positive regulation of lymphocyte migration
BP	GO:0001678	cellular glucose homeostasis
BP	GO:0007219	Notch signaling pathway
BP	GO:0042770	signal transduction in response to DNA damage
BP	GO:0016570	histone modification
BP	GO:0001823	mesonephros development
BP	GO:0003073	regulation of systemic arterial blood pressure
BP	GO:0010923	negative regulation of phosphatase activity
BP	GO:0030224	monocyte differentiation
BP	GO:0042755	eating behavior
BP	GO:0045746	negative regulation of Notch signaling pathway
BP	GO:0051354	negative regulation of oxidoreductase activity
BP	GO:0051385	response to mineralocorticoid
BP	GO:0060338	regulation of type I interferon-mediated signaling pathway
BP	GO:0070897	transcription preinitiation complex assembly

BP	GO:1902229	regulation of intrinsic apoptotic signaling pathway in respo[illegible]
BP	GO:0019439	aromatic compound catabolic process
BP	GO:0031589	cell-substrate adhesion
BP	GO:1904029	regulation of cyclin-dependent protein kinase activity
BP	GO:0001990	regulation of systemic arterial blood pressure by hormone
BP	GO:0003176	aortic valve development
BP	GO:0032570	response to progesterone
BP	GO:0042401	cellular biogenic amine biosynthetic process
BP	GO:0045730	respiratory burst
BP	GO:0048009	insulin-like growth factor receptor signaling pathway
BP	GO:1904358	positive regulation of telomere maintenance via telomere len[illegible]
BP	GO:1905332	positive regulation of morphogenesis of an epithelium
BP	GO:0032412	regulation of ion transmembrane transporter activity
BP	GO:0030324	lung development
BP	GO:0003300	cardiac muscle hypertrophy
BP	GO:0007631	feeding behavior
BP	GO:0060291	long-term synaptic potentiation
BP	GO:1901991	negative regulation of mitotic cell cycle phase transition
BP	GO:0002385	mucosal immune response
BP	GO:0008210	estrogen metabolic process
BP	GO:0009309	amine biosynthetic process
BP	GO:0051930	regulation of sensory perception of pain
BP	GO:0098926	postsynaptic signal transduction
BP	GO:2000279	negative regulation of DNA biosynthetic process
BP	GO:0006476	protein deacetylation
BP	GO:0030323	respiratory tube development
BP	GO:0014897	striated muscle hypertrophy
BP	GO:1990542	mitochondrial transmembrane transport
BP	GO:0002701	negative regulation of production of molecular mediator of i[illegible]
BP	GO:0016233	telomere capping
BP	GO:0016572	histone phosphorylation
BP	GO:0045923	positive regulation of fatty acid metabolic process
BP	GO:0046461	neutral lipid catabolic process
BP	GO:0046464	acylglycerol catabolic process
BP	GO:0051931	regulation of sensory perception
BP	GO:1902742	apoptotic process involved in development
BP	GO:0019395	fatty acid oxidation
BP	GO:0002714	positive regulation of B cell mediated immunity
BP	GO:0002891	positive regulation of immunoglobulin mediated immune respon[illegible]
BP	GO:0032892	positive regulation of organic acid transport
BP	GO:0045022	early endosome to late endosome transport
BP	GO:0046365	monosaccharide catabolic process
BP	GO:0055081	anion homeostasis
BP	GO:0007200	phospholipase C-activating G protein-coupled receptor signal[illegible]
BP	GO:0014896	muscle hypertrophy
BP	GO:0045621	positive regulation of lymphocyte differentiation
BP	GO:0002040	sprouting angiogenesis
BP	GO:0043433	negative regulation of DNA-binding transcription factor acti[illegible]
BP	GO:0120032	regulation of plasma membrane bounded cell projection assemb[illegible]
BP	GO:2001022	positive regulation of response to DNA damage stimulus
BP	GO:0033044	regulation of chromosome organization
BP	GO:0009167	purine ribonucleoside monophosphate metabolic process
BP	GO:0071470	cellular response to osmotic stress
BP	GO:1902622	regulation of neutrophil migration

BP	GO:1905314	semi-lunar valve development
BP	GO:1905521	regulation of macrophage migration
BP	GO:2000008	regulation of protein localization to cell surface
BP	GO:0010498	proteasomal protein catabolic process
BP	GO:0006470	protein dephosphorylation
BP	GO:0090263	positive regulation of canonical Wnt signaling pathway
BP	GO:0060491	regulation of cell projection assembly
BP	GO:0009060	aerobic respiration
BP	GO:0021761	limbic system development
BP	GO:0036503	ERAD pathway
BP	GO:0071868	cellular response to monoamine stimulus
BP	GO:0071870	cellular response to catecholamine stimulus
BP	GO:0009566	fertilization
BP	GO:0009755	hormone-mediated signaling pathway
BP	GO:0009124	nucleoside monophosphate biosynthetic process
BP	GO:0010677	negative regulation of cellular carbohydrate metabolic proce[illegible]
BP	GO:0014912	negative regulation of smooth muscle cell migration
BP	GO:0032689	negative regulation of interferon-gamma production
BP	GO:0032965	regulation of collagen biosynthetic process
BP	GO:0033003	regulation of mast cell activation
BP	GO:0045687	positive regulation of glial cell differentiation
BP	GO:0055090	acylglycerol homeostasis
BP	GO:0060999	positive regulation of dendritic spine development
BP	GO:0070328	triglyceride homeostasis
BP	GO:2000404	regulation of T cell migration
BP	GO:0034440	lipid oxidation
BP	GO:0048562	embryonic organ morphogenesis
BP	GO:0032414	positive regulation of ion transmembrane transporter activit[illegible]
BP	GO:0072009	nephron epithelium development
BP	GO:0140014	mitotic nuclear division
BP	GO:0002861	regulation of inflammatory response to antigenic stimulus
BP	GO:0034142	toll-like receptor 4 signaling pathway
BP	GO:0045776	negative regulation of blood pressure
BP	GO:0098927	vesicle-mediated transport between endosomal compartments
BP	GO:0120178	steroid hormone biosynthetic process
BP	GO:1903793	positive regulation of anion transport
BP	GO:0061387	regulation of extent of cell growth
BP	GO:0050777	negative regulation of immune response
BP	GO:0071867	response to monoamine
BP	GO:0071869	response to catecholamine
BP	GO:0090288	negative regulation of cellular response to growth factor st[illegible]
BP	GO:0008542	visual learning
BP	GO:0009126	purine nucleoside monophosphate metabolic process
BP	GO:0048066	developmental pigmentation
BP	GO:0060711	labyrinthine layer development
BP	GO:0062208	positive regulation of pattern recognition receptor signalin[illegible]
BP	GO:0097178	ruffle assembly
BP	GO:0045664	regulation of neuron differentiation
BP	GO:0001676	long-chain fatty acid metabolic process
BP	GO:0009141	nucleoside triphosphate metabolic process
BP	GO:0010633	negative regulation of epithelial cell migration
BP	GO:0035601	protein deacylation
BP	GO:0048167	regulation of synaptic plasticity
BP	GO:0002920	regulation of humoral immune response

BP	GO:0030850	prostate gland development
BP	GO:0031670	cellular response to nutrient
BP	GO:0035272	exocrine system development
BP	GO:0035305	negative regulation of dephosphorylation
BP	GO:0035987	endodermal cell differentiation
BP	GO:0042551	neuron maturation
BP	GO:0045933	positive regulation of muscle contraction
BP	GO:0051180	vitamin transport
BP	GO:0010948	negative regulation of cell cycle process
BP	GO:0000077	DNA damage checkpoint signaling
BP	GO:0046916	cellular transition metal ion homeostasis
BP	GO:0001974	blood vessel remodeling
BP	GO:0006383	transcription by RNA polymerase III
BP	GO:0042398	cellular modified amino acid biosynthetic process
BP	GO:0043300	regulation of leukocyte degranulation
BP	GO:1900087	positive regulation of G1/S transition of mitotic cell cycle
BP	GO:1900271	regulation of long-term synaptic potentiation
BP	GO:0006473	protein acetylation
BP	GO:0031647	regulation of protein stability
BP	GO:0006720	isoprenoid metabolic process
BP	GO:0098732	macromolecule deacylation
BP	GO:0003044	regulation of systemic arterial blood pressure mediated by a
BP	GO:0010712	regulation of collagen metabolic process
BP	GO:0010761	fibroblast migration
BP	GO:0014911	positive regulation of smooth muscle cell migration
BP	GO:0031641	regulation of myelination
BP	GO:0032369	negative regulation of lipid transport
BP	GO:0045601	regulation of endothelial cell differentiation
BP	GO:0045747	positive regulation of Notch signaling pathway
BP	GO:0045911	positive regulation of DNA recombination
BP	GO:0048483	autonomic nervous system development
BP	GO:0048512	circadian behavior
BP	GO:1900744	regulation of p38MAPK cascade
BP	GO:1903170	negative regulation of calcium ion transmembrane transport
BP	GO:1901888	regulation of cell junction assembly
BP	GO:0009308	amine metabolic process
BP	GO:0030203	glycosaminoglycan metabolic process
BP	GO:0015849	organic acid transport
BP	GO:0043303	mast cell degranulation
BP	GO:0048146	positive regulation of fibroblast proliferation
BP	GO:0120009	intermembrane lipid transfer
BP	GO:0043161	proteasome-mediated ubiquitin-dependent protein catabolic pr
BP	GO:0032411	positive regulation of transporter activity
BP	GO:0048675	axon extension
BP	GO:0002204	somatic recombination of immunoglobulin genes involved in im
BP	GO:0002208	somatic diversification of immunoglobulins involved in immun
BP	GO:0002279	mast cell activation involved in immune response
BP	GO:0002762	negative regulation of myeloid leukocyte differentiation
BP	GO:0007622	rhythmic behavior
BP	GO:0042304	regulation of fatty acid biosynthetic process
BP	GO:0045190	isotype switching
BP	GO:0052372	modulation by symbiont of entry into host
BP	GO:0060986	endocrine hormone secretion
BP	GO:1902905	positive regulation of supramolecular fiber organization

BP	GO:0045931	positive regulation of mitotic cell cycle
BP	GO:0001961	positive regulation of cytokine-mediated signaling pathway
BP	GO:0002448	mast cell mediated immunity
BP	GO:0002639	positive regulation of immunoglobulin production
BP	GO:0007632	visual behavior
BP	GO:0008089	anterograde axonal transport
BP	GO:0016311	dephosphorylation
BP	GO:0031570	DNA integrity checkpoint signaling
BP	GO:0050852	T cell receptor signaling pathway
BP	GO:0009749	response to glucose
BP	GO:0032964	collagen biosynthetic process
BP	GO:0043124	negative regulation of I-kappaB kinase/NF-kappaB signaling
BP	GO:0045104	intermediate filament cytoskeleton organization
BP	GO:0048013	ephrin receptor signaling pathway
BP	GO:0051339	regulation of lyase activity
BP	GO:0060043	regulation of cardiac muscle cell proliferation
BP	GO:0006605	protein targeting
BP	GO:0044242	cellular lipid catabolic process
BP	GO:0002768	immune response-regulating cell surface receptor signaling pa
BP	GO:0044772	mitotic cell cycle phase transition
BP	GO:0002218	activation of innate immune response
BP	GO:0031103	axon regeneration
BP	GO:0035176	social behavior
BP	GO:0045103	intermediate filament-based process
BP	GO:1903426	regulation of reactive oxygen species biosynthetic process
BP	GO:0031929	TOR signaling
BP	GO:0002377	immunoglobulin production
BP	GO:0051224	negative regulation of protein transport
BP	GO:0016925	protein sumoylation
BP	GO:0032210	regulation of telomere maintenance via telomerase
BP	GO:1903202	negative regulation of oxidative stress-induced cell death
BP	GO:1990090	cellular response to nerve growth factor stimulus
BP	GO:0017015	regulation of transforming growth factor beta receptor signal
BP	GO:0006022	aminoglycan metabolic process
BP	GO:0001706	endoderm formation
BP	GO:0002823	negative regulation of adaptive immune response based on soma
BP	GO:0006584	catecholamine metabolic process
BP	GO:0006968	cellular defense response
BP	GO:0009712	catechol-containing compound metabolic process
BP	GO:0051703	biological process involved in intraspecies interaction betwe
BP	GO:0070228	regulation of lymphocyte apoptotic process
BP	GO:0006352	DNA-templated transcription, initiation
BP	GO:0006360	transcription by RNA polymerase I
BP	GO:0061098	positive regulation of protein tyrosine kinase activity
BP	GO:0006612	protein targeting to membrane
BP	GO:0019827	stem cell population maintenance
BP	GO:0045089	positive regulation of innate immune response
BP	GO:0090101	negative regulation of transmembrane receptor protein serine/
BP	GO:1904950	negative regulation of establishment of protein localization
BP	GO:0001912	positive regulation of leukocyte mediated cytotoxicity
BP	GO:0002090	regulation of receptor internalization
BP	GO:0002886	regulation of myeloid leukocyte mediated immunity
BP	GO:0048255	mRNA stabilization
BP	GO:0055078	sodium ion homeostasis

BP	GO:0098781	ncRNA transcription
BP	GO:1990089	response to nerve growth factor
BP	GO:0008277	regulation of G protein-coupled receptor signaling pathway
BP	GO:0032147	activation of protein kinase activity
BP	GO:0098727	maintenance of cell number
BP	GO:0006611	protein export from nucleus
BP	GO:0016447	somatic recombination of immunoglobulin gene segments
BP	GO:0071695	anatomical structure maturation
BP	GO:1903305	regulation of regulated secretory pathway
BP	GO:0001954	positive regulation of cell-matrix adhesion
BP	GO:0009161	ribonucleoside monophosphate metabolic process
BP	GO:0010559	regulation of glycoprotein biosynthetic process
BP	GO:1902808	positive regulation of cell cycle G1/S phase transition
BP	GO:2000736	regulation of stem cell differentiation
BP	GO:0050714	positive regulation of protein secretion
BP	GO:0002820	negative regulation of adaptive immune response
BP	GO:0019369	arachidonic acid metabolic process
BP	GO:0035306	positive regulation of dephosphorylation
BP	GO:2001258	negative regulation of cation channel activity
BP	GO:0007265	Ras protein signal transduction
BP	GO:0032273	positive regulation of protein polymerization
BP	GO:0055076	transition metal ion homeostasis
BP	GO:0006790	sulfur compound metabolic process
BP	GO:0002712	regulation of B cell mediated immunity
BP	GO:0002889	regulation of immunoglobulin mediated immune response
BP	GO:0009620	response to fungus
BP	GO:0031102	neuron projection regeneration
BP	GO:0051851	modulation by host of symbiont process
BP	GO:0060038	cardiac muscle cell proliferation
BP	GO:0030177	positive regulation of Wnt signaling pathway
BP	GO:0048738	cardiac muscle tissue development
BP	GO:0043954	cellular component maintenance
BP	GO:0060998	regulation of dendritic spine development
BP	GO:0072006	nephron development
BP	GO:0009116	nucleoside metabolic process
BP	GO:0045576	mast cell activation
BP	GO:0046148	pigment biosynthetic process
BP	GO:0060675	ureteric bud morphogenesis
BP	GO:0030010	establishment of cell polarity
CC	GO:0045121	membrane raft
CC	GO:0098857	membrane microdomain
CC	GO:0009897	external side of plasma membrane
CC	GO:0005901	caveola
CC	GO:0005769	early endosome
CC	GO:0044853	plasma membrane raft
CC	GO:0031968	organelle outer membrane
CC	GO:0019867	outer membrane
CC	GO:0005788	endoplasmic reticulum lumen
CC	GO:0031983	vesicle lumen
CC	GO:0005741	mitochondrial outer membrane
CC	GO:0046930	pore complex
CC	GO:0034774	secretory granule lumen
CC	GO:0060205	cytoplasmic vesicle lumen
CC	GO:0031143	pseudopodium

CC	GO:0071682	endocytic vesicle lumen
CC	GO:0030139	endocytic vesicle
CC	GO:1904813	ficolin-1-rich granule lumen
CC	GO:0005759	mitochondrial matrix
CC	GO:0009295	nucleoid
CC	GO:0042645	mitochondrial nucleoid
CC	GO:0005925	focal adhesion
CC	GO:0034362	low-density lipoprotein particle
CC	GO:0042627	chylomicron
CC	GO:0043209	myelin sheath
CC	GO:0030055	cell-substrate junction
CC	GO:0062023	collagen-containing extracellular matrix
CC	GO:0016605	PML body
CC	GO:0098978	glutamatergic synapse
CC	GO:0042470	melanosome
CC	GO:0048770	pigment granule
CC	GO:0101002	ficolin-1-rich granule
CC	GO:0030666	endocytic vesicle membrane
CC	GO:0005635	nuclear envelope
CC	GO:0034361	very-low-density lipoprotein particle
CC	GO:0034385	triglyceride-rich plasma lipoprotein particle
CC	GO:0031093	platelet alpha granule lumen
CC	GO:0030669	clathrin-coated endocytic vesicle membrane
CC	GO:0031233	intrinsic component of external side of plasma membrane
CC	GO:0005777	peroxisome
CC	GO:0042579	microbody
CC	GO:0005667	transcription regulator complex
CC	GO:0072562	blood microparticle
CC	GO:0034364	high-density lipoprotein particle
CC	GO:0001891	phagocytic cup
MF	GO:0005126	cytokine receptor binding
MF	GO:0019902	phosphatase binding
MF	GO:0061629	RNA polymerase II-specific DNA-binding transcription factor [illegible]
MF	GO:0005125	cytokine activity
MF	GO:0140297	DNA-binding transcription factor binding
MF	GO:0019903	protein phosphatase binding
MF	GO:0005496	steroid binding
MF	GO:0051721	protein phosphatase 2A binding
MF	GO:0048018	receptor ligand activity
MF	GO:0030546	signaling receptor activator activity
MF	GO:0140296	general transcription initiation factor binding
MF	GO:0044389	ubiquitin-like protein ligase binding
MF	GO:0004879	nuclear receptor activity
MF	GO:0098531	ligand-activated transcription factor activity
MF	GO:0004707	MAP kinase activity
MF	GO:0002020	protease binding
MF	GO:0031625	ubiquitin protein ligase binding
MF	GO:0005178	integrin binding
MF	GO:0004497	monooxygenase activity
MF	GO:0042379	chemokine receptor binding
MF	GO:0001091	RNA polymerase II general transcription initiation factor bi[illegible]
MF	GO:0001221	transcription coregulator binding
MF	GO:0016705	oxidoreductase activity, acting on paired donors, with incor[illegible]
MF	GO:0004252	serine-type endopeptidase activity

MF	GO:0032813	tumor necrosis factor receptor superfamily binding
MF	GO:0016597	amino acid binding
MF	GO:0050661	NADP binding
MF	GO:0008236	serine-type peptidase activity
MF	GO:0020037	heme binding
MF	GO:0017171	serine hydrolase activity
MF	GO:0046906	tetrapyrrole binding
MF	GO:0005164	tumor necrosis factor receptor binding
MF	GO:0051400	BH domain binding
MF	GO:0001223	transcription coactivator binding
MF	GO:0004175	endopeptidase activity
MF	GO:0009055	electron transfer activity
MF	GO:0004712	protein serine/threonine/tyrosine kinase activity
MF	GO:0046982	protein heterodimerization activity
MF	GO:0043177	organic acid binding
MF	GO:0004708	MAP kinase kinase activity
MF	GO:0045236	CXCR chemokine receptor binding
MF	GO:0016209	antioxidant activity
MF	GO:0019838	growth factor binding
MF	GO:0008009	chemokine activity
MF	GO:0016922	nuclear receptor binding
MF	GO:0140677	molecular function activator activity
MF	GO:0001228	DNA-binding transcription activator activity, RNA polymerase
MF	GO:0001216	DNA-binding transcription activator activity
MF	GO:0035035	histone acetyltransferase binding
MF	GO:0051087	chaperone binding
MF	GO:0005507	copper ion binding
MF	GO:0004674	protein serine/threonine kinase activity
MF	GO:0008047	enzyme activator activity
MF	GO:0033218	amide binding
MF	GO:0031406	carboxylic acid binding
MF	GO:0051059	NF-kappaB binding
MF	GO:0001098	basal transcription machinery binding
MF	GO:0001099	basal RNA polymerase II transcription machinery binding
MF	GO:0042826	histone deacetylase binding
MF	GO:0031072	heat shock protein binding
MF	GO:0106310	protein serine kinase activity
MF	GO:0070851	growth factor receptor binding
MF	GO:0001094	TFIID-class transcription factor complex binding
MF	GO:0097199	cysteine-type endopeptidase activity involved in apoptotic s
MF	GO:0050660	flavin adenine dinucleotide binding
MF	GO:0016712	oxidoreductase activity, acting on paired donors, with incor
MF	GO:0030331	estrogen receptor binding
MF	GO:0001664	G protein-coupled receptor binding
MF	GO:0051219	phosphoprotein binding
MF	GO:0070513	death domain binding
MF	GO:0051117	ATPase binding
MF	GO:0001046	core promoter sequence-specific DNA binding
MF	GO:0016709	oxidoreductase activity, acting on paired donors, with incor
MF	GO:0051879	Hsp90 protein binding
MF	GO:0048156	tau protein binding
MF	GO:0048020	CCR chemokine receptor binding
MF	GO:0071837	HMG box domain binding
MF	GO:0008201	heparin binding

MF	GO:0015485	cholesterol binding
MF	GO:0050998	nitric-oxide synthase binding
MF	GO:0097153	cysteine-type endopeptidase activity involved in apoptotic p[illegible]
MF	GO:1990782	protein tyrosine kinase binding
MF	GO:0045296	cadherin binding
MF	GO:0005159	insulin-like growth factor receptor binding
MF	GO:0010181	FMN binding
MF	GO:0005149	interleukin-1 receptor binding
MF	GO:1901681	sulfur compound binding
MF	GO:0016627	oxidoreductase activity, acting on the CH-CH group of donors
MF	GO:0032934	sterol binding
MF	GO:1990841	promoter-specific chromatin binding
MF	GO:0000979	RNA polymerase II core promoter sequence-specific DNA bindin[illegible]
MF	GO:0004714	transmembrane receptor protein tyrosine kinase activity
MF	GO:0005543	phospholipid binding
MF	GO:0016810	hydrolase activity, acting on carbon-nitrogen (but not pepti[illegible]
MF	GO:0005123	death receptor binding
MF	GO:0070182	DNA polymerase binding
MF	GO:0120020	cholesterol transfer activity
MF	GO:0044325	transmembrane transporter binding
MF	GO:0005158	insulin receptor binding
MF	GO:0017025	TBP-class protein binding
MF	GO:0050750	low-density lipoprotein particle receptor binding
MF	GO:0070412	R-SMAD binding
MF	GO:0120015	sterol transfer activity
MF	GO:0004713	protein tyrosine kinase activity
MF	GO:0003707	steroid hormone receptor activity
MF	GO:0070330	aromatase activity
MF	GO:0019199	transmembrane receptor protein kinase activity
MF	GO:0004709	MAP kinase kinase kinase activity
MF	GO:0030971	receptor tyrosine kinase binding
MF	GO:0046332	SMAD binding
MF	GO:0019842	vitamin binding
MF	GO:0005539	glycosaminoglycan binding
MF	GO:0070325	lipoprotein particle receptor binding
MF	GO:0042277	peptide binding
MF	GO:0043325	phosphatidylinositol-3,4-bisphosphate binding
MF	GO:0001540	amyloid-beta binding
MF	GO:0008013	beta-catenin binding
MF	GO:0042562	hormone binding
MF	GO:0090482	vitamin transmembrane transporter activity
MF	GO:0001222	transcription corepressor binding
MF	GO:0008083	growth factor activity
MF	GO:0043178	alcohol binding
MF	GO:0001530	lipopolysaccharide binding
MF	GO:0016248	channel inhibitor activity
MF	GO:0015248	sterol transporter activity
MF	GO:0042805	actinin binding
MF	GO:0097718	disordered domain specific binding
MF	GO:0008395	steroid hydroxylase activity
MF	GO:0019825	oxygen binding

GeneRatio	BgRatio	pvalue	p.adjust	qvalue	geneID	Count
33/108	343/18723	1.81E-31	6.79E-28	2.86E-28	TNF/IL1B/IL6/	33
33/108	363/18723	1.19E-30	2.23E-27	9.38E-28	TNF/IL1B/IL6/	33
31/108	446/18723	3.55E-25	4.44E-22	1.87E-22	IL6/PTGS2/TLI	31
31/108	456/18723	6.94E-25	6.52E-22	2.74E-22	CXCL10/THBD/I	31
25/108	246/18723	2.22E-24	1.66E-21	7.01E-22	TNF/IL1B/IL6/	25
24/108	222/18723	4.55E-24	2.84E-21	1.20E-21	IL6/EGFR/REL/	24
27/108	337/18723	1.55E-23	8.30E-21	3.49E-21	IL6/PTGS2/TLI	27
30/108	474/18723	3.64E-23	1.71E-20	7.19E-21	GPT/CXCL10/PT	30
27/108	373/18723	2.29E-22	9.54E-20	4.02E-20	PTGS2/HSPA5/I	27
22/108	209/18723	7.70E-22	2.89E-19	1.22E-19	TNF/IL1B/IL6/	22
26/108	356/18723	1.22E-21	4.16E-19	1.75E-19	TNF/IL1B/IL4/	26
24/108	288/18723	2.38E-21	7.46E-19	3.14E-19	IL6/TLR4/EGFI	24
22/108	221/18723	2.66E-21	7.68E-19	3.23E-19	TNF/IL1B/IL6/	22
25/108	331/18723	3.57E-21	8.93E-19	3.76E-19	IL1B/PTGS2/HS	25
25/108	331/18723	3.57E-21	8.93E-19	3.76E-19	IL1B/PTGS2/HS	25
16/108	81/18723	1.17E-20	2.74E-18	1.15E-18	TNF/IFNG/IL1I	16
16/108	82/18723	1.44E-20	3.03E-18	1.27E-18	TNF/IFNG/IL1I	16
19/108	149/18723	1.45E-20	3.03E-18	1.27E-18	PTGS2/EGFR/RI	19
22/108	253/18723	5.20E-20	1.03E-17	4.33E-18	TNF/CXCL8/PT(	22
26/108	422/18723	8.86E-20	1.66E-17	7.00E-18	TNF/IL6/RAF1/	26
24/108	339/18723	1.11E-19	1.99E-17	8.37E-18	PTGS2/RELA/B/	24
26/108	436/18723	2.00E-19	3.30E-17	1.39E-17	TNF/IL6/RAF1/	26
15/108	76/18723	2.02E-19	3.30E-17	1.39E-17	TNF/IFNG/IL1I	15
18/108	151/18723	5.54E-19	8.67E-17	3.65E-17	TNF/IL1B/IL4/	18
26/108	462/18723	8.42E-19	1.26E-16	5.32E-17	IL1B/PTGS2/HS	26
20/108	219/18723	1.14E-18	1.64E-16	6.91E-17	TNF/IFNG/IL1I	20
20/108	224/18723	1.78E-18	2.47E-16	1.04E-16	TNF/IL1B/IL4/	20
12/108	40/18723	3.53E-18	4.74E-16	2.00E-16	TNF/IFNG/IL1I	12
12/108	41/18723	4.97E-18	6.44E-16	2.71E-16	TNF/IFNG/IL1I	12
23/108	359/18723	6.51E-18	8.15E-16	3.43E-16	IL1B/GPT/TLR4	23
22/108	320/18723	8.29E-18	1.00E-15	4.23E-16	IL1B/PTGS2/HS	22
15/108	97/18723	9.97E-18	1.17E-15	4.93E-16	TNF/IL1B/IL4/	15
19/108	216/18723	1.82E-17	2.07E-15	8.71E-16	IL1B/RAF1/CX(	19
13/108	62/18723	2.44E-17	2.70E-15	1.14E-15	TNF/IFNG/IL1I	13
13/108	64/18723	3.83E-17	4.10E-15	1.73E-15	TNF/IFNG/IL1I	13
19/108	229/18723	5.45E-17	5.64E-15	2.37E-15	TNF/CXCL8/CCI	19
21/108	307/18723	5.56E-17	5.64E-15	2.37E-15	RAF1/PTGS2/B(	21
13/108	68/18723	8.96E-17	8.85E-15	3.73E-15	EGFR/CAT/NCF1	13
22/108	361/18723	1.07E-16	1.03E-14	4.32E-15	TNF/IFNG/HSP/	22
19/108	239/18723	1.21E-16	1.14E-14	4.79E-15	TNF/TLR4/EGFI	19
21/108	320/18723	1.29E-16	1.18E-14	4.97E-15	PTGS2/EGFR/RI	21
21/108	322/18723	1.46E-16	1.30E-14	5.49E-15	RAF1/PTGS2/B(	21
23/108	414/18723	1.49E-16	1.30E-14	5.49E-15	IL1B/GPT/PTGS	23
19/108	248/18723	2.42E-16	2.06E-14	8.68E-15	TNF/IFNG/IL6/	19
11/108	40/18723	2.81E-16	2.34E-14	9.86E-15	EGFR/NCF1/AKT	11
21/108	334/18723	3.06E-16	2.50E-14	1.05E-14	TNF/IFNG/IL1I	21
21/108	336/18723	3.45E-16	2.76E-14	1.16E-14	TNF/IFNG/IL1I	21
16/108	155/18723	5.86E-16	4.58E-14	1.93E-14	IL6/EGFR/REL/	16
21/108	347/18723	6.60E-16	5.05E-14	2.13E-14	RAF1/PTGS2/B(	21
23/108	448/18723	8.27E-16	6.21E-14	2.62E-14	TNF/IFNG/IL1I	23
14/108	104/18723	9.80E-16	7.21E-14	3.04E-14	TNF/IL6/PTGS2	14
20/108	319/18723	1.78E-15	1.28E-13	5.40E-14	TNF/IFNG/CCL2	20
21/108	371/18723	2.51E-15	1.77E-13	7.45E-14	TNF/IFNG/IL1I	21

23/108	472/18723	2.54E-15	1.77E-13	7.45E-14	TNF/IFNG/IL1B	23
22/108	427/18723	3.54E-15	2.41E-13	1.02E-13	TNF/IFNG/IL1B	22
16/108	174/18723	3.73E-15	2.50E-13	1.05E-13	CXCL10/PTGS2/	16
19/108	290/18723	4.32E-15	2.85E-13	1.20E-13	IL1B/GPT/RELA	19
21/108	386/18723	5.52E-15	3.57E-13	1.50E-13	TNF/IFNG/IL1B	21
16/108	180/18723	6.40E-15	4.07E-13	1.72E-13	TNF/IFNG/IL6/	16
15/108	148/18723	6.67E-15	4.17E-13	1.76E-13	CXCL10/THBD/H	15
17/108	220/18723	8.93E-15	5.49E-13	2.31E-13	IL1B/THBD/PTG	17
16/108	184/18723	9.07E-15	5.49E-13	2.31E-13	TNF/IFNG/IL6/	16
17/108	226/18723	1.40E-14	8.32E-13	3.51E-13	PTGS2/HSPA5/B	17
16/108	194/18723	2.09E-14	1.23E-12	5.17E-13	EGFR/RELA/SPP	16
22/108	467/18723	2.22E-14	1.28E-12	5.41E-13	TNF/IFNG/IL1B	22
16/108	197/18723	2.66E-14	1.51E-12	6.38E-13	PTGS2/HSPA5/B	16
17/108	239/18723	3.52E-14	1.97E-12	8.31E-13	TNF/IFNG/IL1B	17
14/108	134/18723	3.66E-14	2.02E-12	8.50E-13	TNF/THBD/PLAT	14
15/108	167/18723	4.07E-14	2.21E-12	9.32E-13	TNF/THBD/PLAT	15
20/108	379/18723	4.68E-14	2.51E-12	1.06E-12	IL6/THBD/TLR4	20
20/108	381/18723	5.17E-14	2.72E-12	1.15E-12	TNF/IFNG/IL4/	20
22/108	487/18723	5.22E-14	2.72E-12	1.15E-12	IFNG/IL1B/IL6	22
19/108	339/18723	7.33E-14	3.77E-12	1.59E-12	TNF/IL6/PTGS2	19
14/108	141/18723	7.48E-14	3.79E-12	1.60E-12	PTGS2/EGFR/CA	14
13/108	112/18723	7.88E-14	3.95E-12	1.66E-12	TNF/IL1B/IL6/	13
11/108	66/18723	1.15E-13	5.69E-12	2.40E-12	THBD/PLAT/NOS	11
16/108	217/18723	1.21E-13	5.89E-12	2.48E-12	IL6/THBD/TLR4	16
17/108	262/18723	1.59E-13	7.65E-12	3.22E-12	PTGS2/BAK1/BC	17
11/108	68/18723	1.63E-13	7.74E-12	3.26E-12	THBD/PLAT/NOS	11
16/108	222/18723	1.72E-13	7.98E-12	3.36E-12	IL6/THBD/TLR4	16
16/108	222/18723	1.72E-13	7.98E-12	3.36E-12	IL6/THBD/TLR4	16
15/108	186/18723	2.01E-13	9.18E-12	3.87E-12	PTGS2/HSPA5/B	15
13/108	121/18723	2.18E-13	9.87E-12	4.16E-12	TNF/IL6/IL4/K	13
11/108	71/18723	2.68E-13	1.20E-11	5.05E-12	THBD/PLAT/NOS	11
12/108	96/18723	3.03E-13	1.34E-11	5.63E-12	TNF/IL6/IL4/K	12
19/108	369/18723	3.34E-13	1.45E-11	6.09E-12	TNF/IL6/CXCL8	19
14/108	157/18723	3.35E-13	1.45E-11	6.09E-12	TNF/TLR4/EGFR	14
15/108	193/18723	3.45E-13	1.47E-11	6.20E-12	IL1B/THBD/PTG	15
20/108	426/18723	4.15E-13	1.75E-11	7.37E-12	IFNG/IL1B/IL6	20
20/108	427/18723	4.33E-13	1.81E-11	7.61E-12	TNF/IFNG/IL1B	20
17/108	284/18723	5.90E-13	2.43E-11	1.02E-11	TNF/IFNG/IL1B	17
20/108	437/18723	6.64E-13	2.68E-11	1.13E-11	TNF/IFNG/IL1B	20
20/108	437/18723	6.64E-13	2.68E-11	1.13E-11	TNF/IL6/KDR/C	20
17/108	288/18723	7.39E-13	2.95E-11	1.24E-11	TNF/PTGS2/BAK	17
20/108	440/18723	7.54E-13	2.97E-11	1.25E-11	TNF/IL1B/IL6/	20
10/108	56/18723	7.61E-13	2.97E-11	1.25E-11	CXCL10/HSPA5/	10
16/108	245/18723	7.90E-13	3.06E-11	1.29E-11	IL1B/IL6/IL4/	16
18/108	338/18723	8.44E-13	3.23E-11	1.36E-11	TNF/IFNG/IL6/	18
15/108	210/18723	1.18E-12	4.49E-11	1.89E-11	TNF/IL6/CXCL8	15
11/108	81/18723	1.21E-12	4.54E-11	1.91E-11	IL1B/PTGS2/TL	11
10/108	59/18723	1.32E-12	4.92E-11	2.07E-11	TNF/IFNG/IL1B	10
16/108	260/18723	1.96E-12	7.22E-11	3.04E-11	TNF/IL1B/IL6/	16
10/108	62/18723	2.23E-12	8.14E-11	3.43E-11	TNF/IL1B/IL6/	10
13/108	146/18723	2.51E-12	9.04E-11	3.81E-11	IL6/RELA/BAK1	13
12/108	116/18723	3.03E-12	1.08E-10	4.56E-11	TNF/RELA/MMP2	12
17/108	315/18723	3.12E-12	1.10E-10	4.65E-11	TNF/IFNG/IL6/	17
17/108	318/18723	3.63E-12	1.26E-10	5.31E-11	IL1B/IL6/IL4/	17

10/108	65/18723	3.66E-12	1.26E-10	5.31E-11	TNF/IL1B/IL4/	10
10/108	65/18723	3.66E-12	1.26E-10	5.31E-11	TNF/IL1B/IL4/	10
20/108	480/18723	3.71E-12	1.27E-10	5.33E-11	TNF/IL1B/IL6/	20
19/108	424/18723	3.86E-12	1.31E-10	5.50E-11	PTGS2/HSPA5/V	19
19/108	427/18723	4.37E-12	1.47E-10	6.17E-11	PTGS2/HSPA5/V	19
18/108	374/18723	4.62E-12	1.53E-10	6.44E-11	IFNG/IL1B/IL6	18
9/108	46/18723	4.64E-12	1.53E-10	6.44E-11	TNF/RELA/BCL2	9
17/108	327/18723	5.66E-12	1.85E-10	7.78E-11	TNF/IL1B/IL6/	17
20/108	492/18723	5.81E-12	1.88E-10	7.92E-11	TNF/IFNG/IL1B	20
17/108	329/18723	6.24E-12	2.00E-10	8.43E-11	IFNG/IL1B/IL6	17
11/108	94/18723	6.48E-12	2.06E-10	8.68E-11	TNF/THBD/PLAT	11
17/108	342/18723	1.15E-11	3.62E-10	1.53E-10	TNF/IL1B/IL6/	17
15/108	246/18723	1.16E-11	3.62E-10	1.53E-10	TNF/HSPA5/CCL	15
12/108	131/18723	1.30E-11	4.04E-10	1.70E-10	THBD/PTGS2/HS	12
13/108	167/18723	1.40E-11	4.30E-10	1.81E-10	TNF/IL6/PTGS2	13
17/108	348/18723	1.52E-11	4.63E-10	1.95E-10	TNF/IL1B/IL6/	17
16/108	301/18723	1.80E-11	5.45E-10	2.30E-10	TNF/IFNG/IL1B	16
12/108	135/18723	1.86E-11	5.59E-10	2.35E-10	TNF/RELA/MMP2	12
10/108	78/18723	2.42E-11	7.15E-10	3.01E-10	TNF/THBD/PLAT	10
10/108	78/18723	2.42E-11	7.15E-10	3.01E-10	BAK1/BCL2/STA	10
11/108	107/18723	2.73E-11	7.95E-10	3.35E-10	BAK1/BCL2/TP5	11
11/108	107/18723	2.73E-11	7.95E-10	3.35E-10	TNF/IFNG/IL1B	11
12/108	141/18723	3.12E-11	8.86E-10	3.73E-10	IL4/KDR/TP53/	12
12/108	141/18723	3.12E-11	8.86E-10	3.73E-10	PTGS2/EGFR/AK	12
12/108	141/18723	3.12E-11	8.86E-10	3.73E-10	PTGS2/EGFR/AK	12
12/108	142/18723	3.39E-11	9.56E-10	4.03E-10	PTGS2/EGFR/AK	12
17/108	367/18723	3.50E-11	9.81E-10	4.13E-10	TNF/IFNG/IL6/	17
12/108	143/18723	3.68E-11	1.02E-09	4.31E-10	TNF/IFNG/IL1B	12
11/108	110/18723	3.71E-11	1.02E-09	4.31E-10	CXCL10/PTGS2/	11
16/108	317/18723	3.91E-11	1.07E-09	4.51E-10	IFNG/IL4/KDR/	16
10/108	82/18723	4.04E-11	1.10E-09	4.62E-10	TNF/RAF1/BCL2	10
16/108	319/18723	4.29E-11	1.16E-09	4.88E-10	TNF/IFNG/IL4/	16
14/108	225/18723	4.50E-11	1.20E-09	5.05E-10	TNF/IFNG/IL6/	14
14/108	225/18723	4.50E-11	1.20E-09	5.05E-10	IL1B/IL6/IL4/	14
17/108	375/18723	4.91E-11	1.30E-09	5.47E-10	TNF/IFNG/IL6/	17
9/108	59/18723	4.98E-11	1.31E-09	5.50E-10	TNF/IL4/KDR/C	9
14/108	227/18723	5.07E-11	1.32E-09	5.56E-10	IL1B/IL6/IL4/	14
12/108	148/18723	5.52E-11	1.42E-09	5.98E-10	THBD/PTGS2/HS	12
12/108	148/18723	5.52E-11	1.42E-09	5.98E-10	TNF/IL6/PTGS2	12
17/108	378/18723	5.56E-11	1.42E-09	5.98E-10	TNF/IFNG/IL6/	17
9/108	60/18723	5.83E-11	1.48E-09	6.23E-10	TNF/IL1B/CCL2	9
17/108	381/18723	6.29E-11	1.58E-09	6.67E-10	TNF/KDR/CCL2/	17
12/108	150/18723	6.46E-11	1.62E-09	6.81E-10	IL1B/IL6/IL4/	12
9/108	62/18723	7.92E-11	1.97E-09	8.30E-10	PTGS2/VDR/SPP	9
9/108	63/18723	9.20E-11	2.27E-09	9.57E-10	BAK1/BCL2/STA	9
6/108	14/18723	9.26E-11	2.27E-09	9.57E-10	EGFR/AKT1/CEF	6
10/108	90/18723	1.04E-10	2.53E-09	1.07E-09	PTGS2/BAK1/TF	10
13/108	196/18723	1.05E-10	2.54E-09	1.07E-09	TNF/IL6/IL4/C	13
15/108	288/18723	1.08E-10	2.60E-09	1.09E-09	IL1B/IL6/IL4/	15
9/108	65/18723	1.23E-10	2.94E-09	1.24E-09	TNF/IL4/KDR/C	9
15/108	291/18723	1.25E-10	2.96E-09	1.25E-09	IL1B/IL6/IL4/	15
8/108	44/18723	1.41E-10	3.32E-09	1.40E-09	TNF/IFNG/IL1B	8
8/108	45/18723	1.70E-10	3.99E-09	1.68E-09	RAF1/BCL2/GAT	8
15/108	298/18723	1.74E-10	4.05E-09	1.71E-09	HSPA5/EGFR/TF	15

13/108	208/18723	2.20E-10	5.09E-09	2.14E-09	TNF/IFNG/IL4/	13
10/108	97/18723	2.21E-10	5.09E-09	2.14E-09	TNF/IFNG/TLR4	10
14/108	257/18723	2.62E-10	5.97E-09	2.52E-09	IFNG/IL1B/IL6	14
13/108	211/18723	2.63E-10	5.97E-09	2.52E-09	TNF/IL1B/KDR/	13
13/108	212/18723	2.78E-10	6.29E-09	2.65E-09	TNF/CCL2/BCL2	13
17/108	420/18723	2.84E-10	6.39E-09	2.69E-09	TNF/IL4/THBD/	17
16/108	367/18723	3.39E-10	7.58E-09	3.19E-09	TNF/IFNG/IL4/	16
11/108	135/18723	3.45E-10	7.65E-09	3.22E-09	TNF/IL6/CXCL8	11
8/108	49/18723	3.49E-10	7.65E-09	3.22E-09	TNF/IL1B/IL6/	8
8/108	49/18723	3.49E-10	7.65E-09	3.22E-09	THBD/PLAT/NOS	8
13/108	216/18723	3.51E-10	7.65E-09	3.22E-09	IFNG/IL1B/IL6	13
14/108	263/18723	3.55E-10	7.71E-09	3.25E-09	PTGS2/EGFR/AK	14
10/108	102/18723	3.65E-10	7.88E-09	3.32E-09	TNF/IL1B/IL6/	10
14/108	264/18723	3.73E-10	8.01E-09	3.37E-09	IL1B/GPT/RELA	14
18/108	490/18723	3.86E-10	8.22E-09	3.46E-09	IFNG/IL4/HSD1	18
10/108	103/18723	4.03E-10	8.53E-09	3.59E-09	TNF/IL1B/IL6/	10
11/108	137/18723	4.05E-10	8.53E-09	3.59E-09	FASN/VDR/ESR1	11
9/108	74/18723	4.08E-10	8.54E-09	3.59E-09	KDR/BAK1/TP53	9
7/108	31/18723	4.09E-10	8.54E-09	3.59E-09	RAF1/NOS3/ICA	7
8/108	50/18723	4.14E-10	8.54E-09	3.59E-09	THBD/PLAT/NOS	8
8/108	50/18723	4.14E-10	8.54E-09	3.59E-09	IL4/KDR/NFE2L	8
12/108	178/18723	4.74E-10	9.73E-09	4.10E-09	CXCL10/PTGS2/	12
12/108	179/18723	5.06E-10	1.03E-08	4.35E-09	TNF/KDR/CCL2/	12
14/108	272/18723	5.52E-10	1.12E-08	4.72E-09	IL6/BAK1/BCL2	14
12/108	181/18723	5.75E-10	1.16E-08	4.89E-09	TNF/IL1B/IL6/	12
10/108	108/18723	6.45E-10	1.30E-08	5.46E-09	TNF/IFNG/IL6/	10
8/108	53/18723	6.73E-10	1.34E-08	5.66E-09	THBD/PLAT/NOS	8
11/108	144/18723	6.91E-10	1.36E-08	5.72E-09	KDR/BAK1/TP53	11
11/108	144/18723	6.91E-10	1.36E-08	5.72E-09	TNF/KDR/EGFR/	11
11/108	144/18723	6.91E-10	1.36E-08	5.72E-09	TNF/IFNG/IL6/	11
15/108	330/18723	7.15E-10	1.40E-08	5.89E-09	BCL2/SPP1/STA	15
9/108	81/18723	9.31E-10	1.81E-08	7.62E-09	TNF/IFNG/IL6/	9
8/108	56/18723	1.06E-09	2.06E-08	8.67E-09	TLR4/VCAM1/MM	8
12/108	193/18723	1.20E-09	2.31E-08	9.74E-09	TNF/KDR/CCL2/	12
10/108	116/18723	1.31E-09	2.51E-08	1.06E-08	BAK1/BCL2/STA	10
14/108	292/18723	1.39E-09	2.64E-08	1.11E-08	TNF/IFNG/IL4/	14
17/108	467/18723	1.43E-09	2.72E-08	1.14E-08	TNF/IFNG/IL1E	17
10/108	118/18723	1.55E-09	2.92E-08	1.23E-08	IFNG/CCL2/TLF	10
6/108	21/18723	1.62E-09	3.02E-08	1.27E-08	IFNG/KDR/CCL2	6
6/108	21/18723	1.62E-09	3.02E-08	1.27E-08	IFNG/KDR/CCL2	6
12/108	199/18723	1.70E-09	3.17E-08	1.33E-08	IL1B/IL6/IL4/	12
13/108	246/18723	1.73E-09	3.19E-08	1.34E-08	HSPA5/VDR/BCI	13
9/108	87/18723	1.78E-09	3.27E-08	1.38E-08	TNF/IL1B/IL6/	9
15/108	359/18723	2.27E-09	4.15E-08	1.75E-08	TNF/IFNG/IL1E	15
7/108	39/18723	2.31E-09	4.20E-08	1.77E-08	EGFR/ICAM1/EI	7
10/108	123/18723	2.33E-09	4.22E-08	1.78E-08	PTGS2/BAK1/TI	10
15/108	362/18723	2.54E-09	4.58E-08	1.93E-08	IFNG/IL1B/IL6	15
13/108	255/18723	2.67E-09	4.80E-08	2.02E-08	CXCL10/PTGS2/	13
11/108	164/18723	2.76E-09	4.93E-08	2.08E-08	PTGS2/BCL2/TI	11
7/108	40/18723	2.78E-09	4.95E-08	2.08E-08	TNF/IL1B/IL6/	7
6/108	23/18723	2.98E-09	5.23E-08	2.20E-08	THBD/NFE2L2/I	6
6/108	23/18723	2.98E-09	5.23E-08	2.20E-08	RAF1/RELA/NFK	6
6/108	23/18723	2.98E-09	5.23E-08	2.20E-08	THBD/NFE2L2/I	6
16/108	428/18723	3.13E-09	5.46E-08	2.30E-08	TNF/IFNG/RAF1	16

12/108	210/18723	3.14E-09	5.46E-08	2.30E-08	TNF/IFNG/IL4/	12
9/108	93/18723	3.24E-09	5.61E-08	2.36E-08	THBD/HSPA5/RE	9
15/108	372/18723	3.67E-09	6.32E-08	2.66E-08	TNF/IFNG/IL1E	15
9/108	95/18723	3.92E-09	6.72E-08	2.83E-08	TNF/IL6/TLR4/	9
6/108	24/18723	3.96E-09	6.76E-08	2.85E-08	THBD/NFE2L2/F	6
10/108	130/18723	4.00E-09	6.79E-08	2.86E-08	IFNG/IL4/STAT	10
13/108	264/18723	4.06E-09	6.86E-08	2.89E-08	TNF/IFNG/IL6/	13
12/108	215/18723	4.10E-09	6.90E-08	2.91E-08	HSPA5/VDR/BCI	12
11/108	171/18723	4.28E-09	7.18E-08	3.02E-08	IL1B/IL6/IL4/	11
14/108	320/18723	4.50E-09	7.52E-08	3.17E-08	TNF/IL1B/EGFF	14
6/108	25/18723	5.19E-09	8.62E-08	3.63E-08	TNF/IL6/EGFR/	6
11/108	175/18723	5.46E-09	9.03E-08	3.80E-08	IL6/EGFR/VDR/	11
7/108	44/18723	5.61E-09	9.24E-08	3.89E-08	TNF/IL1B/EGFF	7
11/108	176/18723	5.80E-09	9.51E-08	4.00E-08	IFNG/PTGS2/KI	11
8/108	69/18723	5.89E-09	9.61E-08	4.05E-08	CXCL10/PTGS2/	8
15/108	386/18723	6.04E-09	9.82E-08	4.14E-08	TNF/IFNG/IL1E	15
11/108	177/18723	6.15E-09	9.96E-08	4.19E-08	TNF/IL1B/TLR4	11
11/108	178/18723	6.53E-09	1.05E-07	4.41E-08	BCL2/ESR1/AKT	11
11/108	178/18723	6.53E-09	1.05E-07	4.41E-08	TNF/KDR/EGFR/	11
10/108	137/18723	6.65E-09	1.06E-07	4.48E-08	IL1B/IL6/IL4/	10
6/108	26/18723	6.71E-09	1.07E-07	4.50E-08	TNF/IL6/EGFR/	6
10/108	138/18723	7.14E-09	1.13E-07	4.76E-08	IL1B/IL6/IL4/	10
11/108	181/18723	7.78E-09	1.22E-07	5.14E-08	IL1B/CXCL8/KI	11
11/108	181/18723	7.78E-09	1.22E-07	5.14E-08	IL1B/CXCL8/KI	11
11/108	182/18723	8.24E-09	1.29E-07	5.43E-08	TNF/KDR/EGFR/	11
10/108	141/18723	8.79E-09	1.36E-07	5.74E-08	IFNG/CCL2/TLF	10
10/108	141/18723	8.79E-09	1.36E-07	5.74E-08	IL6/CXCL8/IL4	10
14/108	339/18723	9.38E-09	1.45E-07	6.11E-08	TNF/IFNG/IL1E	14
10/108	143/18723	1.01E-08	1.55E-07	6.53E-08	IL1B/IL6/CXCI	10
8/108	74/18723	1.04E-08	1.59E-07	6.69E-08	EGFR/NCF1/AKT	8
6/108	28/18723	1.09E-08	1.66E-07	6.99E-08	PTGS2/RELA/NF	6
8/108	75/18723	1.15E-08	1.75E-07	7.39E-08	IL1B/BCL2/TP5	8
7/108	49/18723	1.23E-08	1.86E-07	7.83E-08	RAF1/NOS3/ICA	7
15/108	408/18723	1.27E-08	1.91E-07	8.07E-08	TNF/IL6/PTGS2	15
15/108	409/18723	1.31E-08	1.97E-07	8.30E-08	IFNG/IL1B/IL6	15
12/108	239/18723	1.34E-08	2.01E-07	8.46E-08	TNF/IL1B/TLR4	12
8/108	77/18723	1.43E-08	2.12E-07	8.95E-08	TNF/IL1B/AKT1	8
9/108	110/18723	1.44E-08	2.14E-07	9.01E-08	IL1B/SPP1/NFK	9
11/108	193/18723	1.52E-08	2.24E-07	9.44E-08	TNF/IFNG/IL6/	11
15/108	415/18723	1.59E-08	2.35E-07	9.88E-08	TNF/CXCL8/CCI	15
5/108	15/18723	1.67E-08	2.45E-07	1.03E-07	TNF/IL6/STAT3	5
14/108	357/18723	1.80E-08	2.63E-07	1.11E-07	TNF/IFNG/IL4/	14
10/108	152/18723	1.81E-08	2.63E-07	1.11E-07	TNF/IL1B/TLR4	10
9/108	113/18723	1.83E-08	2.65E-07	1.12E-07	IL1B/IL6/CXCI	9
15/108	420/18723	1.87E-08	2.70E-07	1.14E-07	IFNG/IL1B/IL6	15
11/108	197/18723	1.88E-08	2.70E-07	1.14E-07	GPT/HSPA5/BCI	11
7/108	52/18723	1.89E-08	2.70E-07	1.14E-07	BCL2/STAT3/TF	7
14/108	359/18723	1.93E-08	2.76E-07	1.16E-07	IL6/IL4/EGFR/	14
12/108	247/18723	1.94E-08	2.76E-07	1.16E-07	TNF/IL1B/TLR4	12
9/108	114/18723	1.98E-08	2.80E-07	1.18E-07	HSPA5/BAK1/BC	9
14/108	360/18723	2.00E-08	2.83E-07	1.19E-07	TNF/IFNG/IL4/	14
9/108	115/18723	2.13E-08	3.00E-07	1.26E-07	TNF/IL4/CXCL1	9
7/108	53/18723	2.16E-08	3.03E-07	1.28E-07	HSPA5/RELA/TF	7
11/108	200/18723	2.19E-08	3.06E-07	1.29E-07	TNF/IFNG/IL1E	11

14/108	363/18723	2.22E-08	3.09E-07	1.30E-07	IFNG/AKT1/MMF	14
11/108	201/18723	2.31E-08	3.20E-07	1.35E-07	IL6/IL4/EGFR/	11
14/108	365/18723	2.38E-08	3.29E-07	1.38E-07	TNF/IFNG/IL4/	14
5/108	16/18723	2.42E-08	3.32E-07	1.40E-07	IFNG/KDR/CCL2	5
10/108	157/18723	2.47E-08	3.38E-07	1.42E-07	IL6/BAK1/STAT	10
12/108	253/18723	2.53E-08	3.45E-07	1.45E-07	BAK1/CAT/VCAM	12
11/108	203/18723	2.56E-08	3.48E-07	1.47E-07	IL1B/GPT/RELA	11
12/108	254/18723	2.64E-08	3.58E-07	1.51E-07	BAK1/BCL2/STA	12
8/108	84/18723	2.86E-08	3.86E-07	1.63E-07	PTGS2/EGFR/AK	8
12/108	256/18723	2.88E-08	3.87E-07	1.63E-07	CXCL8/HSPA5/I	12
13/108	312/18723	2.97E-08	3.98E-07	1.67E-07	TNF/CXCL8/KDF	13
11/108	206/18723	2.98E-08	3.98E-07	1.67E-07	IFNG/PTGS2/ST	11
9/108	120/18723	3.10E-08	4.12E-07	1.74E-07	TNF/EGFR/INSF	9
6/108	33/18723	3.12E-08	4.13E-07	1.74E-07	TNF/IL1B/BCL2	6
6/108	33/18723	3.12E-08	4.13E-07	1.74E-07	TNF/IL1B/BCL2	6
10/108	162/18723	3.33E-08	4.38E-07	1.85E-07	TNF/IFNG/IL6/	10
15/108	441/18723	3.56E-08	4.67E-07	1.97E-07	TNF/IFNG/IL1E	15
9/108	122/18723	3.58E-08	4.68E-07	1.97E-07	IL6/CXCL8/IL4	9
11/108	210/18723	3.63E-08	4.73E-07	1.99E-07	EGFR/TP53/AHF	11
15/108	442/18723	3.67E-08	4.76E-07	2.01E-07	TNF/IFNG/IL1E	15
6/108	34/18723	3.78E-08	4.87E-07	2.05E-07	TNF/IFNG/IL1E	6
6/108	34/18723	3.78E-08	4.87E-07	2.05E-07	IL4/KDR/NFE2I	6
9/108	124/18723	4.12E-08	5.28E-07	2.22E-07	IFNG/IL4/KDR/	9
9/108	124/18723	4.12E-08	5.28E-07	2.22E-07	PTGS2/CAT/CEF	9
12/108	265/18723	4.21E-08	5.36E-07	2.26E-07	IFNG/IL6/IL4/	12
12/108	265/18723	4.21E-08	5.36E-07	2.26E-07	IFNG/IL6/IL4/	12
6/108	35/18723	4.54E-08	5.73E-07	2.41E-07	PTGS2/VDR/SPF	6
6/108	35/18723	4.54E-08	5.73E-07	2.41E-07	PTGS2/EGFR/AK	6
7/108	59/18723	4.65E-08	5.82E-07	2.45E-07	BAK1/BCL2/TP5	7
7/108	59/18723	4.65E-08	5.82E-07	2.45E-07	TNF/IFNG/IL1E	7
7/108	59/18723	4.65E-08	5.82E-07	2.45E-07	THBD/NFE2L2/F	7
10/108	170/18723	5.26E-08	6.56E-07	2.76E-07	TNF/IFNG/IL6/	10
10/108	171/18723	5.56E-08	6.91E-07	2.91E-07	TNF/IFNG/IL1E	10
14/108	391/18723	5.61E-08	6.95E-07	2.93E-07	TNF/IFNG/IL1E	14
13/108	330/18723	5.73E-08	7.08E-07	2.98E-07	TNF/IL1B/KDR/	13
10/108	173/18723	6.20E-08	7.63E-07	3.22E-07	TNF/IFNG/HSD1	10
12/108	275/18723	6.33E-08	7.76E-07	3.27E-07	IFNG/IL1B/CXC	12
5/108	19/18723	6.34E-08	7.76E-07	3.27E-07	TNF/IFNG/IL4/	5
8/108	93/18723	6.40E-08	7.77E-07	3.27E-07	TNF/IFNG/IL1E	8
8/108	93/18723	6.40E-08	7.77E-07	3.27E-07	CXCL10/PTGS2/	8
6/108	37/18723	6.44E-08	7.79E-07	3.28E-07	AKT1/NFKB1/IN	6
10/108	174/18723	6.55E-08	7.91E-07	3.33E-07	TNF/IL1B/IL4/	10
12/108	276/18723	6.58E-08	7.91E-07	3.33E-07	TNF/IFNG/PTGS	12
7/108	62/18723	6.61E-08	7.91E-07	3.33E-07	IFNG/TLR4/CD4	7
7/108	62/18723	6.61E-08	7.91E-07	3.33E-07	IFNG/TLR4/CD4	7
11/108	223/18723	6.70E-08	7.99E-07	3.36E-07	IL6/CXCL8/IL4	11
8/108	94/18723	6.96E-08	8.27E-07	3.48E-07	IL6/CXCL8/IL4	8
9/108	132/18723	7.10E-08	8.40E-07	3.54E-07	BCL2/TP53/ICA	9
12/108	278/18723	7.12E-08	8.41E-07	3.54E-07	TNF/IFNG/IL1E	12
7/108	63/18723	7.41E-08	8.69E-07	3.66E-07	IL4/KDR/CERS1	7
12/108	279/18723	7.41E-08	8.69E-07	3.66E-07	TNF/IFNG/IL4/	12
8/108	95/18723	7.56E-08	8.82E-07	3.71E-07	HSPA5/BCL2/ES	8
8/108	95/18723	7.56E-08	8.82E-07	3.71E-07	TLR4/BCL2/AKT	8
6/108	38/18723	7.61E-08	8.84E-07	3.72E-07	TNF/IFNG/IL4/	6

11/108	226/18723	7.68E-08	8.86E-07	3.73E-07	TNF/IL1B/IL6/	11
10/108	177/18723	7.70E-08	8.86E-07	3.73E-07	PTGS2/BCL2/TI	10
10/108	177/18723	7.70E-08	8.86E-07	3.73E-07	IL1B/HSPA5/EC	10
10/108	178/18723	8.11E-08	9.32E-07	3.92E-07	IFNG/GPT/STAT	10
15/108	470/18723	8.19E-08	9.37E-07	3.95E-07	TNF/IL1B/PTGS	15
8/108	96/18723	8.21E-08	9.37E-07	3.95E-07	EGFR/SPP1/AKT	8
5/108	20/18723	8.42E-08	9.55E-07	4.02E-07	TNF/IFNG/NFKI	5
5/108	20/18723	8.42E-08	9.55E-07	4.02E-07	TNF/IL1B/IL6/	5
14/108	406/18723	8.92E-08	1.01E-06	4.25E-07	IFNG/CXCL10/I	14
15/108	475/18723	9.39E-08	1.06E-06	4.46E-07	TNF/IFNG/IL4/	15
10/108	181/18723	9.50E-08	1.07E-06	4.49E-07	TNF/IFNG/IL6/	10
10/108	182/18723	1.00E-07	1.12E-06	4.72E-07	CXCL10/BAK1/I	10
7/108	66/18723	1.03E-07	1.15E-06	4.84E-07	PTGS2/EGFR/AI	7
8/108	99/18723	1.05E-07	1.16E-06	4.90E-07	TNF/BAK1/BCL2	8
6/108	40/18723	1.05E-07	1.16E-06	4.90E-07	IL6/THBD/TLR4	6
12/108	290/18723	1.13E-07	1.25E-06	5.26E-07	IFNG/PTGS2/ST	12
8/108	100/18723	1.13E-07	1.25E-06	5.26E-07	HSPA5/BCL2/ES	8
11/108	235/18723	1.14E-07	1.25E-06	5.28E-07	TNF/RAF1/PTGS	11
7/108	67/18723	1.14E-07	1.25E-06	5.28E-07	VDR/ESR1/AKT1	7
12/108	291/18723	1.17E-07	1.28E-06	5.40E-07	IL4/KDR/TP53/	12
6/108	41/18723	1.22E-07	1.33E-06	5.60E-07	IFNG/TLR4/CD4	6
8/108	101/18723	1.22E-07	1.33E-06	5.60E-07	IL1B/IL6/IL4/	8
11/108	237/18723	1.24E-07	1.35E-06	5.67E-07	TNF/IFNG/TLR4	11
11/108	237/18723	1.24E-07	1.35E-06	5.67E-07	TNF/IFNG/PAH/	11
8/108	102/18723	1.32E-07	1.42E-06	6.00E-07	CXCL10/BAK1/I	8
9/108	142/18723	1.33E-07	1.43E-06	6.03E-07	TNF/IFNG/IL1I	9
12/108	295/18723	1.36E-07	1.46E-06	6.13E-07	TNF/IFNG/IL1I	12
12/108	296/18723	1.41E-07	1.50E-06	6.34E-07	TNF/PTGS2/EGI	12
5/108	22/18723	1.42E-07	1.50E-06	6.34E-07	NOS3/NOS2/MAI	5
5/108	22/18723	1.42E-07	1.50E-06	6.34E-07	TNF/IL6/STAT3	5
6/108	42/18723	1.42E-07	1.50E-06	6.34E-07	TNF/RELA/STAT	6
8/108	103/18723	1.42E-07	1.51E-06	6.34E-07	TNF/IFNG/STAT	8
14/108	422/18723	1.43E-07	1.51E-06	6.37E-07	CXCL10/PTGS2/	14
9/108	144/18723	1.50E-07	1.58E-06	6.65E-07	PTGS2/VDR/SPI	9
6/108	43/18723	1.64E-07	1.71E-06	7.21E-07	IFNG/IL4/STAT	6
6/108	43/18723	1.64E-07	1.71E-06	7.21E-07	IFNG/IL4/STAT	6
6/108	43/18723	1.64E-07	1.71E-06	7.21E-07	TNF/IL1B/BCL2	6
8/108	106/18723	1.78E-07	1.85E-06	7.80E-07	AKT1/MAPK14/I	8
12/108	303/18723	1.81E-07	1.88E-06	7.91E-07	TNF/IFNG/IL1I	12
10/108	194/18723	1.82E-07	1.88E-06	7.91E-07	IFNG/IL6/IL4/	10
7/108	72/18723	1.89E-07	1.93E-06	8.15E-07	TNF/IL6/TLR4/	7
7/108	72/18723	1.89E-07	1.93E-06	8.15E-07	TNF/IL6/TLR4/	7
7/108	72/18723	1.89E-07	1.93E-06	8.15E-07	THBD/NFE2L2/I	7
6/108	44/18723	1.89E-07	1.93E-06	8.15E-07	AKT1/NFKB1/IN	6
10/108	196/18723	2.00E-07	2.04E-06	8.59E-07	TNF/GPT/TP53/	10
9/108	149/18723	2.01E-07	2.04E-06	8.61E-07	IL1B/SPP1/AKT	9
12/108	306/18723	2.02E-07	2.04E-06	8.61E-07	TNF/IFNG/IL1I	12
4/108	10/18723	2.14E-07	2.15E-06	9.07E-07	TNF/IFNG/NFKI	4
4/108	10/18723	2.14E-07	2.15E-06	9.07E-07	THBD/F2/SERPI	4
4/108	10/18723	2.14E-07	2.15E-06	9.07E-07	TP53/CERS1/VI	4
6/108	45/18723	2.17E-07	2.18E-06	9.18E-07	BCL2/AKT1/NFI	6
12/108	309/18723	2.24E-07	2.24E-06	9.44E-07	TNF/IL1B/KDR/	12
9/108	151/18723	2.25E-07	2.25E-06	9.47E-07	PTGS2/BCL2/TI	9
5/108	24/18723	2.27E-07	2.25E-06	9.48E-07	TNF/IL6/STAT3	5

5/108	24/18723	2.27E-07	2.25E-06	9.48E-07	TNF/IL6/STAT3	5
7/108	74/18723	2.29E-07	2.27E-06	9.54E-07	KDR/BAK1/BCL2	7
12/108	310/18723	2.32E-07	2.29E-06	9.64E-07	IL6/CXCL8/IL4	12
8/108	110/18723	2.37E-07	2.34E-06	9.85E-07	TNF/IL1B/AKT1	8
9/108	152/18723	2.38E-07	2.34E-06	9.86E-07	IL4/SOAT1/CAT	9
14/108	443/18723	2.59E-07	2.54E-06	1.07E-06	TNF/IL6/TP53/	14
11/108	255/18723	2.60E-07	2.54E-06	1.07E-06	PTGS2/EGFR/IN	11
9/108	154/18723	2.66E-07	2.60E-06	1.09E-06	TNF/IFNG/IL1B	9
8/108	112/18723	2.73E-07	2.65E-06	1.11E-06	TNF/IL1B/TLR4	8
8/108	112/18723	2.73E-07	2.65E-06	1.11E-06	IFNG/IL6/IL4/	8
7/108	76/18723	2.75E-07	2.66E-06	1.12E-06	TLR4/EGFR/TP5	7
9/108	155/18723	2.81E-07	2.70E-06	1.14E-06	IFNG/PTGS2/ST	9
5/108	25/18723	2.82E-07	2.70E-06	1.14E-06	THBD/PLAT/F2/	5
5/108	25/18723	2.82E-07	2.70E-06	1.14E-06	RELA/NFKB1/NF	5
5/108	25/18723	2.82E-07	2.70E-06	1.14E-06	TNF/IL6/RELA/	5
6/108	47/18723	2.84E-07	2.70E-06	1.14E-06	HSPA5/TP53/NF	6
6/108	47/18723	2.84E-07	2.70E-06	1.14E-06	HSPA5/TP53/HS	6
14/108	448/18723	2.96E-07	2.82E-06	1.19E-06	TNF/IL1B/IL6/	14
9/108	156/18723	2.97E-07	2.82E-06	1.19E-06	HSPA5/BCL2/TP	9
11/108	259/18723	3.03E-07	2.87E-06	1.21E-06	EGFR/BCL2/AKT	11
12/108	318/18723	3.04E-07	2.87E-06	1.21E-06	IFNG/IL4/CAT/	12
8/108	114/18723	3.13E-07	2.94E-06	1.24E-06	IFNG/IL6/IL4/	8
8/108	114/18723	3.13E-07	2.94E-06	1.24E-06	TNF/IL1B/AKT1	8
12/108	319/18723	3.15E-07	2.95E-06	1.24E-06	TNF/IFNG/IL1B	12
6/108	48/18723	3.23E-07	3.01E-06	1.27E-06	TNF/IFNG/RELA	6
10/108	207/18723	3.32E-07	3.09E-06	1.30E-06	KDR/EGFR/STAT	10
4/108	11/18723	3.35E-07	3.10E-06	1.31E-06	TNF/IL1B/PTGS	4
4/108	11/18723	3.35E-07	3.10E-06	1.31E-06	CERS1/MMP9/MM	4
10/108	208/18723	3.47E-07	3.20E-06	1.35E-06	CCL2/BCL2/STA	10
5/108	26/18723	3.47E-07	3.20E-06	1.35E-06	HSD17B6/SPP1/	5
5/108	26/18723	3.47E-07	3.20E-06	1.35E-06	PTGS2/VDR/SPP	5
9/108	159/18723	3.49E-07	3.21E-06	1.35E-06	IFNG/PTGS2/ST	9
8/108	116/18723	3.58E-07	3.27E-06	1.38E-06	TNF/IL1B/AKT1	8
8/108	116/18723	3.58E-07	3.27E-06	1.38E-06	PTGS2/CAT/NOS	8
10/108	209/18723	3.62E-07	3.30E-06	1.39E-06	TNF/RAF1/PTGS	10
6/108	49/18723	3.66E-07	3.33E-06	1.40E-06	TNF/IL6/EGFR/	6
11/108	265/18723	3.81E-07	3.46E-06	1.46E-06	TLR4/VDR/RELA	11
9/108	161/18723	3.88E-07	3.51E-06	1.48E-06	PTGS2/BCL2/TP	9
8/108	118/18723	4.08E-07	3.66E-06	1.54E-06	TNF/IL6/VDR/E	8
8/108	118/18723	4.08E-07	3.66E-06	1.54E-06	TNF/IL1B/AKT1	8
8/108	118/18723	4.08E-07	3.66E-06	1.54E-06	IFNG/MMP9/GAT	8
14/108	461/18723	4.18E-07	3.75E-06	1.58E-06	TNF/RAF1/PTGS	14
11/108	268/18723	4.26E-07	3.81E-06	1.60E-06	IFNG/IL1B/TP5	11
14/108	463/18723	4.41E-07	3.93E-06	1.65E-06	TNF/IFNG/IL1B	14
10/108	214/18723	4.50E-07	4.01E-06	1.69E-06	CXCL10/HSPA5/	10
8/108	120/18723	4.64E-07	4.12E-06	1.73E-06	TNF/IFNG/IL4/	8
7/108	82/18723	4.65E-07	4.12E-06	1.73E-06	TNF/IFNG/IL1B	7
12/108	331/18723	4.67E-07	4.12E-06	1.74E-06	TNF/IFNG/IL1B	12
6/108	52/18723	5.26E-07	4.62E-06	1.95E-06	TNF/IL6/EGFR/	6
6/108	52/18723	5.26E-07	4.62E-06	1.95E-06	TNF/IL6/EGFR/	6
7/108	84/18723	5.49E-07	4.79E-06	2.02E-06	IFNG/TP53/NFE	7
7/108	84/18723	5.49E-07	4.79E-06	2.02E-06	IL1B/SPP1/NFK	7
7/108	84/18723	5.49E-07	4.79E-06	2.02E-06	IFNG/IL4/STAT	7
11/108	275/18723	5.50E-07	4.79E-06	2.02E-06	IFNG/IL6/IL4/	11

10/108	219/18723	5.57E-07	4.84E-06	2.04E-06	IFNG/CXCL10/C	10
8/108	123/18723	5.61E-07	4.86E-06	2.05E-06	IL6/THBD/TLR4	8
11/108	276/18723	5.70E-07	4.93E-06	2.08E-06	HSPA5/BAK1/BC	11
10/108	220/18723	5.80E-07	5.01E-06	2.11E-06	IL6/CXCL8/IL4	10
6/108	53/18723	5.90E-07	5.06E-06	2.13E-06	IL1B/PTGS2/SF	6
6/108	53/18723	5.90E-07	5.06E-06	2.13E-06	RAF1/NOS3/NOS	6
6/108	53/18723	5.90E-07	5.06E-06	2.13E-06	TNF/IL6/EGFR/	6
7/108	85/18723	5.95E-07	5.09E-06	2.14E-06	IL6/TP53/AKT1	7
10/108	221/18723	6.05E-07	5.16E-06	2.17E-06	HSPA5/BCL2/ES	10
5/108	29/18723	6.19E-07	5.23E-06	2.20E-06	IL6/BCL2/STAT	5
5/108	29/18723	6.19E-07	5.23E-06	2.20E-06	AKT1/INSR/IGF	5
5/108	29/18723	6.19E-07	5.23E-06	2.20E-06	AKT1/INSR/IGF	5
5/108	29/18723	6.19E-07	5.23E-06	2.20E-06	TNF/IL6/STAT3	5
11/108	279/18723	6.35E-07	5.34E-06	2.25E-06	IFNG/IL6/IL4/	11
8/108	125/18723	6.35E-07	5.34E-06	2.25E-06	CXCL8/IL4/CXC	8
7/108	86/18723	6.45E-07	5.42E-06	2.28E-06	IFNG/IL4/STAT	7
6/108	54/18723	6.60E-07	5.50E-06	2.32E-06	CXCL10/BAK1/H	6
6/108	54/18723	6.60E-07	5.50E-06	2.32E-06	TNF/IL6/EGFR/	6
6/108	54/18723	6.60E-07	5.50E-06	2.32E-06	TNF/IL6/EGFR/	6
6/108	54/18723	6.60E-07	5.50E-06	2.32E-06	TNF/RELA/STAT	6
7/108	87/18723	6.98E-07	5.80E-06	2.44E-06	IFNG/PTGS2/AK	7
14/108	482/18723	7.12E-07	5.90E-06	2.49E-06	EGFR/BCL2/SPF	14
8/108	127/18723	7.16E-07	5.92E-06	2.49E-06	IL6/RAF1/BAK1	8
4/108	13/18723	7.19E-07	5.93E-06	2.50E-06	TNF/IFNG/NFKE	4
6/108	55/18723	7.38E-07	6.06E-06	2.55E-06	TNF/RELA/STAT	6
6/108	55/18723	7.38E-07	6.06E-06	2.55E-06	TNF/IFNG/RELA	6
5/108	30/18723	7.39E-07	6.06E-06	2.55E-06	HSD17B6/SPP1/	5
13/108	414/18723	7.69E-07	6.29E-06	2.65E-06	EGFR/BCL2/SPF	13
10/108	227/18723	7.73E-07	6.31E-06	2.66E-06	HSPA5/BCL2/ES	10
7/108	89/18723	8.16E-07	6.64E-06	2.80E-06	IFNG/TP53/NFE	7
6/108	56/18723	8.23E-07	6.68E-06	2.82E-06	AKT1/MAPK14/I	6
10/108	230/18723	8.71E-07	7.06E-06	2.97E-06	IL6/CXCL8/IL4	10
6/108	57/18723	9.15E-07	7.39E-06	3.11E-06	TNF/IFNG/RELA	6
6/108	57/18723	9.15E-07	7.39E-06	3.11E-06	TNF/IL6/RELA/	6
11/108	290/18723	9.28E-07	7.48E-06	3.15E-06	CXCL8/EGFR/IN	11
10/108	232/18723	9.42E-07	7.58E-06	3.19E-06	TNF/PTGS2/KDF	10
13/108	422/18723	9.53E-07	7.65E-06	3.22E-06	IFNG/PTGS2/HS	13
11/108	291/18723	9.60E-07	7.69E-06	3.24E-06	IL1B/IL6/BCL2	11
4/108	14/18723	1.00E-06	8.01E-06	3.37E-06	THBD/F2/SERPI	4
12/108	356/18723	1.01E-06	8.02E-06	3.38E-06	TNF/IL1B/IL6/	12
6/108	58/18723	1.02E-06	8.08E-06	3.40E-06	BCL2/TP53/AKT	6
8/108	133/18723	1.02E-06	8.08E-06	3.40E-06	PTGS2/KDR/AKT	8
10/108	235/18723	1.06E-06	8.39E-06	3.53E-06	TNF/IFNG/IL1E	10
10/108	236/18723	1.10E-06	8.70E-06	3.66E-06	IL1B/IL6/BCL2	10
6/108	59/18723	1.13E-06	8.88E-06	3.74E-06	TNF/AKT1/NFE2	6
10/108	237/18723	1.14E-06	9.00E-06	3.79E-06	TNF/GPT/TP53/	10
8/108	136/18723	1.21E-06	9.46E-06	3.99E-06	IFNG/PTGS2/AK	8
13/108	432/18723	1.24E-06	9.70E-06	4.09E-06	TNF/RAF1/PTGS	13
9/108	185/18723	1.25E-06	9.75E-06	4.11E-06	TNF/EGFR/INSF	9
7/108	95/18723	1.27E-06	9.90E-06	4.17E-06	TNF/IFNG/SOAT	7
8/108	137/18723	1.27E-06	9.90E-06	4.17E-06	IL4/SOAT1/CAT	8
8/108	137/18723	1.27E-06	9.90E-06	4.17E-06	BAK1/CAT/VCAM	8
11/108	301/18723	1.34E-06	1.03E-05	4.36E-06	IFNG/IL1B/PTC	11
11/108	301/18723	1.34E-06	1.03E-05	4.36E-06	IFNG/IL1B/PTC	11

7/108	96/18723	1.37E-06	1.05E-05	4.44E-06	TNF/IL1B/IL6/	7
7/108	97/18723	1.46E-06	1.13E-05	4.75E-06	GPT/AKT1/NFKE	7
13/108	439/18723	1.48E-06	1.14E-05	4.79E-06	TNF/IL1B/PTGS	13
6/108	62/18723	1.51E-06	1.16E-05	4.89E-06	IFNG/IL1B/PTC	6
13/108	440/18723	1.52E-06	1.16E-05	4.89E-06	TNF/IL1B/IL6/	13
13/108	440/18723	1.52E-06	1.16E-05	4.89E-06	TNF/IL1B/PTGS	13
7/108	98/18723	1.57E-06	1.20E-05	5.03E-06	TNF/IL1B/IL6/	7
7/108	98/18723	1.57E-06	1.20E-05	5.03E-06	PTGS2/BCL2/AK	7
8/108	141/18723	1.58E-06	1.20E-05	5.07E-06	IL6/IL4/CD40I	8
5/108	35/18723	1.65E-06	1.25E-05	5.24E-06	CXCL10/PTGS2/	5
5/108	35/18723	1.65E-06	1.25E-05	5.24E-06	PTGS2/AKT1/NC	5
6/108	63/18723	1.67E-06	1.26E-05	5.30E-06	BAK1/BCL2/TP5	6
7/108	99/18723	1.68E-06	1.27E-05	5.34E-06	IFNG/IL4/ICAM	7
9/108	192/18723	1.70E-06	1.28E-05	5.38E-06	TNF/IL1B/TLR4	9
8/108	143/18723	1.76E-06	1.32E-05	5.56E-06	TNF/IL1B/TLR4	8
12/108	376/18723	1.78E-06	1.33E-05	5.62E-06	GPT/PAH/HSD17	12
10/108	249/18723	1.79E-06	1.34E-05	5.63E-06	TNF/IFNG/IL1E	10
4/108	16/18723	1.81E-06	1.35E-05	5.68E-06	TNF/IFNG/IL1E	4
5/108	36/18723	1.90E-06	1.41E-05	5.95E-06	TNF/IL1B/IL6/	5
5/108	36/18723	1.90E-06	1.41E-05	5.95E-06	AKT1/INSR/IGF	5
7/108	101/18723	1.92E-06	1.43E-05	6.01E-06	PTGS2/CAT/NOS	7
9/108	195/18723	1.93E-06	1.43E-05	6.02E-06	TNF/IL1B/TLR4	9
7/108	102/18723	2.06E-06	1.52E-05	6.39E-06	STAT3/TP53/AK	7
8/108	146/18723	2.06E-06	1.52E-05	6.39E-06	GPT/STAT3/TP5	8
9/108	197/18723	2.10E-06	1.54E-05	6.51E-06	TNF/BAK1/STAT	9
8/108	147/18723	2.17E-06	1.59E-05	6.70E-06	IL4/SOAT1/CAT	8
5/108	37/18723	2.19E-06	1.60E-05	6.74E-06	STAT3/AKT1/AS	5
7/108	103/18723	2.20E-06	1.60E-05	6.74E-06	TNF/IL1B/IL6/	7
6/108	66/18723	2.20E-06	1.60E-05	6.74E-06	TNF/IL1B/IL6/	6
6/108	66/18723	2.20E-06	1.60E-05	6.74E-06	TNF/IFNG/IL1E	6
11/108	317/18723	2.21E-06	1.61E-05	6.77E-06	TNF/IFNG/IL1E	11
8/108	148/18723	2.28E-06	1.65E-05	6.97E-06	CXCL8/IL4/CXC	8
13/108	457/18723	2.31E-06	1.67E-05	7.04E-06	TNF/IL1B/CCL2	13
4/108	17/18723	2.35E-06	1.70E-05	7.16E-06	CCL2/TP53/CAS	4
10/108	257/18723	2.37E-06	1.71E-05	7.21E-06	TNF/GPT/TP53/	10
8/108	149/18723	2.40E-06	1.73E-05	7.27E-06	TNF/IFNG/IL1E	8
6/108	67/18723	2.40E-06	1.73E-05	7.27E-06	TNF/IFNG/RELA	6
11/108	320/18723	2.42E-06	1.74E-05	7.31E-06	TNF/IL1B/HSD1	11
5/108	38/18723	2.51E-06	1.79E-05	7.56E-06	SOAT1/NFKB1/S	5
5/108	38/18723	2.51E-06	1.79E-05	7.56E-06	SOAT1/NFKB1/S	5
9/108	202/18723	2.58E-06	1.84E-05	7.75E-06	TNF/CXCL8/KDF	9
6/108	68/18723	2.62E-06	1.86E-05	7.85E-06	IFNG/IL6/IL4/	6
6/108	68/18723	2.62E-06	1.86E-05	7.85E-06	TNF/IFNG/RELA	6
8/108	151/18723	2.65E-06	1.87E-05	7.90E-06	TNF/PTGS2/KDF	8
8/108	151/18723	2.65E-06	1.87E-05	7.90E-06	TNF/KDR/VDR/E	8
7/108	106/18723	2.66E-06	1.88E-05	7.93E-06	IL6/BAK1/TP53	7
8/108	152/18723	2.78E-06	1.96E-05	8.25E-06	TNF/IL1B/AKT1	8
8/108	152/18723	2.78E-06	1.96E-05	8.25E-06	PTGS2/CAT/NOS	8
9/108	204/18723	2.80E-06	1.97E-05	8.28E-06	EGFR/ESR2/ESF	9
11/108	325/18723	2.81E-06	1.97E-05	8.30E-06	TNF/CXCL10/KI	11
6/108	69/18723	2.86E-06	1.99E-05	8.38E-06	IFNG/IL6/IL4/	6
6/108	69/18723	2.86E-06	1.99E-05	8.38E-06	IFNG/IL6/IL4/	6
6/108	69/18723	2.86E-06	1.99E-05	8.38E-06	TNF/IFNG/NFKE	6
6/108	69/18723	2.86E-06	1.99E-05	8.38E-06	TNF/IL1B/TLR4	6

11/108	327/18723	2.98E-06	2.07E-05	8.72E-06	IL4/TLR4/NCF1	11
4/108	18/18723	3.01E-06	2.08E-05	8.78E-06	TNF/IL1B/PTGS	4
4/108	18/18723	3.01E-06	2.08E-05	8.78E-06	IL1B/IL6/IL4/	4
12/108	396/18723	3.04E-06	2.10E-05	8.86E-06	IFNG/IL4/FASN	12
6/108	70/18723	3.11E-06	2.15E-05	9.04E-06	TNF/CCL2/VCAM	6
12/108	398/18723	3.20E-06	2.21E-05	9.29E-06	IL1B/SOAT1/SF	12
7/108	109/18723	3.21E-06	2.21E-05	9.29E-06	IL1B/IL6/IL4/	7
5/108	40/18723	3.26E-06	2.24E-05	9.42E-06	TNF/IL6/RELA/	5
8/108	156/18723	3.38E-06	2.31E-05	9.73E-06	IFNG/IL6/IL4/	8
6/108	71/18723	3.38E-06	2.31E-05	9.73E-06	TNF/IFNG/IL1E	6
6/108	71/18723	3.38E-06	2.31E-05	9.73E-06	TNF/MMP2/HSF1	6
10/108	268/18723	3.45E-06	2.35E-05	9.90E-06	TNF/IFNG/IL1E	10
9/108	210/18723	3.55E-06	2.41E-05	1.02E-05	IL4/THBD/ERBE	9
7/108	111/18723	3.62E-06	2.46E-05	1.03E-05	KDR/STAT3/AKT	7
7/108	111/18723	3.62E-06	2.46E-05	1.03E-05	TNF/KDR/EGFR/	7
11/108	334/18723	3.65E-06	2.47E-05	1.04E-05	IL6/IL4/TLR4/	11
6/108	72/18723	3.67E-06	2.48E-05	1.04E-05	AKT1/NFKB1/IN	6
6/108	72/18723	3.67E-06	2.48E-05	1.04E-05	HSPA5/TP53/GA	6
5/108	41/18723	3.70E-06	2.48E-05	1.05E-05	IL6/IL4/TLR4/	5
5/108	41/18723	3.70E-06	2.48E-05	1.05E-05	IL4/STAT3/NOS	5
4/108	19/18723	3.80E-06	2.54E-05	1.07E-05	THBD/NOS3/F2/	4
7/108	112/18723	3.85E-06	2.57E-05	1.08E-05	TNF/IL1B/TLR4	7
11/108	337/18723	3.98E-06	2.66E-05	1.12E-05	IFNG/IL1B/PTC	11
8/108	160/18723	4.07E-06	2.71E-05	1.14E-05	PTGS2/BCL2/AK	8
8/108	160/18723	4.07E-06	2.71E-05	1.14E-05	CXCL10/BAK1/E	8
8/108	160/18723	4.07E-06	2.71E-05	1.14E-05	IFNG/IL1B/PTC	8
5/108	42/18723	4.18E-06	2.76E-05	1.16E-05	IL4/STAT3/NOS	5
5/108	42/18723	4.18E-06	2.76E-05	1.16E-05	NFE2L2/IL1A/H	5
6/108	74/18723	4.32E-06	2.85E-05	1.20E-05	TNF/AKT1/NFE2	6
6/108	74/18723	4.32E-06	2.85E-05	1.20E-05	TLR4/AKT1/NFE	6
8/108	162/18723	4.47E-06	2.94E-05	1.24E-05	AKT1/NFKBIA/J	8
7/108	115/18723	4.59E-06	3.02E-05	1.27E-05	HSPA5/RELA/IC	7
9/108	217/18723	4.63E-06	3.04E-05	1.28E-05	TNF/KDR/CCL2/	9
6/108	75/18723	4.67E-06	3.05E-05	1.28E-05	IFNG/IL6/IL4/	6
6/108	75/18723	4.67E-06	3.05E-05	1.28E-05	ESR1/GATA3/CA	6
6/108	75/18723	4.67E-06	3.05E-05	1.28E-05	IFNG/GPT/AKT1	6
5/108	43/18723	4.70E-06	3.05E-05	1.28E-05	TNF/IFNG/IL6/	5
5/108	43/18723	4.70E-06	3.05E-05	1.28E-05	IL1B/INSR/IL1	5
5/108	43/18723	4.70E-06	3.05E-05	1.28E-05	VDR/ESR1/PML/	5
5/108	43/18723	4.70E-06	3.05E-05	1.28E-05	IL1B/SPP1/NOS	5
4/108	20/18723	4.72E-06	3.05E-05	1.29E-05	TNF/IFNG/NFKE	4
4/108	20/18723	4.72E-06	3.05E-05	1.29E-05	TNF/IFNG/RELA	4
13/108	489/18723	4.83E-06	3.11E-05	1.31E-05	IFNG/IL4/FASN	13
7/108	116/18723	4.86E-06	3.12E-05	1.32E-05	IL1B/RELA/AKT	7
7/108	116/18723	4.86E-06	3.12E-05	1.32E-05	TNF/IFNG/IL6/	7
8/108	164/18723	4.89E-06	3.13E-05	1.32E-05	TNF/IL1B/IL6/	8
8/108	164/18723	4.89E-06	3.13E-05	1.32E-05	TNF/CCL2/VDR/	8
8/108	164/18723	4.89E-06	3.13E-05	1.32E-05	TNF/PTGS2/CCI	8
10/108	279/18723	4.93E-06	3.14E-05	1.32E-05	TNF/PTGS2/KDF	10
9/108	219/18723	4.99E-06	3.18E-05	1.34E-05	EGFR/BCL2/TP5	9
12/108	416/18723	5.03E-06	3.20E-05	1.35E-05	IFNG/IL4/FASN	12
6/108	76/18723	5.05E-06	3.20E-05	1.35E-05	IL6/EGFR/BAK1	6
6/108	76/18723	5.05E-06	3.20E-05	1.35E-05	PTGS2/KDR/AKT	6
8/108	165/18723	5.12E-06	3.23E-05	1.36E-05	TNF/IFNG/IL1E	8

8/108	165/18723	5.12E-06	3.23E-05	1.36E-05	TNF/IFNG/IL1E	8
7/108	117/18723	5.14E-06	3.25E-05	1.37E-05	CXCL10/CCL2/C	7
9/108	220/18723	5.18E-06	3.26E-05	1.37E-05	TNF/IL1B/FASN	9
10/108	281/18723	5.25E-06	3.30E-05	1.39E-05	TNF/IL1B/TLR4	10
5/108	44/18723	5.28E-06	3.30E-05	1.39E-05	AKT1/INSR/IGF	5
5/108	44/18723	5.28E-06	3.30E-05	1.39E-05	TNF/IFNG/NFKE	5
5/108	44/18723	5.28E-06	3.30E-05	1.39E-05	AKT1/INSR/IGF	5
5/108	44/18723	5.28E-06	3.30E-05	1.39E-05	EGFR/NCF1/AKT	5
10/108	282/18723	5.41E-06	3.38E-05	1.42E-05	IFNG/IL1B/IL4	10
13/108	495/18723	5.51E-06	3.43E-05	1.44E-05	IL6/PAH/HSD17	13
8/108	167/18723	5.59E-06	3.48E-05	1.46E-05	TNF/IL1B/TLR4	8
13/108	497/18723	5.75E-06	3.57E-05	1.50E-05	IFNG/IL4/FASN	13
7/108	119/18723	5.76E-06	3.57E-05	1.50E-05	GPT/TP53/AKT1	7
4/108	21/18723	5.81E-06	3.59E-05	1.51E-05	KDR/EGFR/CAV1	4
5/108	45/18723	5.92E-06	3.65E-05	1.54E-05	IL6/RELA/STAT	5
5/108	45/18723	5.92E-06	3.65E-05	1.54E-05	TLR4/VCAM1/IC	5
7/108	120/18723	6.08E-06	3.74E-05	1.58E-05	IL1B/AKT1/HMG	7
6/108	79/18723	6.33E-06	3.89E-05	1.64E-05	TNF/KDR/CAT/N	6
5/108	46/18723	6.61E-06	4.05E-05	1.71E-05	HSPA5/TP53/NF	5
9/108	227/18723	6.68E-06	4.09E-05	1.72E-05	KDR/EGFR/AKT1	9
7/108	122/18723	6.79E-06	4.15E-05	1.75E-05	CXCL10/CCL2/E	7
6/108	80/18723	6.81E-06	4.15E-05	1.75E-05	TNF/MMP2/HSF1	6
4/108	22/18723	7.07E-06	4.31E-05	1.81E-05	VDR/ESR1/PML/	4
9/108	229/18723	7.17E-06	4.36E-05	1.84E-05	IFNG/IL1B/PTC	9
11/108	359/18723	7.25E-06	4.39E-05	1.85E-05	TNF/IFNG/IL1E	11
11/108	359/18723	7.25E-06	4.39E-05	1.85E-05	TNF/IL4/CCL2/	11
8/108	173/18723	7.25E-06	4.39E-05	1.85E-05	IFNG/MMP9/GAT	8
6/108	81/18723	7.32E-06	4.42E-05	1.86E-05	TNF/IL6/EGFR/	6
5/108	47/18723	7.36E-06	4.43E-05	1.87E-05	EGFR/NOS3/SOD	5
5/108	47/18723	7.36E-06	4.43E-05	1.87E-05	TP53/CERS1/HS	5
11/108	360/18723	7.44E-06	4.48E-05	1.89E-05	TNF/IFNG/IL1E	11
8/108	174/18723	7.57E-06	4.54E-05	1.91E-05	BCL2/AKT1/INS	8
9/108	231/18723	7.69E-06	4.61E-05	1.94E-05	TNF/IFNG/IL1E	9
12/108	434/18723	7.73E-06	4.63E-05	1.95E-05	TNF/IL4/CCL2/	12
6/108	82/18723	7.86E-06	4.70E-05	1.98E-05	HSPA5/BAK1/NF	6
8/108	175/18723	7.89E-06	4.71E-05	1.98E-05	TNF/IFNG/EGFF	8
5/108	48/18723	8.18E-06	4.86E-05	2.05E-05	TNF/IL1B/IL6/	5
5/108	48/18723	8.18E-06	4.86E-05	2.05E-05	BAK1/TP53/AKT	5
8/108	176/18723	8.23E-06	4.89E-05	2.06E-05	TNF/PTGS2/KDF	8
11/108	364/18723	8.26E-06	4.90E-05	2.06E-05	TNF/IFNG/IL1E	11
6/108	83/18723	8.43E-06	4.98E-05	2.10E-05	IFNG/IL6/IL4/	6
6/108	83/18723	8.43E-06	4.98E-05	2.10E-05	KDR/BCL2/EDN1	6
4/108	23/18723	8.52E-06	5.02E-05	2.11E-05	IL6/BCL2/STAT	4
4/108	23/18723	8.52E-06	5.02E-05	2.11E-05	IL6/BCL2/STAT	4
11/108	367/18723	8.92E-06	5.25E-05	2.21E-05	TNF/IL4/CCL2/	11
5/108	49/18723	9.06E-06	5.30E-05	2.23E-05	IFNG/MMP9/HSF	5
5/108	49/18723	9.06E-06	5.30E-05	2.23E-05	IL6/RELA/STAT	5
5/108	49/18723	9.06E-06	5.30E-05	2.23E-05	EGFR/AKT1/ERE	5
5/108	49/18723	9.06E-06	5.30E-05	2.23E-05	TNF/MMP9/MMP2	5
9/108	236/18723	9.13E-06	5.33E-05	2.25E-05	TNF/IFNG/IL1E	9
11/108	368/18723	9.15E-06	5.34E-05	2.25E-05	TNF/IFNG/IL1E	11
8/108	179/18723	9.31E-06	5.42E-05	2.28E-05	TNF/BAK1/STAT	8
12/108	443/18723	9.50E-06	5.52E-05	2.33E-05	TNF/IFNG/IL1E	12
10/108	301/18723	9.59E-06	5.56E-05	2.34E-05	TNF/IFNG/IL1E	10

6/108	85/18723	9.68E-06	5.59E-05	2.35E-05	IL1B/IL6/IL4/	6
6/108	85/18723	9.68E-06	5.59E-05	2.35E-05	BCL2/STAT3/TF	6
6/108	85/18723	9.68E-06	5.59E-05	2.35E-05	TNF/IFNG/IL6/	6
7/108	129/18723	9.80E-06	5.65E-05	2.38E-05	STAT3/GATA3/S	7
5/108	50/18723	1.00E-05	5.77E-05	2.43E-05	IL6/BCL2/STAT	5
4/108	24/18723	1.02E-05	5.82E-05	2.45E-05	IL1B/IL6/IL4/	4
4/108	24/18723	1.02E-05	5.82E-05	2.45E-05	IL4/STAT3/NOS	4
4/108	24/18723	1.02E-05	5.82E-05	2.45E-05	HSPA5/TP53/NF	4
4/108	24/18723	1.02E-05	5.82E-05	2.45E-05	TNF/IFNG/IL1E	4
4/108	24/18723	1.02E-05	5.82E-05	2.45E-05	IL4/STAT3/NOS	4
8/108	182/18723	1.05E-05	6.00E-05	2.53E-05	TNF/KDR/VDR/E	8
12/108	448/18723	1.06E-05	6.06E-05	2.55E-05	CXCL10/VDR/BA	12
8/108	183/18723	1.09E-05	6.22E-05	2.62E-05	TNF/IL1B/IL6/	8
5/108	51/18723	1.11E-05	6.25E-05	2.63E-05	NFE2L2/HSF1/H	5
5/108	51/18723	1.11E-05	6.25E-05	2.63E-05	TNF/IL1B/IL6/	5
5/108	51/18723	1.11E-05	6.25E-05	2.63E-05	CXCL8/IL4/EDN	5
6/108	87/18723	1.11E-05	6.25E-05	2.63E-05	IL6/BAK1/BCL2	6
6/108	87/18723	1.11E-05	6.25E-05	2.63E-05	IFNG/IL4/STAT	6
9/108	242/18723	1.12E-05	6.30E-05	2.65E-05	TNF/IFNG/IL1E	9
7/108	132/18723	1.14E-05	6.41E-05	2.70E-05	BCL2/STAT3/TF	7
6/108	88/18723	1.18E-05	6.65E-05	2.80E-05	TNF/IFNG/IL6/	6
4/108	25/18723	1.21E-05	6.76E-05	2.85E-05	TNF/IFNG/IL6/	4
4/108	25/18723	1.21E-05	6.76E-05	2.85E-05	TNF/IFNG/RELA	4
10/108	310/18723	1.24E-05	6.93E-05	2.92E-05	IFNG/IL1B/IL4	10
7/108	134/18723	1.26E-05	7.02E-05	2.96E-05	TNF/IL1B/IL6/	7
6/108	89/18723	1.26E-05	7.04E-05	2.97E-05	TLR4/AKT1/NFE	6
8/108	188/18723	1.33E-05	7.41E-05	3.12E-05	IFNG/IL4/ICAM	8
5/108	53/18723	1.34E-05	7.45E-05	3.14E-05	BCL2/ESR1/ICA	5
6/108	90/18723	1.35E-05	7.48E-05	3.15E-05	TNF/MMP9/MMP2	6
10/108	314/18723	1.38E-05	7.67E-05	3.23E-05	IL1B/PTGS2/FA	10
12/108	460/18723	1.38E-05	7.67E-05	3.23E-05	CXCL10/VDR/BA	12
11/108	385/18723	1.40E-05	7.72E-05	3.25E-05	IFNG/IL1B/BAK	11
4/108	26/18723	1.42E-05	7.84E-05	3.30E-05	TNF/IL6/TLR4/	4
6/108	91/18723	1.43E-05	7.91E-05	3.33E-05	TNF/MMP9/MMP2	6
9/108	250/18723	1.45E-05	7.97E-05	3.36E-05	IL4/HSPA5/STA	9
7/108	137/18723	1.45E-05	7.98E-05	3.36E-05	TNF/CXCL8/EGF	7
10/108	316/18723	1.46E-05	8.02E-05	3.38E-05	IL1B/PTGS2/FA	10
11/108	390/18723	1.57E-05	8.63E-05	3.63E-05	IL1B/PTGS2/FA	11
10/108	319/18723	1.59E-05	8.68E-05	3.66E-05	CXCL10/BAK1/E	10
7/108	139/18723	1.60E-05	8.72E-05	3.67E-05	TNF/IL6/PTGS2	7
5/108	55/18723	1.61E-05	8.78E-05	3.70E-05	NFKB1/NFKBIA/	5
11/108	391/18723	1.61E-05	8.78E-05	3.70E-05	TNF/IL4/CCL2/	11
6/108	93/18723	1.63E-05	8.84E-05	3.72E-05	EGFR/VDR/APRT	6
4/108	27/18723	1.66E-05	9.00E-05	3.79E-05	EGFR/NOS3/NOS	4
4/108	27/18723	1.66E-05	9.00E-05	3.79E-05	IFNG/TP53/IRF	4
6/108	94/18723	1.73E-05	9.35E-05	3.94E-05	TNF/IFNG/IL4/	6
6/108	94/18723	1.73E-05	9.35E-05	3.94E-05	HSPA5/AKT1/PM	6
8/108	195/18723	1.73E-05	9.37E-05	3.95E-05	GPT/PAH/NOS3/	8
9/108	256/18723	1.75E-05	9.43E-05	3.97E-05	IL4/HSPA5/STA	9
5/108	56/18723	1.76E-05	9.46E-05	3.98E-05	IL1B/IL6/IL4/	5
5/108	56/18723	1.76E-05	9.46E-05	3.98E-05	EGFR/ICAM1/EI	5
8/108	196/18723	1.80E-05	9.65E-05	4.07E-05	TNF/KDR/VDR/E	8
8/108	196/18723	1.80E-05	9.65E-05	4.07E-05	AKT1/MMP9/HSF	8
6/108	95/18723	1.84E-05	9.83E-05	4.14E-05	TNF/IFNG/IL1E	6

9/108	258/18723	1.86E-05	9.94E-05	4.19E-05	IL6/BCL2/STAT	9
7/108	143/18723	1.92E-05	0.000102158	4.30E-05	TNF/IL1B/AKT1	7
5/108	57/18723	1.92E-05	0.000102158	4.30E-05	TNF/IL6/NFKB1	5
5/108	57/18723	1.92E-05	0.000102158	4.30E-05	IL1B/INSR/IL1	5
4/108	28/18723	1.93E-05	0.000102158	4.30E-05	TP53/CERS1/VI	4
4/108	28/18723	1.93E-05	0.000102158	4.30E-05	TNF/IL1B/IL6/	4
4/108	28/18723	1.93E-05	0.000102158	4.30E-05	IL1B/IL6/PTGS	4
8/108	198/18723	1.94E-05	0.000102542	4.32E-05	IL6/EGFR/BAK1	8
5/108	58/18723	2.09E-05	0.00011021	4.64E-05	TNF/IFNG/HSF1	5
5/108	58/18723	2.09E-05	0.00011021	4.64E-05	EGFR/CAT/STAT	5
5/108	58/18723	2.09E-05	0.00011021	4.64E-05	TNF/TP53/CASP	5
3/108	10/18723	2.17E-05	0.000114028	4.80E-05	IL1B/ERBB2/II	3
3/108	10/18723	2.17E-05	0.000114028	4.80E-05	IFNG/IL4/TLR4	3
3/108	10/18723	2.17E-05	0.000114028	4.80E-05	TLR4/RELA/MAP	3
3/108	10/18723	2.17E-05	0.000114028	4.80E-05	TLR4/RELA/MAP	3
6/108	98/18723	2.19E-05	0.000114631	4.83E-05	TNF/IFNG/IL1B	6
6/108	98/18723	2.19E-05	0.000114631	4.83E-05	TLR4/AKT1/NFB	6
4/108	29/18723	2.23E-05	0.000116012	4.89E-05	EGFR/ICAM1/CA	4
4/108	29/18723	2.23E-05	0.000116012	4.89E-05	AKT1/NFE2L2/S	4
9/108	264/18723	2.23E-05	0.000116045	4.89E-05	TNF/IFNG/IL1B	9
8/108	202/18723	2.24E-05	0.00011628	4.90E-05	IFNG/IL1B/PTG	8
5/108	59/18723	2.27E-05	0.000118042	4.97E-05	IL1B/HMGCR/CA	5
7/108	147/18723	2.29E-05	0.000118866	5.01E-05	IL6/EGFR/RELA	7
6/108	99/18723	2.32E-05	0.000120121	5.06E-05	TNF/IFNG/IL1B	6
6/108	99/18723	2.32E-05	0.000120121	5.06E-05	IL4/TLR4/CD40	6
12/108	486/18723	2.38E-05	0.000123082	5.18E-05	CXCL10/VDR/BA	12
7/108	148/18723	2.39E-05	0.000123498	5.20E-05	TNF/BAK1/HSF1	7
6/108	100/18723	2.46E-05	0.000126671	5.33E-05	TNF/IFNG/NFKB	6
4/108	30/18723	2.56E-05	0.000131082	5.52E-05	VDR/ESR1/PML/	4
4/108	30/18723	2.56E-05	0.000131082	5.52E-05	IL1B/IL6/RELA	4
4/108	30/18723	2.56E-05	0.000131082	5.52E-05	IL6/EGFR/STAT	4
10/108	338/18723	2.60E-05	0.00013337	5.62E-05	BCL2/CAT/ESR1	10
7/108	150/18723	2.61E-05	0.000133554	5.62E-05	IL6/EGFR/RELA	7
5/108	61/18723	2.68E-05	0.000136724	5.76E-05	EGFR/ICAM1/EI	5
6/108	102/18723	2.75E-05	0.000140223	5.91E-05	IFNG/IL6/IL4/	6
6/108	102/18723	2.75E-05	0.000140223	5.91E-05	TNF/IL1B/AKT1	6
10/108	342/18723	2.88E-05	0.000146416	6.17E-05	IFNG/IL1B/BAK	10
5/108	62/18723	2.90E-05	0.000146417	6.17E-05	BAK1/BCL2/AKT	5
5/108	62/18723	2.90E-05	0.000146417	6.17E-05	TNF/IL1B/IL6/	5
5/108	62/18723	2.90E-05	0.000146417	6.17E-05	IL6/IL4/TLR4/	5
5/108	62/18723	2.90E-05	0.000146417	6.17E-05	IL6/IL4/TLR4/	5
5/108	62/18723	2.90E-05	0.000146417	6.17E-05	TNF/IFNG/IL6/	5
6/108	103/18723	2.91E-05	0.000146417	6.17E-05	PTGS2/CCL2/EI	6
6/108	103/18723	2.91E-05	0.000146417	6.17E-05	IFNG/IL6/TLR4	6
6/108	103/18723	2.91E-05	0.000146417	6.17E-05	TNF/IFNG/IL6/	6
4/108	31/18723	2.92E-05	0.00014663	6.18E-05	IL6/EGFR/STAT	4
4/108	31/18723	2.92E-05	0.00014663	6.18E-05	HSPA5/TP53/HS	4
3/108	11/18723	2.98E-05	0.000148459	6.25E-05	TNF/IFNG/NFKB	3
3/108	11/18723	2.98E-05	0.000148459	6.25E-05	TNF/IL1B/PTGS	3
3/108	11/18723	2.98E-05	0.000148459	6.25E-05	IL1B/ERBB2/II	3
3/108	11/18723	2.98E-05	0.000148459	6.25E-05	HSD17B6/SPP1/	3
3/108	11/18723	2.98E-05	0.000148459	6.25E-05	TNF/IFNG/HMGC	3
10/108	344/18723	3.02E-05	0.000150587	6.34E-05	TNF/IL4/CCL2/	10
6/108	104/18723	3.07E-05	0.000152816	6.44E-05	TNF/IFNG/EGFR	6

5/108	63/18723	3.13E-05	0.000155621	6.55E-05	IL6/MMP9/MMP2	5
8/108	212/18723	3.16E-05	0.000156721	6.60E-05	IFNG/IL1B/AKT	8
5/108	64/18723	3.38E-05	0.000167155	7.04E-05	IL4/TLR4/BCL2	5
5/108	64/18723	3.38E-05	0.000167155	7.04E-05	TNF/IFNG/IL4/	5
5/108	64/18723	3.38E-05	0.000167155	7.04E-05	PTGS2/CAT/EDN	5
6/108	106/18723	3.42E-05	0.000168204	7.08E-05	IFNG/INSR/NOS	6
6/108	106/18723	3.42E-05	0.000168204	7.08E-05	IFNG/IL1B/PTG	6
6/108	106/18723	3.42E-05	0.000168204	7.08E-05	TNF/IFNG/EGFR	6
6/108	106/18723	3.42E-05	0.000168204	7.08E-05	TP53/CERS1/HS	6
7/108	157/18723	3.50E-05	0.000171496	7.22E-05	TNF/IFNG/IL4/	7
7/108	157/18723	3.50E-05	0.000171496	7.22E-05	TNF/IFNG/IL4/	7
10/108	351/18723	3.59E-05	0.000175582	7.39E-05	TNF/RAF1/PTGS	10
6/108	107/18723	3.61E-05	0.000175987	7.41E-05	TNF/IL1B/IL6/	6
6/108	107/18723	3.61E-05	0.000175987	7.41E-05	AKT1/NFKB1/IN	6
6/108	107/18723	3.61E-05	0.000175987	7.41E-05	IFNG/IL6/TLR4	6
11/108	427/18723	3.62E-05	0.000176193	7.42E-05	TNF/CXCL8/KDR	11
5/108	65/18723	3.65E-05	0.000176542	7.44E-05	IL1B/IL6/IL4/	5
5/108	65/18723	3.65E-05	0.000176542	7.44E-05	HSPA5/AKT1/PM	5
5/108	65/18723	3.65E-05	0.000176542	7.44E-05	AKT1/INSR/IGF	5
5/108	65/18723	3.65E-05	0.000176542	7.44E-05	TNF/IL4/CXCL1	5
5/108	65/18723	3.65E-05	0.000176542	7.44E-05	TNF/CXCL10/ES	5
10/108	353/18723	3.76E-05	0.000181253	7.63E-05	CXCL10/BAK1/B	10
4/108	33/18723	3.77E-05	0.000181253	7.63E-05	THBD/TP53/GAT	4
4/108	33/18723	3.77E-05	0.000181253	7.63E-05	HSPA5/FASN/GA	4
4/108	33/18723	3.77E-05	0.000181253	7.63E-05	TP53/CERS1/VD	4
9/108	283/18723	3.84E-05	0.00018439	7.77E-05	GPT/STAT3/TP5	9
9/108	283/18723	3.84E-05	0.00018439	7.77E-05	IL4/CXCL10/BC	9
5/108	66/18723	3.93E-05	0.000187644	7.90E-05	IL6/IL4/STAT3	5
5/108	66/18723	3.93E-05	0.000187644	7.90E-05	CXCL10/CCL2/C	5
5/108	66/18723	3.93E-05	0.000187644	7.90E-05	TNF/IL6/AKT1/	5
9/108	284/18723	3.95E-05	0.000187644	7.90E-05	SOAT1/SPP1/NF	9
3/108	12/18723	3.95E-05	0.000187644	7.90E-05	IL1B/ERBB2/IL	3
3/108	12/18723	3.95E-05	0.000187644	7.90E-05	CAV1/MAPK1/MA	3
3/108	12/18723	3.95E-05	0.000187644	7.90E-05	CCL2/ICAM1/FA	3
3/108	12/18723	3.95E-05	0.000187644	7.90E-05	CERS1/VDAC1/S	3
3/108	12/18723	3.95E-05	0.000187644	7.90E-05	IL6/EGFR/TP53	3
10/108	357/18723	4.14E-05	0.000195938	8.25E-05	TNF/IFNG/IL6/	10
10/108	357/18723	4.14E-05	0.000195938	8.25E-05	IFNG/CXCL10/C	10
11/108	434/18723	4.19E-05	0.000198281	8.35E-05	KDR/BAK1/BCL2	11
6/108	110/18723	4.22E-05	0.00019896	8.38E-05	TNF/IFNG/IL6/	6
6/108	110/18723	4.22E-05	0.00019896	8.38E-05	TNF/IFNG/IL6/	6
4/108	34/18723	4.25E-05	0.000199648	8.41E-05	APRT/MTOR/MAP	4
4/108	34/18723	4.25E-05	0.000199648	8.41E-05	TP53/AKT1/MTO	4
4/108	34/18723	4.25E-05	0.000199648	8.41E-05	BCL2/AKT1/CAV	4
5/108	68/18723	4.54E-05	0.000212375	8.94E-05	TNF/IFNG/IL6/	5
5/108	68/18723	4.54E-05	0.000212375	8.94E-05	HSPA5/EGFR/ES	5
5/108	68/18723	4.54E-05	0.000212375	8.94E-05	PTGS2/BCL2/AK	5
5/108	68/18723	4.54E-05	0.000212375	8.94E-05	TP53/CERS1/VD	5
6/108	112/18723	4.67E-05	0.000217985	9.18E-05	TNF/IL1B/IL6/	6
4/108	35/18723	4.78E-05	0.000222186	9.36E-05	NOS3/NFE2L2/N	4
4/108	35/18723	4.78E-05	0.000222186	9.36E-05	IL6/EGFR/HMOX	4
4/108	35/18723	4.78E-05	0.000222186	9.36E-05	BAK1/NFE2L2/C	4
8/108	225/18723	4.82E-05	0.000223975	9.43E-05	TNF/IFNG/IL1B	8
5/108	69/18723	4.88E-05	0.000226233	9.53E-05	SOAT1/NFKBIA/	5

6/108	113/18723	4.91E-05	0.000227442	9.58E-05	TNF/IL1B/IL6/	6
7/108	166/18723	4.99E-05	0.000231076	9.73E-05	EGFR/BCL2/AKT	7
3/108	13/18723	5.12E-05	0.000235472	9.92E-05	HSPA5/TP53/GS	3
3/108	13/18723	5.12E-05	0.000235472	9.92E-05	EGFR/ASS1/CPS	3
3/108	13/18723	5.12E-05	0.000235472	9.92E-05	IL6/IL4/GATA3	3
3/108	13/18723	5.12E-05	0.000235472	9.92E-05	IFNG/NOS2/BCL	3
3/108	13/18723	5.12E-05	0.000235472	9.92E-05	IL6/IL4/GATA3	3
6/108	114/18723	5.16E-05	0.000236915	9.98E-05	TNF/IFNG/IL6/	6
10/108	368/18723	5.34E-05	0.000244908	0.00010315	IFNG/IL4/FASN	10
4/108	36/18723	5.35E-05	0.000244908	0.00010315	HSPA5/FASN/GA	4
4/108	36/18723	5.35E-05	0.000244908	0.00010315	EGFR/AKT1/NFE	4
7/108	168/18723	5.39E-05	0.000245978	0.0001036	TNF/IL1B/IL6/	7
7/108	168/18723	5.39E-05	0.000245978	0.0001036	TNF/IL1B/IL6/	7
8/108	229/18723	5.46E-05	0.000248979	0.00010486	TNF/IL6/FASN/	8
6/108	116/18723	5.68E-05	0.000258931	0.00010905	BCL2/AKT1/MTO	6
9/108	298/18723	5.73E-05	0.000260712	0.0001098	TNF/IFNG/IL1E	9
6/108	117/18723	5.96E-05	0.000270086	0.00011375	SOAT1/NFKB1/N	6
4/108	37/18723	5.97E-05	0.000270086	0.00011375	IL1B/IL6/IL4/	4
4/108	37/18723	5.97E-05	0.000270086	0.00011375	IL1B/IL6/IL4/	4
4/108	37/18723	5.97E-05	0.000270086	0.00011375	BCL2/AKT1/CAS	4
4/108	37/18723	5.97E-05	0.000270086	0.00011375	IL6/BCL2/STAT	4
5/108	72/18723	5.99E-05	0.000270462	0.00011391	AKT1/INSR/IGF	5
5/108	73/18723	6.40E-05	0.000287641	0.00012114	AKT1/INSR/IGF	5
5/108	73/18723	6.40E-05	0.000287641	0.00012114	TNF/IFNG/IL6/	5
5/108	73/18723	6.40E-05	0.000287641	0.00012114	KDR/RELA/VCAM	5
5/108	73/18723	6.40E-05	0.000287641	0.00012114	AKT1/INSR/IGF	5
7/108	173/18723	6.48E-05	0.000290599	0.00012239	TNF/IFNG/CCL2	7
3/108	14/18723	6.49E-05	0.000290599	0.00012239	TNF/IL1B/PTGS	3
3/108	14/18723	6.49E-05	0.000290599	0.00012239	HSPA5/BAK1/PT	3
4/108	38/18723	6.64E-05	0.000296948	0.00012506	EGFR/ESR2/ESF	4
4/108	38/18723	6.64E-05	0.000296948	0.00012506	IFNG/IL4/STAT	4
6/108	120/18723	6.87E-05	0.000306552	0.00012911	STAT3/GATA3/S	6
8/108	237/18723	6.95E-05	0.00030968	0.00013043	IFNG/IL1B/AKT	8
6/108	121/18723	7.19E-05	0.000319908	0.00013473	EGFR/HAP1/NCF	6
6/108	121/18723	7.19E-05	0.000319908	0.00013473	IL1B/NOS3/EDN	6
4/108	39/18723	7.37E-05	0.000327472	0.00013792	AKT1/NFE2L2/I	4
5/108	76/18723	7.76E-05	0.000343857	0.00014482	TNF/CCL2/STAT	5
5/108	76/18723	7.76E-05	0.000343857	0.00014482	IL6/CAT/HSF1/	5
10/108	385/18723	7.78E-05	0.000343857	0.00014482	IFNG/IL4/FASN	10
10/108	385/18723	7.78E-05	0.000343857	0.00014482	TNF/PTGS2/CCL	10
3/108	15/18723	8.07E-05	0.000355363	0.00014967	IL1B/NOS2/EDN	3
3/108	15/18723	8.07E-05	0.000355363	0.00014967	IFNG/IL4/TLR4	3
3/108	15/18723	8.07E-05	0.000355363	0.00014967	AKT1/INSR/IGF	3
3/108	15/18723	8.07E-05	0.000355363	0.00014967	TLR4/IL1A/HMO	3
4/108	40/18723	8.15E-05	0.000358012	0.00015078	CXCL10/HAP1/C	4
4/108	40/18723	8.15E-05	0.000358012	0.00015078	TNF/TP53/FADD	4
5/108	77/18723	8.26E-05	0.000362401	0.00015263	IL6/BAK1/AKT1	5
7/108	180/18723	8.32E-05	0.000364091	0.00015334	TNF/IL1B/IL6/	7
7/108	180/18723	8.32E-05	0.000364091	0.00015334	IL1B/TLR4/AKT	7
9/108	313/18723	8.35E-05	0.000364845	0.00015366	IL1B/EGFR/AKT	9
7/108	181/18723	8.62E-05	0.000374802	0.00015785	IFNG/IL6/IL4/	7
7/108	181/18723	8.62E-05	0.000374802	0.00015785	IFNG/IL6/IL4/	7
6/108	125/18723	8.62E-05	0.000374802	0.00015785	BCL2/AKT1/MTO	6
6/108	125/18723	8.62E-05	0.000374802	0.00015785	TNF/IFNG/EGFF	6

5/108	78/18723	8.79E-05	0.000380569	0.00016028	TNF/BAK1/F3/I	5
5/108	78/18723	8.79E-05	0.000380569	0.00016028	NOS3/GLS/NOS2	5
5/108	78/18723	8.79E-05	0.000380569	0.00016028	AKT1/INSR/IGF	5
5/108	78/18723	8.79E-05	0.000380569	0.00016028	TNF/RAF1/PTGS	5
4/108	41/18723	9.00E-05	0.000388119	0.00016346	GPT/AKT1/INSR	4
4/108	41/18723	9.00E-05	0.000388119	0.00016346	IFNG/PTGS2/MA	4
4/108	41/18723	9.00E-05	0.000388119	0.00016346	MAPK14/EDN1/M	4
6/108	126/18723	9.01E-05	0.000388119	0.00016346	TNF/TP53/PML/	6
9/108	317/18723	9.20E-05	0.000395903	0.00016674	TNF/CXCL8/CCL	9
6/108	127/18723	9.41E-05	0.000404546	0.00017038	TNF/CXCL8/EGF	6
8/108	249/18723	9.80E-05	0.000419874	0.00017684	TNF/IL1B/TLR4	8
10/108	396/18723	9.81E-05	0.000419874	0.00017684	IFNG/IL4/FASN	10
6/108	128/18723	9.82E-05	0.000419874	0.00017684	TNF/IFNG/IL6/	6
6/108	128/18723	9.82E-05	0.000419874	0.00017684	TNF/IFNG/IL6/	6
3/108	16/18723	9.90E-05	0.000419874	0.00017684	IL6/IL4/GATA3	3
3/108	16/18723	9.90E-05	0.000419874	0.00017684	MTOR/MAPK1/CF	3
3/108	16/18723	9.90E-05	0.000419874	0.00017684	STAT3/TP53/HS	3
3/108	16/18723	9.90E-05	0.000419874	0.00017684	IFNG/IL4/TLR4	3
3/108	16/18723	9.90E-05	0.000419874	0.00017684	TLR4/RELA/NFK	3
3/108	16/18723	9.90E-05	0.000419874	0.00017684	NFKB1/SOD1/AF	3
4/108	42/18723	9.90E-05	0.000419874	0.00017684	SPP1/HSF1/EDN	4
4/108	42/18723	9.90E-05	0.000419874	0.00017684	HSPA5/AKT1/PM	4
5/108	80/18723	9.92E-05	0.00042022	0.00017698	BAK1/BCL2/GAT	5
7/108	186/18723	0.000102165	0.000431415	0.0001817	PTGS2/NOS3/NO	7
7/108	186/18723	0.000102165	0.000431415	0.0001817	IFNG/IL6/IL4/	7
7/108	186/18723	0.000102165	0.000431415	0.0001817	IFNG/IL6/IL4/	7
6/108	129/18723	0.000102561	0.000432602	0.0001822	TNF/BAK1/HSF1	6
5/108	81/18723	0.000105222	0.000442828	0.0001865	TP53/CERS1/VI	5
5/108	81/18723	0.000105222	0.000442828	0.0001865	TP53/CERS1/VI	5
6/108	130/18723	0.000107025	0.000449914	0.00018949	SOAT1/NFKB1/N	6
4/108	43/18723	0.000108655	0.000455234	0.00019173	TNF/IFNG/IL1B	4
4/108	43/18723	0.000108655	0.000455234	0.00019173	TP53/MAPK8/GS	4
4/108	43/18723	0.000108655	0.000455234	0.00019173	IFNG/PTGS2/MA	4
6/108	131/18723	0.000111642	0.000467231	0.00019678	HSP90AA1/PML/	6
8/108	254/18723	0.000112442	0.000470051	0.00019797	IL4/STAT3/NOS	8
8/108	256/18723	0.000118698	0.000495102	0.00020852	BAK1/BCL2/STA	8
8/108	256/18723	0.000118698	0.000495102	0.00020852	IL4/STAT3/NOS	8
4/108	44/18723	0.000118992	0.000495228	0.00020857	TNF/IL6/IL4/I	4
4/108	44/18723	0.000118992	0.000495228	0.00020857	RAF1/MMP2/MAF	4
3/108	17/18723	0.000119667	0.000496387	0.00020906	AKT1/INSR/IGF	3
3/108	17/18723	0.000119667	0.000496387	0.00020906	HSF1/MTOR/GSK	3
3/108	17/18723	0.000119667	0.000496387	0.00020906	MMP9/GATA3/ST	3
7/108	191/18723	0.000120517	0.00049936	0.00021031	IL4/STAT3/NOS	7
6/108	133/18723	0.000121351	0.000502262	0.00021154	TNF/IL1B/TLR4	6
5/108	84/18723	0.00012506	0.000515907	0.00021728	AKT1/INSR/IGF	5
5/108	84/18723	0.00012506	0.000515907	0.00021728	TNF/PTGS2/EGF	5
5/108	84/18723	0.00012506	0.000515907	0.00021728	IL6/CXCL10/NF	5
8/108	258/18723	0.000125239	0.00051608	0.00021735	IL6/RAF1/STAT	8
11/108	491/18723	0.000125951	0.000518443	0.00021835	IFNG/CXCL10/C	11
8/108	259/18723	0.00012862	0.000528851	0.00022273	IL6/RAF1/STAT	8
4/108	45/18723	0.000130024	0.00053287	0.00022443	MAPK14/EDN1/M	4
4/108	45/18723	0.000130024	0.00053287	0.00022443	TP53/MAPK8/GS	4
4/108	45/18723	0.000130024	0.00053287	0.00022443	IFNG/CCL2/NCF	4
5/108	85/18723	0.000132271	0.000541491	0.00022806	MMP9/HSF1/CAV	5

7/108	195/18723	0.000137054	0.000559667	0.00023571	TNF/IFNG/IL1E	7
6/108	136/18723	0.000137159	0.000559667	0.00023571	TNF/IL1B/CXCI	6
6/108	136/18723	0.000137159	0.000559667	0.00023571	TNF/IL6/ERBB2	6
8/108	262/18723	0.000139219	0.000567456	0.00023899	BCL2/STAT3/TF	8
5/108	86/18723	0.000139797	0.000569195	0.00023973	TNF/RAF1/PTGS	5
7/108	196/18723	0.000141463	0.000571689	0.00024078	IL4/AKT1/ERBE	7
7/108	196/18723	0.000141463	0.000571689	0.00024078	TNF/IFNG/IL1E	7
4/108	46/18723	0.000141778	0.000571689	0.00024078	AKT1/NFE2L2/I	4
4/108	46/18723	0.000141778	0.000571689	0.00024078	IL6/BAK1/AKT1	4
4/108	46/18723	0.000141778	0.000571689	0.00024078	IL4/CD40LG/AK	4
4/108	46/18723	0.000141778	0.000571689	0.00024078	MTOR/MAPK1/MA	4
4/108	46/18723	0.000141778	0.000571689	0.00024078	TNF/IL1B/FASN	4
6/108	137/18723	0.000142776	0.000571689	0.00024078	HSPA5/BAK1/NF	6
3/108	18/18723	0.000142998	0.000571689	0.00024078	IL6/STAT3/MTC	3
3/108	18/18723	0.000142998	0.000571689	0.00024078	TNF/IL4/VCAM1	3
3/108	18/18723	0.000142998	0.000571689	0.00024078	IL1B/NOS2/EDN	3
3/108	18/18723	0.000142998	0.000571689	0.00024078	HSPA5/NFE2L2/	3
3/108	18/18723	0.000142998	0.000571689	0.00024078	APRT/MTOR/CPS	3
3/108	18/18723	0.000142998	0.000571689	0.00024078	BCL2/HMGCR/IC	3
3/108	18/18723	0.000142998	0.000571689	0.00024078	TNF/IL1B/IL6	3
3/108	18/18723	0.000142998	0.000571689	0.00024078	HSPA5/HSF1/EI	3
3/108	18/18723	0.000142998	0.000571689	0.00024078	TLR4/MMP3/SOI	3
5/108	87/18723	0.000147646	0.000589642	0.00024834	GPT/PAH/NOS3/	5
7/108	198/18723	0.000150627	0.000600909	0.00025308	HSPA5/STAT3/T	7
4/108	47/18723	0.000154282	0.000613532	0.0002584	TNF/IFNG/IL6/	4
4/108	47/18723	0.000154282	0.000613532	0.0002584	VDR/APRT/CAV1	4
4/108	47/18723	0.000154282	0.000613532	0.0002584	TNF/TP53/FADI	4
5/108	88/18723	0.000155828	0.000618371	0.00026044	IL6/TLR4/F2/N	5
5/108	88/18723	0.000155828	0.000618371	0.00026044	CXCL8/CXCL10/	5
7/108	200/18723	0.000160268	0.000634646	0.00026729	TNF/IFNG/IL1E	7
7/108	200/18723	0.000160268	0.000634646	0.00026729	IL4/STAT3/NOS	7
6/108	140/18723	0.000160729	0.000635132	0.0002675	NFKB1/HMGCR/C	6
6/108	140/18723	0.000160729	0.000635132	0.0002675	TNF/IL1B/IL6/	6
8/108	268/18723	0.000162585	0.000641792	0.0002703	IL6/PTGS2/TLF	8
6/108	141/18723	0.000167094	0.000657987	0.00027712	BCL2/ESR1/GAT	6
6/108	141/18723	0.000167094	0.000657987	0.00027712	IL4/AKT1/ERBE	6
4/108	48/18723	0.000167564	0.000657987	0.00027712	HSPA5/BCL2/TF	4
4/108	48/18723	0.000167564	0.000657987	0.00027712	TP53/MAPK8/GS	4
4/108	48/18723	0.000167564	0.000657987	0.00027712	TNF/PML/FADD/	4
3/108	19/18723	0.000169099	0.000662627	0.00027908	HSPA5/BAK1/PT	3
3/108	19/18723	0.000169099	0.000662627	0.00027908	IL4/CXCL10/MA	3
7/108	202/18723	0.000170402	0.000666342	0.00028064	GPT/AKT1/NFKE	7
7/108	202/18723	0.000170402	0.000666342	0.00028064	TNF/IFNG/IL1E	7
6/108	142/18723	0.000173657	0.000677661	0.00028541	BCL2/ESR1/GAT	6
6/108	142/18723	0.000173657	0.000677661	0.00028541	CCL2/EGFR/BCI	6
7/108	203/18723	0.00017566	0.000684764	0.0002884	CXCL8/EGFR/IN	7
8/108	272/18723	0.000179889	0.000700524	0.00029504	TNF/IFNG/IL1E	8
4/108	49/18723	0.000181653	0.000702292	0.00029578	TP53/AKT1/BCI	4
4/108	49/18723	0.000181653	0.000702292	0.00029578	ICAM1/SOD1/AS	4
4/108	49/18723	0.000181653	0.000702292	0.00029578	IFNG/MMP9/STA	4
4/108	49/18723	0.000181653	0.000702292	0.00029578	NFKBIA/CAV1/M	4
4/108	49/18723	0.000181653	0.000702292	0.00029578	IFNG/IL4/AKT1	4
4/108	49/18723	0.000181653	0.000702292	0.00029578	TNF/IL6/IL4/F	4
4/108	49/18723	0.000181653	0.000702292	0.00029578	MTOR/MAPK1/MA	4

6/108	144/18723	0.000187395	0.000723745	0.00030482	PTGS2/INSR/HM	6
7/108	206/18723	0.000192228	0.000741648	0.00031236	STAT3/TP53/ES	7
9/108	350/18723	0.000193727	0.000746664	0.00031447	TNF/IL1B/IL6/	9
6/108	145/18723	0.000194578	0.000748408	0.0003152	CCL2/BCL2/HMC	6
6/108	145/18723	0.000194578	0.000748408	0.0003152	TNF/IL1B/IL6/	6
4/108	50/18723	0.000196577	0.00075378	0.00031747	TP53/NFE2L2/I	4
4/108	50/18723	0.000196577	0.00075378	0.00031747	BAK1/TP53/AKT	4
4/108	50/18723	0.000196577	0.00075378	0.00031747	NFKB1/SOD1/AF	4
3/108	20/18723	0.000198106	0.000755783	0.00031831	NOS3/NOS2/ASS	3
3/108	20/18723	0.000198106	0.000755783	0.00031831	RELA/NFKBIA/M	3
3/108	20/18723	0.000198106	0.000755783	0.00031831	BCL2/HMGCR/IC	3
3/108	20/18723	0.000198106	0.000755783	0.00031831	BCL2/HMGCR/IC	3
3/108	20/18723	0.000198106	0.000755783	0.00031831	KDR/BCL2/PARF	3
6/108	146/18723	0.000201977	0.00076932	0.00032401	IFNG/IL4/ERBE	6
5/108	93/18723	0.000202064	0.00076932	0.00032401	IL1B/PTGS2/AK	5
8/108	277/18723	0.000203625	0.000774476	0.00032618	IFNG/IL4/STAT	8
9/108	353/18723	0.000206412	0.000784282	0.00033031	IL4/SOAT1/CAT	9
5/108	94/18723	0.000212446	0.000806392	0.00033962	AKT1/CAV1/MAF	5
9/108	355/18723	0.000215247	0.0008162	0.00034375	HSPA5/STAT3/T	9
7/108	211/18723	0.000222634	0.000842508	0.00035483	IL4/SELE/INSF	7
7/108	211/18723	0.000222634	0.000842508	0.00035483	IL1B/PTGS2/HS	7
4/108	52/18723	0.000229047	0.000864868	0.00036425	TP53/NFE2L2/I	4
4/108	52/18723	0.000229047	0.000864868	0.00036425	IL4/SELE/INSF	4
3/108	21/18723	0.000230155	0.000864868	0.00036425	TNF/TLR4/AKT1	3
3/108	21/18723	0.000230155	0.000864868	0.00036425	IL6/STAT3/MTC	3
3/108	21/18723	0.000230155	0.000864868	0.00036425	PTGS2/NFE2L2/	3
3/108	21/18723	0.000230155	0.000864868	0.00036425	MAPK8/GSK3B/S	3
3/108	21/18723	0.000230155	0.000864868	0.00036425	BCL2/HMGCR/IC	3
5/108	96/18723	0.000234423	0.000877391	0.00036953	HSPA5/BAK1/NF	5
5/108	96/18723	0.000234423	0.000877391	0.00036953	SOAT1/HMGCR/C	5
5/108	96/18723	0.000234423	0.000877391	0.00036953	GLS/SDHB/SDHA	5
5/108	96/18723	0.000234423	0.000877391	0.00036953	AKT1/INSR/IGF	5
8/108	284/18723	0.000241128	0.000901587	0.00037972	GPT/PAH/NOS3/	8
6/108	151/18723	0.000242371	0.000904433	0.00038092	HSPA5/BCL2/HA	6
6/108	151/18723	0.000242371	0.000904433	0.00038092	IFNG/CXCL10/C	6
5/108	97/18723	0.000246039	0.000914487	0.00038515	TLR4/BCL2/PLA	5
5/108	97/18723	0.000246039	0.000914487	0.00038515	SOAT1/HMGCR/C	5
5/108	97/18723	0.000246039	0.000914487	0.00038515	CXCL8/CXCL10/	5
5/108	97/18723	0.000246039	0.000914487	0.00038515	CXCL8/CXCL10/	5
4/108	53/18723	0.000246651	0.000914949	0.00038534	SOAT1/HMOX1/A	4
4/108	53/18723	0.000246651	0.000914949	0.00038534	HSPA5/AHR/ASS	4
6/108	152/18723	0.000251162	0.000930764	0.000392	IL1B/TP53/GAT	6
5/108	98/18723	0.000258089	0.000955488	0.00040242	IL6/RELA/NFE2	5
4/108	54/18723	0.000265208	0.000975744	0.00041095	TP53/NFE2L2/I	4
4/108	54/18723	0.000265208	0.000975744	0.00041095	ESR2/ESR1/AR/	4
3/108	22/18723	0.00026538	0.000975744	0.00041095	SOD1/APOE/APC	3
3/108	22/18723	0.00026538	0.000975744	0.00041095	TNF/KDR/SERPI	3
3/108	22/18723	0.00026538	0.000975744	0.00041095	SOD1/APOE/APC	3
3/108	22/18723	0.00026538	0.000975744	0.00041095	HSPA5/NFE2L2/	3
3/108	22/18723	0.00026538	0.000975744	0.00041095	RELA/TP53/NFE	3
5/108	99/18723	0.000270581	0.000993895	0.00041859	TNF/RELA/TP53	5
7/108	218/18723	0.00027161	0.000995727	0.00041937	HSD17B6/SPP1/	7
7/108	218/18723	0.00027161	0.000995727	0.00041937	TLR4/NCF1/IRF	7
9/108	367/18723	0.000275132	0.001007653	0.00042439	TP53/AKT1/GAT	9

6/108	155/18723	0.000279053	0.001021017	0.00043002	AKT1/NFE2L2/I	6
5/108	100/18723	0.000283527	0.001035372	0.00043606	CAT/CAV1/APOI	5
5/108	100/18723	0.000283527	0.001035372	0.00043606	IL4/SELE/INSI	5
4/108	55/18723	0.000284747	0.001037805	0.00043709	GPT/AKT1/INSI	4
4/108	55/18723	0.000284747	0.001037805	0.00043709	TNF/EDN1/MTOI	4
6/108	156/18723	0.000288871	0.001051816	0.00044299	EGFR/VCAM1/EI	6
8/108	293/18723	0.000297493	0.00108216	0.00045577	BCL2/CAT/MMP(	8
6/108	157/18723	0.000298959	0.00108644	0.00045757	IL4/TLR4/PRKA	6
3/108	23/18723	0.000303909	0.001098049	0.00046246	TNF/IL6/NFKBI	3
3/108	23/18723	0.000303909	0.001098049	0.00046246	NOS3/NFE2L2/S	3
3/108	23/18723	0.000303909	0.001098049	0.00046246	KDR/BCL2/PARI	3
3/108	23/18723	0.000303909	0.001098049	0.00046246	IL1B/CXCL8/CA	3
3/108	23/18723	0.000303909	0.001098049	0.00046246	PTGS2/MAPK14/	3
3/108	23/18723	0.000303909	0.001098049	0.00046246	IL4/STAT3/PAI	3
4/108	56/18723	0.000305297	0.001099891	0.00046324	TP53/NFE2L2/I	4
4/108	56/18723	0.000305297	0.001099891	0.00046324	IFNG/MMP9/GAT	4
4/108	56/18723	0.000305297	0.001099891	0.00046324	IFNG/STAT3/TI	4
7/108	223/18723	0.000311633	0.001121641	0.0004724	TNF/IFNG/IL6/	7
6/108	159/18723	0.000319964	0.001148321	0.00048363	HSP90AA1/PML/	6
6/108	159/18723	0.000319964	0.001148321	0.00048363	HSPA5/BAK1/NI	6
6/108	159/18723	0.000319964	0.001148321	0.00048363	CXCL10/HAP1/(	6
5/108	103/18723	0.0003252	0.001165998	0.00049108	CXCL8/CXCL10/	5
4/108	57/18723	0.000326891	0.0011676	0.00049175	HMGCR/SOD1/AI	4
4/108	57/18723	0.000326891	0.0011676	0.00049175	TNF/IFNG/IL1I	4
4/108	57/18723	0.000326891	0.0011676	0.00049175	BCL2/MAPK14/I	4
4/108	57/18723	0.000326891	0.0011676	0.00049175	HMGCR/SOD1/AI	4
7/108	225/18723	0.000328912	0.001173703	0.00049432	BAK1/PLAT/F3/	7
5/108	104/18723	0.000340072	0.001212374	0.00051061	IL6/MMP9/MMP2	5
3/108	24/18723	0.00034587	0.001226059	0.00051637	RAF1/MAPK1/MA	3
3/108	24/18723	0.00034587	0.001226059	0.00051637	EGFR/AKT1/SOI	3
3/108	24/18723	0.00034587	0.001226059	0.00051637	RAF1/HAP1/CAS	3
3/108	24/18723	0.00034587	0.001226059	0.00051637	BCL2/HMGCR/I(	3
3/108	24/18723	0.00034587	0.001226059	0.00051637	TNF/AKT1/APOI	3
3/108	24/18723	0.00034587	0.001226059	0.00051637	IFNG/GATA3/PA	3
4/108	58/18723	0.000349556	0.001235626	0.0005204	VCAM1/PARP1/A	4
4/108	58/18723	0.000349556	0.001235626	0.0005204	IL1B/IL6/PTGS	4
4/108	58/18723	0.000349556	0.001235626	0.0005204	TLR4/IRF1/HSI	4
6/108	162/18723	0.000353623	0.001248825	0.00052596	IL6/TLR4/FADI	6
8/108	302/18723	0.00036421	0.001285005	0.0005412	BCL2/CAT/MMP(	8
6/108	163/18723	0.000365438	0.001288113	0.00054251	IL1B/PTGS2/FA	6
7/108	229/18723	0.000365777	0.001288113	0.00054251	TNF/IL6/PTGS2	7
5/108	106/18723	0.000371355	0.001304085	0.00054923	IFNG/STAT3/IN	5
5/108	106/18723	0.000371355	0.001304085	0.00054923	TNF/IFNG/NFKI	5
5/108	106/18723	0.000371355	0.001304085	0.00054923	TNF/IFNG/IL6/	5
8/108	303/18723	0.000372321	0.001306256	0.00055015	IL4/AKT1/ERBI	8
4/108	59/18723	0.000373325	0.001308555	0.00055112	IL1B/KDR/MAPI	4
7/108	230/18723	0.000375492	0.001314924	0.0005538	IFNG/IL4/CAT/	7
10/108	468/18723	0.000376422	0.001316951	0.00055465	TLR4/RELA/BCI	10
9/108	384/18723	0.000383106	0.001339086	0.00056398	IL4/CXCL10/B(	9
5/108	107/18723	0.000387789	0.001352938	0.00056981	TNF/PTGS2/EGI	5
5/108	107/18723	0.000387789	0.001352938	0.00056981	TNF/PTGS2/EGI	5
6/108	165/18723	0.000389995	0.00135542	0.00057086	BCL2/ESR1/GAT	6
6/108	165/18723	0.000389995	0.00135542	0.00057086	TNF/IFNG/IL1I	6
3/108	25/18723	0.000391389	0.00135542	0.00057086	BAK1/BCL2/PMI	3

3/108	25/18723	0.000391389	0.00135542	0.00057086	CXCL8/KDR/F3	3
3/108	25/18723	0.000391389	0.00135542	0.00057086	TNF/IL1B/AKT1	3
3/108	25/18723	0.000391389	0.00135542	0.00057086	TLR4/RELA/NFK	3
3/108	25/18723	0.000391389	0.00135542	0.00057086	NOS3/NFE2L2/S	3
3/108	25/18723	0.000391389	0.00135542	0.00057086	NOS3/NFE2L2/S	3
4/108	60/18723	0.000398228	0.001376563	0.00057976	BAK1/PLAT/SEF	4
4/108	60/18723	0.000398228	0.001376563	0.00057976	IFNG/PTGS2/MA	4
6/108	166/18723	0.000402748	0.001389629	0.00058526	CXCL8/SELE/IN	6
6/108	166/18723	0.000402748	0.001389629	0.00058526	BCL2/HMGCR/MA	6
5/108	108/18723	0.000404767	0.001394034	0.00058712	EGFR/HAP1/NCF	5
5/108	108/18723	0.000404767	0.001394034	0.00058712	HSPA5/BCL2/HA	5
8/108	308/18723	0.000415117	0.001428369	0.00060158	TNF/IL1B/IL6/	8
6/108	167/18723	0.000415825	0.001429492	0.00060205	IFNG/CXCL10/C	6
5/108	109/18723	0.000422301	0.001450427	0.00061087	AKT1/MMP9/VDA	5
4/108	61/18723	0.000424295	0.001451963	0.00061152	BCL2/NFE2L2/C	4
4/108	61/18723	0.000424295	0.001451963	0.00061152	BCL2/STAT3/IC	4
4/108	61/18723	0.000424295	0.001451963	0.00061152	TNF/IL1B/AKT1	4
4/108	61/18723	0.000424295	0.001451963	0.00061152	CXCL10/CCL2/A	4
6/108	168/18723	0.000429231	0.001467515	0.00061807	CCL2/EGFR/BCL	6
7/108	236/18723	0.000438216	0.001492752	0.00062869	GPT/PAH/AKT1/	7
7/108	236/18723	0.000438216	0.001492752	0.00062869	IL1B/EGFR/AKT	7
5/108	110/18723	0.000440401	0.001492752	0.00062869	IL1B/INSR/IL1	5
5/108	110/18723	0.000440401	0.001492752	0.00062869	GPT/PAH/NOS3/	5
5/108	110/18723	0.000440401	0.001492752	0.00062869	TLR4/BCL2/PLA	5
3/108	26/18723	0.000440589	0.001492752	0.00062869	NFKBIA/CAV1/A	3
3/108	26/18723	0.000440589	0.001492752	0.00062869	ESR1/GATA3/MA	3
3/108	26/18723	0.000440589	0.001492752	0.00062869	TLR4/RELA/NFK	3
3/108	26/18723	0.000440589	0.001492752	0.00062869	CXCL8/KDR/F3	3
3/108	26/18723	0.000440589	0.001492752	0.00062869	IL1B/CXCL8/CA	3
4/108	62/18723	0.000451558	0.001527161	0.00064319	TNF/TP53/FADD	4
4/108	62/18723	0.000451558	0.001527161	0.00064319	TNF/IFNG/EGFF	4
8/108	314/18723	0.000471675	0.00159376	0.00067124	IL6/PTGS2/TLF	8
5/108	112/18723	0.000478352	0.001613417	0.00067951	TNF/IL1B/TLR4	5
5/108	112/18723	0.000478352	0.001613417	0.00067951	TNF/IL1B/TLR4	5
4/108	63/18723	0.000480047	0.001616231	0.0006807	IFNG/NOS2/FAD	4
4/108	63/18723	0.000480047	0.001616231	0.0006807	IFNG/PTGS2/MA	4
7/108	240/18723	0.000484493	0.001629736	0.00068639	GPT/PAH/AKT1/	7
6/108	172/18723	0.000486266	0.001634237	0.00068828	TLR4/RELA/ESF	6
3/108	27/18723	0.000493589	0.001652928	0.00069615	NFKB1/SOD1/AF	3
3/108	27/18723	0.000493589	0.001652928	0.00069615	SOAT1/APOE/AF	3
3/108	27/18723	0.000493589	0.001652928	0.00069615	IL4/CXCL10/MA	3
3/108	27/18723	0.000493589	0.001652928	0.00069615	CXCL8/IL4/EDN	3
4/108	64/18723	0.000509794	0.001699616	0.00071582	TNF/IL1B/FASN	4
4/108	64/18723	0.000509794	0.001699616	0.00071582	PTGS2/EDN1/CA	4
4/108	64/18723	0.000509794	0.001699616	0.00071582	HMGCR/SOD1/AF	4
4/108	64/18723	0.000509794	0.001699616	0.00071582	IL1B/RELA/IGF	4
4/108	64/18723	0.000509794	0.001699616	0.00071582	TLR4/NCF1/STA	4
6/108	174/18723	0.000516913	0.001721822	0.00072517	IFNG/IL4/ERBE	6
5/108	114/18723	0.000518715	0.001724762	0.00072641	KDR/BCL2/TP53	5
5/108	114/18723	0.000518715	0.001724762	0.00072641	TP53/CERS1/VI	5
6/108	175/18723	0.00053279	0.001769996	0.00074546	HSD17B6/NCF1/	6
7/108	244/18723	0.000534592	0.001774409	0.00074732	CXCL8/IL4/SEI	7
5/108	115/18723	0.000539832	0.001790219	0.00075398	GATA3/EDN1/MA	5
6/108	176/18723	0.000549045	0.001819164	0.00076617	IL4/NFKB1/GAT	6

3/108	28/18723	0.000550507	0.001819194	0.00076618	NOS3/NFE2L2/S	3
3/108	28/18723	0.000550507	0.001819194	0.00076618	NOS3/GLS/NOS2	3
3/108	28/18723	0.000550507	0.001819194	0.00076618	BAK1/TP53/MMP	3
5/108	116/18723	0.000561588	0.00185418	0.00078092	HSPA5/BAK1/NF	5
4/108	66/18723	0.000573187	0.001887495	0.00079495	IL1B/RELA/IGF	4
4/108	66/18723	0.000573187	0.001887495	0.00079495	CXCL8/CXCL10/	4
4/108	66/18723	0.000573187	0.001887495	0.00079495	HSP90AA1/MAPK	4
5/108	117/18723	0.000583997	0.001919725	0.00080852	IL1B/IL6/IL4/	5
5/108	117/18723	0.000583997	0.001919725	0.00080852	IL4/CXCL10/BC	5
5/108	118/18723	0.000607071	0.001992084	0.000839	PTGS2/INSR/MT	5
5/108	118/18723	0.000607071	0.001992084	0.000839	TNF/IL1B/KDR/	5
3/108	29/18723	0.00061146	0.001997755	0.00084138	NOS3/NFE2L2/S	3
3/108	29/18723	0.00061146	0.001997755	0.00084138	FASN/AKT1/GSK	3
3/108	29/18723	0.00061146	0.001997755	0.00084138	IL1B/GATA3/MA	3
3/108	29/18723	0.00061146	0.001997755	0.00084138	TP53/FADD/CAV	3
3/108	29/18723	0.00061146	0.001997755	0.00084138	IL6/IL4/STAT3	3
6/108	180/18723	0.000617956	0.002017224	0.00084958	TP53/CERS1/HS	6
6/108	181/18723	0.000636189	0.002074939	0.00087389	SPP1/AKT1/EDN	6
4/108	68/18723	0.000641987	0.002090216	0.00088033	IFNG/F2/NOS2/	4
4/108	68/18723	0.000641987	0.002090216	0.00088033	PAH/GLS/ASS1/	4
7/108	252/18723	0.000647158	0.002105226	0.00088665	PTGS2/NOS3/ED	7
7/108	253/18723	0.000662459	0.002153137	0.00090683	RAF1/PTGS2/CA	7
3/108	30/18723	0.00067656	0.002187602	0.00092134	AKT1/EDN1/APC	3
3/108	30/18723	0.00067656	0.002187602	0.00092134	NFKB1/SOD1/AP	3
3/108	30/18723	0.00067656	0.002187602	0.00092134	TNF/IFNG/APOE	3
3/108	30/18723	0.00067656	0.002187602	0.00092134	PTGS2/CCL2/EC	3
3/108	30/18723	0.00067656	0.002187602	0.00092134	HSPA5/BAK1/PT	3
3/108	30/18723	0.00067656	0.002187602	0.00092134	STAT3/TP53/PA	3
4/108	69/18723	0.000678494	0.002190082	0.00092239	TNF/PTGS2/CCL	4
4/108	69/18723	0.000678494	0.002190082	0.00092239	TNF/IFNG/EGFR	4
5/108	121/18723	0.000680407	0.002190607	0.00092261	TLR4/ESR1/IRF	5
5/108	121/18723	0.000680407	0.002190607	0.00092261	EGFR/RELA/HMG	5
5/108	121/18723	0.000680407	0.002190607	0.00092261	TP53/PML/MTOR	5
7/108	255/18723	0.000693924	0.002232213	0.00094013	AKT1/NFE2L2/H	7
5/108	122/18723	0.000706267	0.00226997	0.00095603	CXCL8/CXCL10/	5
7/108	256/18723	0.000710095	0.002280324	0.00096039	PTGS2/EGFR/AK	7
4/108	70/18723	0.000716447	0.002298754	0.00096815	IL6/CXCL10/CC	4
3/108	31/18723	0.000745918	0.002375044	0.00100029	IL1B/PTGS2/ED	3
3/108	31/18723	0.000745918	0.002375044	0.00100029	BAK1/BCL2/CAS	3
3/108	31/18723	0.000745918	0.002375044	0.00100029	IL4/HSP90AA1/	3
3/108	31/18723	0.000745918	0.002375044	0.00100029	IL6/IL4/GATA3	3
3/108	31/18723	0.000745918	0.002375044	0.00100029	IL1B/AKT1/NFK	3
3/108	31/18723	0.000745918	0.002375044	0.00100029	IL1B/PTGS2/ED	3
3/108	31/18723	0.000745918	0.002375044	0.00100029	TNF/KDR/SERPI	3
3/108	31/18723	0.000745918	0.002375044	0.00100029	SOAT1/APOE/AP	3
3/108	31/18723	0.000745918	0.002375044	0.00100029	TP53/ESR1/JUN	3
6/108	188/18723	0.000775801	0.0024681	0.00103948	NFE2L2/GATA3/	6
5/108	125/18723	0.000788269	0.00250564	0.00105529	TP53/CERS1/GA	5
4/108	72/18723	0.00079682	0.002526405	0.00106403	TNF/IL1B/TLR4	4
4/108	72/18723	0.00079682	0.002526405	0.00106403	MAPK14/EDN1/M	4
4/108	72/18723	0.00079682	0.002526405	0.00106403	BAK1/TP53/AKT	4
5/108	126/18723	0.000817121	0.002588583	0.00109022	TNF/IL1B/TLR4	5
3/108	32/18723	0.000819643	0.002590018	0.00109082	NFKB1/NFKBIA/	3
3/108	32/18723	0.000819643	0.002590018	0.00109082	MAPK8/GSK3B/S	3

3/108	32/18723	0.000819643	0.002590018	0.00109082	IL1B/AKT1/EDN	3
4/108	73/18723	0.000839304	0.002645463	0.00111418	TNF/IL6/IL4/C	4
4/108	73/18723	0.000839304	0.002645463	0.00111418	EGFR/HAP1/NCF	4
4/108	73/18723	0.000839304	0.002645463	0.00111418	CXCL10/HAP1/C	4
5/108	128/18723	0.000877181	0.002760215	0.00116251	CAT/CAV1/APOE	5
5/108	128/18723	0.000877181	0.002760215	0.00116251	TNF/IL1B/TLR4	5
4/108	74/18723	0.000883361	0.00277501	0.00116874	TNF/GATA3/ICA	4
4/108	74/18723	0.000883361	0.00277501	0.00116874	HSPA5/BAK1/NF	4
3/108	33/18723	0.000897842	0.002813437	0.00118492	STAT3/STAT1/M	3
3/108	33/18723	0.000897842	0.002813437	0.00118492	TNF/VCAM1/SEL	3
3/108	33/18723	0.000897842	0.002813437	0.00118492	GATA3/EDN1/AF	3
5/108	129/18723	0.000908415	0.002844196	0.00119788	CAT/CAV1/APOE	5
5/108	130/18723	0.000940471	0.002942106	0.00123911	EGFR/HSF1/PML	5
4/108	76/18723	0.000976324	0.003039833	0.00128027	PAH/GLS/ASS1/	4
4/108	76/18723	0.000976324	0.003039833	0.00128027	IL6/RELA/F2/M	4
3/108	34/18723	0.000980617	0.003039833	0.00128027	BCL2/VDAC1/AF	3
3/108	34/18723	0.000980617	0.003039833	0.00128027	IFNG/TP53/HMC	3
3/108	34/18723	0.000980617	0.003039833	0.00128027	RELA/FADD/MTC	3
3/108	34/18723	0.000980617	0.003039833	0.00128027	SOAT1/HMOX1/A	3
3/108	34/18723	0.000980617	0.003039833	0.00128027	RELA/FADD/MTC	3
3/108	34/18723	0.000980617	0.003039833	0.00128027	HSP90AA1/MAPK	3
3/108	34/18723	0.000980617	0.003039833	0.00128027	TLR4/AKT1/APC	3
3/108	34/18723	0.000980617	0.003039833	0.00128027	TNF/MTOR/GSK3	3
3/108	34/18723	0.000980617	0.003039833	0.00128027	IL1B/GATA3/MA	3
6/108	198/18723	0.001015029	0.00314391	0.0013241	IL6/IL4/TLR4/	6
4/108	77/18723	0.001025293	0.003173082	0.00133639	IL1B/GATA3/MA	4
5/108	133/18723	0.001041698	0.003221199	0.00135666	IL1B/PTGS2/AK	5
3/108	35/18723	0.001068072	0.003286511	0.00138416	BCL2/VDAC1/AF	3
3/108	35/18723	0.001068072	0.003286511	0.00138416	NFE2L2/HSF1/H	3
3/108	35/18723	0.001068072	0.003286511	0.00138416	IL1B/RELA/PTF	3
3/108	35/18723	0.001068072	0.003286511	0.00138416	PTGS2/KDR/HMC	3
3/108	35/18723	0.001068072	0.003286511	0.00138416	SOD1/APOE/APC	3
3/108	35/18723	0.001068072	0.003286511	0.00138416	TNF/MTOR/GSK3	3
4/108	78/18723	0.001075963	0.003305372	0.00139211	NFKB1/NFKBIA/	4
4/108	78/18723	0.001075963	0.003305372	0.00139211	NFKB1/NFKBIA/	4
4/108	79/18723	0.001128366	0.003455046	0.00145514	CXCL10/HAP1/C	4
4/108	79/18723	0.001128366	0.003455046	0.00145514	MAPK14/EDN1/M	4
4/108	79/18723	0.001128366	0.003455046	0.00145514	CXCL8/CXCL10/	4
4/108	79/18723	0.001128366	0.003455046	0.00145514	EGFR/HAP1/NCF	4
3/108	36/18723	0.001160307	0.003538416	0.00149026	SELE/ICAM1/FA	3
3/108	36/18723	0.001160307	0.003538416	0.00149026	RAF1/HAP1/CAS	3
3/108	36/18723	0.001160307	0.003538416	0.00149026	IL6/IL4/GATA3	3
3/108	36/18723	0.001160307	0.003538416	0.00149026	HAP1/NCF1/MMF	3
3/108	36/18723	0.001160307	0.003538416	0.00149026	TLR4/AKT1/APC	3
4/108	80/18723	0.001182534	0.00360035	0.00151634	RELA/AKT1/ERE	4
4/108	80/18723	0.001182534	0.00360035	0.00151634	PML/MAPK1/MAF	4
5/108	137/18723	0.001188986	0.003617061	0.00152338	HSD17B6/SPP1/	5
9/108	452/18723	0.001220179	0.003708949	0.00156208	IL1B/PTGS2/NC	9
4/108	81/18723	0.001238498	0.003749453	0.00157914	TNF/EGFR/RELA	4
4/108	81/18723	0.001238498	0.003749453	0.00157914	IFNG/STAT3/IN	4
4/108	81/18723	0.001238498	0.003749453	0.00157914	HSP90AA1/MAPK	4
4/108	81/18723	0.001238498	0.003749453	0.00157914	BCL2/MAPK14/E	4
4/108	81/18723	0.001238498	0.003749453	0.00157914	INSR/JUN/BCL2	4
3/108	37/18723	0.001257418	0.0038006	0.00160068	BCL2/HMGCR/IC	3

3/108	37/18723	0.001257418	0.0038006	0.00160068	IL1B/RELA/PTI	3
5/108	139/18723	0.001268157	0.003829976	0.00161305	IL1B/INSR/IL1	5
4/108	82/18723	0.001296291	0.003908654	0.00164619	IL6/IL4/TLR4/	4
4/108	82/18723	0.001296291	0.003908654	0.00164619	IFNG/STAT3/IN	4
4/108	83/18723	0.001355943	0.004066587	0.0017127	IL1B/NOS3/GAT	4
3/108	38/18723	0.001359501	0.004066587	0.0017127	EDN1/MTOR/PAI	3
3/108	38/18723	0.001359501	0.004066587	0.0017127	NFKBIA/CAV1/A	3
3/108	38/18723	0.001359501	0.004066587	0.0017127	NFKBIA/CAV1/A	3
3/108	38/18723	0.001359501	0.004066587	0.0017127	BCL2/VDAC1/AI	3
3/108	38/18723	0.001359501	0.004066587	0.0017127	CCL2/MAPK1/MA	3
3/108	38/18723	0.001359501	0.004066587	0.0017127	EDN1/ASS1/CPS	3
3/108	38/18723	0.001359501	0.004066587	0.0017127	IL6/IL4/STAT3	3
3/108	38/18723	0.001359501	0.004066587	0.0017127	HAP1/NCF1/MMI	3
3/108	38/18723	0.001359501	0.004066587	0.0017127	MAPK8/GSK3B/S	3
4/108	84/18723	0.001417487	0.004219863	0.00177726	TNF/IFNG/MTOI	4
4/108	84/18723	0.001417487	0.004219863	0.00177726	TNF/EGFR/RELA	4
4/108	84/18723	0.001417487	0.004219863	0.00177726	TNF/EGFR/RELA	4
4/108	84/18723	0.001417487	0.004219863	0.00177726	BCL2/APRT/TYI	4
4/108	84/18723	0.001417487	0.004219863	0.00177726	TNF/CXCL10/HM	4
4/108	84/18723	0.001417487	0.004219863	0.00177726	CCL2/BCL2/TP5	4
2/108	10/18723	0.001439371	0.004247956	0.00178909	ASS1/CPS1	2
2/108	10/18723	0.001439371	0.004247956	0.00178909	BCL2/MTOR	2
2/108	10/18723	0.001439371	0.004247956	0.00178909	NOS3/NOS2	2
2/108	10/18723	0.001439371	0.004247956	0.00178909	CASP3/CASP9	2
2/108	10/18723	0.001439371	0.004247956	0.00178909	AKT1/PCNA	2
2/108	10/18723	0.001439371	0.004247956	0.00178909	AKT1/MTOR	2
2/108	10/18723	0.001439371	0.004247956	0.00178909	TP53/ESR1	2
2/108	10/18723	0.001439371	0.004247956	0.00178909	TP53/CERS1	2
2/108	10/18723	0.001439371	0.004247956	0.00178909	BAK1/ESR1	2
2/108	10/18723	0.001439371	0.004247956	0.00178909	MAPK1/MAPK3	2
2/108	10/18723	0.001439371	0.004247956	0.00178909	ESR1/AR	2
6/108	212/18723	0.001440787	0.004248794	0.00178944	SOAT1/AKT1/CY	6
3/108	39/18723	0.00146665	0.004314893	0.00181728	EDN1/MTOR/PAI	3
3/108	39/18723	0.00146665	0.004314893	0.00181728	TLR4/IRF1/HSI	3
3/108	39/18723	0.00146665	0.004314893	0.00181728	BCL2/HMGCR/IC	3
6/108	213/18723	0.001475713	0.004338158	0.00182708	BCL2/GATA3/EI	6
4/108	85/18723	0.001480955	0.004346259	0.00183049	SOAT1/HMOX1/A	4
4/108	85/18723	0.001480955	0.004346259	0.00183049	TNF/EGFR/RELA	4
5/108	144/18723	0.0014831	0.004346259	0.00183049	IL4/TLR4/PRKA	5
5/108	144/18723	0.0014831	0.004346259	0.00183049	IL4/TLR4/PRKA	5
6/108	214/18723	0.001511282	0.004421943	0.00186237	CCL2/EGFR/BCI	6
6/108	214/18723	0.001511282	0.004421943	0.00186237	IL1B/PTGS2/FA	6
4/108	86/18723	0.001546377	0.004514074	0.00190117	BCL2/EDN1/MAI	4
4/108	86/18723	0.001546377	0.004514074	0.00190117	IL1B/AKT1/EDN	4
4/108	86/18723	0.001546377	0.004514074	0.00190117	IL6/EGFR/SPP1	4
3/108	40/18723	0.001578956	0.004587772	0.00193221	EGFR/SELE/ESI	3
3/108	40/18723	0.001578956	0.004587772	0.00193221	RELA/AKT1/SOI	3
3/108	40/18723	0.001578956	0.004587772	0.00193221	AKT1/MTOR/CPS	3
3/108	40/18723	0.001578956	0.004587772	0.00193221	EGFR/JUN/PCNA	3
3/108	40/18723	0.001578956	0.004587772	0.00193221	MAPK8/GSK3B/S	3
3/108	40/18723	0.001578956	0.004587772	0.00193221	TP53/ESR1/JUN	3
7/108	294/18723	0.001582903	0.004595682	0.00193554	BAK1/PLAT/F3/	7
5/108	147/18723	0.0016243	0.004712227	0.00198462	IL1B/HMGCR/EI	5
3/108	41/18723	0.001696509	0.004910327	0.00206806	IL6/IL4/NOS2	3

3/108	41/18723	0.001696509	0.004910327	0.00206806	BCL2/HMGCR/IC	3
3/108	41/18723	0.001696509	0.004910327	0.00206806	IL4/HSP90AA1/	3
8/108	384/18723	0.001723439	0.004981543	0.00209805	BCL2/HMGCR/MA	8
5/108	149/18723	0.001723768	0.004981543	0.00209805	TNF/CXCL10/ST	5
2/108	11/18723	0.001752614	0.00499401	0.0021033	APRT/ATIC	2
2/108	11/18723	0.001752614	0.00499401	0.0021033	NOS3/NOS2	2
2/108	11/18723	0.001752614	0.00499401	0.0021033	INSR/BCL2L1	2
2/108	11/18723	0.001752614	0.00499401	0.0021033	IL1B/EDN1	2
2/108	11/18723	0.001752614	0.00499401	0.0021033	AR/PARP1	2
2/108	11/18723	0.001752614	0.00499401	0.0021033	SOAT1/APOB	2
2/108	11/18723	0.001752614	0.00499401	0.0021033	HSF1/SOD1	2
2/108	11/18723	0.001752614	0.00499401	0.0021033	NFE2L2/HMOX1	2
2/108	11/18723	0.001752614	0.00499401	0.0021033	MTOR/AR	2
2/108	11/18723	0.001752614	0.00499401	0.0021033	ASS1/CPS1	2
2/108	11/18723	0.001752614	0.00499401	0.0021033	MAPK8/GSK3B	2
2/108	11/18723	0.001752614	0.00499401	0.0021033	IL6/HSF1	2
2/108	11/18723	0.001752614	0.00499401	0.0021033	HSPA5/PTPN1	2
2/108	11/18723	0.001752614	0.00499401	0.0021033	KDR/PARP1	2
2/108	11/18723	0.001752614	0.00499401	0.0021033	HSPA5/TP53	2
2/108	11/18723	0.001752614	0.00499401	0.0021033	TNF/TLR4	2
2/108	11/18723	0.001752614	0.00499401	0.0021033	APOE/PTPN1	2
4/108	89/18723	0.001754688	0.00499401	0.0021033	TLR4/BCL2/NFE	4
4/108	89/18723	0.001754688	0.00499401	0.0021033	TNF/IL1B/TLR4	4
4/108	89/18723	0.001754688	0.00499401	0.0021033	IL1B/CAT/IL1A	4
5/108	150/18723	0.001775145	0.005048405	0.00212621	IL6/FADD/EDN1	5
6/108	221/18723	0.001778907	0.005055273	0.0021291	AKT1/NFE2L2/F	6
7/108	301/18723	0.001809253	0.005135346	0.00216283	TNF/IL6/MMP9/	7
3/108	42/18723	0.001819395	0.005135346	0.00216283	IL4/CXCL10/MA	3
3/108	42/18723	0.001819395	0.005135346	0.00216283	TP53/FADD/CAV	3
3/108	42/18723	0.001819395	0.005135346	0.00216283	MMP9/MMP2/MMP	3
3/108	42/18723	0.001819395	0.005135346	0.00216283	IL4/TLR4/BCL2	3
3/108	42/18723	0.001819395	0.005135346	0.00216283	TNF/EDN1/PTPN	3
3/108	42/18723	0.001819395	0.005135346	0.00216283	ICAM1/CASP3/E	3
3/108	42/18723	0.001819395	0.005135346	0.00216283	EGFR/SELE/ESF	3
3/108	42/18723	0.001819395	0.005135346	0.00216283	IL6/CCL2/CD40	3
5/108	151/18723	0.001827637	0.005144849	0.00216683	BCL2/IL1A/EDN	5
5/108	151/18723	0.001827637	0.005144849	0.00216683	TNF/CXCL10/ST	5
4/108	90/18723	0.001828244	0.005144849	0.00216683	AKT1/NFE2L2/C	4
4/108	90/18723	0.001828244	0.005144849	0.00216683	IFNG/STAT3/IN	4
7/108	302/18723	0.001843542	0.005184013	0.00218332	TNF/IL6/MMP9/	7
7/108	303/18723	0.001878335	0.005277895	0.00222286	EGFR/NFKB1/GA	7
5/108	152/18723	0.001881259	0.005282159	0.00222466	TNF/CXCL10/ST	5
4/108	91/18723	0.001903912	0.005337779	0.00224808	FASN/AKT1/SEF	4
4/108	91/18723	0.001903912	0.005337779	0.00224808	BCL2/EDN1/MAF	4
7/108	304/18723	0.001913635	0.005357037	0.0022562	TNF/IL6/MMP9/	7
7/108	304/18723	0.001913635	0.005357037	0.0022562	IL1B/IL4/HSPA	7
6/108	225/18723	0.001947178	0.005436185	0.00228953	RAF1/PTGS2/CA	6
3/108	43/18723	0.001947701	0.005436185	0.00228953	TNF/MTOR/GSK3	3
3/108	43/18723	0.001947701	0.005436185	0.00228953	IL1B/TLR4/MTO	3
3/108	43/18723	0.001947701	0.005436185	0.00228953	BCL2/TP53/PML	3
4/108	92/18723	0.001981723	0.005522931	0.00232606	IL1B/TP53/GAT	4
4/108	92/18723	0.001981723	0.005522931	0.00232606	TNF/PTGS2/CCL	4
5/108	154/18723	0.001991948	0.005547309	0.00233633	IFNG/STAT3/TF	5
5/108	155/18723	0.002049044	0.005697862	0.00239974	BCL2/HMGCR/MA	5

5/108	155/18723	0.002049044	0.005697862	0.00239974	BCL2/MAPK14/I	5
7/108	308/18723	0.002060021	0.005707903	0.00240397	TNF/IFNG/IL1E	7
4/108	93/18723	0.002061708	0.005707903	0.00240397	TP53/PML/MAPK	4
4/108	93/18723	0.002061708	0.005707903	0.00240397	BCL2/TP53/AKT	4
4/108	93/18723	0.002061708	0.005707903	0.00240397	CCL2/BCL2/TP5	4
3/108	44/18723	0.002081509	0.005707903	0.00240397	FASN/AKT1/GSK	3
3/108	44/18723	0.002081509	0.005707903	0.00240397	CD40LG/AKT1/C	3
3/108	44/18723	0.002081509	0.005707903	0.00240397	IFNG/IL6/TLR4	3
3/108	44/18723	0.002081509	0.005707903	0.00240397	IFNG/IL6/TLR4	3
3/108	44/18723	0.002081509	0.005707903	0.00240397	IL6/EGFR/SPP1	3
3/108	44/18723	0.002081509	0.005707903	0.00240397	CASP3/FADD/IC	3
3/108	44/18723	0.002081509	0.005707903	0.00240397	BAK1/ESR1/AR	3
3/108	44/18723	0.002081509	0.005707903	0.00240397	HSPA5/BCL2L1/	3
2/108	12/18723	0.002095229	0.005707903	0.00240397	BCL2/IL1A	2
2/108	12/18723	0.002095229	0.005707903	0.00240397	ASS1/CPS1	2
2/108	12/18723	0.002095229	0.005707903	0.00240397	IL1B/EDN1	2
2/108	12/18723	0.002095229	0.005707903	0.00240397	IL4/GATA3	2
2/108	12/18723	0.002095229	0.005707903	0.00240397	IL1B/MAPK3	2
2/108	12/18723	0.002095229	0.005707903	0.00240397	MMP9/MTOR	2
2/108	12/18723	0.002095229	0.005707903	0.00240397	EDN1/MTOR	2
2/108	12/18723	0.002095229	0.005707903	0.00240397	MAPK1/MAPK3	2
2/108	12/18723	0.002095229	0.005707903	0.00240397	MAPK1/MAPK3	2
2/108	12/18723	0.002095229	0.005707903	0.00240397	MAPK1/MAPK3	2
2/108	12/18723	0.002095229	0.005707903	0.00240397	ASS1/CPS1	2
2/108	12/18723	0.002095229	0.005707903	0.00240397	GATA3/STAT1	2
2/108	12/18723	0.002095229	0.005707903	0.00240397	FASN/ICAM1	2
2/108	12/18723	0.002095229	0.005707903	0.00240397	TLR4/AKT1	2
2/108	12/18723	0.002095229	0.005707903	0.00240397	IL6/HSF1	2
2/108	12/18723	0.002095229	0.005707903	0.00240397	RELA/NFKB1	2
4/108	94/18723	0.002143897	0.005827799	0.00245446	PTGS2/KDR/AKT	4
4/108	94/18723	0.002143897	0.005827799	0.00245446	F2/PLG/JUN/AF	4
4/108	94/18723	0.002143897	0.005827799	0.00245446	ICAM1/PML/SLC	4
5/108	157/18723	0.002166814	0.005885833	0.00247891	IL1B/IL6/GATA	5
3/108	45/18723	0.002220901	0.006000804	0.00252733	KDR/BCL2/PARF	3
3/108	45/18723	0.002220901	0.006000804	0.00252733	IFNG/STAT3/IN	3
3/108	45/18723	0.002220901	0.006000804	0.00252733	HSPA5/ESR1/CA	3
3/108	45/18723	0.002220901	0.006000804	0.00252733	IL1B/NOS2/EDN	3
3/108	45/18723	0.002220901	0.006000804	0.00252733	PAH/GATA3/TYF	3
3/108	45/18723	0.002220901	0.006000804	0.00252733	EGFR/AKT1/ERE	3
3/108	45/18723	0.002220901	0.006000804	0.00252733	IL1B/GATA3/MA	3
4/108	95/18723	0.002228322	0.006000804	0.00252733	STAT3/AKT1/NO	4
4/108	95/18723	0.002228322	0.006000804	0.00252733	TLR4/IRF1/HSF	4
4/108	95/18723	0.002228322	0.006000804	0.00252733	TLR4/IRF1/HSF	4
4/108	95/18723	0.002228322	0.006000804	0.00252733	RELA/AKT1/CAV	4
4/108	95/18723	0.002228322	0.006000804	0.00252733	ICAM1/PML/SLC	4
4/108	96/18723	0.002315013	0.006229792	0.00262377	MAPK14/EDN1/M	4
8/108	403/18723	0.002326908	0.006257315	0.00263536	BCL2/HMGCR/MA	8
3/108	46/18723	0.002365956	0.006348676	0.00267384	VCAM1/SELE/IC	3
3/108	46/18723	0.002365956	0.006348676	0.00267384	BCL2/MAPK14/M	3
3/108	46/18723	0.002365956	0.006348676	0.00267384	SOAT1/APOE/AF	3
6/108	234/18723	0.002369722	0.00635424	0.00267618	SPP1/HSP90AA1	6
4/108	97/18723	0.002404002	0.006441557	0.00271296	IL4/PRKAB1/HS	4
6/108	235/18723	0.002420617	0.006481453	0.00272976	TNF/CCL2/EGFF	6
2/108	13/18723	0.002466874	0.00655853	0.00276222	NOS3/MTOR	2

2/108	13/18723	0.002466874	0.00655853	0.00276222	IL6/STAT3	2
2/108	13/18723	0.002466874	0.00655853	0.00276222	NOS3/NOS2	2
2/108	13/18723	0.002466874	0.00655853	0.00276222	IL1B/PTGS2	2
2/108	13/18723	0.002466874	0.00655853	0.00276222	TP53/PARP1	2
2/108	13/18723	0.002466874	0.00655853	0.00276222	IL6/RELA	2
2/108	13/18723	0.002466874	0.00655853	0.00276222	AR/PARP1	2
2/108	13/18723	0.002466874	0.00655853	0.00276222	TP53/NFE2L2	2
2/108	13/18723	0.002466874	0.00655853	0.00276222	BAK1/PTPN1	2
2/108	13/18723	0.002466874	0.00655853	0.00276222	IFNG/HSP90AA1	2
4/108	98/18723	0.002495317	0.006629455	0.00279209	NOS3/EDN1/MT(	4
3/108	47/18723	0.002516752	0.006653441	0.0028022	MMP9/GATA3/ST	3
3/108	47/18723	0.002516752	0.006653441	0.0028022	IL4/ERBB2/IRI	3
3/108	47/18723	0.002516752	0.006653441	0.0028022	STAT3/STAT1/M	3
3/108	47/18723	0.002516752	0.006653441	0.0028022	CASP3/FADD/IC	3
3/108	47/18723	0.002516752	0.006653441	0.0028022	CCL2/AKT1/VDA	3
3/108	47/18723	0.002516752	0.006653441	0.0028022	CCL2/STAT3/IC	3
3/108	47/18723	0.002516752	0.006653441	0.0028022	CCL2/STAT3/IC	3
5/108	163/18723	0.002549789	0.006736036	0.00283698	BCL2/TP53/AKT	5
4/108	99/18723	0.00258899	0.006815616	0.0028705	IL6/IL4/F2/HM	4
4/108	99/18723	0.00258899	0.006815616	0.0028705	IFNG/STAT3/IN	4
4/108	99/18723	0.00258899	0.006815616	0.0028705	IL1B/IL6/GATA	4
4/108	99/18723	0.00258899	0.006815616	0.0028705	HSPA5/HAP1/TI	4
4/108	99/18723	0.00258899	0.006815616	0.0028705	BCL2/MAPK14/I	4
5/108	164/18723	0.002618098	0.00688259	0.0028987	TNF/CXCL8/BCI	5
5/108	164/18723	0.002618098	0.00688259	0.0028987	AKT1/NFE2L2/I	5
3/108	48/18723	0.002673364	0.007008247	0.00295163	CCL2/AKT1/HM(	3
3/108	48/18723	0.002673364	0.007008247	0.00295163	STAT3/TP53/GS	3
3/108	48/18723	0.002673364	0.007008247	0.00295163	IFNG/TP53/MAI	3
3/108	48/18723	0.002673364	0.007008247	0.00295163	TNF/MTOR/GSK3	3
7/108	324/18723	0.002734275	0.007162923	0.00301677	TNF/EGFR/VDR/	7
6/108	241/18723	0.002743492	0.007182056	0.00302483	CCL2/EGFR/BCI	6
4/108	101/18723	0.00278353	0.007281792	0.00306683	IFNG/STAT3/IN	4
6/108	242/18723	0.002800299	0.00732056	0.00308316	HSPA5/RELA/AK	6
5/108	167/18723	0.002830999	0.007392948	0.00311365	SOAT1/HMGCR/(	5
3/108	49/18723	0.002835867	0.007392948	0.00311365	IL1B/PTGS2/EI	3
3/108	49/18723	0.002835867	0.007392948	0.00311365	IL1B/PTGS2/EI	3
3/108	49/18723	0.002835867	0.007392948	0.00311365	CXCL10/HSP90A	3
2/108	14/18723	0.002867208	0.007402681	0.00311775	IL1B/NFKB1	2
2/108	14/18723	0.002867208	0.007402681	0.00311775	NFE2L2/ASS1	2
2/108	14/18723	0.002867208	0.007402681	0.00311775	AKT1/VDAC1	2
2/108	14/18723	0.002867208	0.007402681	0.00311775	VDR/HMGCR	2
2/108	14/18723	0.002867208	0.007402681	0.00311775	BCL2/IRF1	2
2/108	14/18723	0.002867208	0.007402681	0.00311775	MMP9/MTOR	2
2/108	14/18723	0.002867208	0.007402681	0.00311775	TNF/SERPINE1	2
2/108	14/18723	0.002867208	0.007402681	0.00311775	HSPA5/TP53	2
2/108	14/18723	0.002867208	0.007402681	0.00311775	GATA3/STAT1	2
2/108	14/18723	0.002867208	0.007402681	0.00311775	IL6/STAT3	2
2/108	14/18723	0.002867208	0.007402681	0.00311775	AKT1/VDAC1	2
2/108	14/18723	0.002867208	0.007402681	0.00311775	IFNG/IL4	2
2/108	14/18723	0.002867208	0.007402681	0.00311775	NFE2L2/CAV1	2
2/108	14/18723	0.002867208	0.007402681	0.00311775	IL4/STAT3	2
7/108	327/18723	0.002877649	0.007424533	0.00312695	CXCL10/BCL2/I	7
4/108	102/18723	0.002884457	0.00743188	0.00313005	IL4/TLR4/ERBI	4
4/108	102/18723	0.002884457	0.00743188	0.00313005	AKT1/MMP9/VDA	4

5/108	169/18723	0.002979724	0.007672075	0.00323121	IL4/STAT3/APF	5
4/108	103/18723	0.002987861	0.007677228	0.00323338	IFNG/STAT3/IN	4
4/108	103/18723	0.002987861	0.007677228	0.00323338	IFNG/STAT3/IN	4
4/108	103/18723	0.002987861	0.007677228	0.00323338	BCL2/IL1A/MAF	4
3/108	50/18723	0.003004332	0.007687978	0.00323791	PTGS2/MAPK14/	3
3/108	50/18723	0.003004332	0.007687978	0.00323791	STAT1/FADD/PT	3
3/108	50/18723	0.003004332	0.007687978	0.00323791	TNF/MAPK1/MAF	3
3/108	50/18723	0.003004332	0.007687978	0.00323791	TNF/ESR1/AR	3
3/108	50/18723	0.003004332	0.007687978	0.00323791	SOAT1/APOE/AF	3
3/108	50/18723	0.003004332	0.007687978	0.00323791	IFNG/GATA3/PA	3
4/108	104/18723	0.003093772	0.007906073	0.00332976	IFNG/IL4/IRF1	4
4/108	104/18723	0.003093772	0.007906073	0.00332976	MAPK14/EDN1/M	4
5/108	171/18723	0.003134021	0.008003479	0.00337078	IL1B/HMGCR/ED	5
3/108	51/18723	0.00317883	0.008090393	0.00340739	IL1B/PTGS2/ED	3
3/108	51/18723	0.00317883	0.008090393	0.00340739	NFKBIA/CAV1/A	3
3/108	51/18723	0.00317883	0.008090393	0.00340739	IFNG/IL4/GATA	3
3/108	51/18723	0.00317883	0.008090393	0.00340739	TNF/CXCL10/MA	3
3/108	51/18723	0.00317883	0.008090393	0.00340739	IL6/EGFR/SPP1	3
7/108	333/18723	0.00318167	0.008092133	0.00340812	TNF/IFNG/EGFF	7
4/108	105/18723	0.003202221	0.008138887	0.00342781	TLR4/ESR1/IRF	4
6/108	249/18723	0.003223023	0.008186218	0.00344775	BCL2/SPP1/TP5	6
2/108	15/18723	0.003295894	0.008303883	0.0034973	PAH/TYR	2
2/108	15/18723	0.003295894	0.008303883	0.0034973	APOE/APOB	2
2/108	15/18723	0.003295894	0.008303883	0.0034973	IL4/GATA3	2
2/108	15/18723	0.003295894	0.008303883	0.0034973	PTGS2/SOD1	2
2/108	15/18723	0.003295894	0.008303883	0.0034973	MAPK1/MAPK3	2
2/108	15/18723	0.003295894	0.008303883	0.0034973	MAPK1/MAPK3	2
2/108	15/18723	0.003295894	0.008303883	0.0034973	MTOR/GSK3B	2
2/108	15/18723	0.003295894	0.008303883	0.0034973	MTOR/GSK3B	2
2/108	15/18723	0.003295894	0.008303883	0.0034973	HSPA5/PTPN1	2
2/108	15/18723	0.003295894	0.008303883	0.0034973	IL4/MAPK14	2
2/108	15/18723	0.003295894	0.008303883	0.0034973	CCL2/AKT1	2
2/108	15/18723	0.003295894	0.008303883	0.0034973	IL1B/PTGS2	2
4/108	106/18723	0.003313235	0.008336384	0.00351099	IFNG/STAT3/IN	4
4/108	106/18723	0.003313235	0.008336384	0.00351099	IL4/TLR4/ERBE	4
3/108	52/18723	0.003359429	0.008430012	0.00355042	IL1B/IL6/MTOF	3
3/108	52/18723	0.003359429	0.008430012	0.00355042	PML/MAPK1/MAF	3
3/108	52/18723	0.003359429	0.008430012	0.00355042	NFKBIA/JUN/HM	3
3/108	52/18723	0.003359429	0.008430012	0.00355042	STAT1/FADD/PT	3
6/108	252/18723	0.00341809	0.008571483	0.00361001	TNF/RAF1/PTGS	6
4/108	107/18723	0.003426845	0.008581971	0.00361442	EGFR/TP53/JUN	4
4/108	107/18723	0.003426845	0.008581971	0.00361442	AKT1/NFE2L2/C	4
5/108	175/18723	0.003459797	0.008658719	0.00364675	CXCL8/EGFR/IC	5
6/108	253/18723	0.003485028	0.008716054	0.0036709	EGFR/NFKB1/MA	6
3/108	53/18723	0.003546196	0.008863129	0.00373284	IL1B/NOS2/EDN	3
5/108	177/18723	0.003631512	0.009070324	0.0038201	IL1B/CAT/IL1A	5
3/108	54/18723	0.003739195	0.009298517	0.00391621	IL1B/GATA3/MA	3
2/108	16/18723	0.003752598	0.009298517	0.00391621	ICAM1/FADD	2
2/108	16/18723	0.003752598	0.009298517	0.00391621	BAK1/TP53	2
2/108	16/18723	0.003752598	0.009298517	0.00391621	BAK1/ESR1	2
2/108	16/18723	0.003752598	0.009298517	0.00391621	TNF/IL1B	2
2/108	16/18723	0.003752598	0.009298517	0.00391621	APOE/APOB	2
2/108	16/18723	0.003752598	0.009298517	0.00391621	HSF1/SOD1	2
2/108	16/18723	0.003752598	0.009298517	0.00391621	HSF1/FADD	2

2/108	16/18723	0.003752598	0.009298517	0.00391621	BAK1/TP53	2
2/108	16/18723	0.003752598	0.009298517	0.00391621	ESR1/AR	2
2/108	16/18723	0.003752598	0.009298517	0.00391621	PTGS2/MAPK14	2
2/108	16/18723	0.003752598	0.009298517	0.00391621	BAK1/FADD	2
4/108	110/18723	0.003783534	0.009350484	0.00393809	TNF/IL4/AHR/H	4
4/108	110/18723	0.003783534	0.009350484	0.00393809	PTGS2/EDN1/CA	4
4/108	110/18723	0.003783534	0.009350484	0.00393809	PAH/BCL2/GATA	4
4/108	110/18723	0.003783534	0.009350484	0.00393809	IL4/SELE/INSR	4
6/108	258/18723	0.00383448	0.009470157	0.0039885	IL4/NFKB1/GAT	6
4/108	111/18723	0.003907812	0.009644922	0.0040621	IL1B/NOS2/EDN	4
3/108	55/18723	0.003938491	0.00966978	0.00407257	IL4/CXCL10/MA	3
3/108	55/18723	0.003938491	0.00966978	0.00407257	FASN/AKT1/GSK	3
3/108	55/18723	0.003938491	0.00966978	0.00407257	NOS3/GATA3/MT	3
3/108	55/18723	0.003938491	0.00966978	0.00407257	IL1B/NCF1/MAP	3
3/108	55/18723	0.003938491	0.00966978	0.00407257	HSPA5/FADD/MT	3
3/108	55/18723	0.003938491	0.00966978	0.00407257	IL4/ERBB2/IRF	3
3/108	55/18723	0.003938491	0.00966978	0.00407257	IL4/CXCL10/MA	3
3/108	55/18723	0.003938491	0.00966978	0.00407257	CCL2/MAPK1/MA	3
6/108	260/18723	0.00398131	0.009768522	0.00411416	HSPA5/EGFR/NO	6
5/108	182/18723	0.004087487	0.010022486	0.00422112	IL4/STAT3/APP	5
6/108	262/18723	0.004132273	0.010108585	0.00425738	TNF/RAF1/PTGS	6
3/108	56/18723	0.004144143	0.010108585	0.00425738	EGFR/SELE/ESR	3
3/108	56/18723	0.004144143	0.010108585	0.00425738	ICAM1/CAV1/MT	3
3/108	56/18723	0.004144143	0.010108585	0.00425738	TLR4/IRF1/HSP	3
3/108	56/18723	0.004144143	0.010108585	0.00425738	TLR4/IRF1/HSP	3
3/108	56/18723	0.004144143	0.010108585	0.00425738	TNF/IL6/GATA3	3
3/108	56/18723	0.004144143	0.010108585	0.00425738	IL1B/PTGS2/ED	3
3/108	56/18723	0.004144143	0.010108585	0.00425738	PLAT/F3/PTPN1	3
4/108	113/18723	0.004164613	0.010145332	0.00427286	CXCL8/SELE/IN	4
4/108	113/18723	0.004164613	0.010145332	0.00427286	CXCL8/CCL2/GA	4
6/108	263/18723	0.004209326	0.010235301	0.00431075	TNF/EGFR/VDR/	6
2/108	17/18723	0.004236989	0.010235301	0.00431075	GLS/ASS1	2
2/108	17/18723	0.004236989	0.010235301	0.00431075	RELA/BCL2	2
2/108	17/18723	0.004236989	0.010235301	0.00431075	MAPK14/MTOR	2
2/108	17/18723	0.004236989	0.010235301	0.00431075	IL1B/CAV1	2
2/108	17/18723	0.004236989	0.010235301	0.00431075	IL1B/GSK3B	2
2/108	17/18723	0.004236989	0.010235301	0.00431075	NCF1/INSR	2
2/108	17/18723	0.004236989	0.010235301	0.00431075	TP53/SERPINE1	2
2/108	17/18723	0.004236989	0.010235301	0.00431075	TNF/IL1B	2
2/108	17/18723	0.004236989	0.010235301	0.00431075	TNF/IL1B	2
2/108	17/18723	0.004236989	0.010235301	0.00431075	MAPK1/MAPK3	2
2/108	17/18723	0.004236989	0.010235301	0.00431075	MAPK1/MAPK3	2
2/108	17/18723	0.004236989	0.010235301	0.00431075	BCL2/CAV1	2
8/108	445/18723	0.004247897	0.010255053	0.00431907	HSPA5/SPP1/AK	8
5/108	184/18723	0.004280859	0.010327986	0.00434978	IFNG/CCL2/HAP	5
4/108	114/18723	0.004297191	0.010360729	0.00436357	AKT1/NFE2L2/C	4
3/108	57/18723	0.004356213	0.010469412	0.00440935	IL4/STAT3/PAR	3
3/108	57/18723	0.004356213	0.010469412	0.00440935	IL4/CXCL10/MA	3
3/108	57/18723	0.004356213	0.010469412	0.00440935	EGFR/HSP90AA1	3
3/108	57/18723	0.004356213	0.010469412	0.00440935	TLR4/FADD/EDN	3
3/108	57/18723	0.004356213	0.010469412	0.00440935	MMP9/GATA3/ST	3
4/108	115/18723	0.004432591	0.010639353	0.00448092	BCL2/MAPK14/M	4
4/108	115/18723	0.004432591	0.010639353	0.00448092	CXCL10/HAP1/C	4
5/108	186/18723	0.004480682	0.010747912	0.00452664	TNF/TLR4/RELA	5

4/108	116/18723	0.004570841	0.010924707	0.0046011	ESR2/ESR1/AR/	4
4/108	116/18723	0.004570841	0.010924707	0.0046011	IL1B/PTGS2/ED	4
4/108	116/18723	0.004570841	0.010924707	0.0046011	CXCL10/HAP1/C	4
3/108	58/18723	0.004574757	0.010924707	0.0046011	STAT1/FADD/PT	3
3/108	58/18723	0.004574757	0.010924707	0.0046011	TNF/EDN1/PTPN	3
3/108	58/18723	0.004574757	0.010924707	0.0046011	TNF/MTOR/GSK3	3
3/108	58/18723	0.004574757	0.010924707	0.0046011	TP53/CAV1/SOD	3
5/108	187/18723	0.004583049	0.010930602	0.00460358	IL4/ERBB2/IRF	5
5/108	187/18723	0.004583049	0.010930602	0.00460358	AKT1/NFE2L2/C	5
5/108	188/18723	0.004687073	0.0111716	0.00470508	BCL2/SPP1/TP5	5
2/108	18/18723	0.004748738	0.011176653	0.00470721	NFE2L2/CYP3A4	2
2/108	18/18723	0.004748738	0.011176653	0.00470721	TP53/HMGCR	2
2/108	18/18723	0.004748738	0.011176653	0.00470721	TP53/HMGCR	2
2/108	18/18723	0.004748738	0.011176653	0.00470721	TNF/IL1B	2
2/108	18/18723	0.004748738	0.011176653	0.00470721	IL1B/EDN1	2
2/108	18/18723	0.004748738	0.011176653	0.00470721	NFKB1/CAV1	2
2/108	18/18723	0.004748738	0.011176653	0.00470721	SOAT1/APOE	2
2/108	18/18723	0.004748738	0.011176653	0.00470721	SOAT1/APOE	2
2/108	18/18723	0.004748738	0.011176653	0.00470721	SOAT1/APOE	2
2/108	18/18723	0.004748738	0.011176653	0.00470721	PAH/SLC6A4	2
2/108	18/18723	0.004748738	0.011176653	0.00470721	AKT1/SOD1	2
2/108	18/18723	0.004748738	0.011176653	0.00470721	APRT/ATIC	2
2/108	18/18723	0.004748738	0.011176653	0.00470721	STAT3/SOD1	2
2/108	18/18723	0.004748738	0.011176653	0.00470721	MAPK1/MAPK3	2
2/108	18/18723	0.004748738	0.011176653	0.00470721	FADD/CAV1	2
2/108	18/18723	0.004748738	0.011176653	0.00470721	AKT1/MAPK1	2
2/108	18/18723	0.004748738	0.011176653	0.00470721	ESR1/AR	2
2/108	18/18723	0.004748738	0.011176653	0.00470721	ESR1/AR	2
2/108	18/18723	0.004748738	0.011176653	0.00470721	PTGS2/SOD1	2
2/108	18/18723	0.004748738	0.011176653	0.00470721	MAPK1/MAPK3	2
3/108	59/18723	0.004799834	0.011289835	0.00475488	NFKB1/SOD1/AF	3
4/108	118/18723	0.004856	0.011414793	0.00480751	CXCL10/HAP1/C	4
5/108	190/18723	0.004900146	0.011511356	0.00484818	IL4/STAT3/APF	5
3/108	60/18723	0.005031498	0.011812534	0.00497502	TLR4/RELA/NFK	3
4/108	120/18723	0.005152883	0.012082401	0.00508868	CXCL8/CCL2/GA	4
4/108	120/18723	0.005152883	0.012082401	0.00508868	AKT1/HSP90AA1	4
3/108	61/18723	0.005269801	0.012237575	0.00515404	TNF/IL1B/VCAM	3
3/108	61/18723	0.005269801	0.012237575	0.00515404	IL6/IL4/TLR4	3
3/108	61/18723	0.005269801	0.012237575	0.00515404	MAPK1/MAPK3/F	3
2/108	19/18723	0.005287519	0.012237575	0.00515404	TP53/HK2	2
2/108	19/18723	0.005287519	0.012237575	0.00515404	IL6/HSF1	2
2/108	19/18723	0.005287519	0.012237575	0.00515404	TP53/PML	2
2/108	19/18723	0.005287519	0.012237575	0.00515404	APRT/ATIC	2
2/108	19/18723	0.005287519	0.012237575	0.00515404	NFKB1/APOB	2
2/108	19/18723	0.005287519	0.012237575	0.00515404	MAPK1/MAPK3	2
2/108	19/18723	0.005287519	0.012237575	0.00515404	TNF/IL1B	2
2/108	19/18723	0.005287519	0.012237575	0.00515404	CASP3/CASP9	2
2/108	19/18723	0.005287519	0.012237575	0.00515404	IL4/GATA3	2
2/108	19/18723	0.005287519	0.012237575	0.00515404	IL4/GATA3	2
2/108	19/18723	0.005287519	0.012237575	0.00515404	CASP3/CASP9	2
2/108	19/18723	0.005287519	0.012237575	0.00515404	INSR/BCL2L1	2
2/108	19/18723	0.005287519	0.012237575	0.00515404	EGFR/SPP1	2
2/108	19/18723	0.005287519	0.012237575	0.00515404	IFNG/INSR	2
2/108	19/18723	0.005287519	0.012237575	0.00515404	IL4/CCL2	2

2/108	19/18723	0.005287519	0.012237575	0.00515404	MMP9/MTOR	2
2/108	19/18723	0.005287519	0.012237575	0.00515404	ESR1/GATA3	2
2/108	19/18723	0.005287519	0.012237575	0.00515404	FADD/CAV1	2
4/108	122/18723	0.005461705	0.012617378	0.005314	CXCL8/CXCL10/	4
4/108	122/18723	0.005461705	0.012617378	0.005314	IL4/ERBB2/IRF	4
4/108	122/18723	0.005461705	0.012617378	0.005314	CXCL10/HAP1/C	4
6/108	278/18723	0.005497124	0.012691393	0.00534517	IFNG/CCL2/BCL	6
3/108	62/18723	0.005514797	0.012708746	0.00535248	EDN1/HMOX1/AE	3
3/108	62/18723	0.005514797	0.012708746	0.00535248	IL1B/GATA3/IL	3
3/108	62/18723	0.005514797	0.012708746	0.00535248	IL1B/GATA3/IL	3
4/108	123/18723	0.005620658	0.012944754	0.00545187	IL1B/PTGS2/ED	4
8/108	467/18723	0.005651753	0.013008389	0.00547868	BCL2/SPP1/ERE	8
8/108	468/18723	0.005723072	0.013164468	0.00554441	TNF/IL6/STAT3	8
3/108	63/18723	0.005766534	0.013256319	0.00558309	IL1B/GATA3/MA	3
4/108	124/18723	0.005782673	0.013277159	0.00559187	ICAM1/F2/NOS2	4
4/108	124/18723	0.005782673	0.013277159	0.00559187	IFNG/STAT3/IN	4
2/108	20/18723	0.005853009	0.013308417	0.00560504	AKT1/VDAC1	2
2/108	20/18723	0.005853009	0.013308417	0.00560504	PAH/GATA3	2
2/108	20/18723	0.005853009	0.013308417	0.00560504	PTGS2/HSPA5	2
2/108	20/18723	0.005853009	0.013308417	0.00560504	TNF/IFNG	2
2/108	20/18723	0.005853009	0.013308417	0.00560504	EGFR/SOD1	2
2/108	20/18723	0.005853009	0.013308417	0.00560504	PAH/GATA3	2
2/108	20/18723	0.005853009	0.013308417	0.00560504	GATA3/STAT1	2
2/108	20/18723	0.005853009	0.013308417	0.00560504	HSF1/HMOX1	2
2/108	20/18723	0.005853009	0.013308417	0.00560504	AKT1/APOB	2
2/108	20/18723	0.005853009	0.013308417	0.00560504	TNF/TLR4	2
2/108	20/18723	0.005853009	0.013308417	0.00560504	AKT1/BCL2L1	2
2/108	20/18723	0.005853009	0.013308417	0.00560504	AKT1/NFE2L2	2
2/108	20/18723	0.005853009	0.013308417	0.00560504	BCL2L1/PTPN1	2
2/108	20/18723	0.005853009	0.013308417	0.00560504	NFE2L2/CAV1	2
2/108	20/18723	0.005853009	0.013308417	0.00560504	TNF/AKT1	2
2/108	20/18723	0.005853009	0.013308417	0.00560504	TNF/TLR4	2
4/108	125/18723	0.005947778	0.013507536	0.0056889	AKT1/HSP90AA1	4
4/108	125/18723	0.005947778	0.013507536	0.0056889	TNF/GATA3/EDN	4
3/108	64/18723	0.006025062	0.013658263	0.00575238	NFE2L2/GSR/SC	3
3/108	64/18723	0.006025062	0.013658263	0.00575238	IL1B/HMGCR/ED	3
3/108	64/18723	0.006025062	0.013658263	0.00575238	CXCL10/CCL2/C	3
7/108	375/18723	0.006050909	0.013708577	0.00577357	IL1B/TLR4/REL	7
6/108	284/18723	0.00608559	0.013778834	0.00580316	TNF/IFNG/EGFF	6
3/108	65/18723	0.006290428	0.014216898	0.00598766	NOS3/GATA3/MT	3
3/108	65/18723	0.006290428	0.014216898	0.00598766	IL6/EGFR/SPP1	3
3/108	65/18723	0.006290428	0.014216898	0.00598766	AR/MAPK1/MAPK	3
5/108	202/18723	0.006325358	0.014287242	0.00601728	IFNG/IL4/HAP1	5
7/108	379/18723	0.006400994	0.014435628	0.00607978	TNF/RAF1/PTGS	7
2/108	21/18723	0.006444889	0.014435628	0.00607978	TP53/PARP1	2
2/108	21/18723	0.006444889	0.014435628	0.00607978	HAP1/SOD1	2
2/108	21/18723	0.006444889	0.014435628	0.00607978	APRT/ATIC	2
2/108	21/18723	0.006444889	0.014435628	0.00607978	SOAT1/APOB	2
2/108	21/18723	0.006444889	0.014435628	0.00607978	AKT1/MTOR	2
2/108	21/18723	0.006444889	0.014435628	0.00607978	IL1B/EDN1	2
2/108	21/18723	0.006444889	0.014435628	0.00607978	ASS1/CPS1	2
2/108	21/18723	0.006444889	0.014435628	0.00607978	MTOR/GSK3B	2
2/108	21/18723	0.006444889	0.014435628	0.00607978	GATA3/STAT1	2
2/108	21/18723	0.006444889	0.014435628	0.00607978	CXCL10/SERPIN	2

2/108	21/18723	0.006444889	0.014435628	0.00607978	INSR/APOE	2
2/108	21/18723	0.006444889	0.014435628	0.00607978	BCL2/MTOR	2
2/108	21/18723	0.006444889	0.014435628	0.00607978	TP53/SLC6A4	2
5/108	203/18723	0.006455946	0.014447813	0.00608491	TNF/EGFR/MAPK	5
4/108	128/18723	0.006461875	0.014447813	0.00608491	KDR/BCL2/SERF	4
4/108	128/18723	0.006461875	0.014447813	0.00608491	TNF/IFNG/MTOF	4
3/108	66/18723	0.006562678	0.014655736	0.00617248	HSD17B6/NFKB1	3
3/108	66/18723	0.006562678	0.014655736	0.00617248	PTGS2/AKT1/MA	3
7/108	383/18723	0.006766003	0.015100818	0.00635993	SPP1/AKT1/ICA	7
3/108	67/18723	0.006841855	0.015233882	0.00641597	IFNG/HMOX1/SO	3
3/108	67/18723	0.006841855	0.015233882	0.00641597	EGFR/SELE/ESF	3
3/108	67/18723	0.006841855	0.015233882	0.00641597	IL1B/AKT1/EDN	3
3/108	67/18723	0.006841855	0.015233882	0.00641597	IFNG/IL4/GATA	3
4/108	131/18723	0.007004672	0.015577926	0.00656087	TNF/CXCL8/CCI	4
4/108	131/18723	0.007004672	0.015577926	0.00656087	IL4/HSPA5/TP5	4
2/108	22/18723	0.00706284	0.015596412	0.00656866	BCL2/GATA3	2
2/108	22/18723	0.00706284	0.015596412	0.00656866	MTOR/AR	2
2/108	22/18723	0.00706284	0.015596412	0.00656866	STAT3/MMP3	2
2/108	22/18723	0.00706284	0.015596412	0.00656866	AKT1/PCNA	2
2/108	22/18723	0.00706284	0.015596412	0.00656866	IFNG/TLR4	2
2/108	22/18723	0.00706284	0.015596412	0.00656866	TLR4/STAT1	2
2/108	22/18723	0.00706284	0.015596412	0.00656866	KDR/VCAM1	2
2/108	22/18723	0.00706284	0.015596412	0.00656866	BAK1/TP53	2
2/108	22/18723	0.00706284	0.015596412	0.00656866	CAT/CAV1	2
2/108	22/18723	0.00706284	0.015596412	0.00656866	NFKB1/CAV1	2
2/108	22/18723	0.00706284	0.015596412	0.00656866	STAT3/PARP1	2
2/108	22/18723	0.00706284	0.015596412	0.00656866	IL1B/EDN1	2
3/108	68/18723	0.007128003	0.015721812	0.00662147	IL4/STAT3/PAF	3
3/108	68/18723	0.007128003	0.015721812	0.00662147	IFNG/IL4/GATA	3
4/108	133/18723	0.007382806	0.016264704	0.00685012	EGFR/SPP1/EDN	4
4/108	133/18723	0.007382806	0.016264704	0.00685012	AKT1/NFE2L2/C	4
3/108	69/18723	0.007421163	0.016287629	0.00685978	HSP90AA1/MAPK	3
3/108	69/18723	0.007421163	0.016287629	0.00685978	IL4/STAT3/PAF	3
3/108	69/18723	0.007421163	0.016287629	0.00685978	EDN1/MTOR/PAF	3
3/108	69/18723	0.007421163	0.016287629	0.00685978	IL4/HSP90AA1/	3
3/108	69/18723	0.007421163	0.016287629	0.00685978	IL1B/PTGS2/SI	3
3/108	69/18723	0.007421163	0.016287629	0.00685978	EGFR/SELE/ESF	3
5/108	210/18723	0.007423584	0.016287629	0.00685978	HSPA5/AKT1/HS	5
7/108	390/18723	0.007441819	0.0163181	0.00687261	CCL2/EGFR/BCI	7
4/108	134/18723	0.007576826	0.016594752	0.00698913	AKT1/NFE2L2/C	4
4/108	134/18723	0.007576826	0.016594752	0.00698913	AKT1/ERBB2/MT	4
2/108	23/18723	0.007706547	0.016713102	0.00703897	AKT1/GSK3B	2
2/108	23/18723	0.007706547	0.016713102	0.00703897	GLS/CPS1	2
2/108	23/18723	0.007706547	0.016713102	0.00703897	ERBB2/SOD1	2
2/108	23/18723	0.007706547	0.016713102	0.00703897	KDR/GATA3	2
2/108	23/18723	0.007706547	0.016713102	0.00703897	HSPA5/HSP90AA	2
2/108	23/18723	0.007706547	0.016713102	0.00703897	TNF/CXCL10	2
2/108	23/18723	0.007706547	0.016713102	0.00703897	IL1B/PTGS2	2
2/108	23/18723	0.007706547	0.016713102	0.00703897	PTGS2/SOD1	2
2/108	23/18723	0.007706547	0.016713102	0.00703897	ERBB2/MTOR	2
2/108	23/18723	0.007706547	0.016713102	0.00703897	STAT3/PARP1	2
2/108	23/18723	0.007706547	0.016713102	0.00703897	NOS3/NOS2	2
2/108	23/18723	0.007706547	0.016713102	0.00703897	IL4/MAPK14	2
2/108	23/18723	0.007706547	0.016713102	0.00703897	STAT3/PTPN1	2

2/108	23/18723	0.007706547	0.016713102	0.00703897	FADD/CAV1	2
2/108	23/18723	0.007706547	0.016713102	0.00703897	EDN1/MTOR	2
2/108	23/18723	0.007706547	0.016713102	0.00703897	IL4/CCL2	2
2/108	23/18723	0.007706547	0.016713102	0.00703897	STAT3/NFE2L2	2
6/108	299/18723	0.007756632	0.016812008	0.00708063	CCL2/EGFR/BCl	6
4/108	136/18723	0.007974887	0.017245233	0.00726308	AKT1/ERBB2/MT	4
4/108	136/18723	0.007974887	0.017245233	0.00726308	AKT1/ERBB2/MT	4
4/108	136/18723	0.007974887	0.017245233	0.00726308	ESR2/ESR1/AR/	4
4/108	136/18723	0.007974887	0.017245233	0.00726308	BCL2/CAT/GATA	4
3/108	71/18723	0.008028675	0.017341568	0.00730366	BCL2/VDAC1/AF	3
3/108	71/18723	0.008028675	0.017341568	0.00730366	STAT3/MTOR/AF	3
4/108	137/18723	0.008178974	0.017656048	0.00743611	MAPK14/CAV1/A	4
3/108	72/18723	0.008343103	0.017854842	0.00751983	EDN1/MTOR/PAF	3
3/108	72/18723	0.008343103	0.017854842	0.00751983	CXCL8/PML/CAV	3
3/108	72/18723	0.008343103	0.017854842	0.00751983	IL1B/HMGCR/AF	3
2/108	24/18723	0.0083757	0.017854842	0.00751983	GATA3/ICAM1	2
2/108	24/18723	0.0083757	0.017854842	0.00751983	TLR4/IRF1	2
2/108	24/18723	0.0083757	0.017854842	0.00751983	TNF/IL1B	2
2/108	24/18723	0.0083757	0.017854842	0.00751983	BCL2/TYR	2
2/108	24/18723	0.0083757	0.017854842	0.00751983	PTGS2/NFE2L2	2
2/108	24/18723	0.0083757	0.017854842	0.00751983	PTGS2/CAT	2
2/108	24/18723	0.0083757	0.017854842	0.00751983	PLAT/SERPINE1	2
2/108	24/18723	0.0083757	0.017854842	0.00751983	IRF1/STAT1	2
2/108	24/18723	0.0083757	0.017854842	0.00751983	STAT3/EDN1	2
2/108	24/18723	0.0083757	0.017854842	0.00751983	HSF1/FADD	2
2/108	24/18723	0.0083757	0.017854842	0.00751983	TP53/ESR1	2
2/108	24/18723	0.0083757	0.017854842	0.00751983	TP53/HK2	2
2/108	24/18723	0.0083757	0.017854842	0.00751983	VDR/BAK1	2
2/108	24/18723	0.0083757	0.017854842	0.00751983	STAT3/PTPN1	2
2/108	24/18723	0.0083757	0.017854842	0.00751983	CCL2/AKT1	2
2/108	24/18723	0.0083757	0.017854842	0.00751983	CXCL8/EDN1	2
2/108	24/18723	0.0083757	0.017854842	0.00751983	BCL2/CAV1	2
2/108	24/18723	0.0083757	0.017854842	0.00751983	IFNG/CCL2	2
2/108	24/18723	0.0083757	0.017854842	0.00751983	CCL2/CD40LG	2
3/108	73/18723	0.008664695	0.018439492	0.00776606	EGFR/PARP1/PC	3
3/108	73/18723	0.008664695	0.018439492	0.00776606	TNF/MTOR/GSK3	3
3/108	73/18723	0.008664695	0.018439492	0.00776606	BCL2/GATA3/ST	3
5/108	219/18723	0.008810901	0.018740013	0.00789263	RAF1/PTGS2/HM	5
3/108	74/18723	0.008993484	0.019117518	0.00805163	IL4/STAT3/PAF	3
4/108	141/18723	0.009029561	0.019183346	0.00807935	RAF1/HSPA5/HS	4
2/108	25/18723	0.009069988	0.019193198	0.0080835	CD40LG/GATA3	2
2/108	25/18723	0.009069988	0.019193198	0.0080835	HSP90AA1/MAPK	2
2/108	25/18723	0.009069988	0.019193198	0.0080835	TP53/ESR1	2
2/108	25/18723	0.009069988	0.019193198	0.0080835	ESR1/AR	2
2/108	25/18723	0.009069988	0.019193198	0.0080835	IFNG/TP53	2
2/108	25/18723	0.009069988	0.019193198	0.0080835	CXCL10/CCL2	2
2/108	25/18723	0.009069988	0.019193198	0.0080835	STAT3/IGF2	2
5/108	221/18723	0.009141958	0.019334597	0.00814305	KDR/BCL2/SERF	5
6/108	310/18723	0.009176742	0.019397236	0.00816943	IFNG/CCL2/BCl	6
4/108	142/18723	0.009250878	0.019531943	0.00822617	AKT1/CAV1/AR/	4
4/108	142/18723	0.009250878	0.019531943	0.00822617	CXCL10/HAP1/C	4
3/108	75/18723	0.009329503	0.019653734	0.00827746	HSP90AA1/MAPK	3
3/108	75/18723	0.009329503	0.019653734	0.00827746	ESR1/IRF1/CAV	3
3/108	75/18723	0.009329503	0.019653734	0.00827746	IFNG/HMOX1/AF	3

3/108	75/18723	0.009329503	0.019653734	0.00827746	IFNG/CCL2/HAI	3
6/108	312/18723	0.00945368	0.019904159	0.00838293	CXCL10/HAP1/(	6
3/108	76/18723	0.009672784	0.020331261	0.00856281	STAT3/MAPK14/	3
3/108	76/18723	0.009672784	0.020331261	0.00856281	BCL2/GATA3/ST	3
3/108	76/18723	0.009672784	0.020331261	0.00856281	BCL2/TP53/PMI	3
2/108	26/18723	0.009789104	0.020472589	0.00862233	PTGS2/SLC6A4	2
2/108	26/18723	0.009789104	0.020472589	0.00862233	SELE/INSR	2
2/108	26/18723	0.009789104	0.020472589	0.00862233	TNF/GSK3B	2
2/108	26/18723	0.009789104	0.020472589	0.00862233	TNF/IL1B	2
2/108	26/18723	0.009789104	0.020472589	0.00862233	TNF/IFNG	2
2/108	26/18723	0.009789104	0.020472589	0.00862233	FADD/CAV1	2
2/108	26/18723	0.009789104	0.020472589	0.00862233	IL6/STAT3	2
2/108	26/18723	0.009789104	0.020472589	0.00862233	MAPK1/MAPK3	2
2/108	26/18723	0.009789104	0.020472589	0.00862233	MAPK1/MAPK3	2
3/108	78/18723	0.010381254	0.021686826	0.00913373	PTGS2/KDR/HM(	3
3/108	78/18723	0.010381254	0.021686826	0.00913373	HSD17B6/CYP3A	3
2/108	27/18723	0.010532746	0.021869429	0.00921063	IL6/HSF1	2
2/108	27/18723	0.010532746	0.021869429	0.00921063	IL4/NOS2	2
2/108	27/18723	0.010532746	0.021869429	0.00921063	TNF/HMOX1	2
2/108	27/18723	0.010532746	0.021869429	0.00921063	TNF/TP53	2
2/108	27/18723	0.010532746	0.021869429	0.00921063	RELA/NFKB1	2
2/108	27/18723	0.010532746	0.021869429	0.00921063	MAPK1/MAPK3	2
2/108	27/18723	0.010532746	0.021869429	0.00921063	BCL2/IRF1	2
2/108	27/18723	0.010532746	0.021869429	0.00921063	FADD/IGF2	2
2/108	27/18723	0.010532746	0.021869429	0.00921063	BCL2/NOS3	2
2/108	27/18723	0.010532746	0.021869429	0.00921063	ESR1/AR	2
2/108	27/18723	0.010532746	0.021869429	0.00921063	TLR4/PARP1	2
4/108	148/18723	0.010653244	0.022097361	0.00930663	TNF/CXCL8/BCI	4
7/108	418/18723	0.010654295	0.022097361	0.00930663	BCL2/SPP1/ERI	7
4/108	149/18723	0.010899575	0.022593596	0.00951563	HSPA5/EGFR/EI	4
3/108	80/18723	0.011119121	0.023035972	0.00970194	TP53/ESR1/PMI	3
2/108	28/18723	0.01130061	0.0232452	0.00979006	CXCL10/CXCL11	2
2/108	28/18723	0.01130061	0.0232452	0.00979006	NFKB1/APOB	2
2/108	28/18723	0.01130061	0.0232452	0.00979006	IL6/HSF1	2
2/108	28/18723	0.01130061	0.0232452	0.00979006	TLR4/STAT1	2
2/108	28/18723	0.01130061	0.0232452	0.00979006	TLR4/STAT1	2
2/108	28/18723	0.01130061	0.0232452	0.00979006	APOE/PTPN1	2
2/108	28/18723	0.01130061	0.0232452	0.00979006	IFNG/IRF1	2
2/108	28/18723	0.01130061	0.0232452	0.00979006	IL4/TLR4	2
2/108	28/18723	0.01130061	0.0232452	0.00979006	STAT3/MTOR	2
2/108	28/18723	0.01130061	0.0232452	0.00979006	TLR4/HMOX1	2
2/108	28/18723	0.01130061	0.0232452	0.00979006	NFE2L2/HSF1	2
2/108	28/18723	0.01130061	0.0232452	0.00979006	CXCL8/EDN1	2
2/108	28/18723	0.01130061	0.0232452	0.00979006	MAPK1/MAPK3	2
5/108	233/18723	0.011310463	0.023252727	0.00979323	KDR/BCL2/VCAM	5
4/108	151/18723	0.011403217	0.023430583	0.00986814	CXCL8/EGFR/I(	4
3/108	81/18723	0.011499145	0.023563205	0.00992399	EDN1/MAPK1/MA	3
3/108	81/18723	0.011499145	0.023563205	0.00992399	HSPA5/TP53/CA	3
3/108	81/18723	0.011499145	0.023563205	0.00992399	BCL2/MAPK1/MA	3
3/108	81/18723	0.011499145	0.023563205	0.00992399	TP53/ESR1/PMI	3
3/108	81/18723	0.011499145	0.023563205	0.00992399	BCL2/HSF1/BCI	3
5/108	234/18723	0.011505676	0.023563725	0.00992421	SPP1/ESR1/HSI	5
4/108	152/18723	0.011660567	0.023867921	0.01005233	IL6/IL4/TLR4/	4
3/108	82/18723	0.011886596	0.024254851	0.01021529	AKT1/IGF2/MAI	3

3/108	82/18723	0.011886596	0.024254851	0.01021529	ICAM1/NOS2/FA	3
3/108	82/18723	0.011886596	0.024254851	0.01021529	IL4/STAT3/PAF	3
3/108	82/18723	0.011886596	0.024254851	0.01021529	MTOR/MAPK1/CF	3
3/108	82/18723	0.011886596	0.024254851	0.01021529	PML/MAPK1/MAF	3
6/108	328/18723	0.011888366	0.024254851	0.01021529	EGFR/NFKB1/MA	6
2/108	29/18723	0.012092399	0.024471626	0.01030659	AKT1/GSK3B	2
2/108	29/18723	0.012092399	0.024471626	0.01030659	TP53/HK2	2
2/108	29/18723	0.012092399	0.024471626	0.01030659	APOB/CPS1	2
2/108	29/18723	0.012092399	0.024471626	0.01030659	AKT1/SOD1	2
2/108	29/18723	0.012092399	0.024471626	0.01030659	AKT1/SOD1	2
2/108	29/18723	0.012092399	0.024471626	0.01030659	IL6/BCL2	2
2/108	29/18723	0.012092399	0.024471626	0.01030659	BCL2/IGF2	2
2/108	29/18723	0.012092399	0.024471626	0.01030659	TP53/PML	2
2/108	29/18723	0.012092399	0.024471626	0.01030659	EGFR/AKT1	2
2/108	29/18723	0.012092399	0.024471626	0.01030659	CXCL10/SERPIN	2
2/108	29/18723	0.012092399	0.024471626	0.01030659	TP53/BCL2L1	2
2/108	29/18723	0.012092399	0.024471626	0.01030659	BCL2/BCL2L1	2
2/108	29/18723	0.012092399	0.024471626	0.01030659	BCL2/TP53	2
2/108	29/18723	0.012092399	0.024471626	0.01030659	HAP1/APOE	2
2/108	29/18723	0.012092399	0.024471626	0.01030659	CXCL10/FADD	2
4/108	154/18723	0.01218642	0.02464861	0.01038113	KDR/CCL2/ICAM	4
3/108	83/18723	0.012281495	0.024814172	0.01045086	AKT1/CAV1/MTO	3
3/108	83/18723	0.012281495	0.024814172	0.01045086	BCL2/SPP1/SOD	3
2/108	30/18723	0.012907816	0.025829393	0.01087843	SDHB/SDHA	2
2/108	30/18723	0.012907816	0.025829393	0.01087843	IL6/HSF1	2
2/108	30/18723	0.012907816	0.025829393	0.01087843	EGFR/SELE	2
2/108	30/18723	0.012907816	0.025829393	0.01087843	PAH/TYR	2
2/108	30/18723	0.012907816	0.025829393	0.01087843	TP53/HK2	2
2/108	30/18723	0.012907816	0.025829393	0.01087843	TP53/PML	2
2/108	30/18723	0.012907816	0.025829393	0.01087843	BAK1/CAV1	2
2/108	30/18723	0.012907816	0.025829393	0.01087843	APOE/APOB	2
2/108	30/18723	0.012907816	0.025829393	0.01087843	APOE/APOB	2
2/108	30/18723	0.012907816	0.025829393	0.01087843	IFNG/EDN1	2
2/108	30/18723	0.012907816	0.025829393	0.01087843	IFNG/EDN1	2
2/108	30/18723	0.012907816	0.025829393	0.01087843	CAT/CAV1	2
2/108	30/18723	0.012907816	0.025829393	0.01087843	IL4/HMOX1	2
2/108	30/18723	0.012907816	0.025829393	0.01087843	TNF/MTOR	2
2/108	30/18723	0.012907816	0.025829393	0.01087843	IL1B/GSK3B	2
2/108	30/18723	0.012907816	0.025829393	0.01087843	TP53/NFE2L2	2
2/108	30/18723	0.012907816	0.025829393	0.01087843	AKT1/MAPK1	2
2/108	30/18723	0.012907816	0.025829393	0.01087843	HSP90AA1/MAPK	2
4/108	157/18723	0.013003356	0.026006712	0.01095311	IL4/ERBB2/IRF	4
3/108	85/18723	0.013093728	0.026104013	0.01099409	BCL2/GATA3/ST	3
3/108	85/18723	0.013093728	0.026104013	0.01099409	IL4/STAT3/PAF	3
3/108	85/18723	0.013093728	0.026104013	0.01099409	HSPA5/NFE2L2/	3
3/108	85/18723	0.013093728	0.026104013	0.01099409	TNF/CXCL8/BCL	3
3/108	85/18723	0.013093728	0.026104013	0.01099409	IFNG/HMOX1/SO	3
3/108	85/18723	0.013093728	0.026104013	0.01099409	HSF1/PML/PARF	3
3/108	86/18723	0.013511102	0.026907522	0.0113325	EDN1/MAPK1/MA	3
3/108	86/18723	0.013511102	0.026907522	0.0113325	IL1B/AKT1/NFK	3
2/108	31/18723	0.013746568	0.027174626	0.011445	TP53/MAPK8	2
2/108	31/18723	0.013746568	0.027174626	0.011445	INSR/MTOR	2
2/108	31/18723	0.013746568	0.027174626	0.011445	IL4/HMOX1	2
2/108	31/18723	0.013746568	0.027174626	0.011445	TLR4/PARP1	2

2/108	31/18723	0.013746568	0.027174626	0.011445	PAH/SLC6A4	2
2/108	31/18723	0.013746568	0.027174626	0.011445	TP53/PML	2
2/108	31/18723	0.013746568	0.027174626	0.011445	IFNG/IRF1	2
2/108	31/18723	0.013746568	0.027174626	0.011445	PTGS2/EDN1	2
2/108	31/18723	0.013746568	0.027174626	0.011445	TNF/CXCL8	2
2/108	31/18723	0.013746568	0.027174626	0.011445	IL6/F2	2
2/108	31/18723	0.013746568	0.027174626	0.011445	MAPK14/MAPK1	2
2/108	31/18723	0.013746568	0.027174626	0.011445	EDN1/ABCG2	2
2/108	31/18723	0.013746568	0.027174626	0.011445	GATA3/STAT1	2
2/108	31/18723	0.013746568	0.027174626	0.011445	ICAM1/CAV1	2
3/108	87/18723	0.013936007	0.027520131	0.01159051	IL1B/EDN1/MT(	3
3/108	87/18723	0.013936007	0.027520131	0.01159051	INSR/ABCG2/AI	3
5/108	246/18723	0.014030109	0.027691394	0.01166264	TP53/NOS3/INS	5
3/108	88/18723	0.014368458	0.028329408	0.01193135	IL4/STAT3/PAI	3
3/108	88/18723	0.014368458	0.028329408	0.01193135	INSR/ABCG2/AI	3
7/108	444/18723	0.014450286	0.028475787	0.011993	EGFR/NFKB1/GA	7
2/108	32/18723	0.014608362	0.028601914	0.01204612	NOS3/GATA3	2
2/108	32/18723	0.014608362	0.028601914	0.01204612	IL6/HSF1	2
2/108	32/18723	0.014608362	0.028601914	0.01204612	BCL2/HMOX1	2
2/108	32/18723	0.014608362	0.028601914	0.01204612	NFE2L2/HSF1	2
2/108	32/18723	0.014608362	0.028601914	0.01204612	AKT1/SOD1	2
2/108	32/18723	0.014608362	0.028601914	0.01204612	APOE/APOB	2
2/108	32/18723	0.014608362	0.028601914	0.01204612	IRF1/STAT1	2
2/108	32/18723	0.014608362	0.028601914	0.01204612	AKT1/GSK3B	2
2/108	32/18723	0.014608362	0.028601914	0.01204612	CXCL8/EDN1	2
2/108	32/18723	0.014608362	0.028601914	0.01204612	IL1B/NCF1	2
2/108	32/18723	0.014608362	0.028601914	0.01204612	TNF/IL1B	2
2/108	32/18723	0.014608362	0.028601914	0.01204612	BCL2L1/PTPN1	2
7/108	445/18723	0.014613338	0.028601914	0.01204612	IL6/AKT1/NFE2	7
5/108	249/18723	0.014715065	0.02878601	0.01212365	CCL2/BCL2/TP5	5
4/108	163/18723	0.014740329	0.028820413	0.01213814	HSPA5/NFE2L2/	4
7/108	446/18723	0.014777705	0.028878451	0.01216259	EGFR/NFKB1/GA	7
3/108	89/18723	0.014808473	0.028923522	0.01218157	IL4/STAT3/PAI	3
3/108	90/18723	0.015256068	0.029735866	0.0125237	TNF/MTOR/GSK3	3
3/108	90/18723	0.015256068	0.029735866	0.0125237	IL4/TLR4/NFKI	3
3/108	90/18723	0.015256068	0.029735866	0.0125237	IL6/EGFR/SPP1	3
3/108	90/18723	0.015256068	0.029735866	0.0125237	NCF1/CAV1/PTI	3
2/108	33/18723	0.015492912	0.029995045	0.01263286	ICAM1/SOD1	2
2/108	33/18723	0.015492912	0.029995045	0.01263286	APRT/ATIC	2
2/108	33/18723	0.015492912	0.029995045	0.01263286	TP53/PML	2
2/108	33/18723	0.015492912	0.029995045	0.01263286	TP53/EDN1	2
2/108	33/18723	0.015492912	0.029995045	0.01263286	IL1B/PTPN1	2
2/108	33/18723	0.015492912	0.029995045	0.01263286	CD40LG/GATA3	2
2/108	33/18723	0.015492912	0.029995045	0.01263286	CD40LG/GATA3	2
2/108	33/18723	0.015492912	0.029995045	0.01263286	PTGS2/SOD1	2
2/108	33/18723	0.015492912	0.029995045	0.01263286	AKT1/MTOR	2
2/108	33/18723	0.015492912	0.029995045	0.01263286	HSF1/HMOX1	2
2/108	33/18723	0.015492912	0.029995045	0.01263286	TP53/ESR1	2
2/108	33/18723	0.015492912	0.029995045	0.01263286	HAP1/APOE	2
2/108	33/18723	0.015492912	0.029995045	0.01263286	EGFR/AKT1	2
6/108	348/18723	0.015516533	0.030025292	0.0126456	CCL2/ERBB2/I(	6
7/108	451/18723	0.01561946	0.030208888	0.01272292	IL6/AKT1/HSF1	7
3/108	91/18723	0.015711255	0.030292784	0.01275825	BCL2/CAT/GAT/	3
3/108	91/18723	0.015711255	0.030292784	0.01275825	IFNG/IL4/HMO)	3

3/108	91/18723	0.015711255	0.030292784	0.01275825	EDN1/MAPK1/MA	3
3/108	91/18723	0.015711255	0.030292784	0.01275825	IFNG/IL4/GATA	3
3/108	91/18723	0.015711255	0.030292784	0.01275825	EGFR/SELE/ESR	3
3/108	91/18723	0.015711255	0.030292784	0.01275825	BCL2/GATA3/ST	3
6/108	350/18723	0.015917263	0.030674233	0.01291891	TNF/IL6/TLR4/	6
3/108	92/18723	0.016174048	0.031089286	0.01309371	TNF/STAT1/PML	3
3/108	92/18723	0.016174048	0.031089286	0.01309371	CCL2/AKT1/VDA	3
3/108	92/18723	0.016174048	0.031089286	0.01309371	BCL2/CAT/GATA	3
3/108	92/18723	0.016174048	0.031089286	0.01309371	BCL2/CAT/GATA	3
3/108	92/18723	0.016174048	0.031089286	0.01309371	MAPK1/MAPK3/C	3
2/108	34/18723	0.016399931	0.031330962	0.0131955	IL6/F2	2
2/108	34/18723	0.016399931	0.031330962	0.0131955	ERBB2/MTOR	2
2/108	34/18723	0.016399931	0.031330962	0.0131955	MTOR/APOE	2
2/108	34/18723	0.016399931	0.031330962	0.0131955	TP53/MTOR	2
2/108	34/18723	0.016399931	0.031330962	0.0131955	TP53/PML	2
2/108	34/18723	0.016399931	0.031330962	0.0131955	IFNG/MTOR	2
2/108	34/18723	0.016399931	0.031330962	0.0131955	TP53/HK2	2
2/108	34/18723	0.016399931	0.031330962	0.0131955	MAPK1/MAPK3	2
2/108	34/18723	0.016399931	0.031330962	0.0131955	IL1B/IL1A	2
2/108	34/18723	0.016399931	0.031330962	0.0131955	TNF/GSK3B	2
2/108	34/18723	0.016399931	0.031330962	0.0131955	BCL2/FADD	2
2/108	34/18723	0.016399931	0.031330962	0.0131955	TP53/FADD	2
4/108	169/18723	0.016617724	0.031730894	0.01336394	PTGS2/EDN1/CA	4
3/108	93/18723	0.016644459	0.031733519	0.01336504	BCL2/GATA3/ST	3
3/108	93/18723	0.016644459	0.031733519	0.01336504	INSR/APOE/GSK	3
3/108	93/18723	0.016644459	0.031733519	0.01336504	MAPK1/MAPK3/C	3
4/108	170/18723	0.016944509	0.032289181	0.01359907	MAPK14/CAV1/A	4
3/108	94/18723	0.0171225	0.032595266	0.01372798	EGFR/AKT1/CAS	3
3/108	94/18723	0.0171225	0.032595266	0.01372798	BCL2/GATA3/ST	3
2/108	35/18723	0.017329134	0.032805633	0.01381658	KDR/EDN1	2
2/108	35/18723	0.017329134	0.032805633	0.01381658	EDN1/MTOR	2
2/108	35/18723	0.017329134	0.032805633	0.01381658	EDN1/MTOR	2
2/108	35/18723	0.017329134	0.032805633	0.01381658	IFNG/PARP1	2
2/108	35/18723	0.017329134	0.032805633	0.01381658	NFKB1/GATA3	2
2/108	35/18723	0.017329134	0.032805633	0.01381658	AR/PARP1	2
2/108	35/18723	0.017329134	0.032805633	0.01381658	IL6/PTGS2	2
2/108	35/18723	0.017329134	0.032805633	0.01381658	TNF/INSR	2
2/108	35/18723	0.017329134	0.032805633	0.01381658	EDN1/MTOR	2
2/108	35/18723	0.017329134	0.032805633	0.01381658	MTOR/CPS1	2
2/108	35/18723	0.017329134	0.032805633	0.01381658	CXCL10/FADD	2
4/108	172/18723	0.017610109	0.033287184	0.01401939	RAF1/HMGCR/IC	4
4/108	172/18723	0.017610109	0.033287184	0.01401939	STAT3/AKT1/NC	4
4/108	172/18723	0.017610109	0.033287184	0.01401939	TP53/PML/MAPK	4
7/108	463/18723	0.017778421	0.03358842	0.01414626	IL1B/PADI4/TF	7
3/108	96/18723	0.018101507	0.034164434	0.01438886	BCL2/CAT/GATA	3
3/108	96/18723	0.018101507	0.034164434	0.01438886	NOS3/EDN1/AR	3
2/108	36/18723	0.018280242	0.03434636	0.01446548	TNF/GSK3B	2
2/108	36/18723	0.018280242	0.03434636	0.01446548	FASN/JUN	2
2/108	36/18723	0.018280242	0.03434636	0.01446548	STAT3/MTOR	2
2/108	36/18723	0.018280242	0.03434636	0.01446548	AKT1/NFKBIA	2
2/108	36/18723	0.018280242	0.03434636	0.01446548	NFKB1/CAV1	2
2/108	36/18723	0.018280242	0.03434636	0.01446548	EDN1/PARP1	2
2/108	36/18723	0.018280242	0.03434636	0.01446548	FADD/PTPN1	2
2/108	36/18723	0.018280242	0.03434636	0.01446548	TP53/ESR1	2

2/108	36/18723	0.018280242	0.03434636	0.01446548	BCL2/BCL2L1	2
7/108	467/18723	0.01854279	0.034822229	0.0146659	IL6/PAH/AKT1/	7
6/108	363/18723	0.018698436	0.035096965	0.01478161	KDR/BCL2/VCAM	6
3/108	98/18723	0.019111139	0.035853681	0.01510031	EGFR/AKT1/CAS	3
2/108	37/18723	0.019252975	0.035975941	0.0151518	NOS3/EDN1	2
2/108	37/18723	0.019252975	0.035975941	0.0151518	NOS3/GATA3	2
2/108	37/18723	0.019252975	0.035975941	0.0151518	RELA/CAV1	2
2/108	37/18723	0.019252975	0.035975941	0.0151518	PAH/GATA3	2
2/108	37/18723	0.019252975	0.035975941	0.0151518	NCF1/INSR	2
2/108	37/18723	0.019252975	0.035975941	0.0151518	AKT1/AR	2
2/108	37/18723	0.019252975	0.035975941	0.0151518	MAPK1/MAPK3	2
2/108	37/18723	0.019252975	0.035975941	0.0151518	GATA3/AR	2
5/108	267/18723	0.019299551	0.036045032	0.0151809	IFNG/CCL2/HAF	5
4/108	177/18723	0.019344914	0.036111789	0.01520901	TNF/EGFR/MAPK	4
3/108	99/18723	0.019627456	0.036584643	0.01540816	EDN1/MTOR/PAF	3
3/108	99/18723	0.019627456	0.036584643	0.01540816	STAT3/GLS/MTC	3
3/108	99/18723	0.019627456	0.036584643	0.01540816	MAPK1/APOE/GS	3
4/108	179/18723	0.020067427	0.037386164	0.01574574	CCL2/BCL2/TP5	4
2/108	38/18723	0.020247056	0.037608832	0.01583952	IL4/NOS2	2
2/108	38/18723	0.020247056	0.037608832	0.01583952	CYP3A4/CYP19A	2
2/108	38/18723	0.020247056	0.037608832	0.01583952	PAH/GATA3	2
2/108	38/18723	0.020247056	0.037608832	0.01583952	EDN1/MTOR	2
2/108	38/18723	0.020247056	0.037608832	0.01583952	RELA/STAT3	2
2/108	38/18723	0.020247056	0.037608832	0.01583952	TP53/CERS1	2
3/108	101/18723	0.020683119	0.038399817	0.01617265	IFNG/TP53/MAF	3
4/108	181/18723	0.020806422	0.038609643	0.01626102	TNF/EGFR/MAPK	4
3/108	102/18723	0.021222473	0.039261362	0.0165355	EDN1/MTOR/PAF	3
3/108	102/18723	0.021222473	0.039261362	0.0165355	AKT1/VDAC1/SI	3
2/108	39/18723	0.021262213	0.039261362	0.0165355	TNF/HMOX1	2
2/108	39/18723	0.021262213	0.039261362	0.0165355	MAPK1/MAPK3	2
2/108	39/18723	0.021262213	0.039261362	0.0165355	IL1B/MAPK3	2
2/108	39/18723	0.021262213	0.039261362	0.0165355	IL1B/PTGS2	2
2/108	39/18723	0.021262213	0.039261362	0.0165355	APOB/CPS1	2
2/108	39/18723	0.021262213	0.039261362	0.0165355	APOB/CPS1	2
2/108	39/18723	0.021262213	0.039261362	0.0165355	EDN1/MTOR	2
2/108	39/18723	0.021262213	0.039261362	0.0165355	VDR/BAK1	2
3/108	103/18723	0.021769511	0.04017834	0.0169217	AKT1/MAPK14/M	3
2/108	40/18723	0.022298174	0.040998845	0.01726727	TNF/IL4	2
2/108	40/18723	0.022298174	0.040998845	0.01726727	TNF/IL4	2
2/108	40/18723	0.022298174	0.040998845	0.01726727	IL1B/EDN1	2
2/108	40/18723	0.022298174	0.040998845	0.01726727	MAPK1/MAPK3	2
2/108	40/18723	0.022298174	0.040998845	0.01726727	TP53/HK2	2
2/108	40/18723	0.022298174	0.040998845	0.01726727	GLS/CPS1	2
3/108	104/18723	0.022324236	0.040998845	0.01726727	ESR1/F2/EDN1	3
3/108	104/18723	0.022324236	0.040998845	0.01726727	EDN1/MTOR/PAF	3
3/108	104/18723	0.022324236	0.040998845	0.01726727	IFNG/IL4/GATA	3
4/108	185/18723	0.022334214	0.040998845	0.01726727	PTGS2/KDR/AKT	4
4/108	185/18723	0.022334214	0.040998845	0.01726727	CAT/ESR1/NFKE	4
4/108	186/18723	0.022726599	0.041698755	0.01756205	HAP1/ICAM1/CA	4
3/108	105/18723	0.022886648	0.041971899	0.01767709	EGFR/PARP1/PC	3
4/108	187/18723	0.023123179	0.042384968	0.01785106	PML/MAPK1/MAF	4
2/108	41/18723	0.02335467	0.042684241	0.0179771	APRT/ATIC	2
2/108	41/18723	0.02335467	0.042684241	0.0179771	PTGS2/CASP3	2
2/108	41/18723	0.02335467	0.042684241	0.0179771	CXCL8/EDN1	2

2/108	41/18723	0.02335467	0.042684241	0.0179771	NOS3/GATA3	2
2/108	41/18723	0.02335467	0.042684241	0.0179771	MAPK1/MAPK3	2
2/108	41/18723	0.02335467	0.042684241	0.0179771	TNF/AKT1	2
7/108	490/18723	0.023392345	0.042732293	0.01799734	HSPA5/AKT1/NF	7
5/108	281/18723	0.023453383	0.042808279	0.01802934	TNF/BCL2/MTOR	5
3/108	106/18723	0.023456748	0.042808279	0.01802934	EGFR/NFKB1/CA	3
4/108	188/18723	0.023523964	0.042910088	0.01807222	HAP1/ICAM1/CA	4
4/108	189/18723	0.023928963	0.043627648	0.01837443	CAT/AKT1/SDHE	4
3/108	107/18723	0.024034533	0.043735161	0.01841971	HAP1/CASP3/GS	3
3/108	107/18723	0.024034533	0.043735161	0.01841971	HSPA5/NFE2L2/	3
3/108	107/18723	0.024034533	0.043735161	0.01841971	MAPK1/MAPK3/C	3
3/108	107/18723	0.024034533	0.043735161	0.01841971	MAPK1/MAPK3/C	3
4/108	190/18723	0.024338185	0.044178999	0.01860664	PLAT/AR/BCL2L	4
4/108	190/18723	0.024338185	0.044178999	0.01860664	ESR2/ESR1/AR/	4
2/108	42/18723	0.024431434	0.044178999	0.01860664	APRT/ATIC	2
2/108	42/18723	0.024431434	0.044178999	0.01860664	STAT3/GSK3B	2
2/108	42/18723	0.024431434	0.044178999	0.01860664	NFE2L2/SERPIN	2
2/108	42/18723	0.024431434	0.044178999	0.01860664	TLR4/GATA3	2
2/108	42/18723	0.024431434	0.044178999	0.01860664	IL6/F2	2
2/108	42/18723	0.024431434	0.044178999	0.01860664	IL4/HMOX1	2
2/108	42/18723	0.024431434	0.044178999	0.01860664	RELA/MTOR	2
2/108	42/18723	0.024431434	0.044178999	0.01860664	HMGCR/APOE	2
2/108	42/18723	0.024431434	0.044178999	0.01860664	MTOR/APOE	2
2/108	42/18723	0.024431434	0.044178999	0.01860664	HMGCR/APOE	2
2/108	42/18723	0.024431434	0.044178999	0.01860664	CXCL10/FADD	2
3/108	108/18723	0.024620003	0.044498552	0.01874122	AKT1/MAPK14/M	3
5/108	285/18723	0.024738518	0.04469124	0.01882238	GATA3/EDN1/MA	5
3/108	109/18723	0.025213155	0.045504897	0.01916506	IFNG/CCL2/HAF	3
3/108	109/18723	0.025213155	0.045504897	0.01916506	BCL2/GATA3/ST	3
5/108	287/18723	0.025397788	0.045816097	0.01929613	IL1B/INSR/IL1	5
2/108	43/18723	0.025528201	0.045918959	0.01933945	TNF/MAPK14	2
2/108	43/18723	0.025528201	0.045918959	0.01933945	TLR4/NFKBIA	2
2/108	43/18723	0.025528201	0.045918959	0.01933945	NOS3/NOS2	2
2/108	43/18723	0.025528201	0.045918959	0.01933945	MAPK1/MAPK3	2
2/108	43/18723	0.025528201	0.045918959	0.01933945	HSD17B6/CYP19	2
2/108	43/18723	0.025528201	0.045918959	0.01933945	IL1B/EDN1	2
3/108	110/18723	0.025813986	0.046410777	0.01954659	SPP1/APOE/GSK	3
4/108	194/18723	0.026017475	0.046754237	0.01969124	IL4/AHR/MAPK1	4
3/108	111/18723	0.026422491	0.047413972	0.0199691	MAPK1/MAPK3/C	3
3/108	111/18723	0.026422491	0.047413972	0.0199691	MAPK1/MAPK3/C	3
3/108	111/18723	0.026422491	0.047413972	0.0199691	IL4/GATA3/PTF	3
2/108	44/18723	0.02664471	0.047675998	0.02007945	HMGCR/MTOR	2
2/108	44/18723	0.02664471	0.047675998	0.02007945	APRT/ATIC	2
2/108	44/18723	0.02664471	0.047675998	0.02007945	BCL2/TYR	2
2/108	44/18723	0.02664471	0.047675998	0.02007945	AKT1/MAPK1	2
2/108	44/18723	0.02664471	0.047675998	0.02007945	TLR4/CAV1	2
2/108	44/18723	0.02664471	0.047675998	0.02007945	ICAM1/CAV1	2
4/108	196/18723	0.026882675	0.048078876	0.02024913	BCL2/MTOR/SLC	4
3/108	112/18723	0.027038665	0.048265881	0.02032789	PTGS2/CYP3A4/	3
3/108	112/18723	0.027038665	0.048265881	0.02032789	IL4/STAT3/PAF	3
3/108	112/18723	0.027038665	0.048265881	0.02032789	TNF/IL4/APOE	3
3/108	112/18723	0.027038665	0.048265881	0.02032789	IFNG/TP53/MAF	3
4/108	198/18723	0.027764991	0.049355776	0.02078691	PTGS2/MAPK1/A	4
2/108	45/18723	0.027780702	0.049355776	0.02078691	TNF/IL1B	2

2/108	45/18723	0.027780702	0.049355776	0.02078691	ESR1/AR	2
2/108	45/18723	0.027780702	0.049355776	0.02078691	VDR/HMOX1	2
2/108	45/18723	0.027780702	0.049355776	0.02078691	TNF/IGF2	2
2/108	45/18723	0.027780702	0.049355776	0.02078691	TNF/GSK3B	2
2/108	45/18723	0.027780702	0.049355776	0.02078691	MMP9/MMP2	2
2/108	45/18723	0.027780702	0.049355776	0.02078691	BCL2/MTOR	2
2/108	45/18723	0.027780702	0.049355776	0.02078691	PTGS2/EDN1	2
2/108	45/18723	0.027780702	0.049355776	0.02078691	GC/ABCG2	2
5/108	294/18723	0.027793905	0.049355875	0.02078696	CCL2/BCL2/TP5	5
3/108	115/18723	0.028933142	0.051190119	0.02155948	TP53/PML/MAPK	3
3/108	115/18723	0.028933142	0.051190119	0.02155948	IFNG/HMOX1/SC	3
2/108	46/18723	0.028935917	0.051190119	0.02155948	BAK1/NOS3	2
2/108	46/18723	0.028935917	0.051190119	0.02155948	MTOR/AR	2
2/108	46/18723	0.028935917	0.051190119	0.02155948	NFE2L2/ATIC	2
2/108	46/18723	0.028935917	0.051190119	0.02155948	IL4/HMOX1	2
2/108	46/18723	0.028935917	0.051190119	0.02155948	EGFR/AKT1	2
2/108	46/18723	0.028935917	0.051190119	0.02155948	APOE/GSK3B	2
4/108	201/18723	0.02912068	0.051492714	0.02168692	IL1B/GATA3/MA	4
5/108	298/18723	0.029225687	0.051654062	0.02175487	BCL2/TP53/HSP	5
3/108	116/18723	0.029579929	0.052230976	0.02199785	EGFR/HMGCR/CY	3
3/108	116/18723	0.029579929	0.052230976	0.02199785	IFNG/TP53/MAP	3
2/108	47/18723	0.030110102	0.052844004	0.02225603	NOS3/EDN1	2
2/108	47/18723	0.030110102	0.052844004	0.02225603	IL6/F2	2
2/108	47/18723	0.030110102	0.052844004	0.02225603	AKT1/PML	2
2/108	47/18723	0.030110102	0.052844004	0.02225603	TLR4/BCL2	2
2/108	47/18723	0.030110102	0.052844004	0.02225603	AKT1/MTOR	2
2/108	47/18723	0.030110102	0.052844004	0.02225603	AKT1/NFKB1	2
2/108	47/18723	0.030110102	0.052844004	0.02225603	TNF/IL1B	2
2/108	47/18723	0.030110102	0.052844004	0.02225603	STAT3/NOS3	2
2/108	47/18723	0.030110102	0.052844004	0.02225603	IL4/PARP1	2
2/108	47/18723	0.030110102	0.052844004	0.02225603	VCAM1/GATA3	2
2/108	47/18723	0.030110102	0.052844004	0.02225603	TP53/MTOR	2
2/108	47/18723	0.030110102	0.052844004	0.02225603	IL1B/NCF1	2
2/108	47/18723	0.030110102	0.052844004	0.02225603	AKT1/VDAC1	2
4/108	204/18723	0.030515172	0.053529886	0.0225449	TNF/IL1B/KDR/	4
3/108	118/18723	0.030896393	0.05414802	0.02280524	PAH/VCAM1/GAT	3
3/108	118/18723	0.030896393	0.05414802	0.02280524	IL1B/AKT1/NFK	3
5/108	303/18723	0.031080164	0.054444673	0.02293018	IL1B/AKT1/EDN	5
2/108	48/18723	0.031303004	0.054758377	0.0230623	IL4/HMOX1	2
2/108	48/18723	0.031303004	0.054758377	0.0230623	ESR1/PML	2
2/108	48/18723	0.031303004	0.054758377	0.0230623	APOE/APOB	2
6/108	412/18723	0.032142585	0.056200868	0.02366983	HSPA5/AKT1/NF	6
3/108	120/18723	0.032243311	0.056324519	0.0237219	IFNG/CCL2/HAF	3
3/108	120/18723	0.032243311	0.056324519	0.0237219	HSP90AA1/APOE	3
2/108	49/18723	0.032514372	0.056561145	0.02382156	IL4/CD40LG	2
2/108	49/18723	0.032514372	0.056561145	0.02382156	IL4/CD40LG	2
2/108	49/18723	0.032514372	0.056561145	0.02382156	IL4/HMOX1	2
2/108	49/18723	0.032514372	0.056561145	0.02382156	IL4/TLR4	2
2/108	49/18723	0.032514372	0.056561145	0.02382156	TP53/MTOR	2
2/108	49/18723	0.032514372	0.056561145	0.02382156	IL1B/PTGS2	2
2/108	49/18723	0.032514372	0.056561145	0.02382156	IL4/CD40LG	2
2/108	49/18723	0.032514372	0.056561145	0.02382156	CXCL8/CAV1	2
2/108	49/18723	0.032514372	0.056561145	0.02382156	IL1B/GATA3	2
4/108	209/18723	0.032925953	0.057227929	0.02410239	ICAM1/EDN1/MT	4

3/108	121/18723	0.032928164	0.057227929	0.02410239	EGFR/AKT1/HSI	3
2/108	50/18723	0.033743957	0.058510306	0.02464248	FADD/EDN1	2
2/108	50/18723	0.033743957	0.058510306	0.02464248	IL4/HMOX1	2
2/108	50/18723	0.033743957	0.058510306	0.02464248	IL6/IL4	2
2/108	50/18723	0.033743957	0.058510306	0.02464248	HMGCR/MTOR	2
2/108	50/18723	0.033743957	0.058510306	0.02464248	HAP1/SOD1	2
6/108	417/18723	0.033795049	0.058571844	0.0246684	TNF/IFNG/BCL2	6
3/108	123/18723	0.034320594	0.059427818	0.0250289	TP53/PML/MAPK	3
3/108	123/18723	0.034320594	0.059427818	0.0250289	RELA/GATA3/MA	3
4/108	212/18723	0.034424595	0.059580419	0.02509317	RAF1/HMGCR/IC	4
2/108	51/18723	0.034991513	0.060394547	0.02543606	IL6/F2	2
2/108	51/18723	0.034991513	0.060394547	0.02543606	ESR1/STAT1	2
2/108	51/18723	0.034991513	0.060394547	0.02543606	RAF1/SOD1	2
2/108	51/18723	0.034991513	0.060394547	0.02543606	MMP9/MMP2	2
2/108	51/18723	0.034991513	0.060394547	0.02543606	NOS3/NOS2	2
2/108	51/18723	0.034991513	0.060394547	0.02543606	MAPK14/MAPK1	2
5/108	314/18723	0.035416529	0.061100023	0.02573318	HSPA5/HAP1/NC	5
4/108	214/18723	0.03544549	0.061121898	0.02574239	AKT1/MTOR/APC	4
5/108	315/18723	0.035828405	0.061753825	0.02600854	RELA/BCL2/GAT	5
6/108	424/18723	0.036199967	0.062320519	0.02624721	CCL2/EGFR/BCL	6
2/108	52/18723	0.036256796	0.062320519	0.02624721	TLR4/HSP90AA1	2
2/108	52/18723	0.036256796	0.062320519	0.02624721	BCL2/SPP1	2
2/108	52/18723	0.036256796	0.062320519	0.02624721	MTOR/SLC6A4	2
2/108	52/18723	0.036256796	0.062320519	0.02624721	RAF1/SOD1	2
2/108	52/18723	0.036256796	0.062320519	0.02624721	TLR4/STAT3	2
3/108	126/18723	0.036465852	0.062651172	0.02638647	AKT1/GATA3/MT	3
4/108	216/18723	0.036483855	0.062653427	0.02638742	TNF/IL6/IL4/C	4
3/108	127/18723	0.037195977	0.063847142	0.02689017	IL1B/HMGCR/AI	3
2/108	53/18723	0.037539565	0.064319272	0.02708901	RELA/PML	2
2/108	53/18723	0.037539565	0.064319272	0.02708901	MAPK1/MAPK3	2
2/108	53/18723	0.037539565	0.064319272	0.02708901	AKT1/NFE2L2	2
2/108	53/18723	0.037539565	0.064319272	0.02708901	HSPA5/AKT1	2
3/108	128/18723	0.037933596	0.064964744	0.02736086	HSPA5/TP53/CA	3
3/108	129/18723	0.038678691	0.066210582	0.02788557	IL1B/AKT1/NFK	3
2/108	54/18723	0.038839579	0.066274445	0.02791246	MMP9/MMP2	2
2/108	54/18723	0.038839579	0.066274445	0.02791246	IL4/AHR	2
2/108	54/18723	0.038839579	0.066274445	0.02791246	PAH/GATA3	2
2/108	54/18723	0.038839579	0.066274445	0.02791246	RELA/NCF1	2
2/108	54/18723	0.038839579	0.066274445	0.02791246	PAH/GATA3	2
2/108	54/18723	0.038839579	0.066274445	0.02791246	MTOR/SLC6A4	2
2/108	54/18723	0.038839579	0.066274445	0.02791246	TP53/FADD	2
3/108	130/18723	0.039431247	0.067253476	0.0283248	TP53/ESR1/JUN	3
2/108	55/18723	0.040156601	0.068332397	0.0287792	ERBB2/MTOR	2
2/108	55/18723	0.040156601	0.068332397	0.0287792	NCF1/PTPN1	2
3/108	131/18723	0.040191245	0.068332397	0.0287792	HSPA5/NCF1/EI	3
3/108	131/18723	0.040191245	0.068332397	0.0287792	RAF1/PADI4/ST	3
3/108	131/18723	0.040191245	0.068332397	0.0287792	TLR4/HSP90AA1	3
3/108	131/18723	0.040191245	0.068332397	0.0287792	HSPA5/TP53/CA	3
3/108	131/18723	0.040191245	0.068332397	0.0287792	IL1B/HMGCR/AI	3
2/108	56/18723	0.041490396	0.07031826	0.02961558	NOS2/FADD	2
2/108	56/18723	0.041490396	0.07031826	0.02961558	SELE/INSR	2
2/108	56/18723	0.041490396	0.07031826	0.02961558	IL4/HMOX1	2
2/108	56/18723	0.041490396	0.07031826	0.02961558	MAPK14/MTOR	2
2/108	56/18723	0.041490396	0.07031826	0.02961558	IL1A/EDN1	2

2/108	56/18723	0.041490396	0.07031826	0.02961558	TP53/MTOR	2
2/108	56/18723	0.041490396	0.07031826	0.02961558	HSPA5/AKT1	2
3/108	134/18723	0.042515708	0.071958507	0.03030639	CXCL8/F2/EDN1	3
3/108	134/18723	0.042515708	0.071958507	0.03030639	IL4/INSR/MTOF	3
3/108	134/18723	0.042515708	0.071958507	0.03030639	RAF1/PADI4/ST	3
2/108	57/18723	0.04284073	0.072443288	0.03051056	IL1B/GSK3B	2
2/108	57/18723	0.04284073	0.072443288	0.03051056	IL4/CD40LG	2
4/108	229/18723	0.043660018	0.073795456	0.03108005	BCL2/MMP2/GAT	4
3/108	136/18723	0.044102215	0.074509322	0.0313807	IL4/HMOX1/GSK	3
2/108	58/18723	0.044207372	0.074519296	0.0313849	KDR/GSK3B	2
2/108	58/18723	0.044207372	0.074519296	0.0313849	APRT/ATIC	2
2/108	58/18723	0.044207372	0.074519296	0.0313849	SOAT1/BCL2	2
2/108	58/18723	0.044207372	0.074519296	0.0313849	EGFR/AKT1	2
2/108	58/18723	0.044207372	0.074519296	0.0313849	STAT3/NFE2L2	2
3/108	137/18723	0.044906466	0.075663768	0.03186691	TLR4/EGFR/IL1	3
2/108	59/18723	0.045590092	0.07667796	0.03229406	IL4/AHR	2
2/108	59/18723	0.045590092	0.07667796	0.03229406	PTGS2/MAPK3	2
2/108	59/18723	0.045590092	0.07667796	0.03229406	IFNG/MTOR	2
2/108	59/18723	0.045590092	0.07667796	0.03229406	MMP9/CAV1	2
5/108	337/18723	0.045645836	0.076737335	0.03231906	RAF1/TP53/JUN	5
3/108	138/18723	0.045718022	0.076789912	0.03234121	HSP90AA1/ICAM	3
3/108	138/18723	0.045718022	0.076789912	0.03234121	IFNG/HMOX1/SO	3
5/108	339/18723	0.046610672	0.078254232	0.03295793	FASN/NFE2L2/C	5
2/108	60/18723	0.046988665	0.078677719	0.03313629	TNF/IL4	2
2/108	60/18723	0.046988665	0.078677719	0.03313629	TNF/IL4	2
2/108	60/18723	0.046988665	0.078677719	0.03313629	TLR4/BAK1	2
2/108	60/18723	0.046988665	0.078677719	0.03313629	BCL2/SPP1	2
2/108	60/18723	0.046988665	0.078677719	0.03313629	JUN/APOE	2
2/108	60/18723	0.046988665	0.078677719	0.03313629	MAPK14/MAPK1	2
3/108	140/18723	0.047362957	0.079269078	0.03338534	EGFR/NFKB1/CA	3
4/108	236/18723	0.047830743	0.080016314	0.03370005	MAPK14/EDN1/M	4
2/108	61/18723	0.048402863	0.080901313	0.03407278	INSR/APOE	2
2/108	61/18723	0.048402863	0.080901313	0.03407278	MTOR/APOE	2
3/108	142/18723	0.04903684	0.081924477	0.03450371	BCL2/GATA3/ST	3
2/108	62/18723	0.049832465	0.083105764	0.03500122	APRT/ATIC	2
2/108	62/18723	0.049832465	0.083105764	0.03500122	IL4/HMOX1	2
2/108	62/18723	0.049832465	0.083105764	0.03500122	APRT/TYR	2
2/108	62/18723	0.049832465	0.083105764	0.03500122	BCL2/GATA3	2
3/108	143/18723	0.049884579	0.083155733	0.03502227	GATA3/HSP90AA	3
18/108	335/19550	3.55E-13	4.17E-11	3.10E-11	TNF/PTGS2/KDF	18
18/108	335/19550	3.55E-13	4.17E-11	3.10E-11	TNF/PTGS2/KDF	18
16/108	421/19550	1.34E-09	1.05E-07	7.82E-08	TNF/CXCL10/TH	16
8/108	84/19550	2.05E-08	1.21E-06	8.97E-07	PTGS2/SELE/NO	8
14/108	394/19550	3.63E-08	1.71E-06	1.27E-06	KDR/TLR4/EGFF	14
8/108	116/19550	2.59E-07	1.01E-05	7.53E-06	PTGS2/SELE/NO	8
10/108	230/19550	5.91E-07	1.88E-05	1.40E-05	RAF1/PTGS2/BA	10
10/108	232/19550	6.40E-07	1.88E-05	1.40E-05	RAF1/PTGS2/BA	10
11/108	313/19550	1.29E-06	3.37E-05	2.51E-05	IL6/PTGS2/HSF	11
11/108	327/19550	1.97E-06	4.39E-05	3.26E-05	EGFR/CAT/NFKE	11
9/108	205/19550	2.05E-06	4.39E-05	3.26E-05	RAF1/BAK1/BCI	9
4/108	26/19550	1.20E-05	0.00023479	0.00017458	BAK1/BCL2/VDA	4
9/108	322/19550	7.48E-05	0.001347109	0.00100166	CAT/NFKB1/APF	9
9/108	325/19550	8.03E-05	0.001347109	0.00100166	CAT/NFKB1/APF	9
3/108	18/19550	0.000125943	0.001973109	0.00146713	RAF1/MAPK1/MA	3

3/108	23/19550	0.000267899	0.003934769	0.00292574	HSP90AA1/APOE	3
8/108	336/19550	0.000555501	0.007678978	0.00570979	EGFR/NCF1/NOS	8
5/108	124/19550	0.000626635	0.008181066	0.00608312	CAT/MMP9/HSP9	5
9/108	480/19550	0.001373626	0.016989588	0.0126328	TP53/GLS/VDAC	9
3/108	44/19550	0.001841675	0.020609216	0.01532421	VDAC1/SLC25A5	3
3/108	44/19550	0.001841675	0.020609216	0.01532421	VDAC1/SLC25A5	3
8/108	418/19550	0.002236353	0.021487248	0.01597708	HSPA5/EGFR/CA	8
2/108	13/19550	0.002266552	0.021487248	0.01597708	APOE/APOB	2
2/108	13/19550	0.002266552	0.021487248	0.01597708	APOE/APOB	2
3/108	48/19550	0.002366992	0.021487248	0.01597708	BCL2/ERBB2/HS	3
8/108	425/19550	0.00247743	0.021487248	0.01597708	HSPA5/EGFR/CA	8
8/108	425/19550	0.00247743	0.021487248	0.01597708	MMP9/MMP2/ICA	8
4/108	103/19550	0.002560183	0.021487248	0.01597708	TP53/HSF1/PML	4
7/108	338/19550	0.002724476	0.022077647	0.01641608	RELA/PLAT/STA	7
4/108	109/19550	0.003141242	0.023812643	0.01770615	HSPA5/FASN/HS	4
4/108	109/19550	0.003141242	0.023812643	0.01770615	HSPA5/FASN/HS	4
5/108	185/19550	0.00365235	0.026821944	0.01994375	CAT/MMP9/HSP9	5
5/108	193/19550	0.004369843	0.03068149	0.02281356	EGFR/NOS3/CAV	5
8/108	468/19550	0.004439024	0.03068149	0.02281356	PTGS2/EGFR/BC	8
2/108	20/19550	0.005383683	0.035143483	0.02613132	APOE/APOB	2
2/108	20/19550	0.005383683	0.035143483	0.02613132	APOE/APOB	2
3/108	67/19550	0.00607782	0.038602367	0.02870322	SERPINE1/PLG/	3
3/108	72/19550	0.007417829	0.044865656	0.03336035	EGFR/APOE/APO	3
2/108	24/19550	0.007708947	0.044865656	0.03336035	F3/F10	2
4/108	142/19550	0.007983127	0.044865656	0.03336035	CAT/HMGCR/NOS	4
4/108	142/19550	0.007983127	0.044865656	0.03336035	CAT/HMGCR/NOS	4
7/108	413/19550	0.008018543	0.044865656	0.03336035	VDR/RELA/STAT	7
4/108	146/19550	0.008783892	0.04800499	0.03569464	GC/F2/PLG/APO	4
2/108	27/19550	0.00969885	0.051800677	0.03851696	APOE/APOB	2
2/108	28/19550	0.010407553	0.054350556	0.04041296	TNF/TLR4	2
16/108	271/18368	4.90E-12	2.08E-09	1.43E-09	TNF/IFNG/IL1B	16
13/108	196/18368	1.32E-10	2.81E-08	1.93E-08	EGFR/BCL2/STA	13
15/108	299/18368	2.37E-10	3.35E-08	2.30E-08	VDR/RELA/STAT	15
13/108	235/18368	1.24E-09	9.23E-08	6.33E-08	TNF/IFNG/IL1B	13
16/108	394/18368	1.25E-09	9.23E-08	6.33E-08	VDR/RELA/BCL2	16
11/108	150/18368	1.30E-09	9.23E-08	6.33E-08	EGFR/BCL2/STA	11
9/108	100/18368	7.30E-09	4.43E-07	3.04E-07	SOAT1/VDR/ESR	9
6/108	32/18368	2.87E-08	1.53E-06	1.05E-06	BCL2/TP53/AKT	6
15/108	487/18368	1.66E-07	7.82E-06	5.36E-06	TNF/IFNG/IL1B	15
15/108	495/18368	2.04E-07	8.68E-06	5.95E-06	TNF/IFNG/IL1B	15
6/108	46/18368	2.78E-07	1.07E-05	7.37E-06	RELA/TP53/ESR	6
12/108	316/18368	3.48E-07	1.23E-05	8.46E-06	HSPA5/EGFR/RE	12
6/108	52/18368	5.87E-07	1.78E-05	1.22E-05	VDR/STAT3/ESR	6
6/108	52/18368	5.87E-07	1.78E-05	1.22E-05	VDR/STAT3/ESR	6
4/108	14/18368	1.08E-06	3.06E-05	2.10E-05	MAPK14/MAPK1/	4
8/108	135/18368	1.31E-06	3.49E-05	2.39E-05	TNF/BCL2/TP53	8
11/108	297/18368	1.41E-06	3.52E-05	2.42E-05	HSPA5/EGFR/RE	11
8/108	144/18368	2.13E-06	5.04E-05	3.46E-05	IL1B/KDR/EGFR	8
7/108	102/18368	2.33E-06	5.21E-05	3.58E-05	PAH/NOS3/NOS2	7
6/108	72/18368	4.10E-06	8.70E-05	5.97E-05	CXCL8/CXCL10/	6
4/108	20/18368	5.09E-06	0.000103072	7.07E-05	TP53/ESR1/AHR	4
6/108	78/18368	6.54E-06	0.000126431	8.67E-05	RELA/ESR1/AHR	6
8/108	170/18368	7.32E-06	0.000135317	9.28E-05	PTGS2/PAH/NOS	8
8/108	174/18368	8.69E-06	0.000153801	0.00010552	PLAT/MMP9/MMP	8

5/108	49/18368	9.94E-06	0.00016892	0.00011589	TNF/CD40LG/ST	5
5/108	52/18368	1.34E-05	0.000218294	0.00014976	PADI4/NOS3/NC	5
5/108	53/18368	1.47E-05	0.000231026	0.0001585	CAT/NOS3/HMGC	5
8/108	191/18368	1.71E-05	0.000259751	0.00017821	PLAT/MMP9/MMF	8
7/108	139/18368	1.80E-05	0.000264137	0.00018122	PTGS2/CAT/NOS	7
8/108	195/18368	1.99E-05	0.000281541	0.00019316	PLAT/MMP9/MMF	8
7/108	149/18368	2.82E-05	0.000387035	0.00026553	PTGS2/CAT/NOS	7
4/108	31/18368	3.15E-05	0.00040592	0.00027849	TNF/CD40LG/ST	4
3/108	11/18368	3.15E-05	0.00040592	0.00027849	BAK1/BCL2/BCI	3
4/108	32/18368	3.58E-05	0.000447525	0.00030703	RELA/ESR1/AHF	4
11/108	430/18368	4.58E-05	0.000556012	0.00038146	PLAT/MMP9/MMF	11
6/108	126/18368	9.99E-05	0.001179688	0.00080935	HSD17B6/NCF1/	6
4/108	44/18368	0.000128022	0.001470519	0.00100888	AKT1/MAPK14/M	4
9/108	328/18368	0.000137383	0.001536516	0.00105415	TLR4/BAK1/BCI	9
6/108	135/18368	0.000146046	0.001545611	0.00106039	PADI4/VDR/NOS	6
3/108	18/18368	0.000151267	0.001545611	0.00106039	MAPK14/MAPK1/	3
3/108	18/18368	0.000151267	0.001545611	0.00106039	CXCL8/CXCL10/	3
5/108	86/18368	0.000152743	0.001545611	0.00106039	PTGS2/CAT/GSF	5
6/108	141/18368	0.00018517	0.001830173	0.00125562	KDR/EGFR/HAP1	6
4/108	49/18368	0.000195355	0.001886948	0.00129457	CXCL8/CXCL10/	4
6/108	144/18368	0.000207612	0.001960779	0.00134523	VDR/STAT3/ESF	6
6/108	150/18368	0.00025891	0.002392106	0.00164115	RAF1/NCF1/AKT	6
10/108	450/18368	0.00032095	0.002902204	0.00199111	RELA/STAT3/TF	10
10/108	456/18368	0.000356337	0.003155066	0.00216459	RELA/STAT3/TF	10
3/108	25/18368	0.000413785	0.00358895	0.00246226	TP53/STAT1/PC	3
5/108	107/18368	0.000422923	0.003594841	0.00246631	HSPA5/BAK1/TF	5
4/108	61/18368	0.000455834	0.003731097	0.00255979	TP53/IL1A/TYF	4
9/108	386/18368	0.000456511	0.003731097	0.00255979	RAF1/EGFR/AKT	9
5/108	110/18368	0.000480175	0.003850463	0.00264168	RAF1/NCF1/CAS	5
9/108	400/18368	0.000589756	0.004641598	0.00318445	SOAT1/TLR4/FA	9
6/108	176/18368	0.00060655	0.004686974	0.00321558	PADI4/VDR/SEI	6
3/108	29/18368	0.00064624	0.004904498	0.00336482	RELA/NFKBIA/C	3
4/108	70/18368	0.000769115	0.005635758	0.00386651	TP53/ESR1/AHF	4
4/108	70/18368	0.000769115	0.005635758	0.00386651	TP53/ESR1/AHF	4
5/108	127/18368	0.000921861	0.006640527	0.00455585	RELA/TP53/HSF	5
5/108	129/18368	0.000988821	0.007004148	0.00480532	KDR/HSPA5/BAK	5
8/108	360/18368	0.001293476	0.009011926	0.0061828	RAF1/AKT1/MAF	8
5/108	141/18368	0.001469225	0.009925575	0.00680962	IL1B/IL6/IL4/	5
2/108	10/18368	0.001494675	0.009925575	0.00680962	TP53/AHR	2
2/108	10/18368	0.001494675	0.009925575	0.00680962	CASP3/CASP9	2
4/108	85/18368	0.001587814	0.010381861	0.00712266	NOS3/NOS2/GSF	4
3/108	40/18368	0.001667292	0.010736348	0.00736587	CYP3A4/HMOX1/	3
3/108	41/18368	0.001791277	0.011048912	0.00758031	ESR1/PARP1/PC	3
7/108	295/18368	0.001797699	0.011048912	0.00758031	CXCL8/CXCL10/	7
4/108	88/18368	0.00180421	0.011048912	0.00758031	PLAT/MTOR/MAF	4
2/108	11/18368	0.001819821	0.011048912	0.00758031	BCL2/BCL2L1	2
4/108	89/18368	0.001880666	0.011257509	0.00772342	EGFR/ESR1/CAV	4
3/108	42/18368	0.001920875	0.011338496	0.00777898	RELA/TP53/STA	3
3/108	43/18368	0.002056172	0.011970864	0.00821283	NOS3/NOS2/CYF	3
3/108	44/18368	0.002197256	0.012619376	0.00865775	KDR/AHR/HSF1	3
3/108	45/18368	0.002344212	0.013283866	0.00911364	HSP90AA1/APOE	3
3/108	48/18368	0.002821121	0.015776006	0.01082341	CCL2/STAT3/ST	3
2/108	14/18368	0.002976507	0.01630953	0.01118945	GATA3/JUN	2
5/108	166/18368	0.002993278	0.01630953	0.01118945	CXCL10/CXCL11	5

3/108	50/18368	0.003169876	0.017053131	0.01169961	SOAT1/VDAC1/(	3
2/108	15/18368	0.003421287	0.017764628	0.01218775	CAV1/SLC6A4	2
2/108	15/18368	0.003421287	0.017764628	0.01218775	CASP3/CASP9	2
4/108	105/18368	0.003427528	0.017764628	0.01218775	TP53/HSP90AA1	4
7/108	332/18368	0.003474493	0.017791079	0.01220589	KDR/HSPA5/EGI	7
2/108	16/18368	0.003895085	0.019475423	0.01336147	INSR/IGF2	2
2/108	16/18368	0.003895085	0.019475423	0.01336147	NOS3/NOS2	2
2/108	17/18368	0.00439755	0.021732077	0.01490969	IL1B/IL1A	2
6/108	265/18368	0.004786343	0.023381559	0.01604134	CXCL10/SOAT1/	6
3/108	60/18368	0.005304521	0.02533058	0.0173785	FASN/SDHB/SDI	3
3/108	60/18368	0.005304521	0.02533058	0.0173785	SOAT1/VDAC1/(	3
3/108	62/18368	0.005813124	0.027450864	0.01883316	TP53/HSF1/STA	3
2/108	20/18368	0.006073493	0.028365216	0.01946047	RELA/STAT1	2
4/108	124/18368	0.006179762	0.028511796	0.01956103	KDR/EGFR/ERBI	4
8/108	466/18368	0.006239052	0.028511796	0.01956103	NCF1/AKT1/F3/	8
4/108	125/18368	0.006355677	0.028735771	0.0197147	PADI4/CAT/GLS	4
2/108	21/18368	0.006687187	0.029916363	0.02052466	CASP3/FADD	2
2/108	22/18368	0.007327842	0.032106526	0.02202726	HSP90AA1/PCNA	2
2/108	22/18368	0.007327842	0.032106526	0.02202726	APOE/APOB	2
4/108	133/18368	0.007883898	0.032989603	0.02263311	BAK1/HAP1/VDA	4
2/108	23/18368	0.007995127	0.032989603	0.02263311	IGF2/PTPN1	2
2/108	23/18368	0.007995127	0.032989603	0.02263311	ESR1/AHR	2
2/108	23/18368	0.007995127	0.032989603	0.02263311	APOE/APOB	2
2/108	23/18368	0.007995127	0.032989603	0.02263311	JUN/PARP1	2
2/108	23/18368	0.007995127	0.032989603	0.02263311	APOE/APOB	2
4/108	136/18368	0.008514055	0.034793013	0.02387038	KDR/EGFR/ERBI	4
2/108	24/18368	0.008688713	0.035168598	0.02412805	ESR2/ESR1	2
2/108	25/18368	0.009408271	0.037721843	0.02587975	CYP3A4/CYP19A	2
4/108	143/18368	0.010110507	0.040158557	0.02755151	KDR/EGFR/ERBI	4
2/108	27/18368	0.010924019	0.042613226	0.02923558	RAF1/EGFR	2
3/108	78/18368	0.010929039	0.042613226	0.02923558	TP53/PCNA/PTI	3
3/108	79/18368	0.01131267	0.043504149	0.02984681	PML/JUN/PARP1	3
4/108	148/18368	0.01136226	0.043504149	0.02984681	GPT/FASN/VDR/	4
5/108	230/18368	0.011588203	0.043815099	0.03006014	CXCL10/CXCL11	5
2/108	28/18368	0.011719569	0.043815099	0.03006014	APOE/APOB	2
6/108	321/18368	0.011752756	0.043815099	0.03006014	TLR4/RELA/CAT	6
2/108	29/18368	0.012539816	0.046342798	0.03179432	NCF1/AKT1	2
3/108	84/18368	0.013346876	0.048900193	0.03354886	TLR4/INSR/AP(	3
3/108	85/18368	0.013777086	0.050044969	0.03433426	ESR1/AR/GSK3I	3
3/108	86/18368	0.014215132	0.050904115	0.03492369	EGFR/INSR/AR	3
2/108	31/18368	0.014253152	0.050904115	0.03492369	GC/ABCG2	2
2/108	32/18368	0.015145625	0.053640756	0.03680121	ESR1/STAT1	2
4/108	162/18368	0.015384643	0.054036968	0.03707304	IL6/IL4/F2/I(	4
3/108	89/18368	0.015576457	0.054262249	0.0372276	SOAT1/VDAC1/(	3
2/108	33/18368	0.016061562	0.055497267	0.03807491	TLR4/F2	2
2/108	34/18368	0.017000661	0.058268395	0.03997609	BCL2/CAV1	2
2/108	36/18368	0.018947154	0.063405829	0.04350072	APOE/APOB	2
2/108	36/18368	0.018947154	0.063405829	0.04350072	RELA/NFKB1	2
2/108	36/18368	0.018947154	0.063405829	0.04350072	TP53/HSP90AA1	2
2/108	38/18368	0.020982745	0.06966927	0.04779786	CYP3A4/CYP19A	2
2/108	39/18368	0.022033227	0.072590088	0.04980174	CYP3A4/CYP19A	2
