# Supplementary material for: Real-World Study on Chai-Shi-Jie-Du Granules for the Treatment of Dengue Fever and the Possible Mechanisms Based on Network Pharmacology
Source: Evid Based Complement Alternat Med. 2023 Aug 30;2023:9942842. doi: 10.1155/2023/9942842 (PMC10482559; doi:10.1155/2023/9942842)
Supplement: Supplementary Materials — Table S1: duration of symptoms and signs between the CSJD and control groups. Table S2: drug-active ingredient-target database. Table S3: the GO enrichment results for the 108 cotargets. Table S4: the KEGG enrichment results for the 108 cotargets. Figure S1: the PPI network of 108 potential therapeutic target proteins. Figure S2: the “drug-active compound-therapeutic target protein” network of the effect of CSJD on dengue fever. Red represents the most important potential therapeutic targets, and orange represents the less important potential therapeutic targets; other potential therapeutic targets are colored yellow. Green represents the active compound. Purple represents the constituent drugs of CSJD. The edges represent the relationship between constituent drugs, active compounds, and potential therapeutic target proteins. [file 9942842.f1.zip › Table S4. The KEGG enrichment results for the 108 cotargets.pdf]

ID	Description	GeneRatio	BgRatio
hsa04933	AGE-RAGE signaling pathway in diabetic complications	27/106	100/8112
hsa05417	Lipid and atherosclerosis	34/106	215/8112
hsa05418	Fluid shear stress and atherosclerosis	29/106	139/8112
hsa04657	IL-17 signaling pathway	23/106	94/8112
hsa04668	TNF signaling pathway	24/106	112/8112
hsa05164	Influenza A	26/106	171/8112
hsa05161	Hepatitis B	23/106	162/8112
hsa04066	HIF-1 signaling pathway	20/106	109/8112
hsa05215	Prostate cancer	19/106	97/8112
hsa05142	Chagas disease	19/106	102/8112
hsa04620	Toll-like receptor signaling pathway	19/106	104/8112
hsa05162	Measles	21/106	139/8112
hsa05140	Leishmaniasis	17/106	77/8112
hsa04659	Th17 cell differentiation	19/106	108/8112
hsa05145	Toxoplasmosis	19/106	112/8112
hsa05167	Kaposi sarcoma-associated herpesvirus infection	23/106	194/8112
hsa05133	Pertussis	16/106	76/8112
hsa05212	Pancreatic cancer	16/106	76/8112
hsa05163	Human cytomegalovirus infection	23/106	225/8112
hsa05152	Tuberculosis	21/106	180/8112
hsa05208	Chemical carcinogenesis - reactive oxygen species	22/106	223/8112
hsa01522	Endocrine resistance	16/106	98/8112
hsa04210	Apoptosis	18/106	136/8112
hsa05160	Hepatitis C	19/106	157/8112
hsa04621	NOD-like receptor signaling pathway	20/106	184/8112
hsa04917	Prolactin signaling pathway	14/106	70/8112
hsa04625	C-type lectin receptor signaling pathway	16/106	104/8112
hsa05235	PD-L1 expression and PD-1 checkpoint pathway in cancer	15/106	89/8112
hsa05022	Pathways of neurodegeneration - multiple diseases	29/106	476/8112
hsa05321	Inflammatory bowel disease	13/106	65/8112
hsa05210	Colorectal cancer	14/106	86/8112
hsa04660	T cell receptor signaling pathway	15/106	104/8112
hsa05131	Shigellosis	21/106	247/8112
hsa04926	Relaxin signaling pathway	16/106	129/8112
hsa05169	Epstein-Barr virus infection	19/106	202/8112
hsa04932	Non-alcoholic fatty liver disease	17/106	155/8112
hsa05205	Proteoglycans in cancer	19/106	205/8112
hsa05171	Coronavirus disease - COVID-19	20/106	232/8112
hsa04151	PI3K-Akt signaling pathway	24/106	354/8112
hsa05135	Yersinia infection	16/106	137/8112
hsa01521	EGFR tyrosine kinase inhibitor resistance	13/106	79/8112
hsa05170	Human immunodeficiency virus 1 infection	19/106	212/8112
hsa05144	Malaria	11/106	50/8112
hsa04936	Alcoholic liver disease	16/106	142/8112
hsa05132	Salmonella infection	20/106	249/8112
hsa04064	NF-kappa B signaling pathway	14/106	104/8112
hsa05130	Pathogenic Escherichia coli infection	18/106	197/8112
hsa05219	Bladder cancer	10/106	41/8112
hsa04380	Osteoclast differentiation	15/106	128/8112
hsa05010	Alzheimer disease	24/106	384/8112
hsa04931	Insulin resistance	14/106	108/8112
hsa05415	Diabetic cardiomyopathy	18/106	203/8112
hsa05134	Legionellosis	11/106	57/8112

hsa04658	Th1 and Th2 cell differentiation	13/106	92/8112
hsa05207	Chemical carcinogenesis - receptor activation	18/106	212/8112
hsa04218	Cellular senescence	15/106	156/8112
hsa05223	Non-small cell lung cancer	11/106	72/8112
hsa05020	Prion disease	19/106	273/8112
hsa05222	Small cell lung cancer	12/106	92/8112
hsa01524	Platinum drug resistance	11/106	73/8112
hsa05213	Endometrial cancer	10/106	58/8112
hsa04071	Sphingolipid signaling pathway	13/106	119/8112
hsa04722	Neurotrophin signaling pathway	13/106	119/8112
hsa04010	MAPK signaling pathway	19/106	294/8112
hsa04217	Necroptosis	14/106	159/8112
hsa05230	Central carbon metabolism in cancer	10/106	70/8112
hsa04062	Chemokine signaling pathway	15/106	192/8112
hsa04915	Estrogen signaling pathway	13/106	138/8112
hsa05166	Human T-cell leukemia virus 1 infection	16/106	222/8112
hsa05323	Rheumatoid arthritis	11/106	93/8112
hsa05220	Chronic myeloid leukemia	10/106	76/8112
hsa05224	Breast cancer	13/106	147/8112
hsa04370	VEGF signaling pathway	9/106	59/8112
hsa01523	Antifolate resistance	7/106	30/8112
hsa04068	FoxO signaling pathway	12/106	131/8112
hsa04012	ErbB signaling pathway	10/106	85/8112
hsa04215	Apoptosis - multiple species	7/106	32/8112
hsa05221	Acute myeloid leukemia	9/106	67/8112
hsa04910	Insulin signaling pathway	12/106	137/8112
hsa05165	Human papillomavirus infection	18/106	331/8112
hsa04920	Adipocytokine signaling pathway	9/106	69/8112
hsa05225	Hepatocellular carcinoma	13/106	168/8112
hsa04622	RIG-I-like receptor signaling pathway	9/106	70/8112
hsa05120	Epithelial cell signaling in Helicobacter pylori infec	9/106	70/8112
hsa00220	Arginine biosynthesis	6/106	22/8112
hsa05143	African trypanosomiasis	7/106	37/8112
hsa05206	MicroRNAs in cancer	17/106	310/8112
hsa05146	Amoebiasis	10/106	102/8112
hsa04613	Neutrophil extracellular trap formation	13/106	190/8112
hsa04662	B cell receptor signaling pathway	9/106	82/8112
hsa04630	JAK-STAT signaling pathway	12/106	162/8112
hsa04371	Apelin signaling pathway	11/106	139/8112
hsa04930	Type II diabetes mellitus	7/106	46/8112
hsa04510	Focal adhesion	13/106	201/8112
hsa04664	Fc epsilon RI signaling pathway	8/106	68/8112
hsa04935	Growth hormone synthesis, secretion and action	10/106	119/8112
hsa05226	Gastric cancer	11/106	149/8112
hsa04919	Thyroid hormone signaling pathway	10/106	121/8112
hsa05214	Glioma	8/106	75/8112
hsa05202	Transcriptional misregulation in cancer	12/106	192/8112
hsa04014	Ras signaling pathway	13/106	232/8112
hsa05014	Amyotrophic lateral sclerosis	16/106	364/8112
hsa05168	Herpes simplex virus 1 infection	19/106	495/8112
hsa04211	Longevity regulating pathway	8/106	89/8112
hsa04912	GnRH signaling pathway	8/106	93/8112
hsa05231	Choline metabolism in cancer	8/106	98/8112
hsa05218	Melanoma	7/106	72/8112

hsa04610	Complement and coagulation cascades	7/106	85/8112
hsa04072	Phospholipase D signaling pathway	9/106	148/8112
hsa04213	Longevity regulating pathway - multiple species	6/106	62/8112
hsa04623	Cytosolic DNA-sensing pathway	6/106	63/8112
hsa05012	Parkinson disease	12/106	266/8112
hsa04929	GnRH secretion	6/106	64/8112
hsa05332	Graft-versus-host disease	5/106	42/8112
hsa04520	Adherens junction	6/106	71/8112
hsa04061	Viral protein interaction with cytokine and cytokine r	7/106	100/8112
hsa05203	Viral carcinogenesis	10/106	204/8112
hsa04914	Progesterone-mediated oocyte maturation	7/106	102/8112
hsa04115	p53 signaling pathway	6/106	73/8112
hsa04060	Cytokine-cytokine receptor interaction	12/106	295/8112
hsa04928	Parathyroid hormone synthesis, secretion and action	7/106	106/8112
hsa04140	Autophagy - animal	8/106	141/8112
hsa04024	cAMP signaling pathway	10/106	221/8112
hsa04921	Oxytocin signaling pathway	8/106	154/8112
hsa04150	mTOR signaling pathway	8/106	155/8112
hsa05416	Viral myocarditis	5/106	60/8112
hsa00250	Alanine, aspartate and glutamate metabolism	4/106	37/8112
hsa05216	Thyroid cancer	4/106	37/8112
hsa05330	Allograft rejection	4/106	38/8112
hsa04022	cGMP-PKG signaling pathway	8/106	167/8112
hsa04650	Natural killer cell mediated cytotoxicity	7/106	131/8112
hsa04916	Melanogenesis	6/106	101/8112
hsa05211	Renal cell carcinoma	5/106	69/8112
hsa05016	Huntington disease	11/106	306/8112
hsa04940	Type I diabetes mellitus	4/106	43/8112
hsa04137	Mitophagy - animal	5/106	72/8112
hsa04550	Signaling pathways regulating pluripotency of stem cel	7/106	143/8112
hsa04670	Leukocyte transendothelial migration	6/106	114/8112
hsa01200	Carbon metabolism	6/106	115/8112
hsa04726	Serotonergic synapse	6/106	115/8112
hsa04979	Cholesterol metabolism	4/106	50/8112
hsa04152	AMPK signaling pathway	6/106	120/8112
hsa04611	Platelet activation	6/106	124/8112
hsa04015	Rap1 signaling pathway	8/106	210/8112
hsa05310	Asthma	3/106	31/8112
hsa04666	Fc gamma R-mediated phagocytosis	5/106	97/8112
hsa04640	Hematopoietic cell lineage	5/106	99/8112
hsa04960	Aldosterone-regulated sodium reabsorption	3/106	37/8112
hsa01230	Biosynthesis of amino acids	4/106	75/8112
hsa04612	Antigen processing and presentation	4/106	78/8112
hsa04141	Protein processing in endoplasmic reticulum	6/106	171/8112
hsa04672	Intestinal immune network for IgA production	3/106	49/8112
hsa05030	Cocaine addiction	3/106	49/8112
hsa04540	Gap junction	4/106	88/8112
hsa04913	Ovarian steroidogenesis	3/106	51/8112
hsa05410	Hypertrophic cardiomyopathy	4/106	90/8112
hsa04350	TGF-beta signaling pathway	4/106	94/8112
hsa04923	Regulation of lipolysis in adipocytes	3/106	56/8112
hsa04020	Calcium signaling pathway	7/106	240/8112
hsa05017	Spinocerebellar ataxia	5/106	143/8112
hsa04730	Long-term depression	3/106	60/8112

hsa04723	Retrograde endocannabinoid signaling	5/106	148/8112
hsa00140	Steroid hormone biosynthesis	3/106	61/8112
hsa04261	Adrenergic signaling in cardiomyocytes	5/106	150/8112
hsa05217	Basal cell carcinoma	3/106	63/8112

pvalue	p.adjust	qvalue	geneID	Count
3.64E-29	9.22E-27	3.37E-27	TNF/IL1B/IL6/CXCL8	27
2.17E-28	2.75E-26	1.01E-26	TNF/IL1B/IL6/CXCL8	34
8.82E-28	7.44E-26	2.72E-26	TNF/IFNG/IL1B/THBI	29
9.02E-24	5.71E-22	2.09E-22	TNF/IFNG/IL1B/IL6/	23
2.73E-23	1.38E-21	5.07E-22	TNF/IL1B/IL6/CXCL1	24
3.63E-21	1.53E-19	5.60E-20	TNF/IFNG/IL1B/IL6/	26
4.57E-18	1.46E-16	5.33E-17	TNF/IL6/CXCL8/RAF1	23
4.61E-18	1.46E-16	5.33E-17	IFNG/IL6/TLR4/EGFI	20
9.25E-18	2.60E-16	9.52E-17	RAF1/EGFR/RELA/BCI	19
2.52E-17	6.38E-16	2.33E-16	TNF/IFNG/IL1B/IL6/	19
3.70E-17	8.52E-16	3.12E-16	TNF/IL1B/IL6/CXCL8	19
4.11E-17	8.66E-16	3.17E-16	IL1B/IL6/TLR4/REL/	21
6.48E-17	1.26E-15	4.62E-16	TNF/IFNG/IL1B/IL4/	17
7.80E-17	1.41E-15	5.16E-16	IFNG/IL1B/IL6/IL4/	19
1.59E-16	2.69E-15	9.83E-16	TNF/IFNG/TLR4/CD4(	19
2.78E-16	4.39E-15	1.61E-15	IL6/CXCL8/RAF1/PT(	23
1.28E-15	1.80E-14	6.59E-15	TNF/IL1B/IL6/CXCL8	16
1.28E-15	1.80E-14	6.59E-15	RAF1/EGFR/RELA/BAI	16
7.47E-15	9.94E-14	3.64E-14	TNF/IL1B/IL6/CXCL8	23
8.97E-15	1.14E-13	4.16E-14	TNF/IFNG/IL1B/IL6/	21
6.57E-14	7.91E-13	2.90E-13	RAF1/EGFR/RELA/CA1	22
8.89E-14	1.02E-12	3.74E-13	RAF1/EGFR/BCL2/TP5	16
9.73E-14	1.05E-12	3.83E-13	TNF/RAF1/RELA/BAK1	18
9.92E-14	1.05E-12	3.83E-13	TNF/IFNG/RAF1/CXCI	19
1.65E-13	1.67E-12	6.11E-13	TNF/IL1B/IL6/CXCL8	20
1.85E-13	1.80E-12	6.60E-13	RAF1/RELA/STAT3/ES	14
2.33E-13	2.19E-12	8.01E-13	TNF/IL1B/IL6/RAF1/	16
3.45E-13	3.11E-12	1.14E-12	IFNG/RAF1/TLR4/EGI	15
1.10E-12	9.56E-12	3.50E-12	TNF/IL1B/IL6/RAF1/	29
1.43E-12	1.20E-11	4.41E-12	TNF/IFNG/IL1B/IL6/	13
3.67E-12	2.90E-11	1.06E-11	RAF1/EGFR/BAK1/BCI	14
3.67E-12	2.90E-11	1.06E-11	TNF/IFNG/IL4/RAF1/	15
4.87E-12	3.73E-11	1.37E-11	TNF/IL1B/CXCL8/TLI	21
7.19E-12	5.35E-11	1.96E-11	RAF1/EGFR/RELA/AK1	16
9.68E-12	7.00E-11	2.56E-11	TNF/IL6/CXCL10/REI	19
1.13E-11	7.95E-11	2.91E-11	TNF/IL1B/IL6/CXCL8	17
1.26E-11	8.60E-11	3.15E-11	TNF/RAF1/KDR/TLR4/	19
1.30E-11	8.66E-11	3.17E-11	TNF/IL1B/IL6/CXCL8	20
1.55E-11	1.00E-10	3.67E-11	IL6/IL4/RAF1/KDR/1	24
1.84E-11	1.16E-10	4.25E-11	TNF/IL1B/IL6/CXCL8	16
1.97E-11	1.22E-10	4.46E-11	IL6/RAF1/KDR/EGFR/	13
2.28E-11	1.37E-10	5.02E-11	TNF/RAF1/TLR4/REL/	19
2.73E-11	1.61E-10	5.89E-11	TNF/IFNG/IL1B/IL6/	11
3.19E-11	1.84E-10	6.72E-11	TNF/IL1B/IL6/CXCL8	16
4.76E-11	2.68E-10	9.79E-11	TNF/IL1B/IL6/CXCL8	20
5.30E-11	2.91E-10	1.07E-10	TNF/IL1B/CXCL8/PT(	14
5.89E-11	3.17E-10	1.16E-10	TNF/IL1B/IL6/CXCL8	18
7.55E-11	3.98E-10	1.46E-10	CXCL8/RAF1/EGFR/TI	10
7.87E-11	4.07E-10	1.49E-10	TNF/IFNG/IL1B/REL/	15
8.57E-11	4.33E-10	1.59E-10	TNF/IL1B/IL6/RAF1/	24
8.92E-11	4.43E-10	1.62E-10	TNF/IL6/RELA/PRKAI	14
9.72E-11	4.73E-10	1.73E-10	RELA/NCF1/AKT1/NOS	18
1.25E-10	5.98E-10	2.19E-10	TNF/IL1B/IL6/CXCL8	11

1.45E-10	6.80E-10	2.49E-10	IFNG/IL4/RELA/NFKI	13
2.00E-10	9.19E-10	3.36E-10	RAF1/EGFR/VDR/RELA	18
1.33E-09	6.01E-09	2.20E-09	IL6/CXCL8/RAF1/REI	15
1.74E-09	7.74E-09	2.83E-09	RAF1/EGFR/BAK1/STA	11
1.78E-09	7.75E-09	2.84E-09	TNF/IL1B/IL6/HSPA5	19
2.00E-09	8.56E-09	3.14E-09	PTGS2/RELA/BAK1/BC	12
2.03E-09	8.56E-09	3.14E-09	BAK1/BCL2/TP53/AKT	11
2.92E-09	1.21E-08	4.44E-09	RAF1/EGFR/BAK1/TP5	10
3.80E-09	1.52E-08	5.58E-09	TNF/RAF1/RELA/BCL2	13
3.80E-09	1.52E-08	5.58E-09	RAF1/RELA/BCL2/TP5	13
6.10E-09	2.41E-08	8.83E-09	TNF/IL1B/RAF1/KDR/	19
1.55E-08	6.03E-08	2.21E-08	TNF/IFNG/IL1B/TLR4	14
1.95E-08	7.48E-08	2.74E-08	RAF1/EGFR/TP53/AKT	10
2.33E-08	8.69E-08	3.18E-08	CXCL8/RAF1/CXCL10/	15
2.34E-08	8.69E-08	3.18E-08	RAF1/EGFR/BCL2/ESI	13
2.40E-08	8.79E-08	3.22E-08	TNF/IL6/RELA/TP53/	16
2.80E-08	1.01E-07	3.71E-08	TNF/IFNG/IL1B/IL6/	11
4.40E-08	1.57E-07	5.74E-08	RAF1/RELA/BAK1/TP5	10
5.00E-08	1.76E-07	6.43E-08	RAF1/EGFR/BAK1/TP5	13
5.75E-08	1.99E-07	7.30E-08	RAF1/PTGS2/KDR/AKT	9
8.47E-08	2.90E-07	1.06E-07	TNF/IL1B/IL6/RELA/	7
1.15E-07	3.87E-07	1.42E-07	IL6/RAF1/EGFR/PRKA	12
1.31E-07	4.35E-07	1.59E-07	RAF1/EGFR/AKT1/ERI	10
1.37E-07	4.50E-07	1.65E-07	BAK1/BCL2/CASP3/FA	7
1.80E-07	5.82E-07	2.13E-07	RAF1/RELA/STAT3/AI	9
1.89E-07	6.04E-07	2.21E-07	RAF1/FASN/PRKAB1/A	12
2.26E-07	7.16E-07	2.62E-07	TNF/RAF1/PTGS2/EGI	18
2.33E-07	7.27E-07	2.66E-07	TNF/RELA/PRKAB1/ST	9
2.43E-07	7.48E-07	2.74E-07	RAF1/EGFR/BAK1/TP5	13
2.64E-07	7.96E-07	2.91E-07	TNF/CXCL8/CXCL10/I	9
2.64E-07	7.96E-07	2.91E-07	CXCL8/EGFR/RELA/NI	9
2.72E-07	8.09E-07	2.96E-07	GPT/NOS3/GLS/NOS2/	6
3.97E-07	1.17E-06	4.28E-07	TNF/IFNG/IL1B/IL6/	7
4.55E-07	1.32E-06	4.84E-07	RAF1/PTGS2/EGFR/BA	17
7.40E-07	2.13E-06	7.79E-07	TNF/IFNG/IL1B/IL6/	10
1.00E-06	2.85E-06	1.04E-06	RAF1/PADI4/TLR4/RI	13
1.05E-06	2.94E-06	1.08E-06	RAF1/RELA/AKT1/NFI	9
1.16E-06	3.23E-06	1.18E-06	IFNG/IL6/IL4/RAF1/	12
1.74E-06	4.79E-06	1.76E-06	RAF1/PRKAB1/PLAT/S	11
1.88E-06	5.09E-06	1.86E-06	TNF/INSR/MTOR/MAPI	7
1.89E-06	5.09E-06	1.86E-06	RAF1/KDR/EGFR/BCL2	13
2.52E-06	6.70E-06	2.45E-06	TNF/IL4/RAF1/AKT1/	8
3.08E-06	8.10E-06	2.97E-06	RAF1/STAT3/AKT1/ST	10
3.45E-06	9.01E-06	3.30E-06	RAF1/EGFR/BAK1/BCI	11
3.58E-06	9.24E-06	3.38E-06	RAF1/TP53/ESR1/AKT	10
5.33E-06	1.36E-05	4.98E-06	RAF1/EGFR/BAK1/TP5	8
6.90E-06	1.74E-05	6.39E-06	IL6/CXCL8/RELA/BAI	12
9.21E-06	2.31E-05	8.45E-06	RAF1/KDR/EGFR/RELA	13
1.78E-05	4.40E-05	1.61E-05	TNF/HSPA5/BCL2/CAT	16
1.84E-05	4.52E-05	1.65E-05	TNF/IFNG/IL1B/IL6/	19
1.92E-05	4.66E-05	1.71E-05	RELA/PRKAB1/CAT/TI	8
2.65E-05	6.38E-05	2.34E-05	RAF1/EGFR/MMP2/MAI	8
3.88E-05	9.24E-05	3.38E-05	RAF1/EGFR/AKT1/JUN	8
3.91E-05	9.24E-05	3.38E-05	RAF1/EGFR/BAK1/TP5	7

0.000114	0.00026705	9.78E-05	THBD/PLAT/F2/F3/SI	7
0.00012802	0.00029716	0.0001088	CXCL8/RAF1/EGFR/AI	9
0.00014668	0.00033737	0.0001235	PRKAB1/CAT/AKT1/IN	6
0.00016042	0.00036564	0.0001339	IL1B/IL6/CXCL10/RI	6
0.00017049	0.00038513	0.000141	HSPA5/TP53/NFE2L2/	12
0.00017516	0.00039216	0.0001436	RAF1/SPP1/ESR2/AKT	6
0.0002007	0.00044541	0.0001631	TNF/IFNG/IL1B/IL6/	5
0.00031078	0.00068365	0.0002503	EGFR/ERBB2/INSR/MA	6
0.00031502	0.00068365	0.0002503	TNF/IL6/CXCL8/CXCI	7
0.00031615	0.00068365	0.0002503	RELA/BAK1/STAT3/TI	10
0.00035575	0.00076276	0.0002793	RAF1/AKT1/HSP90AA	7
0.00036169	0.00076898	0.0002815	BCL2/TP53/CASP3/SI	6
0.0004416	0.00093104	0.0003409	TNF/IFNG/IL1B/IL6/	12
0.00044988	0.00094065	0.0003444	RAF1/EGFR/VDR/BCL2	7
0.00049111	0.00101845	0.0003729	RAF1/BCL2/AKT1/MT(	8
0.00059583	0.00122557	0.0004487	RAF1/RELA/AKT1/NFI	10
0.00088008	0.00179564	0.0006574	RAF1/PTGS2/EGFR/PI	8
0.00091807	0.00185817	0.0006803	TNF/RAF1/AKT1/INSI	8
0.0010698	0.00214808	0.0007865	CD40LG/ICAM1/CASP3	5
0.00130564	0.00258068	0.0009449	GPT/GLS/ASS1/CPS1	4
0.00130564	0.00258068	0.0009449	BAK1/TP53/MAPK1/MA	4
0.00144473	0.00283345	0.0010374	TNF/IFNG/IL4/CD40I	4
0.00148553	0.00289108	0.0010585	RAF1/AKT1/NOS3/INS	8
0.00157891	0.00304934	0.0011165	TNF/IFNG/RAF1/ICAM	7
0.00200621	0.00381682	0.0013975	RAF1/EDN1/TYR/MAPI	6
0.00200647	0.00381682	0.0013975	RAF1/AKT1/JUN/MAPI	5
0.00209544	0.00395632	0.0014485	HAP1/TP53/CASP3/SI	11
0.00229772	0.0043061	0.0015766	TNF/IFNG/IL1B/IL1/	4
0.00242207	0.00450577	0.0016497	RELA/TP53/JUN/BCL2	5
0.00260317	0.00480732	0.0017601	RAF1/STAT3/AKT1/MA	7
0.00368431	0.00675456	0.0024731	VCAM1/NCF1/MMP9/MN	6
0.00384666	0.00695147	0.0025452	GPT/CAT/SDHB/SDHA/	6
0.00384666	0.00695147	0.0025452	RAF1/PTGS2/CASP3/N	6
0.00399887	0.00717529	0.0026271	SOAT1/VDAC1/APOE/A	4
0.00473934	0.00844403	0.0030916	FASN/PRKAB1/AKT1/I	6
0.00555672	0.00983113	0.0035995	AKT1/NOS3/F2/MAPK1	6
0.00607349	0.01067079	0.0039069	RAF1/KDR/EGFR/AKT1	8
0.00747335	0.01303972	0.0047743	TNF/IL4/CD40LG	3
0.00863466	0.0149628	0.0054784	RAF1/NCF1/AKT1/MAI	5
0.00938915	0.01615956	0.0059165	TNF/IL1B/IL6/IL4/I	5
0.01220951	0.02087165	0.0076418	INSR/MAPK1/MAPK3	3
0.0164751	0.02797451	0.0102424	GPT/PAH/ASS1/CPS1	4
0.01877185	0.03166185	0.0115924	TNF/IFNG/HSPA5/HSI	4
0.02426385	0.040654	0.0148848	HSPA5/BAK1/BCL2/NI	6
0.02587704	0.04279014	0.0156669	IL6/IL4/CD40LG	3
0.02587704	0.04279014	0.0156669	RELA/NFKB1/JUN	3
0.02781681	0.04569904	0.0167319	RAF1/EGFR/MAPK1/MA	4
0.0287096	0.04686148	0.0171575	PTGS2/INSR/CYP19A1	3
0.0298891	0.04847399	0.0177479	TNF/IL6/PRKAB1/EDN	4
0.03430217	0.05527674	0.0202386	TNF/IFNG/MAPK1/MAI	4
0.0364804	0.05841481	0.0213876	PTGS2/AKT1/INSR	3
0.03734347	0.05942075	0.0217559	KDR/EGFR/ERBB2/NOS	7
0.03901072	0.0616857	0.0225852	AKT1/VDAC1/MTOR/MA	5
0.04339633	0.06819424	0.0249681	RAF1/MAPK1/MAPK3	3

0.04415772	0.06896236	0.0252494	PTGS2/MAPK14/MAPK1	5
0.0452208	0.07018933	0.0256986	HSD17B6/CYP3A4/CYI	3
0.04632575	0.07146595	0.026166	BCL2/AKT1/MAPK14/M	5
0.0489826	0.07510665	0.027499	BAK1/TP53/GSK3B	3
